# Recurrent Plant-Specific Duplications of KNL2 and its Conserved Function as a Kinetochore Assembly Factor

**DOI:** 10.1093/molbev/msac123

**Published:** 2022-06-07

**Authors:** Sheng Zuo, Ramakrishna Yadala, Fen Yang, Paul Talbert, Joerg Fuchs, Veit Schubert, Ulkar Ahmadli, Twan Rutten, Ales Pecinka, Martin A Lysak, Inna Lermontova

**Affiliations:** Central European Institute of Technology (CEITEC), Masaryk University, Kamenice 5, CZ-625 00 Brno, Czech Republic; National Centre for Biomolecular Research, Faculty of Science, Masaryk University, Kamenice 5, CZ-625 00 Brno, Czech Republic; Leibniz Institute of Plant Genetics and Crop Plant Research (IPK) Gatersleben, Corrensstrasse 3, D-06466 Seeland, Germany; Institute of Experimental Botany, Czech Acad Sci, Centre of the Region Haná for Biotechnological and Agricultural Research, Šlechtitelů 31, 779 00 Olomouc, Czech Republic; Department of Cell Biology and Genetics, Faculty of Science, Palacký University, Šlechtitelů 27, 779 00 Olomouc, Czech Republic; Howard Hughes Medical Institute, Basic Sciences Division, Fred Hutchinson Cancer Research Center, Seattle, WA 98109, USA; Leibniz Institute of Plant Genetics and Crop Plant Research (IPK) Gatersleben, Corrensstrasse 3, D-06466 Seeland, Germany; Leibniz Institute of Plant Genetics and Crop Plant Research (IPK) Gatersleben, Corrensstrasse 3, D-06466 Seeland, Germany; Leibniz Institute of Plant Genetics and Crop Plant Research (IPK) Gatersleben, Corrensstrasse 3, D-06466 Seeland, Germany; Leibniz Institute of Plant Genetics and Crop Plant Research (IPK) Gatersleben, Corrensstrasse 3, D-06466 Seeland, Germany; Institute of Experimental Botany, Czech Acad Sci, Centre of the Region Haná for Biotechnological and Agricultural Research, Šlechtitelů 31, 779 00 Olomouc, Czech Republic; Department of Cell Biology and Genetics, Faculty of Science, Palacký University, Šlechtitelů 27, 779 00 Olomouc, Czech Republic; Central European Institute of Technology (CEITEC), Masaryk University, Kamenice 5, CZ-625 00 Brno, Czech Republic; National Centre for Biomolecular Research, Faculty of Science, Masaryk University, Kamenice 5, CZ-625 00 Brno, Czech Republic; Central European Institute of Technology (CEITEC), Masaryk University, Kamenice 5, CZ-625 00 Brno, Czech Republic; Leibniz Institute of Plant Genetics and Crop Plant Research (IPK) Gatersleben, Corrensstrasse 3, D-06466 Seeland, Germany

**Keywords:** adaptive evolution, CENH3, centromere, endopolyploidy, gene duplication, kinetochore, KNL2

## Abstract

KINETOCHORE NULL2 (KNL2) plays key role in the recognition of centromeres and new CENH3 deposition. To gain insight into the origin and diversification of the *KNL2* gene, we reconstructed its evolutionary history in the plant kingdom. Our results indicate that the *KNL2* gene in plants underwent three independent ancient duplications in ferns, grasses, and eudicots. Additionally, we demonstrated that previously unclassified *KNL2* genes could be divided into two clades *αKNL2* and *βKNL2* in eudicots and *γKNL2* and *δKNL2* in grasses, respectively. *KNL2*s of all clades encode the conserved SANTA domain, but only the *αKNL2* and *γKNL2* groups additionally encode the CENPC-k motif. In the more numerous eudicot sequences, signatures of positive selection were found in both *αKNL2* and *βKNL2* clades, suggesting recent or ongoing adaptation. The confirmed centromeric localization of βKNL2 and mutant analysis suggests that it participates in loading of new CENH3, similarly to αKNL2. A high rate of seed abortion was found in heterozygous *βknl2* plants and the germinated homozygous mutants did not develop beyond the seedling stage. Taken together, our study provides a new understanding of the evolutionary diversification of the plant kinetochore assembly gene *KNL2*, and suggests that the plant-specific duplicated *KNL2* genes are involved in centromere and/or kinetochore assembly for preserving genome stability.

## Introduction

Centromeres are specific chromosomal regions where kinetochore protein complexes assemble in mitosis and meiosis to attach chromosomes to the spindle microtubules, and thus, are responsible for accurate segregation of chromosomes. Loss of centromere and kinetochore function causes chromosome missegregation, aneuploidy, and cell death (Fachinetti et al. 2013; McKinley and Cheeseman 2016; Barra and Fachinetti 2018). Centromere identity is specified epigenetically by the presence of the histone H3 variant termed CENH3 (also named CENP-A in mammals) which triggers the assembly of a functional kinetochore (Talbert et al. 2002). The kinetochore complexes are formed by dozens of proteins including the constitutive centromere-associated network complexes and outer kinetochore modules (Cheeseman and Desai 2008; Musacchio and Desai 2017; Hara and Fukagawa 2018).

KINETOCHORE NULL2 (KNL2, also termed M18BP1; Moree et al. 2011; Lermontova et al. 2013) plays a key role in new CENH3 deposition after replication. In vertebrates, M18BP1 (KNL2) is part of the Mis18 complex, including additionally Mis18α and Mis18β proteins. However, Mis18α and Mis18β in plants have not yet been identified. The human Mis18 complex is transiently present at centromeres prior to new CENH3 incorporation (Fujita et al. 2007); in chicken and *Xenopus*, the M18BP1 protein is present at centromeres throughout the cell cycle (French et al. 2017; Hori et al. 2017). In plants, KNL2 localizes at centromeres through the cell cycle, except from metaphase to late anaphase (Lermontova et al. 2013). The KNL2 proteins identified so far contain the characteristic SANTA (SANT-associated) domain (Zhang et al. 2006), a protein module of ∼90 amino acids which in some organisms is accompanied by a SANT/Myb-like putative DNA-binding domain. The functional role of SANTA and SANT domains has remained obscure for a long time. For instance, an interaction of KNL2 homologues containing the SANT/Myb domain with DNA has not yet been demonstrated, while *Arabidopsis thaliana* KNL2, which lacks this domain, showed DNA-binding capability *in vitro* and an association with the centromeric repeat *PAL1*  *in vivo* (Sandmann et al. 2017). Deletion of the SANTA domain in *Arabidopsis* KNL2 has not impaired its targeting to centromeres (Lermontova et al. 2013) nor disrupted its interaction with DNA (Sandmann et al. 2017). In *Xenopus*, a direct interaction of M18BP1 with CENH3 nucleosomes also did not require the SANTA domain (French et al. 2017). However, M18BP1 localizes at centromeres during metaphase—prior to CENH3 loading—by binding to CENP-C using the SANTA domain (French and Straight 2019).

A conserved CENPC-k motif, which is highly similar to the previously described CENPC motif of the CENP-C protein (Sugimoto et al. 1994; Talbert et al. 2004; Kato et al. 2013), was identified on the C-terminal part of the KNL2 homologues in a wide spectrum of eukaryotes (Kral 2016; Sandmann et al. 2017). The importance of this domain for the centromeric targeting of KNL2 was demonstrated in *Arabidopsis* (Sandmann et al. 2017), *Xenopus* (French et al. 2017), and chicken (Hori et al. 2017). Moreover, direct binding of CENPC-k to CENH3 nucleosomes was shown (French et al. 2017; Hori et al. 2017). In *Xenopus*, KNL2, similar to CENP-C, recruits the CENH3 chaperone HJURP to centromeres for new CENH3 assembly, and CENP-C competes with KNL2 for binding new CENH3 at centromeres (French et al. 2017). KNL2 in eutherian mammals lacks a CENPC-k motif (Kral 2016; Sandmann et al. 2017), and centromeric localization of human KNL2 may be achieved by direct binding of the SANTA domain to CENP-C (French and Straight 2019). Depletion of KNL2 in different organisms causes defects in CENH3 assembly (Fujita et al. 2007; Lermontova et al. 2013; French et al. 2017). For instance, knockout of M18BP1 as well as other components of the Mis18 complex in human HeLa cells with RNAi abolished centromeric recruitment of newly synthesized CENP-A, leading to chromosome missegregation and interphase micronuclei (Fujita et al. 2007). Embryos of homozygous mis18α mutant of mouse showed decreased DNA methylation, increased centromeric transcription, misaligned chromosomes, anaphase bridges, and lagging chromosomes, which was accompanied by embryo lethality (Kim et al. 2012). Unlike in mammals, the homozygous *knl2* mutant of *Arabidopsis* is viable despite reduced CENH3 levels and mitotic and meiotic abnormalities resulting in reduced growth rate and fertility (Lermontova et al. 2013). The fact that in the *knl2* mutant CENH3 is still localized at the centromeres suggests that this is not the only mechanism responsible for the centromeric loading of CENH3 in plants.

Although the functions of KNL2 are gradually being uncovered, research is still limited to a few model species, and in particular, the precise molecular mechanism of KNL2 interaction remains to be clarified. Up to now, robust phylogenetic analyses of the *KNL2* gene across large evolutionary time scales have not been reported. A better understanding of *KNL2* evolution may yield important insights into its role in CENH3 deposition and kinetochore assembly. To reconstruct the evolutionary history of the *KNL2* gene in plants, we compiled a data set of the proteins encoded by *KNL2* genes across major plant lineages from available genomic resources. Our phylogenetic analyses indicate that the *KNL2* gene in plants underwent three independent ancient duplications in ferns, grasses, and eudicots. We show that previously unclassified *KNL2* genes in eudicots could be divided into two clades (*αKNL2* and *βKNL2*). Both clades encode the conserved SANTA domain, but only the *αKNL2* group additionally encodes the conserved CENPC-k motif. Signatures of positive selection were found in both clades. Two additional *KNL2* clades (*γKNL2* and *δKNL2*) were identified in grasses. Similar to the divergence of αKNL2 and βKNL2 proteins, γKNL2 proteins retain the CENPC-k motif, while δKNL2 proteins have a shortened motif that resembles part of CENPC-k. In addition, analysis of RNA-seq data in *Arabidopsis* shows the *βKNL2* gene expression in nearly all tissues is considerably higher than the expression of *αKNL2*. Moreover, we provide the first evidence that βKNL2 localizes to centromeric regions in *Arabidopsis*. Mutant analysis of βKNL2 suggests that it participates in the loading of new CENH3 similarly to αKNL2. Taken together, our study provides a new understanding of the evolutionary origin and function of plant-specific duplicated KNL2 as a kinetochore assembly factor.

## Results

### Search for *KNL2* Genes in Plants Led to the Finding and Re-annotation of a New *KNL2* Variant in *Arabidopsis*

The KNL2 protein contains a conserved module designated as SANTA due to its association with the SANT domain. Although most metazoans have only one gene coding for a SANTA domain-containing protein, two genes (*At5g02520* and *At1g58210*) were identified in *Arabidopsis* (Zhang et al. 2006). Since the predicted protein encoded by the *At1g58210* gene contained in addition to the SANTA domain, a protein interaction kinase domain 1 (KIP1) and the C-terminal chromosome maintenance structural domain (SMC_Prok_B), completely atypical for previously described KNL2 proteins, we had previously excluded it from our research and focused on *At5g02520* (Lermontova et al. 2013).

**Fig. 1.**
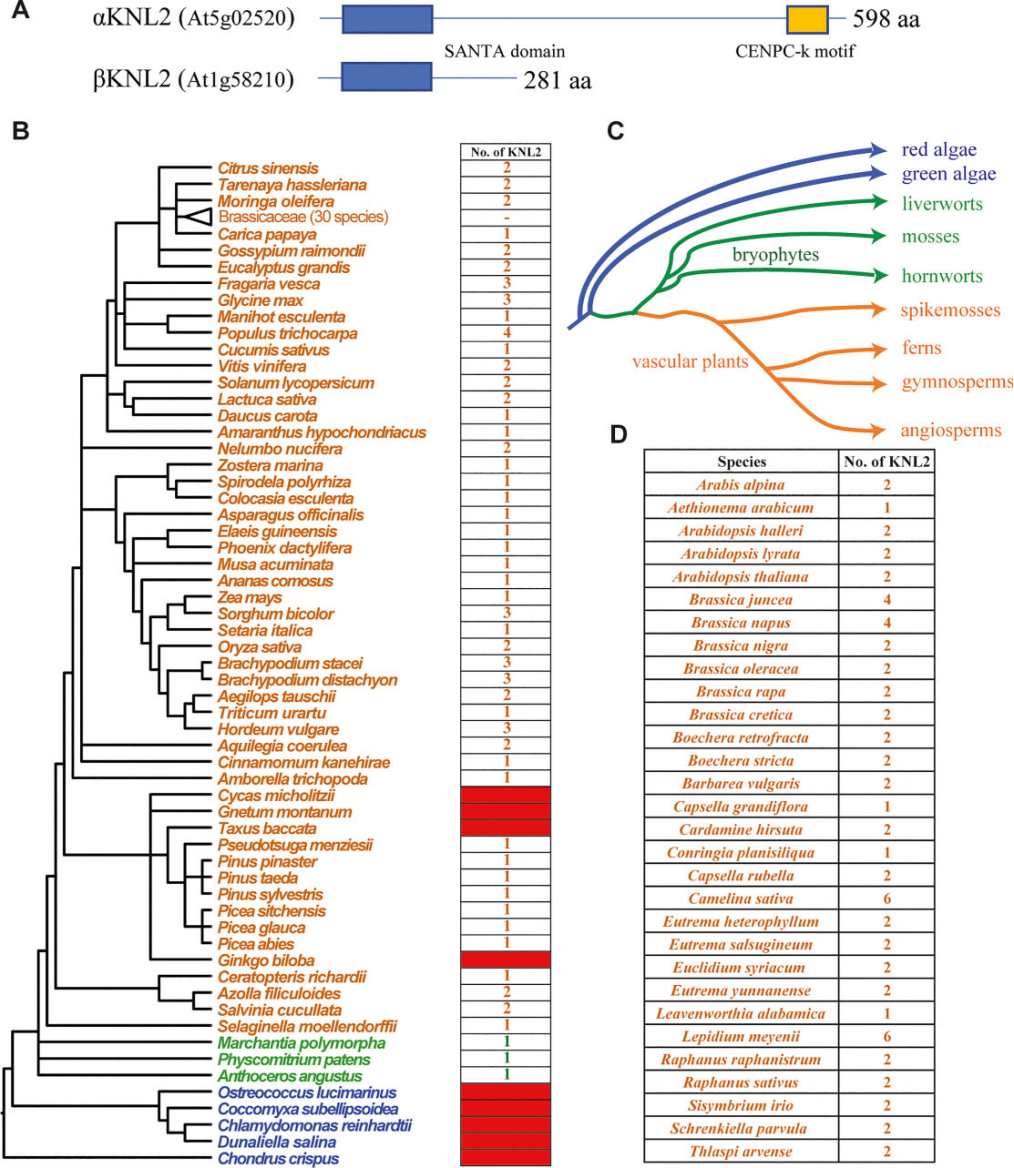
Identification of the *KNL2* gene homologs across major plant lineages. (*A*) Protein structure of KNL2 in *Arabidopsis*. SANTA domain and CENPC-k motif are indicated by differently colored boxes. (*B*) The number of *KNL2* homologs in 90 representative plant species. The phylogenetic tree is adopted from the NCBI common tree. The blue-, green-, and orange-colored species names indicate alga, bryophytes, and vascular plants, respectively. The red filled boxes mean that we could not retrieved *KNL2* from these species. (*C*) Phylogenetic relationships of the analyzed species were adapted from Banks et al. (2011). (*D*) The number of *KNL2* homologs identified in analyzed crucifer (Brassicaceae) genomes.

However, based on the updated Araport-11 annotation (TAIR and Phytozome 13 database) and our in silico analysis, we found that the *At1g58210* gene encodes a protein of 281 amino acids including the SANTA domain but excluding KIP1 and SMC_Prok_B. We designated it as βKNL2 and the previously characterized KNL2 as αKNL2 (fig. 1*A*), in which full-length alpha and beta KNL2 have only 41.5% identity.

To investigate the origin and evolution of *KNL2* genes, we constructed a comprehensive proteome data set across major plant lineages including 90 representative species (fig. 1*B,C*). We performed a genome-wide search using the *Arabidopsis* αKNL2 (*At5g02520*) amino acid sequence and its conserved domains as the query for a local BLASTP search against the data set (supplementary fig. S1, Supplementary Material online). In total, 148 homologous conceptual protein sequences encoded by *KNL2* genes were identified in plant lineages including bryophytes (3 species:3 sequences), lycophytes (1:1), ferns (3:5), gymnosperms (7:7), and angiosperm species (67:132; fig. 1*B,D*; supplementary table S1and file S1, Supplementary Material online). For lycophytes, the *KNL2* gene was retrieved by TBLASTN search from *Selaginella moellendorffii* genome. Comparison with genomic and cDNA sequences in *S. moellendorffii* revealed that there is an intron right in the CENPC-k motif (supplementary file S2, Supplementary Material online). While the *KNL2* gene was detected in all investigated angiosperm species and ferns, it has not been identified in 4 out of 11 gymnosperm species investigated (*Cycas micholitzii*, *Ginkgo biloba*, *Gnetum montanum*, and *Taxus baccata*). The failure to find KNL2 in these species is likely because of incompletely assembled proteomes of gymnosperms at the time they were downloaded from the PLAZA genome database, not because of its absence in their genomes. Additionally, the *KNL2* gene also was not retrieved in any of the five algal species we examined. Based on the quality of the assembled algal proteomes (Merchant et al. 2007; Blanc et al. 2012; Collen et al. 2013), the *KNL2* gene may be absent in these genomes. However, we cannot exclude the possibility that *KNL2* has diverged beyond recognition by BLASTP and tBLASTN in algal genomes. In summary, the *KNL2* genes experienced recurrent ancient plant-specific duplication events.

### *KNL2* Gene in Plants Underwent Independent Duplications in Ferns, Grasses, and Eudicots

**Fig. 2.**
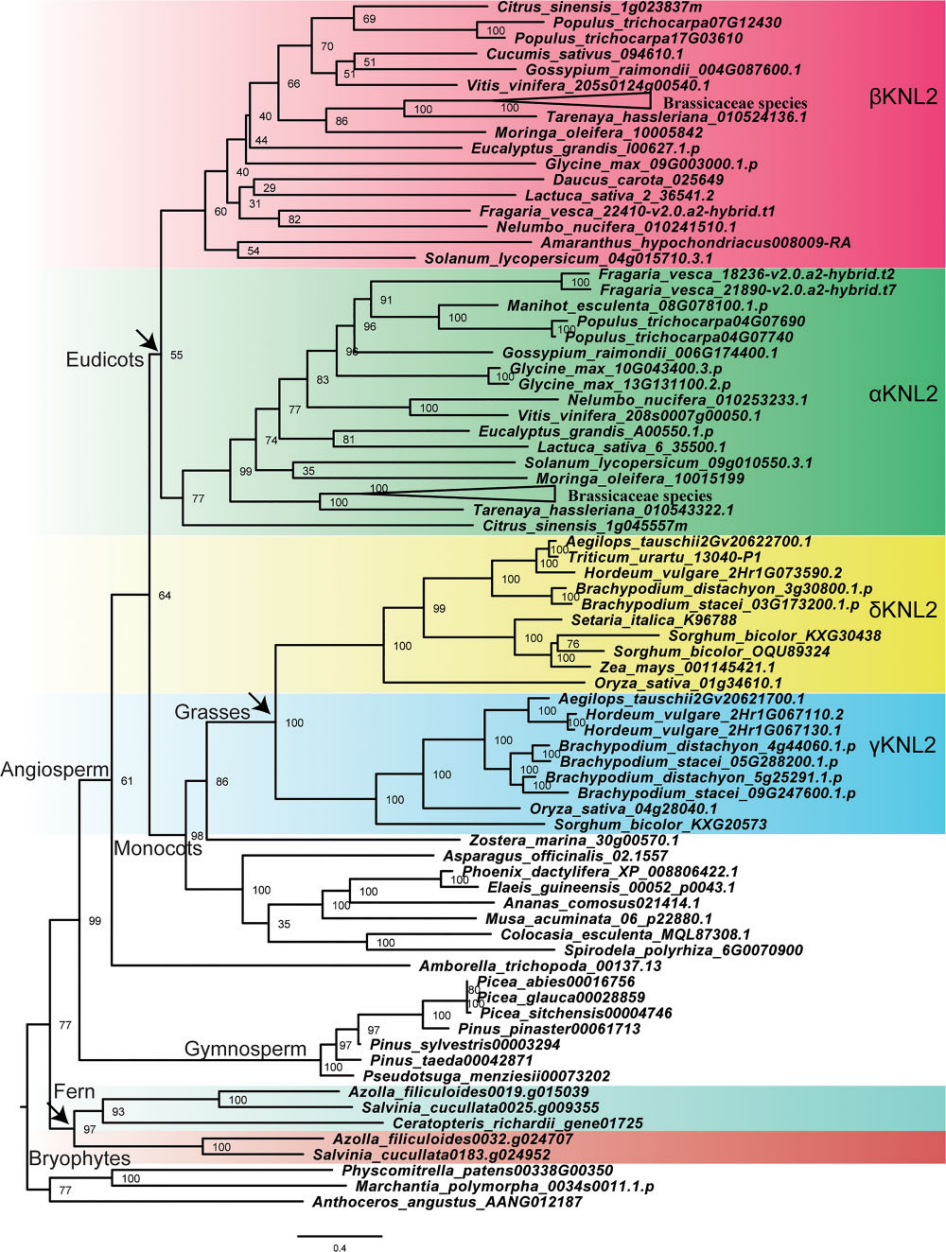
Evolutionary relationship of KNL2 homologs in land plants. Maximum likelihood phylogenetic analysis was performed using IQ-tree with a protein alignment of KNL2 homologs in land plants. The *KNL2* genes cluster into two branches in three plant clades—heterosporous water ferns (Salviniaceae), eudicots, and grasses (Poaceae)—indicating ancient gene duplications (arrows). The KNL2 in eudicots and grasses can be classified into two major groups (αKNL2 and βKNL2, and γKNL2 and δKNL2, respectively). Bootstrap values obtained after 1,000 ultrafast bootstrap replicates (bb) are shown in the tree. The scale bar indicates the number of substitutions per site. The tree is arbitrarily rooted between bryophytes and tracheophytes.

To better understand the *KNL2* gene diversification and evolution across the plant kingdom, we made a multiple sequence alignment of KNL2 proteins (supplementary file S3, Supplementary Material online) and constructed a phylogenetic tree. The topology of the Maximum Likelihood (ML) tree (fig. 2) shows that KNL2 proteins cluster into two branches in three plant clades—heterosporous water ferns (Salviniaceae), eudicots, and grasses (Poaceae)—indicating ancient gene duplications. Despite the deep divergence of the duplicated paralogs in ferns, their CENPC-k motifs are 83% identical. The grouping of a KNL2 protein of *Ceratopteris*, a member of the Polypodiales encompassing ∼80% of fern species, with one of the two KNL2 proteins of water ferns suggests that the duplication of *KNL2* in ferns occurred prior to the divergence of Salviniales and Polypodiales, more than 120 Ma (Qi et al. 2018). In angiosperms, gene duplication occurred after the divergence of *Amborella trichopoda* and monocots, but prior to the divergence of the basal eudicot *Nelumbo nucifera*, estimated at ∼100 Ma (Angiosperm Phylogeny website: http://www.mobot.org/MOBOT/research/APweb/; Friis et al. 2016). This duplication gave rise to the α*KNL2* and *βKNL2* genes of *Arabidopsis* and their orthologs in other eudicots. Monocots except for grasses (Poaceae) appear to have only one *KNL2* gene copy, while two paralogs in grasses indicate another gene duplication in the grass ancestor ∼100 Ma (Wu et al. 2018). In light of their separate origin from *αKNL2* and *βKNL2* in eudicots, these two paralogous copies in grasses were named γ*KNL2* and δ*KNL2*.

### The αKNL2 and βKNL2 Paralogs Contain the SANTA Domain, but only αKNL2 is Characterized by the Presence of the C-terminal CENPC-k motif

Next, we focused on the α*KNL2* and *βKNL2* genes and their proteins mainly in Brassicales due to the extensive availability of genomic resources (supplementary fig. S2, supplementary file S4, Supplementary Material online). Except for a few neopolyploid species, the *αKNL2* and *βKNL2* gene numbers are conserved at one copy each across Brassicales species. These KNL2 proteins present several conserved features: the N-terminus contains the conserved SANTA domain in all KNL2 proteins, whereas only the αKNL2-type C-terminus possesses the CENPC-k motif. αKNL2 and βKNL2 sequences identified from Brassicales showed 41.0 and 57.2% pairwise identity, respectively.

**Fig. 3.**
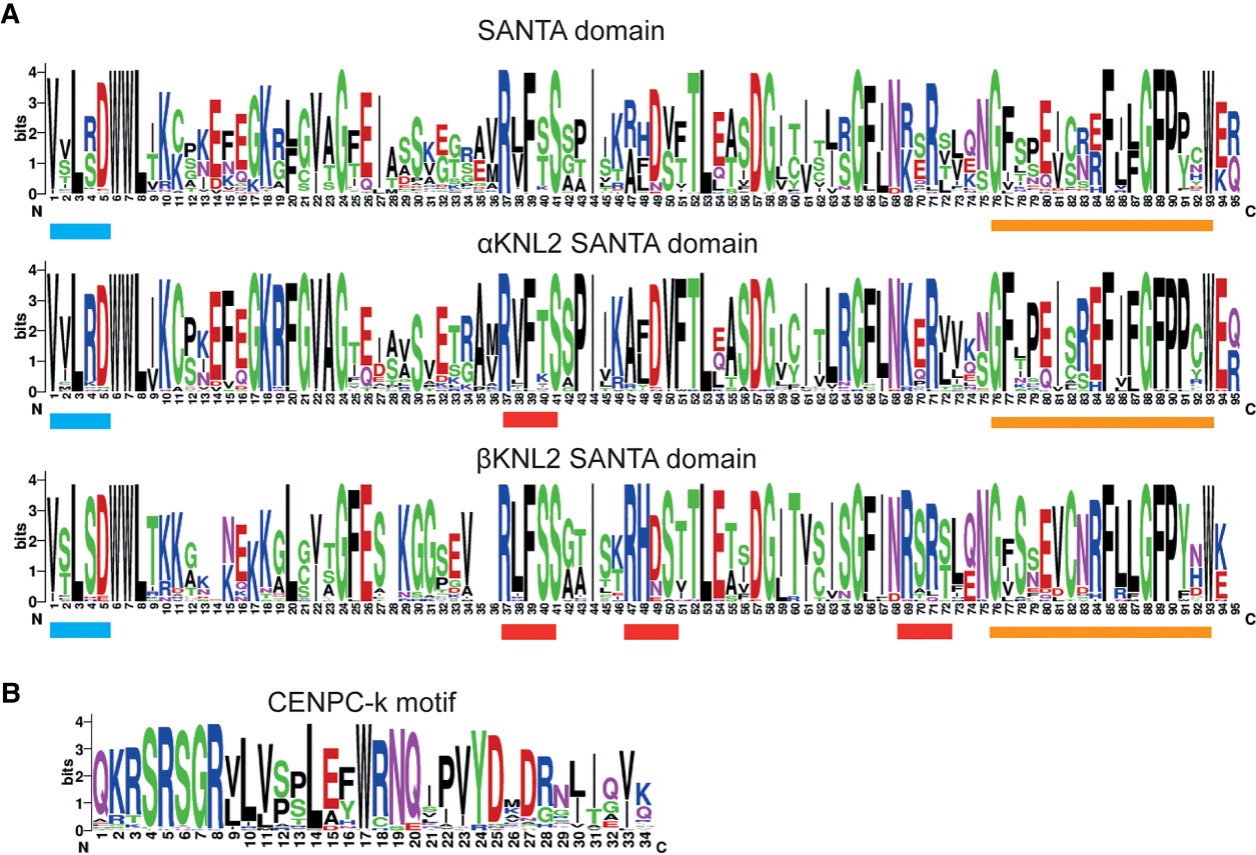
Alignments of SANTA domain and CENPC-k motif in KNL2 homologs presented in LOGO format. (*A*) Variation map of the SANTA domain in the KNL2 homologs. The WebLogo program (http://weblogo.berkeley.edu/logo.cgi) was used to present SANTA domain alignments. The upper panel aligns SANTA domains of all KNL2 homologs from Brassicales, whereas the middle and bottom panels represent SANTA domain alignments of αKNL2 and βKNL2 homologs, respectively. The conserved N-terminal and C-terminal hydrophobic motifs are marked by blue and orange bars, respectively. Putative Aurora kinase phosphorylation consensus sites are underlined with red bars. (*B*) Alignment of CENPC-k motif of KNL2 homologs from land plants.

We aligned all SANTA domains in KNL2 homologs from Brassicales species to show the conservation and variation and also made separate alignments for the SANTA domains in αKNL2 and βKNL2 paralogs (fig. 3*A*). The alignment results showed that SANTA domains from Brassicales species have 55.0% pairwise identity, while the similarity of these domains within αKNL2 paralogs is 71.0% and within βKNL2 paralogs is 72.3%, respectively. Many residues in the SANTA domains are conserved between both αKNL2 and βKNL2 paralogs. However, there are also amino acids specific to αKNL2 or βKNL2, suggesting that they might have different functions or interact with different proteins. For instance, one putative Aurora kinase phosphorylation consensus ((*R*/*K*)*X*_1-3_(*S*/*T*)) can be detected in αKNL2 (fig. 3*A*, middle panel, aa 37–41) and three in βKNL2 (fig. 3*A*, lower panel, aa 37–41, 47–50, 69–72). In addition, we aligned SANTA domains from angiosperm species (minus Brassicales) and early diverging land plants (supplementary fig. S3, Supplementary Material online). As expected, SANTA domain variation increased with the phylogenetic divergence through evolutionary time. However, SANTA domains from nearly all paralogs maintain the previously identified conserved hydrophobic residues at the N- and C-termini, including the VxLxDW motif at the N-terminus of the SANTA domain and the GFxxxxxxxFxxGFPxxW motif at the C-terminus (Zhang et al. 2006).

In contrast to the SANTA domain, the CENPC-k motif is highly conserved throughout the plant kingdom where it is present (fig. 3*B*); however, the CENPC-k motif is missing from the βKNL2 and δKNL2 clades. Given that αKNL2 and βKNL2 paralogs may have been retained to perform distinct functions, we looked for additional conserved motifs in both variants from Brassicales species using the Multiple Em for Motif Elicitation (MEME) tool. Besides the motifs preserved in SANTA and CENPC-k regions (fig. 3), we also identified several additional conserved motifs that are unique to one or the other paralog (supplementary fig. S4, Supplementary Material online). For example, the N-termini of βKNL2 paralogs have a conserved motif 7 (21 aa), which is located upstream of the SANTA domain, but absent in αKNL2 paralogs (supplementary fig. S4, Supplementary Material online).

### The KNL2 of Maize is Represented only by the δKNL2 Variant with a Truncated CENPC-k Motif

To observe the conserved features of KNL2, we also examined the γ*KNL2* and δ*KNL2* genes in grasses. γ*KNL2* encodes a SANTA domain and CENPC-k motif (supplementary file S5, Supplementary Material online), while *δKNL2* encodes a SANTA domain and the motif RRLRSGKV/I, which resembles a truncated version of the CENPC-k motif (supplementary file S6, Supplementary Material online). γKNL2 and δKNL2 sequences from grasses showed 41.4 and 37.8% pairwise identity, respectively. Other non-grass monocot species only have one *KNL2* gene copy (fig. 2 and supplementary table S1, Supplementary Material online), and these single-copy *KNL2* genes more closely resemble the γ clade, encoding SANTA and CENPC-k motif, which is the ancestral state of *KNL2* before the grass-specific gene duplication. Interestingly, in eight reference proteomes of maize, we found only one copy of the *KNL2* gene, though with several splicing variants (supplementary fig. S5, Supplementary Material online). We also checked maize transcriptome data from different tissues and developmental stages; however, only *δKNL2* was identified (Maize RNA-seq Database: http://ipf.sustech.edu.cn/pub/zmrna/). We propose that unlike in other grass species, the maize genome contains only one copy of the δ*KNL2* gene and has lost γ*KNL2*.

### Different Evolutionary Forces act on KNL2 Paralogs

**Fig. 4.**
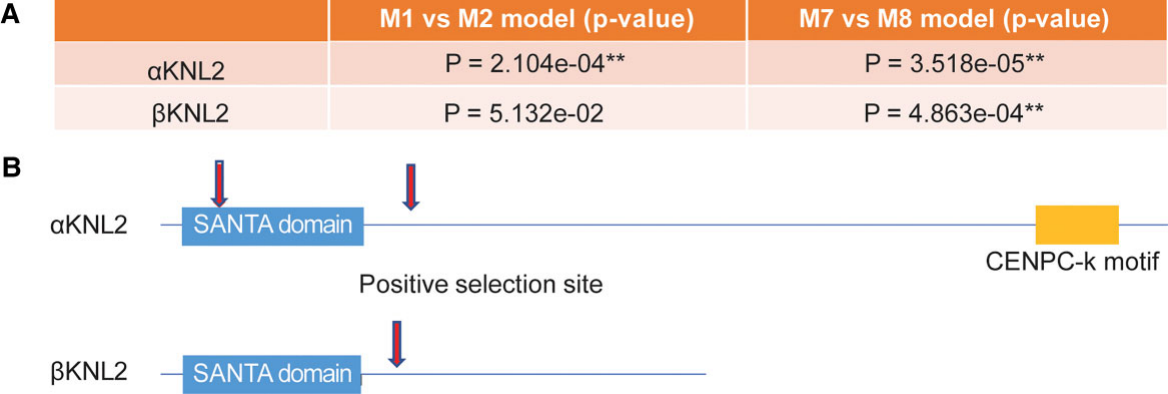
Evolutionary pressures on the KNL2 paralogs. (*A*) Summary of tests for positive selection performed on KNL2 paralogs from Brassicaceae species. Statistically significant tests (*P* < 0.05) are indicated with asterisks. (*B*) A schematic of a representative KNL2 protein, showing sites evolving under positive selection identified by Bayes empirical Bayes analysis (posterior probability > 0.95).

We considered the possibility that selection may act differently on KNL2 paralogs. We used ML methods using the PAML suite (Yang 2007) to test for positive selection on each of the KNL2 paralogs in Brassicaceae species (supplementary file S7, Supplementary Material online). The branch-site model was used to test two KNL2 groups by using Codeml program (Yang 2007). Our PAML analyses revealed positive selection on both αKNL2 (fig. 4*A*, M1 vs. M2, *P* = 2.104 × 10^−4^ and M7 vs. M8, *P* = 3.518 × 10^−5^) and βKNL2 paralogs (M7 vs. M8, *P* = 4.863 × 10^−4^). Bayes empirical Bayes analyses identified two amino acids in αKNL2 paralogs and one amino acid in βKNL2 paralogs as having evolved under positive selection with a high posterior probability (>0.95, fig. 4*B*). In αKNL2, the two positively selected sites are located in and slightly C-terminal to the SANTA domain (fig. 4*B*, supplementary fig. S6, Supplementary Material online). In βKNL2, the positively selected site also is located slightly C-terminal to the SANTA domain (fig. 4*B*, supplementary fig. S6, Supplementary Material online).

### βKNL2 of *Arabidopsis* shows Centromeric Localization

**Fig. 5.**
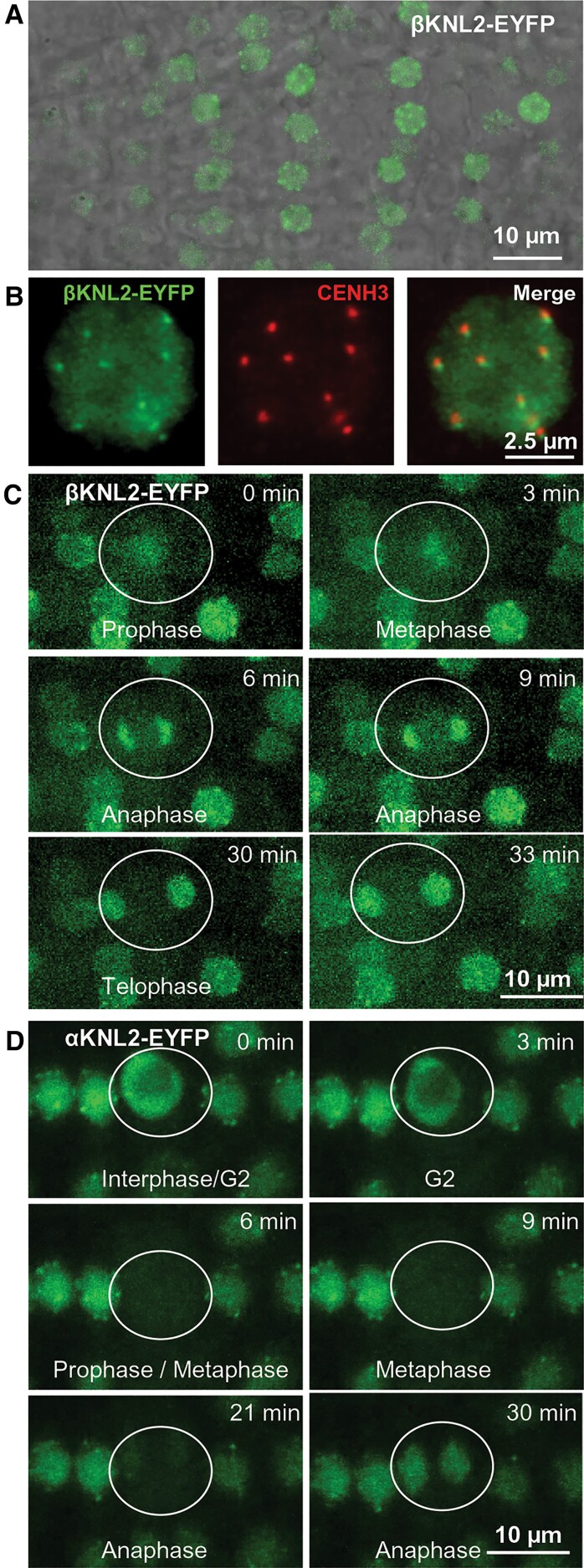
Subcellular localization of βKNL2 in *Arabidopsis*. (*A*) Live imaging of root tip cells of *Arabidopsis* transformed with the βKNL2-EYFP and αKNL2-EYFP fusion constructs. Fluorescent signals showed distinct centromeric and diffused nucleoplasmic distribution. (*B*) Nucleus isolated from seedlings of the βKNL2-EYFP transformants after immunostaining with anti-GFP (left panel) and anti-CENH3 (middle panel) antibodies. Merge of both immunosignals (right panel). (*C*) Live imaging of root tip cells of *Arabidopsis* transformed with the βKNL2-EYFP fusion construct. (*D*) Live imaging of root tip cells of *Arabidopsis* transformed with the αKNL2-EYFP fusion construct. Cell undergoing mitosis is encircled.

We assessed the subcellular localization and putative biological function of the *Arabidopsis* βKNL2 variant *in vivo*. To this end, the *βKNL2* cDNA was cloned into the pDONR221 vector and subcloned into pGWB641 (35Spro, C-EYFP) and pGWB642 (35Spro, N-EYFP) vector, respectively. In *Arabidopsis*, seedlings stably transformed with the βKNL2 fused to EYFP, fluorescent signals were detected at centromeres and in the nucleoplasm of the root tip nuclei (fig. 5*A–C*). An immunostaining experiment with anti-GFP and anti-CENH3 antibodies revealed the colocalization of βKNL2-EYFP with CENH3 at centromeres (fig. 5*B*). Live cell imaging of mitotic cells showed that βKNL2 is present at centromeres during interphase, almost not detectable shortly prior to mitosis, but appears again during the M phase (fig. 5*C*). In contrast, αKNL2 was not detectable during prophase, metaphase, and early anaphase in *Arabidopsis* root tip cells (fig. 5*D*; Lermontova et al. 2013).

### In all Selected Meristematic Tissues, the Expression Level of *βKNL2* is Higher than that of *αKNL2*

**Fig. 6.**
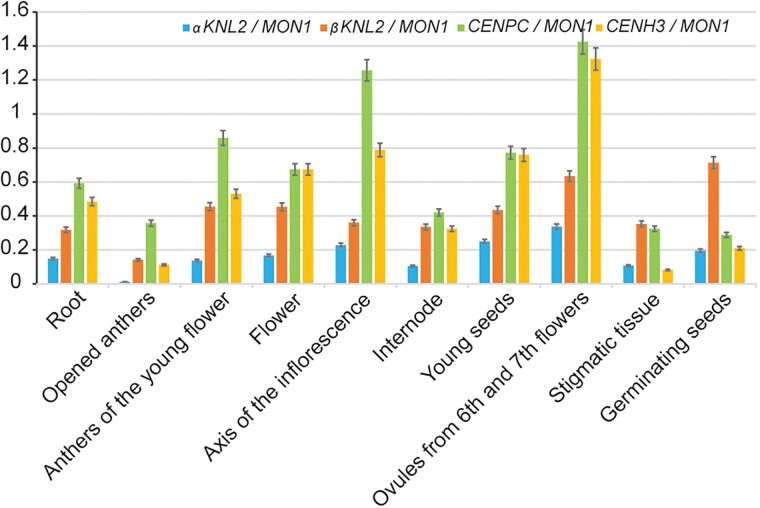
The *CENH3*, *CENP-C*, and *KNL2* gene expression profiles in *Arabidopsis*. Column charts showing different expression levels of the *CENH3*, *CENP-C*, and *KNL2* genes in tissues enriched for dividing cells. The relative fragments per kilobase of exon per million mapped fragments (RPKM) values of *CENH3*, *CENP-C*, and *KNL2* were normalized to the reference gene *MON1* (*At2g28390*) in RNA-seq data sets. The corresponding gene id numbers are: *CENH3* (*At1g01370*), *CENP-C* (*At1g15660*), *αKNL2* (*At5g02520*), and *βKNL2* (*At1g58210*).

To investigate the expression profiles of the *KNL2* genes in different tissues and developmental stages and to compare them with *CENH3* and *CENP-C*, we downloaded the available RNA-seq data in *Arabidopsis* from a public database (Klepikova et al. 2016) and additionally performed expression analysis using the eFP genome browser. In the eFP genome browser analysis, *βKNL2* was excluded from the analysis due to the mis-annotation and consequent lack of correct gene expression data, while we used the correct *βKNL2* annotation for our RNA-seq data analysis. The expression value of selected genes was normalized to the reference gene *MONENSIN SENSITIVITY1* (*MON1*; *At2g28390*) which shows stable transcription during plant development (Czechowski et al. 2005). The data showed that the *KNL2*, *CENH3*, and *CENP-C* genes have high transcriptional activity in tissues enriched for meristematically active cells (fig. 6, supplementary fig. S7, Supplementary Material online), indicating the involvement of these genes in cell division processes. In contrast, a low expression level of the selected genes was observed in the rosette and senescent leaves (supplementary fig. S7, Supplementary Material online). In general, the *CENP-C* and *CENH3* genes show higher expression than *KNL2*. Interestingly, the *βKNL2* has higher expression level than *αKNL2* in nearly all tissues.

### *βKNL2* Knockout Resulted in an Abnormal Seed Development and Semilethal Mutant Phenotype

**Fig. 7.**
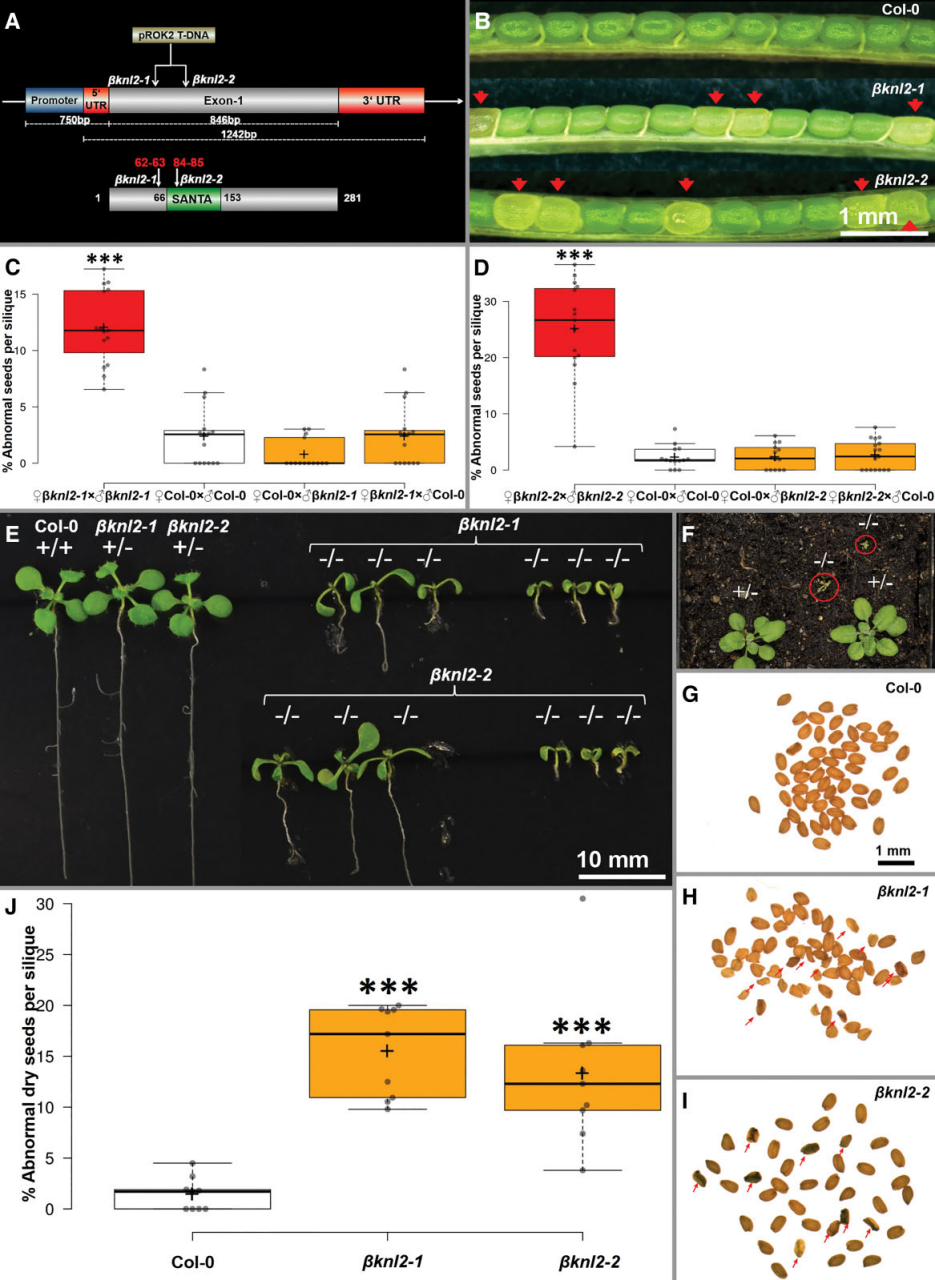
Identification and primary analysis of *βknl2* mutant. (*A*) Schematic representation of the T-DNA insertion position in the genomic fragment and protein with the position of the SANTA domain. (*B*) Representative siliques with red arrowheads showing abnormal whitish glossy-seed phenotype from heterozygous *βknl2-1* and *βknl2-2* plants. (*C,D*) Boxplots depicting the number of abnormal seeds per silique data from the reciprocal crossing of WT and heterozygous *βknl2-1* and *βknl2-2* (***P ≤ 0.001). (*E*) Two weeks old *in vitro* germinated seedlings from Col-0, *βknl2-1*, and *βknl2-2* heterozygous (+/−) and homozygous mutants (−/−). (*F*) *βknl2* homozygous (−/−) and heterozygous (+/−) mutants on soil, homozygous mutants turning yellow in the red circle. (*G–I*) Representative dry seeds of Col-0, *βknl2-1*, and *βknl2-2.* Red arrowheads indicate the abnormal seeds. (*J*) Boxplot depicting the significant increase of abnormal dry seeds per silique of heterozygous *βknl2-1* and *βknl2-2* compared with WT as control.

To characterize and understand the *βKNL2* function, two T-DNA insertion lines SALK_135778 and SALK_091054 were identified and defined as *βknl2-1* and *βknl2-2*, respectively (fig. 7*A*). Both T-DNA insertions are present in the single exon of *βKNL2*, 270 and 335 nucleotides downstream from the transcription start. Thus, in *βknl2-1*, the T-DNA insertion is located upstream and in *βknl2-2* directly in the region encoding the SANTA domain (fig. 7*A*). Polymerase chain reaction (PCR)-based genotyping of soil-grown plants revealed no homozygous mutant lines in either mutant population obtained from the ABRC seed stock (*n* = 26 and *n* = 38, respectively) or in the next generation (*n* = 195 and *n* = 220, respectively). This suggested that the *βKNL2* knockout might be lethal.

Therefore, siliques of both mutants were tested for the seed phenotype. Heterozygous *βknl2* mutant lines show 11 ± 1% (supplementary fig. S8, Supplementary Material online) of abnormal seeds (*P* ≤ 0.01), which look larger and whitish with glossy surface compared with normal green seeds (fig. 7*B*), whereas in the case of wild-type (WT) plants no such seeds were found. However, unlike *βknl2-2*, the *βknl2-1* mutant exhibited an ovule abortion phenotype (supplementary fig. S9, Supplementary Material online). The SALK_135778 (*βknl2-1*) line carries two additional T-DNA insertions in the *AT1G76850* and *AT3G13920* genes according to the ABRC database (https://abrc.osu.edu/stocks/618439). Furthermore, these two genes affect ovule development and pollen acceptance. The corresponding mutations cause an ovule lethal phenotype (Bush et al. 2015; Safavian et al. 2015). Therefore, we speculated that the ovule lethality found in *βknl2-1* might be due to these off-target mutations. Using primers specific to these additional T-DNA insertions, we selected clean *βknl2-1* plants carrying single T-DNA. Indeed, resulting *βknl2-1* lines did not show the aborted ovule phenotype and were selected for further analysis (fig. 7*B*). To assess whether the heterozygous or homozygous state of mutation causes the abnormal seed phenotype and maternal or paternal effects during embryogenesis, reciprocal crosses between WT and heterozygous *βknl2-1* and *βknl2-2* mutants were performed. All these crosses produced <3% of abnormal seeds (fig. 7*C,D* and supplementary table S2, Supplementary Material online) which is similar to the frequency observed in WT self-pollinated siliques. These findings indicate that the appearance of abnormal seeds in the siliques of heterozygous mutants is not the result of defective female gamete formation, but is rather due to defects during postzygotic development. The fact that the abnormal seeds were increased only in self-pollinated heterozygous mutants (fig. 7*C,D*, supplementary table S2, Supplementary Material online), suggests the recessive nature of this phenotype.

As mentioned above, homozygous *βknl2* mutants cannot be selected among the progeny population of heterozygous lines grown on soil. Therefore, we tested whether the abnormal seeds, possibly homozygous for *βknl2* mutations, could germinate and survive under *in vitro* conditions, where seeds and seedlings would be protected from the negative effects of environmental conditions and where the risk that homozygous seedlings would be overgrown by a population of heterozygous plants and WT plants would be minimized.

For both mutants, we found abnormal seedlings, with reduced growth rate and root development (fig. 7*E*). According to the genotyping results, abnormal seedlings represented homozygous mutants, which occur at a frequency of 2–6% of the total number of sown seeds. Unfortunately, our repeated attempts to transfer homozygous seedlings into the soil resulted in their death (fig. 7*F*). At the same time, heterozygous mutant seedlings were not distinguishable from the WT ones (fig. 7*E*). In heterozygous self- or manually pollinated mutants containing single T-DNA insertions, the siliques show <25% of abnormal seeds that does not correspond to the Mendelian monohybrid phenotypic ratio (fig. 7*C*). We hypothesized that this might be due to inaccuracy in the visual phenotyping of immature seeds. Therefore, as the next step, the dry-seed phenotype was analyzed in single siliques (fig. 7*G–J*). The heterozygous mutants in addition to normal seeds contain small, dark-colored, and shriveled ones (fig. 7*H–I*) in contrast to the WT (fig. 7*G*) with uniform seed size and color.

We observed that the abnormal dry-seed phenotype is significantly more frequent in the siliques of both heterozygous mutants compared with WT (fig. 7*J*, *P* ≤0.001) and the frequency is similar to that of the whitish seeds in fresh siliques (supplementary fig. S8, Supplementary Material online). Thus, it can be assumed that a large part of the whitish seeds with a glossy surface became dark and small or shriveled on drying.

**Fig. 8.**
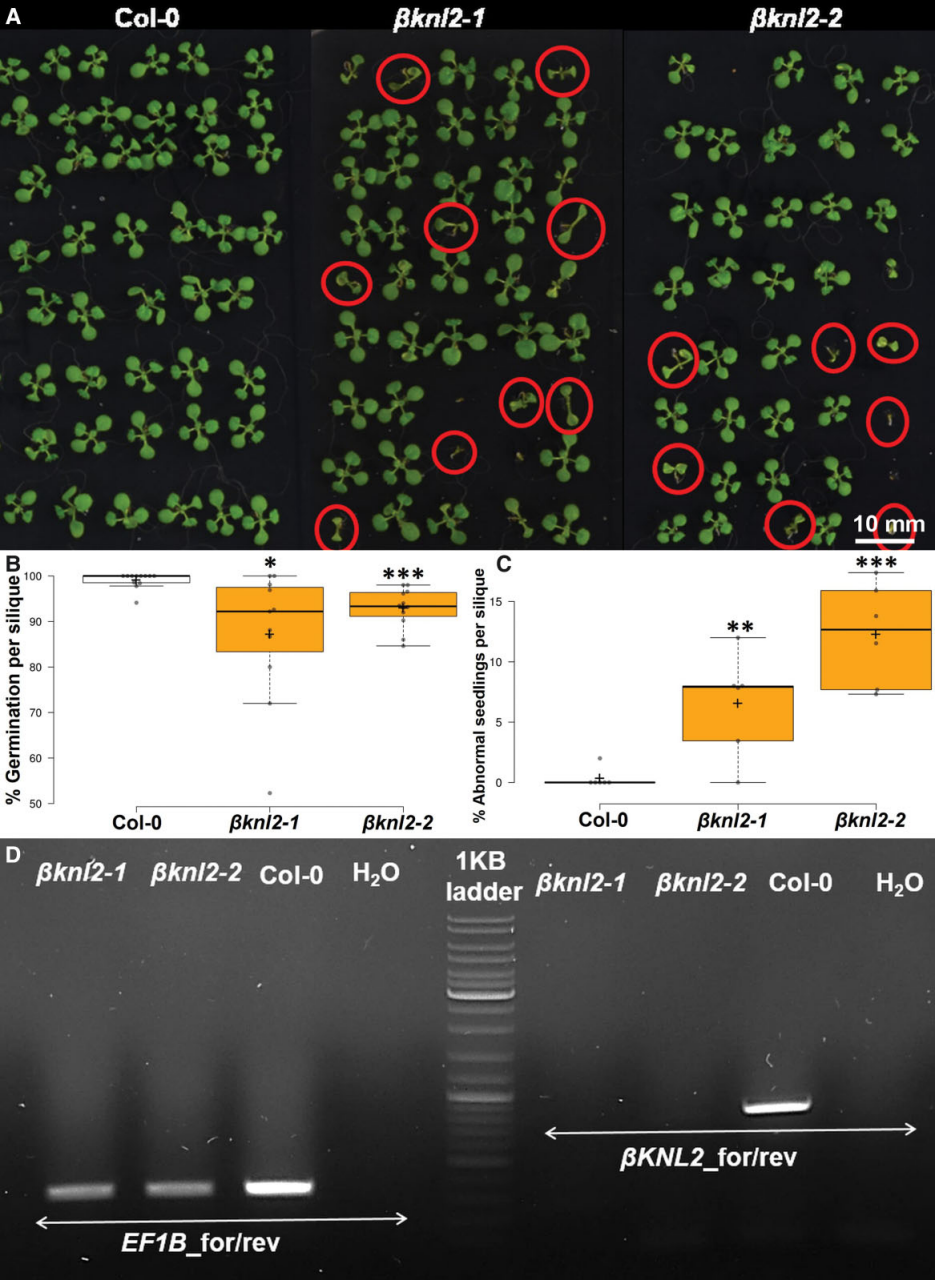
Analysis of single siliques for seeds germination and presence of abnormal seedlings. (*A*) Two-week-old *in vitro* germinated seeds collected from single siliques of WT as control and heterozygous self-pollinated *βknl2-1* and *βknl2-2* plants. *βknl2* homozygous seedlings are indicated by red circles. Bars: 1 cm. (*B*) Boxplot depicting the significant decrease of germination percentage per silique of heterozygous *βknl2-1* and *βknl2-2* compared with WT as control (*P ≤ 0.05, ***P ≤ 0.001). (*C*) Boxplot depicting the significant increase of abnormal seedlings (red color circled seedlings in (*A*) germinated from single silique seeds of heterozygous *βknl2-1* and *βknl2-2* compared with WT as control (**P ≤ 0.01), ***P ≤ 0.001). (*D*) RT-PCR amplification of *βKNL2* from *βknl2-1* and *βknl2-2* homozygous null mutants and WT as the positive control with *βKNL2* (EMB1674) gene-specific primers and *EF1B* primers as housekeeping gene.

Additionally, we analyzed the germination rate of seeds obtained from single siliques of both heterozygous *βknl2* mutants and WT (fig. 8*A,B*). Compared with WT, mutants showed a significantly decreased germination rate (fig. 8*B*, *P* ≤ 0.01) and increased number of abnormal seedlings per single silique (fig. 8*A,C*, *P* < 0.01). To test the Mendelian segregation of phenotype–genotype ratio, we also performed single silique genotyping. In the case of *βknl2-1*, the homozygous mutation represents ∼16% per silique and *βknl2-2* ∼25% (supplementary table S3, Supplementary Material online). The variation between the two mutants may be due to the different quality of the seeds harvested at two different time points and, as a result, the lower germination of the homozygous lines of one of the mutants.

To test whether abnormal seedlings (reduced seedling size and reduced root length) of both *βknl2* mutants possess the *βKNL2* transcripts, the reverse transcription-PCR (RT-PCR) analysis with gene-specific primers for *βKNL2* was performed on RNA isolated from three to five seedlings pooled together. The results showed an absence of full-length *βKNL2* transcript in both mutant lines *βknl2-1* and *βknl2-2*, suggesting that homozygous seedlings for further analysis can be selected based on their abnormal phenotype without additional genotyping (fig. 8*D*).

### *Arabidopsis* βKNL2 is Required for Proper CENH3 Loading and Correct Somatic Cell Division

**Fig. 9.**
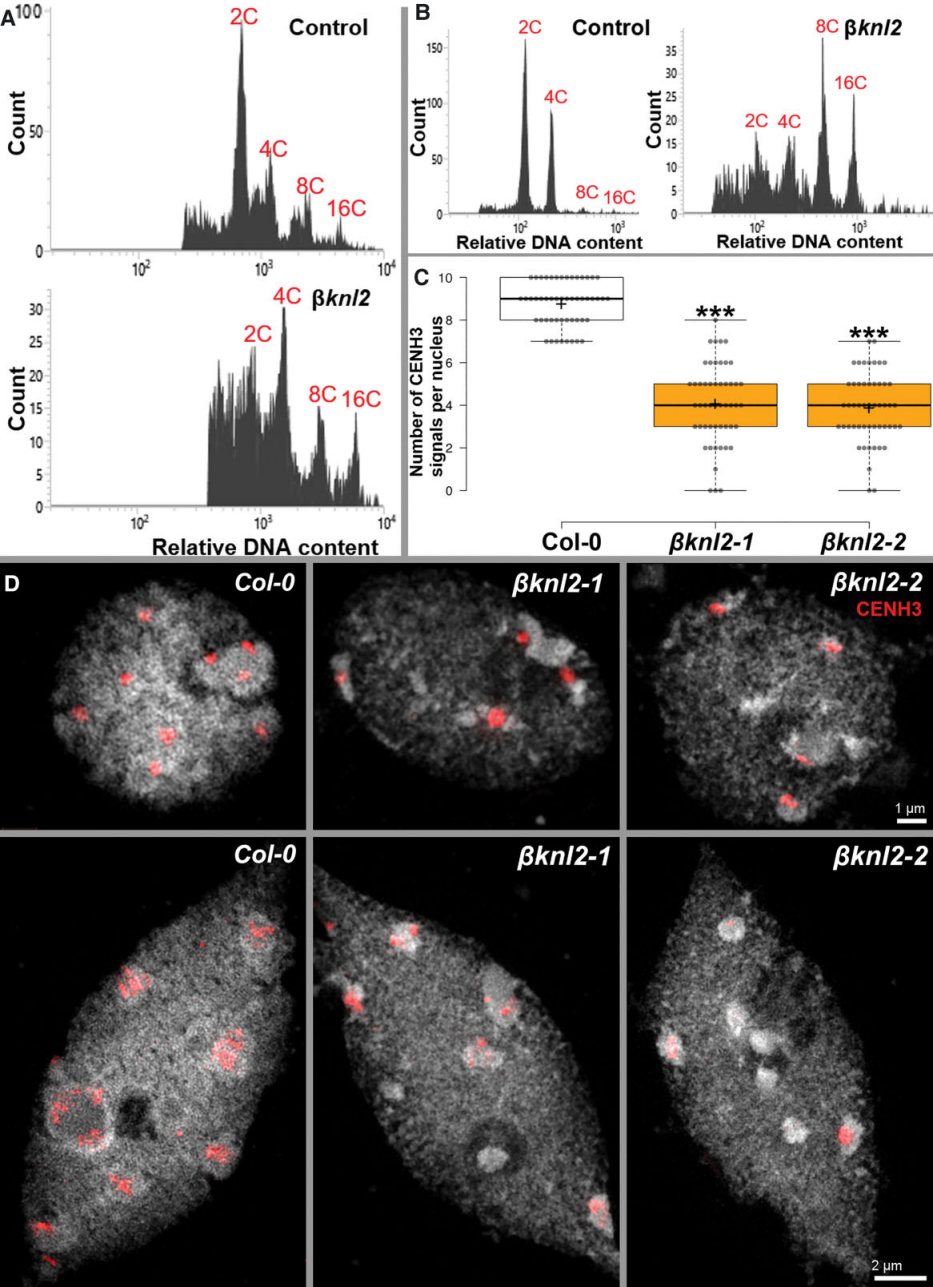
Reduced CENH3 levels in *βknl2* null mutants leading to endoreduplication. (*A*) Representative ploidy analysis histogram of normal (green) seeds of heterozygous *βknl2* mutants and WT as control (upper panel) and white abnormal seeds from *βknl2* heterozygous mutants (lower panel). (*B*) Representative ploidy analysis histogram of WT seedlings as control (left panel) and abnormal seedlings of *βknl2* null mutants (right panel). (*C*) Boxplot showing a significant decrease in the number of centromeric CENH3 signals in *βknl2-1* and *βknl2-2* compared with WT as a control (***P ≤ 0.001). (*D*) Super-resolution microscopy images showing nuclei of WT and *βknl2* null mutants immune-stained with anti-CENH3 antibodies in meristematic cells (top) and differentiated cells (bottom).

We showed that βKNL2 colocalizes at centromeres with CENH3 (fig. 5*B*) and has a localization pattern similar to that of αKNL2 (Lermontova et al. 2013). To analyze whether βKNL2, similar to αKNL2, is involved in the regulation of cell divisions and CENH3 loading, we used homozygous seedlings of both mutants for flow cytometry (FC) analysis and nuclei isolation for immunostaining. The seedlings were selected based on their abnormal phenotype. Thus, leaves of abnormal seedlings and additionally abnormal white seeds were checked by FC for ploidy levels. Comparison of the green seeds of heterozygous mutants with WT showed similar histogram profiles with a pronounced 2C embryo peak (fig. 9*A*, top), whereas the white seeds showed a clear shift toward increased endopolyploidy levels with the 4C nuclei being in most cases the dominant population (fig. 9*A*, bottom; supplementary fig. S10, Supplementary Material online). In addition, we noticed a reduced sharpness of the peaks probably due to the occurrence of aneuploid nuclei. In some cases, it was even impossible to identify nuclear peaks (supplementary fig. S10, Supplementary Material online). To analyze ploidy levels of seedlings we chopped a single leaf from six 14 days old seedlings of WT and homozygous *βknl2*. In contrast to WT leaves with distinct peaks of 2C and 4C nuclei, in mutant leaves high ploidy nuclei such as 8C and 16C were predominant (fig. 9*B*, supplementary fig. S11, Supplementary Material online).

To find whether the *βKNL2* knockout results in reduced loading of CENH3 at centromeres, similar to αKNL2 deregulation, we performed an immunostaining experiment with anti-CENH3 antibodies on nuclei isolated from 14-day-old seedlings of WT and *βknl2* mutants. In *A. thaliana* roots and leaves, there are predominantly two forms of nuclei (flattened sphere and spindle) occurring (Pecinka et al. 2004). Root meristems contain mainly spherical nuclei (fig. 5*A*), while in the elongated differentiated regions spindle-shaped nuclei appear. These differently shaped nuclei were included in the immunostaining experiment. We found that compared with WT, the mutant nuclei contain less CENH3 signals independent of nucleus shape. The CENH3 signals were counted in 50 round-shaped WT, *βknl2-1* and *βknl2-2* nuclei, respectively. In contrast to WT with eight to ten signals, both mutants showed on average only four signals (fig. 9*C* and supplementary fig. S12, Supplementary Material online). We performed the Student’s *t*-test and found that the mutants have significantly lower number of CENH3 signals compared with WT (fig. 9*C*, *n* ≤ 6, *P* < 0.001). Furthermore, Mean Fluorescence Intensities were calculated to quantify the centromeric CENH3 levels. Compared with WT, the signal intensities were reduced to 68.98% (*P* < 0.001) in *βknl2-1*, and to 79.47% (*P* < 0.01) in *βknl2-2*, respectively (supplementary fig. S13, Supplementary Material online). In spindle-shaped nuclei, the CENH3 immunosignals on chromocenters were mostly dispersed in the WT and both *βknl2* mutants, whereas in the mutants some chromocenters were completely free of signals. The observed dispersion of CENH3 signals in spindle-shaped nuclei with increased ploidy levels is in agreement with our previous observations (Lermontova et al. 2006). To analyze the chromatin ultrastructure in more detail, representative nuclei from the same slides were captured by spatial structured illumination super-resolution microscopy (3D-SIM; fig. 9*D*). We observed that in nuclei with reduced CENH3 levels the chromatin remains normal as in WT suggesting that intact non-degraded nuclei were selected for the analysis. In summary, our data suggest that the reduced CENH3 amount in the homozygous *βknl2*-*1*&*2* mutants lead to the inhibition of mitosis and switching of cells to endocycles.

## Discussion

### Duplication of *KNL2*

Most metazoan genomes have only one *KNL2* gene with the SANTA domain, except for the allotetraploid *Xenopus laevis*, where two *KNL2* genes were identified; both with identical CENPC-k motifs, nearly identical SANTA and Myb (SANT) domains, and 74% sequence similarity (Moree et al. 2011; French et al. 2017). In contrast, two genes containing the SANTA domain were identified in water ferns, eudicots, and grasses, whereas only one *KNL2* copy was found in bryophytes and gymnosperms (fig. 2). Though Brassicaceae species experienced multiple whole genome duplication (WGD) events such as the *At-α* and *At-β* WGDs (Edger et al. 2018), most species exhibit two *KNL2* gene copies, *αKNL2* and *βKNL2*, except for a few neopolyploid species which have experienced an extra recent WGD event(s).

We found strong conservation of the SANTA domain of KNL2, notably in the Vx**L**xD**W** motif at the N-terminus and the GFxxxxxxx**F**xxG**F**Pxx**W** motif at the C-terminus (fig. 3*A*), where the bolded residues impaired CENP-C binding when mutated in *Xenopus* M18BP (French and Straight 2019), suggesting that plant KNL2s may also bind CENP-C through the SANTA domain. In addition, analysis of αKNL2 and βKNL2 protein sequences identified numerous paralog-specific motifs, suggesting that the paralogs might be subfunctionalized. A study in *Drosophila* has shown that Cid (CENH3) paralogs evolved new motifs following Cid duplication (Kursel and Malik 2017). Loss of ancestral motifs in *Drosophila* Cids was proposed as direct evidence of subfunctionalization (Kursel and Malik 2017; Kursel et al. 2020).

We identified positive selection sites in and near the SANTA domain of KNL2 in the analyzed Brassicaceae species, similar to what has been previously reported for CENH3 (Talbert et al. 2002) and CENP-C (Talbert et al. 2004). Thus, KNL2 might be responding to centromere drive through interaction with rapidly evolving CENH3 and CENH3 chaperone NASP^SIM3^, which recently was identified in *Arabidopsis* (Le Goff et al. 2020), or with CENP-C. However, the mechanisms of adaptively evolving regions remain to be elucidated.

### Partial or Complete Loss of the CENPC-k Motif in KNL2 in Different Clades of Plants

The CENPC-k motif is found in KNL2 of diverse eukaryotes including non-mammalian vertebrates, many invertebrates, chytrid fungi, cryptomonads, and plants (Kral 2016; Sandmann et al. 2017). In eudicots the conserved CENPC-k motif is present in the αKNL2 clade, but is absent from βKNL2. Similarly, in most grass species the CENPC-k motif is conserved in γKNL2 clade, while δKNL2 clade does not have the motif. However, we found a RRLRSG**K**V/I motif in the δKNL2 clade possibly related to the beginning of the CENPC-k motif (KRSRSG**R**V/LLVSPLEFW; supplementary file S6, Supplementary Material online). We showed previously that the substitution of the bolded seventh Arg in the CENPC-k motif (above) by Ala abolishes centromere targeting of αKNL2 (Sandmann et al. 2017). In the truncated putative CENPC-k motif, Lys is present instead of Arg. Since these two amino acids have similar features, Lys might be required for the targeting of δKNL2 to centromeres. However, the truncated putative CENPC-k motif does not include the Trp which similar to Arg, is also needed for the targeting of αKNL2 to centromeres (Sandmann et al. 2017). Moreover, it remains to be elucidated whether KNL2 variants with the truncated CENPC-k motif can target CENH3 nucleosomes directly, without an additional interacting partner. Among all grass species with sequenced genomes, maize represents an exception, since it has only one *KNL2* gene which belongs to the δ*KNL2* clade with the truncated CENPC-k and has no γKNL2 protein variant with the complete CENPC-k motif. Interestingly, in sorghum, closely related to maize, the γKNL2 protein can be identified (supplementary file S5, Supplementary Material online). On the other hand, for other species, it may be postulated that centromeric targeting of βKNL2 and δKNL2 depends on αKNL2 and γKNL2, respectively, for maize this assumption cannot be applied. This suggests that maize may have evolved a different mechanism for CENH3 deposition compared with other grasses. Notably, δKNL2 retains the hydrophobic residues in the SANTA domain that are important for CENP-C binding in *Xenopus*. Perhaps the mechanism of localization and function of KNL2 in maize relies on CENP-C binding similar to *Xenopus*. Interestingly, two CENP-C proteins were identified in maize (Talbert et al. 2004), in contrast to other species.

### The Function of βKNL2 in Plants

Although KNL2 protein homologues have been identified in different organisms as components of the CENH3 loading machinery, they differ considerably in the composition of their functional domains, interacting partners, and localization timing in the mitotic cell cycle. The mammalian M18BP1, composed of the conserved N-terminal (Mis18α-binding) region, SANTA domain, CENP-C-binding domain, SANT (Myb-like) domain and the C-terminus, is lacking the CENPC-k motif. The N-terminal (Mis18α-binding) region and the CENP-C-binding domain are required for centromere targeting (Stellfox et al. 2016). Deletion of the SANTA domain in mammalian and chicken M18BP1/KNL2 does not abolish its centromeric localization (Stellfox et al. 2016; Hori et al. 2017). In contrast, mutation of the SANTA domain in *Xenopus* reduced centromeric localization of M18BP1/KNL2 by 90% (French et al. 2017). Later, the same authors demonstrated that the SANTA domain is required for the interaction of M18BP1/KNL2 with CENP-C during metaphase (French and Straight 2019).

We showed previously that in *Arabidopsis* the centromeric localization of αKNL2 depends on the CENPC-k motif (Sandmann et al. 2017), while it was not abolished in the complete absence of the N-terminal part of KNL2 containing the SANTA domain (Lermontova et al. 2013). The C-terminal half of *Arabidopsis* KNL2 was not only sufficient for its targeting to centromeres, but also the interaction with DNA (Sandmann et al. 2017). In the present study, we demonstrated that βKNL2 colocalizes with CENH3 at centromeres, despite lacking a CENPC-k motif. In general, both variants of *Arabidopsis* KNL2 showed a similar localization pattern during interphase. However, in contrast to αKNL2, βKNL2 can be detected on chromosomes during metaphase and early anaphase (fig. 5*C,D*). The centromeric location of βKNL2 suggests that *βKNL2* may partially compensate for the loss of *αKNL2* in the corresponding *Arabidopsis* mutant which showed only reduced, but not completely abolished CENH3 loading which would be lethal (Lermontova et al. 2013). Homozygous T-DNA insertions for βKNL2 resulted in plant death at the seedling stage and probably because of reduced root development. However, it should be considered that in the analyzed *αknl2* mutant, the T-DNA was inserted after the SANTA domain coding region, whereas in the case of *βknl2* mutants, one T-DNA was inserted before and the other directly in the SANTA domain coding region. Therefore, it cannot be excluded that truncated αKNL2 with the full SANTA domain may retain some function in the mutant. As reciprocal crosses of *βknl2* mutants with the WT resulted in normal seed development in both directions, we hypothesized that the *βKNL2* null mutations do not affect gametes or fertilization processes, but rather postzygotic cell divisions. In support of this hypothesis, FC ploidy analysis of young seedlings revealed that in contrast to the WT with distinct 2C and 4C peaks, homozygous mutants showed a shift toward endopolyploidization (fig. 9*B*), potentially a consequence of disrupted cell divisions. Impaired mitotic divisions in mutant seedlings can be explained by the reduced levels of CENH3 on the centromeres of both mutants (supplementary figs. 9D and S13, Supplementary Material online). Thus, our data strongly suggest the involvement of βKNL2 protein in CENH3 loading. The ability of cells in homozygous seedlings to undergo some mitotic divisions can be explained by residual amounts of CENH3 from parental plants, and when CENH3 levels are highly diluted, cells switch from mitotic cycle to endocycles. We observed that the development of homozygous seedlings can be inhibited at different stages (fig. 7*E*).

Taken together, our results suggest that the *KNL2* gene in eudicots underwent an early duplication with the core function of CENH3 deposition to define the centromere region. Due to the lack of the CENPC-k motif in βKNL2, we propose that in *Arabidopsis* βKNL2 might localize to centromeres by binding to CENP-C through the SANTA domain as it was shown for *Xenopus* (French and Straight 2019), or through the conserved N-terminal motif located upstream of the SANTA domain similar to what was previously described in human (Stellfox et al. 2016), or through both of these regions.

Although in the SANTA domain of βKNL2, three putative Aurora kinase phosphorylation sites can be identified, there is only one in αKNL2 (fig. 4*A*). This fact might suggest that both KNL2 variants are involved in the formation of different protein complexes. We also could not rule out the possibility that βKNL2 assembles with a Mis18 complex to ensure centromeric localization and subsequent CENH3 deposition. So far, Mis18α and β proteins have not been identified and characterized in *Arabidopsis*. However, in silico analysis (https://bioinformatics.psb.ugent.be/plaza/) revealed a family of seven genes (*At2G40110*, *AT3G08990*, *AT3G11230*, *AT3G55890*, *AT4G27740*, *AT4G27745*, and *AT5G53940*) encoding proteins with the Yippee-Mis18 domain-specific to Mis18 proteins (Stellfox et al. 2016). Recently, it was demonstrated that the direct binding of *Schizosaccharomyces pombe* Mis18 to nucleosomal DNA is important for the recruitment of *sp*Mis18 and Cnp1 (CENH3) to the centromere in fission yeast (Zhang et al. 2020). In contrast to αKNL2, βKNL2 not only lacks the CENPC-k domain but also the part necessary for interaction with DNA. Thus, an association with Mis18 proteins, with the ability to bind to DNA, is plausible. We also cannot exclude that centromere targeting of βKNL2 depends on αKNL2.

We showed previously that manipulation of αKNL2 can be used for the production of haploids and subsequently of double haploids in *Arabidopsis* (Lermontova 2017; Ahmadli et al. 2022a). Double haploid production helps to accelerate plant breeding as it allows to generate true-breeding lines in one generation instead of the seven to nine generations required for conventional selection (Britt and Kuppu 2016; Kalinowska et al. 2019). Here we demonstrate that *KNL2* genes exist in two variants in eudicots (*α, βKNL2*) and monocots (*γ, δKNL2*). The conserved gene structure and expression patterns of *α/γKNL2* in both eudicots and monocots suggest that *α/γKNL2* mutations could be used to develop *in vivo* haploid induction systems in different crop plants. Similarly, the newly identified βKNL2 may become the subject of manipulations to obtain haploids both in *Arabidopsis* and in crops. As homozygous *βknl2* mutants are dying at the seedling stage, we can assume that the heterozygous mutant plants can also induce haploids similar to what was described for the heterozygous *cenh3* mutants of maize and wheat (Lv et al. 2020; Wang et al. 2021).

## Materials and Methods

### Data Sources and Sequences Retrieval

The KNL2 protein sequences of *A. thaliana* were identified by screening the *Arabidopsis* Information Resource (TAIR10) using the specific gene number. To obtain and annotate *KNL2* members in plants, we downloaded 88 representative species reference genomes or transcriptomes including red and green algae, bryophytes, lycophytes, ferns, gymnosperms, and angiosperms from the Phytozome database (Goodstein et al. 2012; https://phytozome.jgi.doe.gov/), NCBI genome database, Ensembl Plants database, PLAZA database, and other single genome website (supplementary table S1, Supplementary Material online). We used the homology search tool BLASTP to scan the reference proteome with a cutoff *e*-value of 0.01 using whole sequences and conserved domains from *Arabidopsis* αKNL2 as the query. TBLASTN was used as an additional method for failed identification case. Two KNL2 protein sequences from *Colocasia esculenta* and *Phoenix dactylifera* were retrieved from GenBank database. Then, we combined the BLAST results and deleted spliced variants in multiple sequence alignments. The protein data are summarized in supplementary table S1 and file S1, Supplementary Material online.

### Alignments and Phylogenetic Analysis

To explore the phylogenetic relationships of the *KNL2* genes in plant lineages, KNL2 protein sequences were aligned using MAFFT software (Yamada et al. 2016) and potentially inaccurate regions of βKNL2 were excluded. Evolutionary relationships among *KNL2* gene family members were determined by using IQ-TREE software (Nguyen et al. 2015) and ML methods based on 1000 bootstrap alignments and single-branch tests. The phylogenetic trees were visualized and modified using the Fig-Tree v1.4.4 software (http://tree.bio.ed.ac.uk/software/figtree/). Sequence logos were generated using WebLogo3 (http://weblogo.berkeley.edu/;  Crooks et al. 2004).

### Sequence Motif Analysis

The unaligned amino acid sequences of KNL2 were collected to search for additional conserved motifs using MEME suite v5.1.0 (Bailey et al. 2009). Due to misleading annotation of the *βKNL2* gene (Lermontova et al. 2013), we manually removed the KIP1 domain regions in some species. The data set was submitted to the MEME server (http://meme-suite.org/) and the conserved domains and motifs were marked. We used the motif search algorithm MAST (Bailey and Gribskov 1998) to identify motifs.

### Plasmid Construction, Plant Transformation, and Cultivation

The entire open reading frame of *βKNL2* (*At1g58210*) was amplified by RT-PCR with RNA isolated from flower buds of *Arabidopsis* WT and cloned into the pDONR221 vector (Invitrogen) via the Gateway BP reaction. From pDONR221 clones, the open reading frame was recombined via Gateway LR reaction (Invitrogen) into the two attR recombination sites of the Gateway-compatible vectors pGWB641and pGWB642 (http://shimane-u.org/nakagawa/gbv.htm), respectively, to study the localization of βKNL2 protein *in vivo*.

Plants of *Arabidopsis* accession Columbia-0 were transformed according to the flower dip method (Clough and Bent 1998). T1 transformants were selected on Murashige and Skoog (MS) medium (Murashige and Skoog 1962) containing 20 mg/l of phosphinotricine. Growth conditions in a cultivation room were 21 °C 8 h light/18 °C 16 h dark or 21 °C 16 h light/18 °C 8 h dark.

### Analysis of T-DNA Insertion Mutants

Seeds of T-DNA insertion lines were obtained from the European *Arabidopsis* stock center (http://arabidopsis.info/). To confirm the presence of the T-DNA, and identify heterozygous versus homozygous T-DNA insertions, we performed PCR with pairs of gene-specific primers flanking the putative positions of T-DNA (supplementary table S4, Supplementary Material online) and with a pair of gene-specific and T-DNA end-specific primers (LBb3.1, supplementary table S4, Supplementary Material online). DNA isolation was performed as described in Edwards et al. (1991).

For the germination and segregation experiments, seeds from individual siliques were germinated *in vitro* on an MS medium as described above.

### Flow Cytometry

For the analysis of (endopoly)ploidy of immature seeds, white and green seeds were selected from the same silique of the heterozygous mutant and compared with the green seeds of the WT. For the analysis of (endopoly)ploidy levels in seedlings, one leaf from 2-week-old heterozygous mutant and WT seedlings was used. Seeds and leaf tissue were chopped with a razor blade in 300 μl of nuclei extraction buffer (CyStain UV Ploidy; Sysmex-Partec). The resulting nuclei suspension was filtered through a 50 μm disposable CellTrics filter (Sysmex-Partec), incubated for 10 min on ice and measured on BD Influx cell sorter (BD Biosciences).

### Immunostaining and Microscopy Analysis of Fluorescent Signals

For analysis of CENH3 loading in homozygous mutants and WT, 2-week-old seedlings were used. Slides were prepared using a cytospin and used for immunostaining as it was described by Ahmadli et al. (2022b). To determine the colocalization of βKNL2-EYFP protein with CENH3, immunostaining of nuclei/chromosomes with anti-CENH3 and anti-GFP antibodies and microscopic analysis of fluorescent signals were performed as previously described (Lermontova et al. 2013).

For time-lapse microscopy, seedlings of transformants were grown in cover slip chambers (Nalge Nunc International) for 7–10 days and analyzed with an LSM 510 META confocal laser scanning microscope (Carl Zeiss GmbH).

To investigate the interphase nucleus and centromeric chromatin ultrastructures at an optical lateral resolution of ∼100 nm (super-resolution achieved with a 405-nm laser excitation), we applied spatial structural illumination microscopy (3D-SIM) using a 63/1.40 objective of an Elyra PS.1 super-resolution microscope system (Carl Zeiss GmbH; Weisshart et al. 2016; Kubalova et al. 2021) DAPI (whole chromatin) and rhodamine (CENH3 signals) were excited by 405 and 561 nm lasers, respectively.

### Expression Profile Analyses

The *Arabidopsis* genome assembly and gene annotation were downloaded from Araport11 (https://bar.utoronto.ca/thalemine/dataCategories.do) with integrative re-annotation (Cheng et al. 2017). The *KNL2* gene models were manually re-examined. The *Arabidopsis* RNA-seq data were downloaded from previous studies (Klepikova et al. 2016). RNA-seq data were selected from ten tissue types in *Arabidopsis*, including germinating seeds, stigmatic tissue, ovules from sixth and seventh flowers, young seeds, internode, the axis of the inflorescence, flower, anthers of the young flower, opened anthers, and root (NCBI SRA: SRR3581356, SRR3581684, SRR3581691, SRR3581693, SRR3581704, SRR3581705, SRR3581719, SRR3581727, SRR3581728, SRR3581732). Transcriptome analysis utilized a standard TopHat-Cufflinks pipeline with minor modification (Trapnell et al. 2012). Transcription levels were normalized to *MON1* and expressed in reads per kilobase of exon model per million mapped reads (RPKM). Expression levels of *CENH3*, *CENP-C*, and *KNL2* normalized to *MON1* in different tissues from microarray experiments were obtained from the *Arabidopsis* eFP Browser website (http://bar.utoronto.ca/efp/cgi-bin/efpWeb.cgi). The corresponding gene IDs are: *CENP-C* (*At1g15660*), *αKNL2* (*At5g02520*), *βKNL2* (*At1g58210*), and *CENH3* (*At1g01370*).

### Positive Selection Analyses

PAML 4.8 software (Yang 2007) was used to test for positive selection on KNL2 homologs from Brassicaceae species. The *KNL2* gene alignments and gene trees were used as input into the CodeML of PAML. Alignments were manually refined as described in phylogenetic analysis. To determine whether αKNL2 and βKNL2 homologs evolve under positive selection, random-site models were selected. Random-site models allow ω to vary among sites but not across lineages. We compared two models that do not allow ω to exceed 1 (M1 and M7), and that allow ω > 1 (M2 and M8). Positively selected sites were classified as those sites with a Bayes empirical Bayes posterior probability >95%.

### Statistical Data Analysis

All statistical analyses were performed in Microsoft Excel using FTEST and two-tailed TTEST functions (supplementary file S8, Supplementary Material online). Box plots were generated using the online tool BoxPlotR (http://shiny.chemgrid.org/boxplotr/, Team RC, 2013).

## Supplementary Material

Supplementary data are available at *Molecular Biology and Evolution* online.

## Supplementary Material

msac123_Supplementary_Data

## Data Availability

All data used in this manuscript are available as supplementary files to this manuscript.
