# Supplementary material for: Recurrent Plant-Specific Duplications of KNL2 and its Conserved Function as a Kinetochore Assembly Factor
Source: Mol Biol Evol. 2022 Jun 7;39(6):msac123. doi: 10.1093/molbev/msac123 (PMC9210943; doi:10.1093/molbev/msac123)
Supplement: msac123_Supplementary_Data [file msac123_supplementary_data.zip › Supplementary Figures S1-S13.pdf]

Supplementary Figure S1

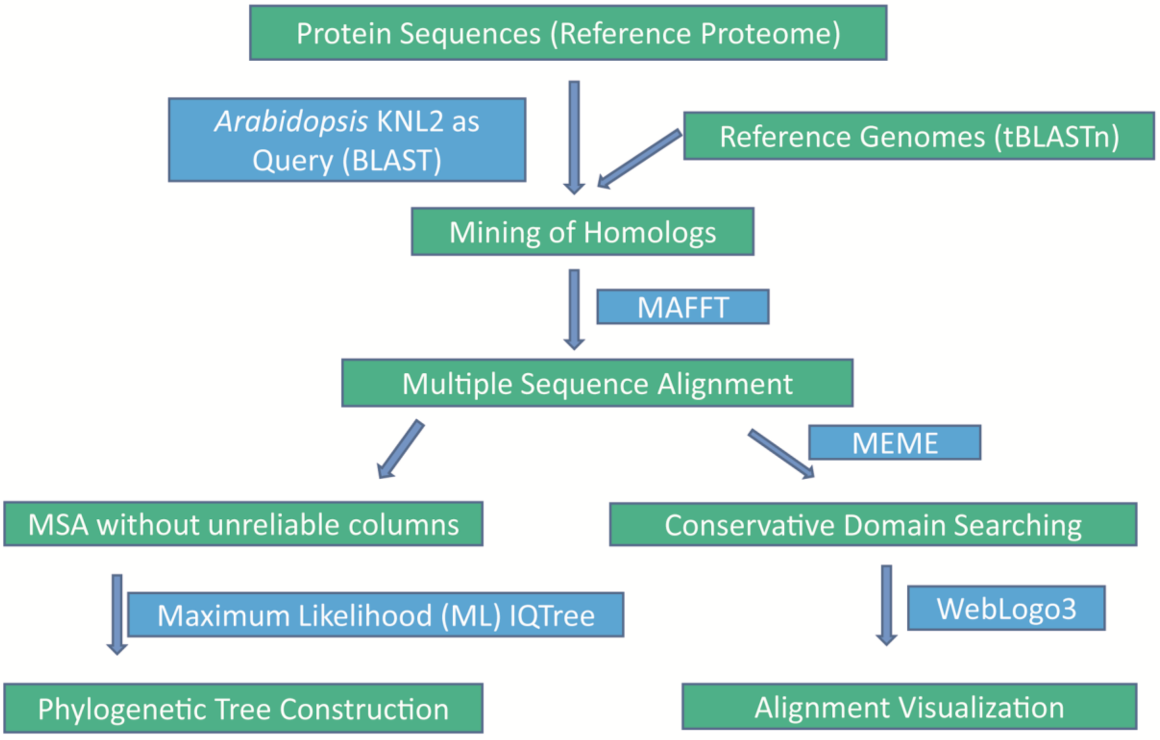

Pipeline for the data mining, phylogenetic analysis and motif identification.

Supplementary Figure S2

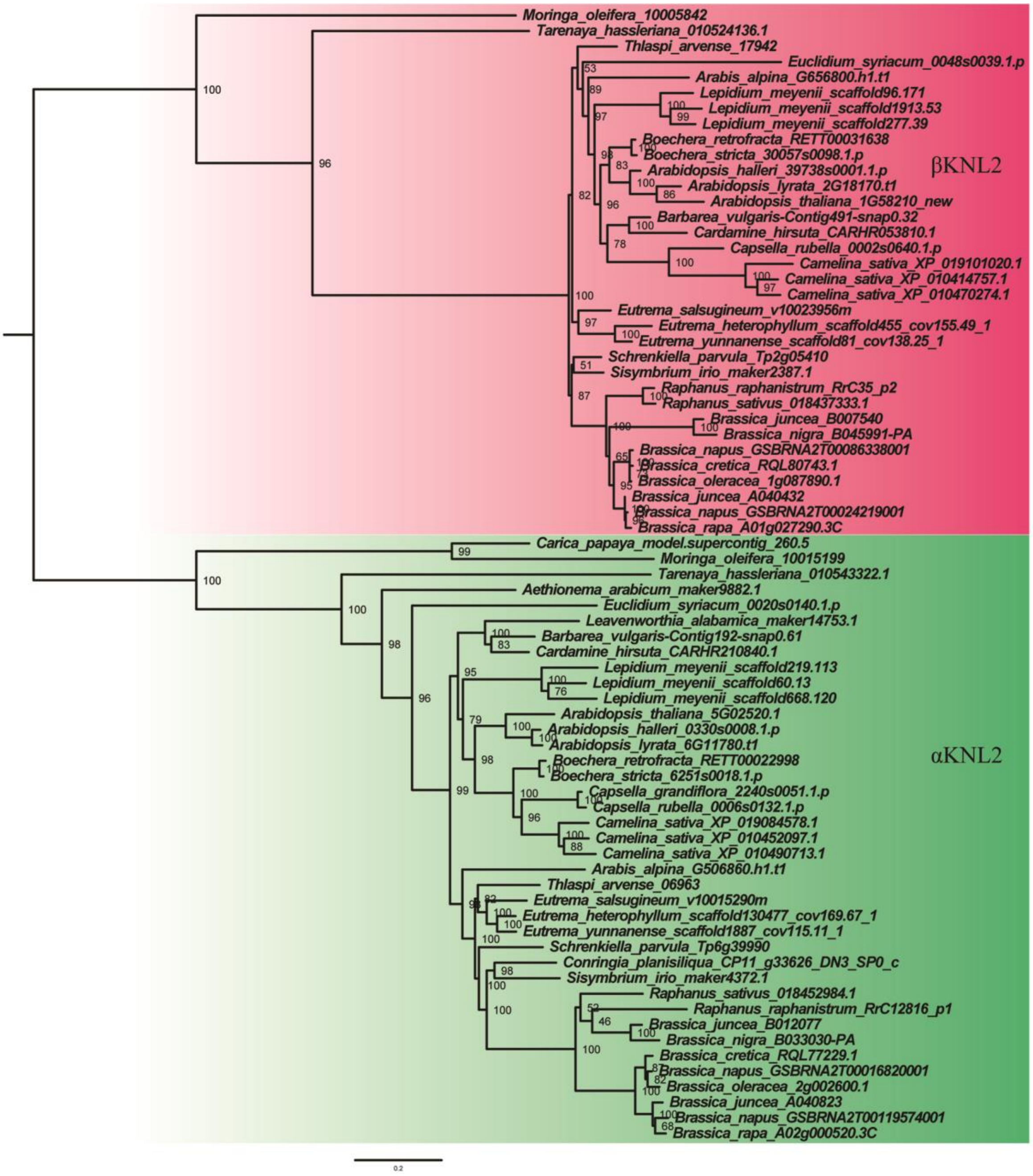

Evolutionary relationship among all KNL2 homologs in Brassicales species.

Maximum likelihood phylogenetic analysis was performed using IQ-tree with a protein alignment of KNL2 homologs in Brassicales plants. The KNL2 in Brassicales can be classified into two major groups (αKNL2 and βKNL2, respectively) indicating ancient gene duplications. Bootstrap values obtained after 1 000 ultrafast bootstrap replicates (bb) are shown in the tree. Scale bar indicates the number of substitutions per site.

Supplementary Figure S3

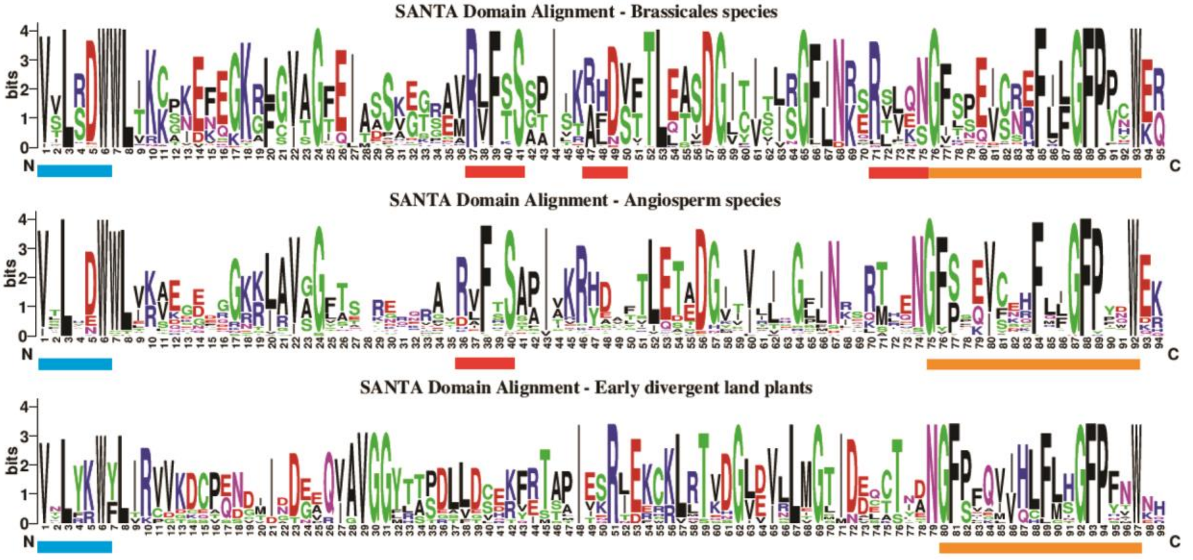

Alignment of SANTA domain in KNL2 homologs.

Variation map of the SANTA domain in the KNL2 homologs. The WebLogo program (http://weblogo.berkeley.edu/logo.cgi) was used to present SANTA domain alignments. Putative Aurora kinase phosphorylation consensus was underlined in red bars. The conserved N and C terminal hydrophobic residues (VxLxDW and GFxxxxxxxFxxGFPxxW) were indicated by blue and green bars.

Supplementary Figure S4

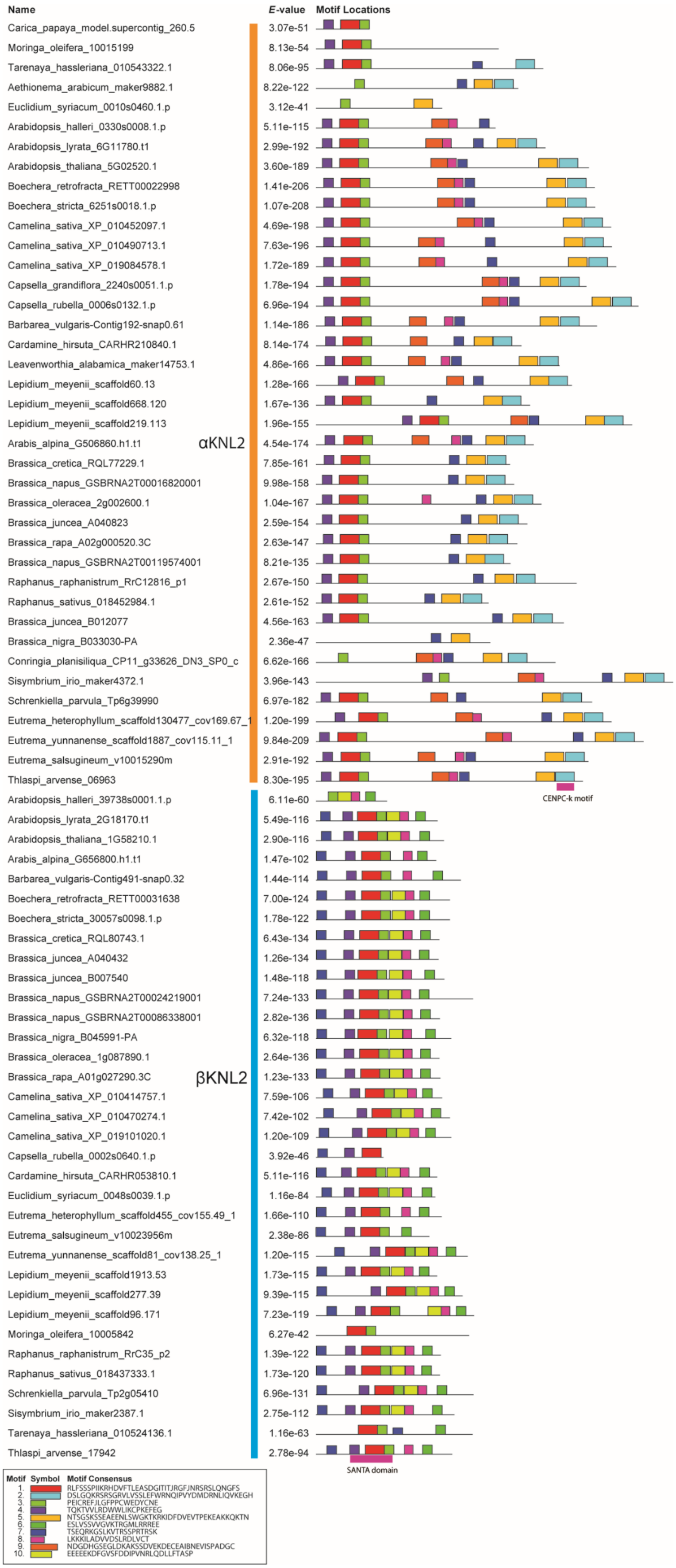

Conserved motif identification of the KNL2 homologs across the Brassicales species.

Conserved motif evolution of alpha and beta KNL2 homologs across the Brassicales. The unaligned amino acid sequences of KNL2 from Brassicales species were used to search for additional conserved motifs of KNL2 using MEME suite v5.1.0. The dataset was submitted to the MEME server (http://meme-suite.org/) and the conserved domains and motifs were marked. The scale relates to amino acid residues. The SANTA domain comprised by several domain was underlined in pink bars.

Supplementary Figure S5

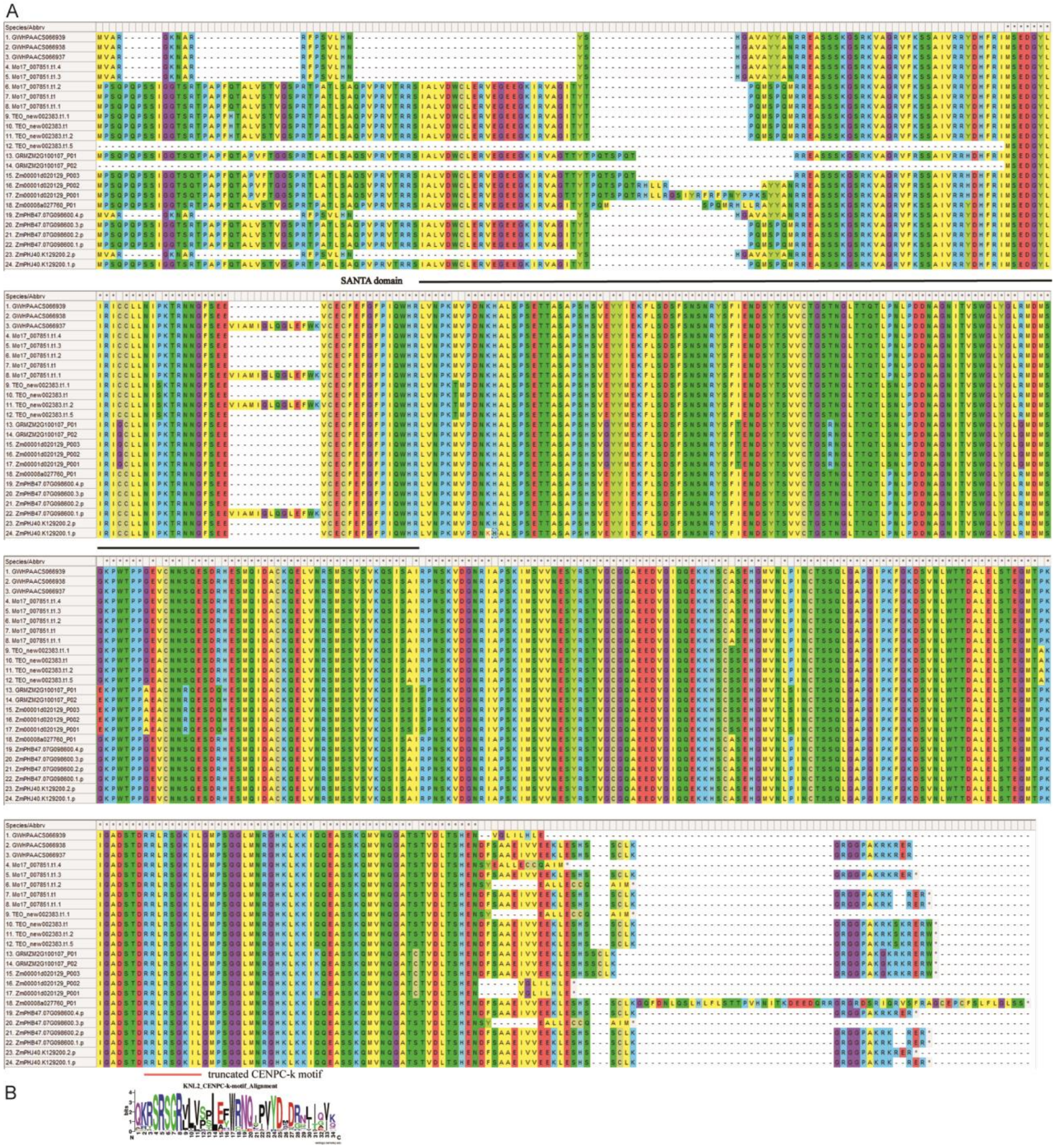

Maize KNL2 alignment from different genome annotations.

Supplementary Figure S6

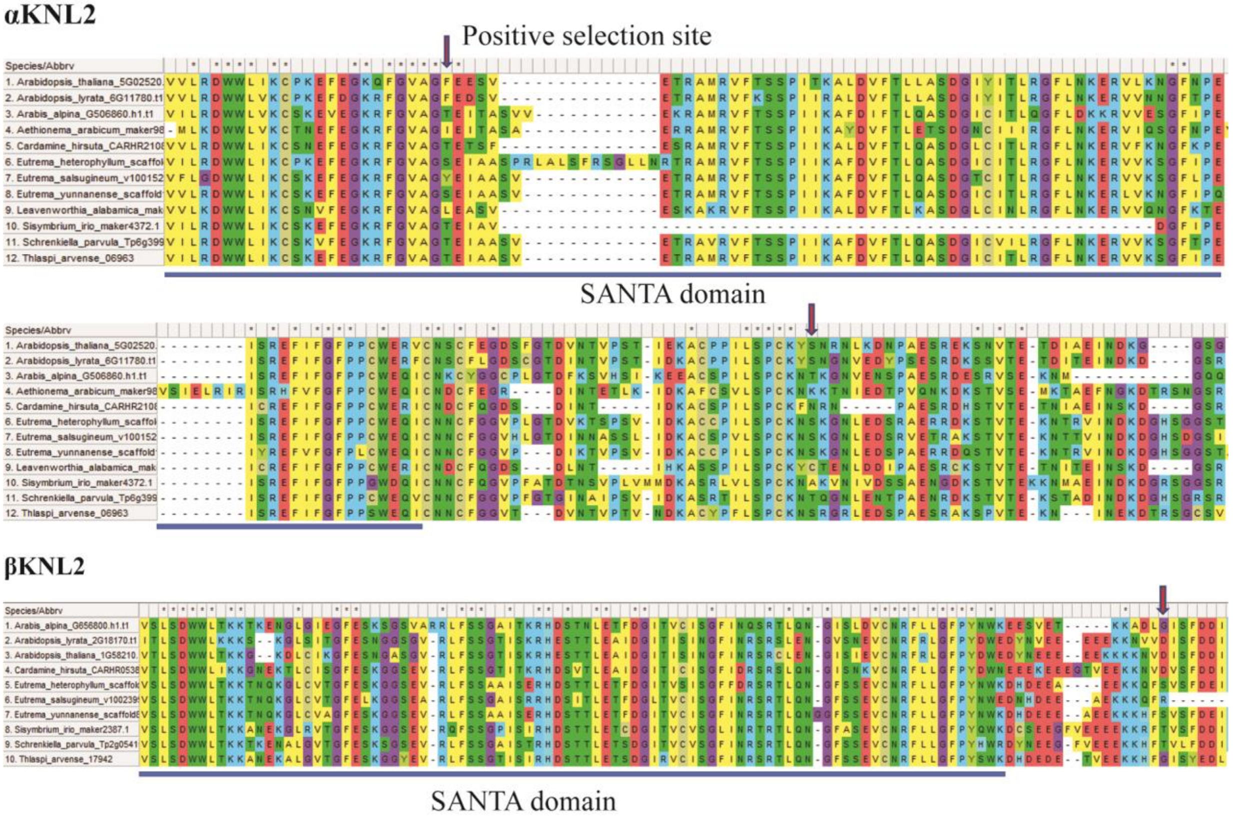

Positive selection site of KNL2 orthologs from Brassicaceae species.

Supplementary Figure S7

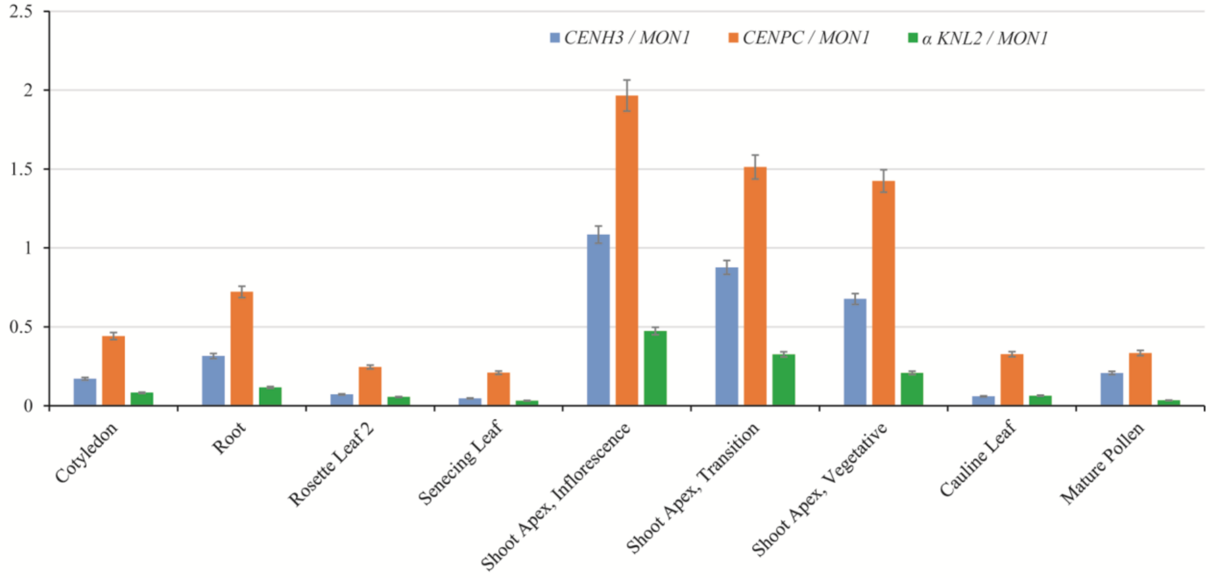

Expression profiles of *CENH3*, *CENP-C* and *KNL2*.

Column charts showing different expression levels of the CENH3, CENP-C and KNL2 genes in tissues enriched for dividing cells. The relative FPKM values Expression levels of CENH3, CENP-C and KNL2 were normalized to the reference gene MON1 (At2g28390). The corresponding gene id numbers are: CENH3 (At1g01370), CENP-C (At1g15660), and βKNL2 (At1g58210).

Supplementary Figure S8

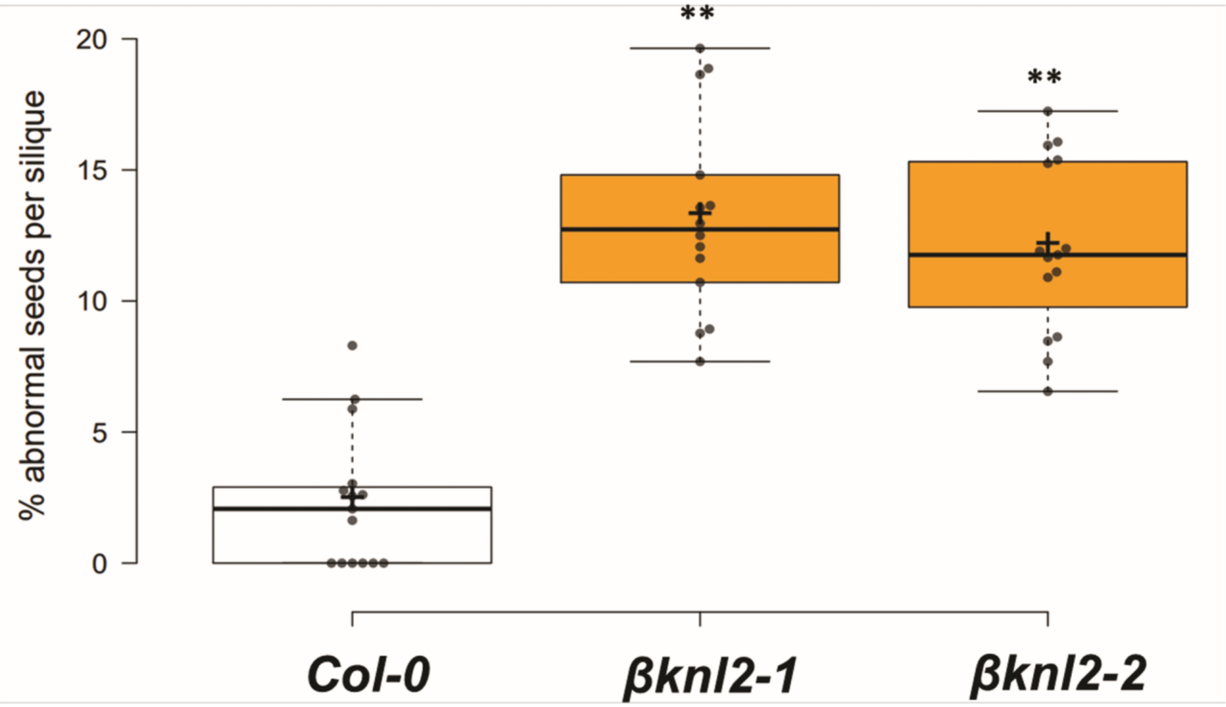

Analysis of abnormal seed phenotype from heterozygous β*knl2* mutants.

Box plots show an increase in the number of abnormal (whitish shiny) seeds per single silique in *βknl2-1* (P≤0.01) and *βknl2-2* (P≤0.01).

Supplementary Figure S9

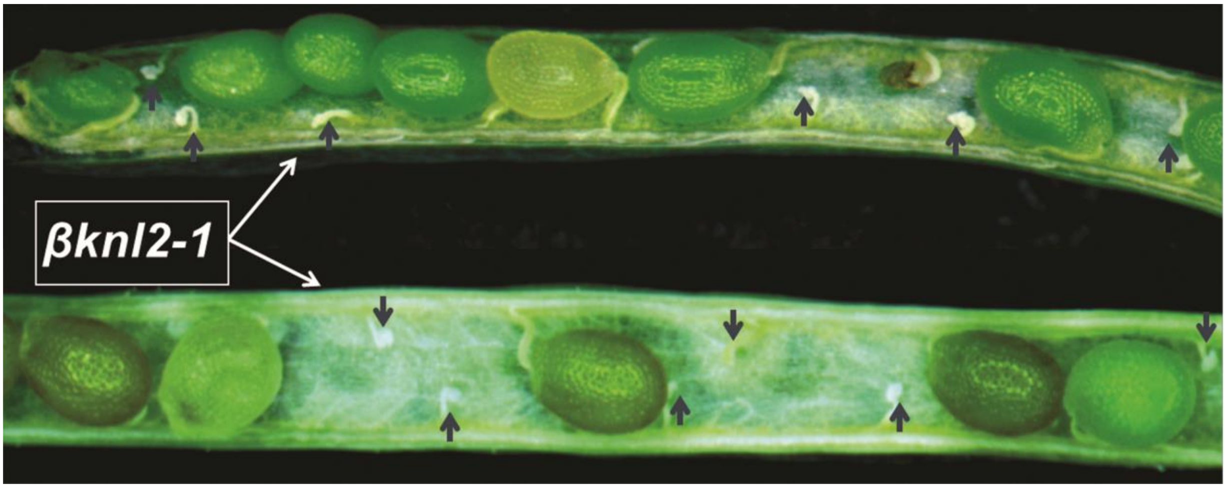

Representative siliques showing early ovule abortion phenotype in heterozygous β*knl2-1* plants along with abnormal whitish seeds and normal green seeds. The early ovule abortions caused by other T-DNA insertions are shown by grey arrows.

Supplementary Figure S10

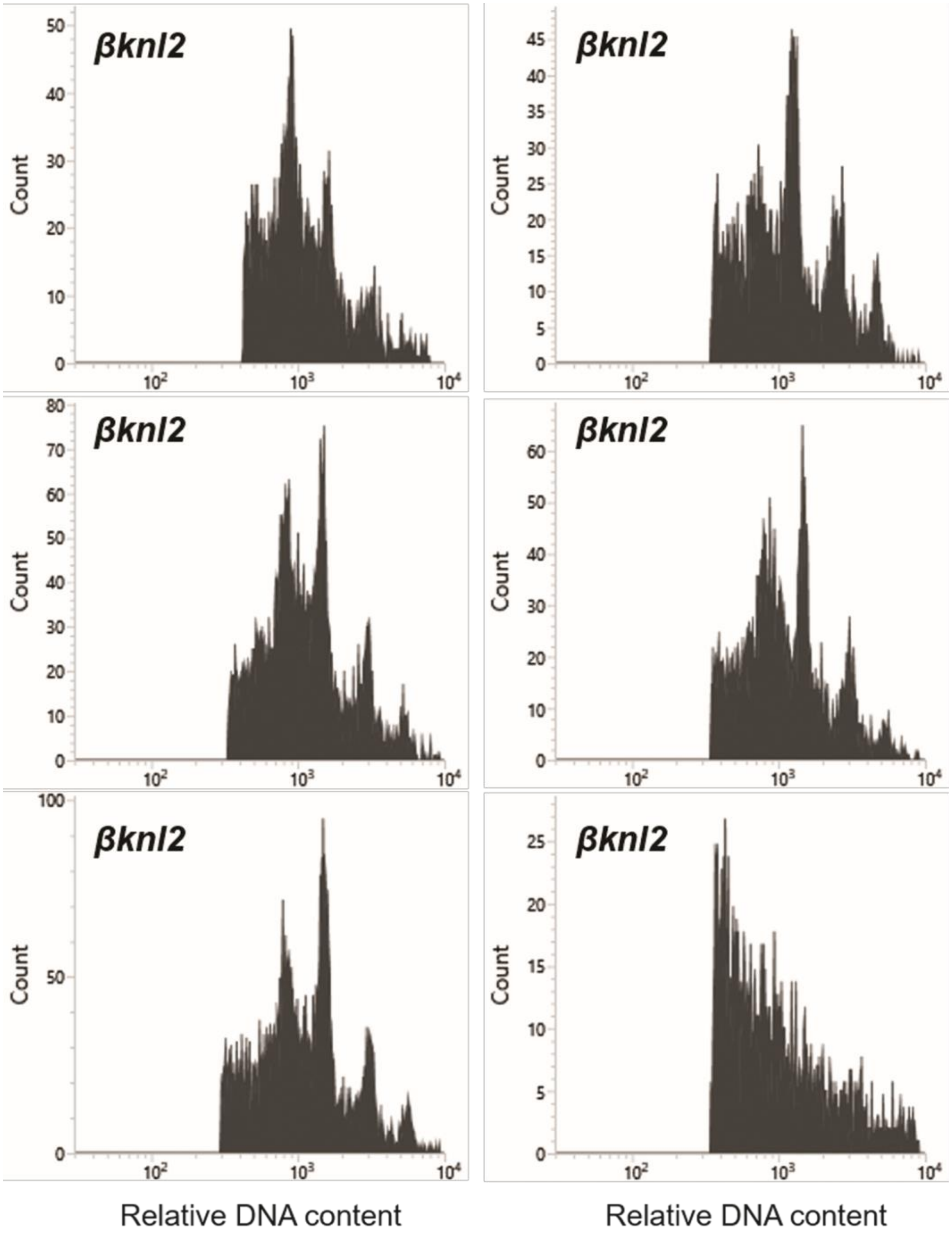

Supplementary Figure 10. Flow cytometric histograms of nuclei from white seeds of *βknl2-2.*
Ploidy analysis histogram of pools consisting 6 white abnormal seeds of heterozygous *βknl2* mutants show increased ploidy level and reduced nuclei count.

Supplementary Figure S11

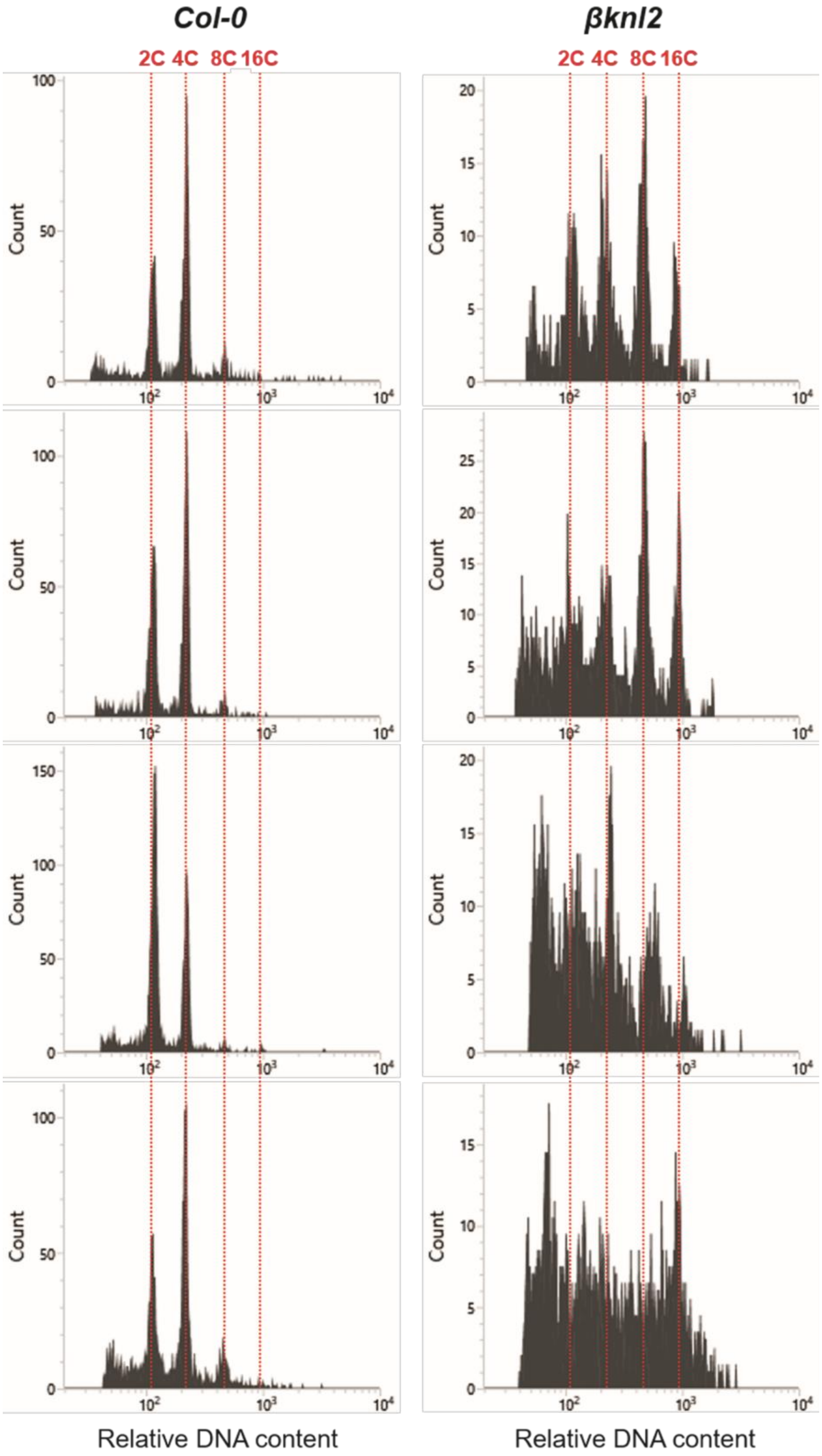

Flow cytometric histograms of nuclei isolated from single leaf of β*knl2* mutant and wild-type.

Ploidy histogram of the abnormal seedling leaf from the heterozygous *βknl2* mutant showing increased ploidy and reduced number of nuclei (right panel) compared to wild-type Col-0 (left panel).

Supplementary Figure S12

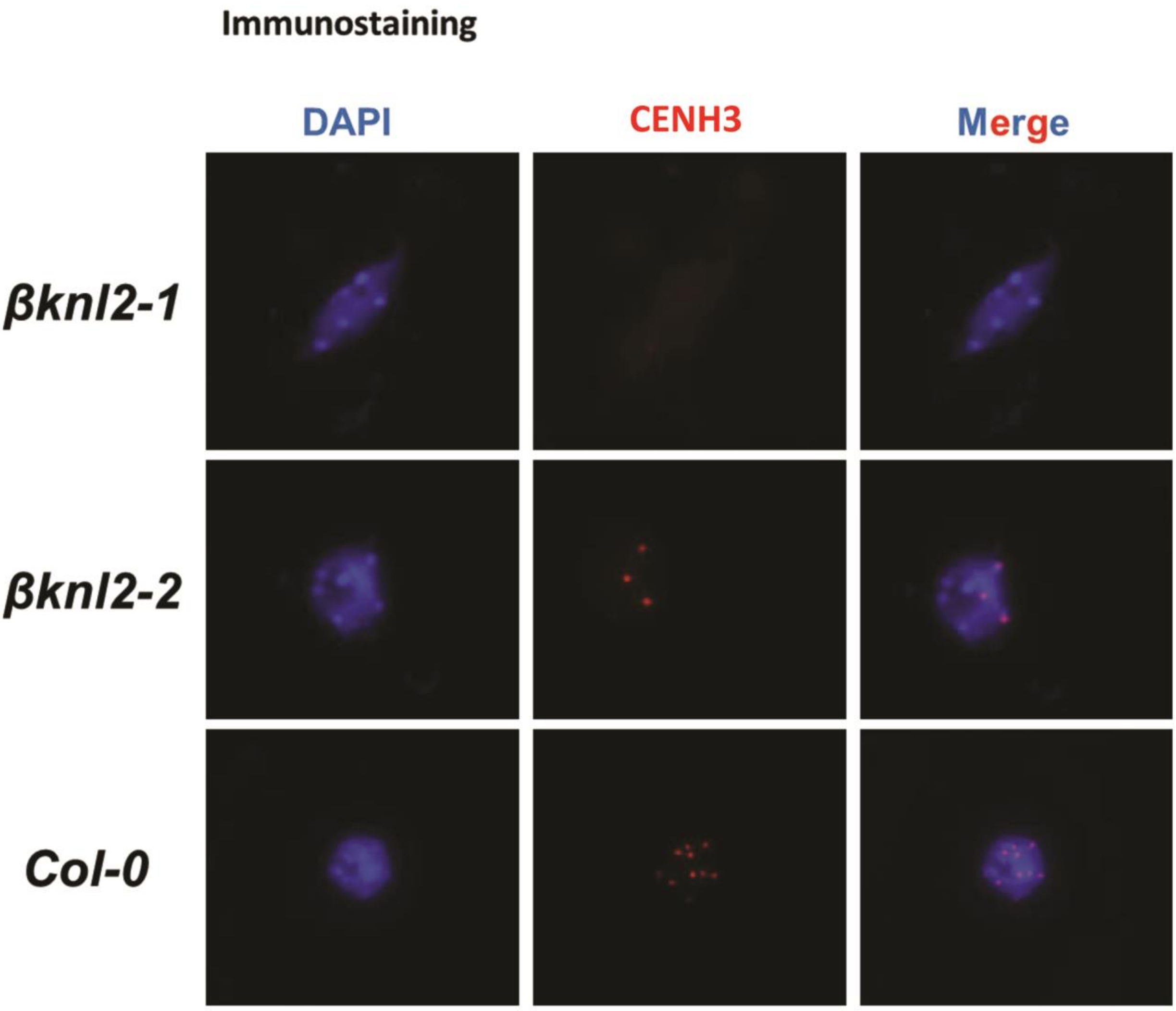

Immunostaining showing reduction of CENH3 signals in *βknl2* mutant.

Representative nuclei isolated from *A. thaliana* wild-type Col-0 and *βknl2* mutant seedlings immunostained with anti-CENH3 antibodies. Nuclei stained with DAPI (left panel), nuclei immunostained with anti-CENH3 antibodies (middle panel), merged images (right panel).

Supplementary Figure S13

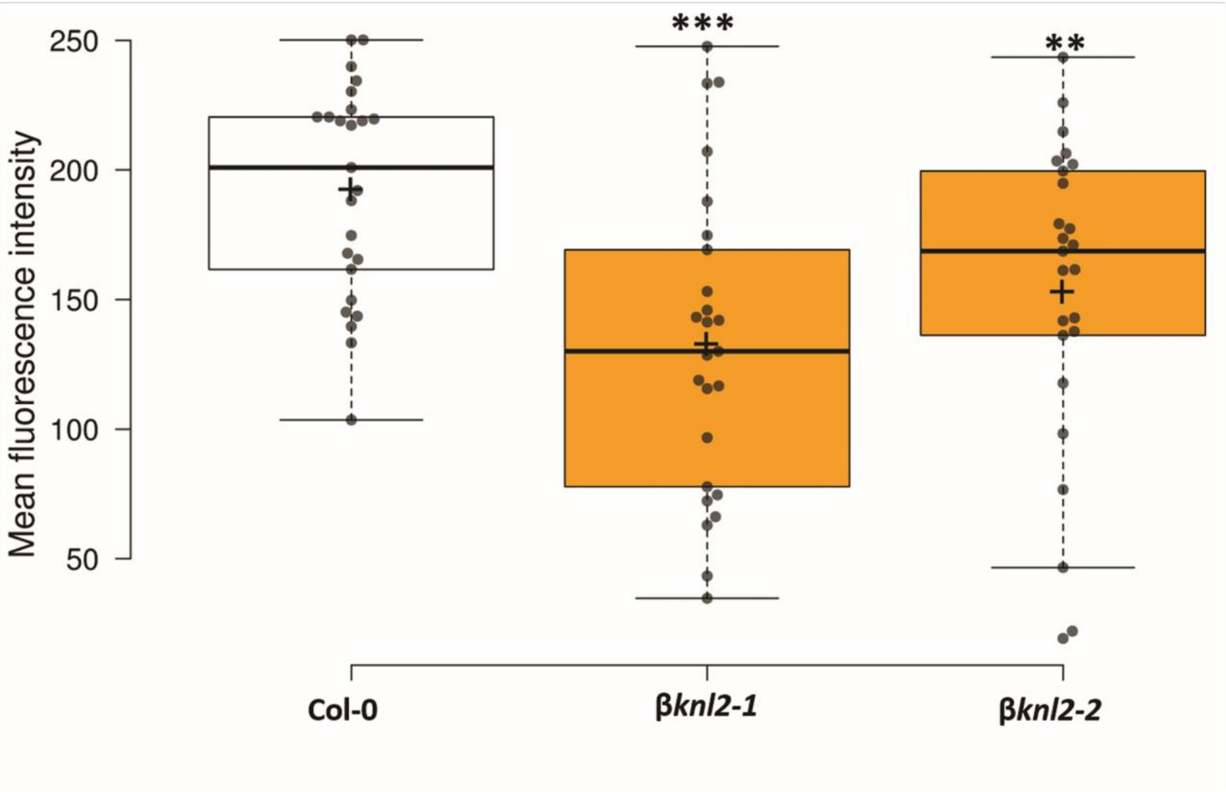

Mean fluorescence intensity of CENH3 immunosignals in nuclei isolated from *A. thaliana* wild-type Col-0 and *βknl2* mutants. The intensity of CENH3 immunosignals per nucleus was estimated for 25 nuclei in each variant.
