# Supplementary material for: Recurrent Plant-Specific Duplications of KNL2 and its Conserved Function as a Kinetochore Assembly Factor
Source: Mol Biol Evol. 2022 Jun 7;39(6):msac123. doi: 10.1093/molbev/msac123 (PMC9210943; doi:10.1093/molbev/msac123)
Supplement: msac123_Supplementary_Data [file msac123_supplementary_data.zip › Supplementary Tables.pdf]

Supplementary Table S1. Characterization and identification of the KNL2s in plants.

Linages	Species	KNL2 number	Database
Algae	*Chlamydomonas reinhardtii*	-	JGI13
	Dunaliella salina	-	JGI13
	Ostreococcus lucimarinus	-	JGI13
	Coccomyxa subellipsoidea	-	JGI13
	Chondrus crispus	-	JGI13
Ferns	*Azolla filiculoides*	2	ftp://ftp.fernbase.org/
	Salvinia cucullata	2	ftp://ftp.fernbase.org/
	Ceratopteris richardii	1	ftp://ftp.fernbase.org/
Lycophytes	*Selaginella moellendorffii*	1	Ensembl plants; JGI; NCBI
Mosses	*Physcomitrella patens*	1	Ensembl plants; JGI; NCBI
Liverworts	*Marchantia polymorpha*	1	Ensembl plants; JGI; NCBI
Hornworts	*Anthoceros angustus*	1	Ensembl plants; JGI; NCBI
Gymnosperms	*Picea glauca*	1	https://bioinformatics.psb.ugent.be/plaza/versions/gymno-plaza/
	Picea abies	1	ftp://plantgenie.org/Data/ConGenIE/
	Picea sitchensis	1	Ensembl plants; JGI; NCBI
	Pinus pinaster	1	Ensembl plants; JGI; NCBI
	Pinus sylvestris	1	Ensembl plants; JGI; NCBI
	Pinus taeda	1	Ensembl plants; JGI; NCBI
	Pseudotsuga menziesii	1	Ensembl plants; JGI; NCBI
	Gnetum montanum	-	Ensembl plants; JGI; NCBI
	Taxus baccata	-	Ensembl plants; JGI; NCBI
	Cycas micholitzii	-	Ensembl plants; JGI; NCBI
	Ginkgo biloba	-	Ensembl plants; JGI; NCBI
Angiosperms	*Aegilops tauschii*	2	Ensembl plants; JGI; NCBI
	Amaranthus hypochondriacus	1	Ensembl plants; JGI; NCBI
	Amborella trichopoda	1	ftp://ftp.ncbi.nlm.nih.gov/genomes/Amborella_trichopoda/
	Ananas comosus	1	Ensembl plants; JGI; NCBI
	Aquilegia coerulea	2	Ensembl plants; JGI; NCBI
	Asparagus officinalis	1	Ensembl plants; JGI; NCBI
	Brachypodium distachyon	3	JGI13
	Brachypodium stacei	3	Ensembl plants; JGI; NCBI
	Colocasia esculenta	1	Ensembl plants; JGI; NCBI
	Cinnamomum kanehirae	1	https://www.ncbi.nlm.nih.gov/genome/57158
	Cucumis sativus	1	Ensembl plants; JGI; NCBI
	Citrus sinensis	2	Ensembl plants; JGI; NCBI
	Daucus carota	1	Ensembl plants; JGI; NCBI
	Elaeis guineensis	1	Ensembl plants; JGI; NCBI
	Eucalyptus grandis	2	Ensembl plants; JGI; NCBI
	Fragaria vesca	3	Ensembl plants; JGI; NCBI
	Glycine max	3	Ensembl plants; JGI; NCBI
	Gossypium raimondii	2	Ensembl plants; JGI; NCBI
	Hordeum vulgare	3	Ensembl plants; JGI; NCBI
	Lactuca sativa	2	Ensembl plants; JGI; NCBI
	Manihot esculenta	1	Ensembl plants; JGI; NCBI
	Musa acuminata	1	https://banana-genome-hub.southgreen.fr/
	Nelumbo nucifera	2	https://www.ncbi.nlm.nih.gov/genome/?term=APLB00000000
	Oryza sativa	2	JGI13
	Populus trichocarpa	4	Ensembl plants; JGI; NCBI
	Phoenix dactylifera	1	Ensembl plants; JGI; NCBI
	Setaria italica	1	Ensembl plants; JGI; NCBI
	Solanum lycopersicum	2	Ensembl plants; JGI; NCBI
	Sorghum bicolor	3	Ensembl plants; JGI; NCBI

	Spirodela polyrhiza	1	Ensembl plants; JGI; NCBI
	Triticum urartu	1	Ensembl plants; JGI; NCBI
	Vitis vinifera	2	JGI13
	Zea mays	1	https://www.ncbi.nlm.nih.gov/genome/12
	Zostera marina	1	Ensembl plants; JGI; NCBI
Brassicales	*Tarenaya hassleriana*	*2*	ftp://ftp.ncbi.nlm.nih.gov/genomes/Tarenaya_hassleriana
	Carica papaya	*1*	JGI_13
	Moringa oleifera	*2*	http://herbalplant.ynau.edu.cn/html/Genomes/2.html
	Arabis alpina	*2*	http://www.arabis-alpina.org/
	Aethionema arabicum	*1*	http://brassicadb.org/brad/datasets/pub/BrassicaceaeGenome/
	Arabidopsis halleri	*2*	JGI_13
	Arabidopsis lyrata	*2*	JGI_13
	Arabidopsis thaliana	*2*	JGI_13; TAIR10
	Brassica juncea	*4*	http://brassicadb.org/brad/datasets/pub/Genomes/
	Brassica napus	*4*	http://brassicadb.org/brad/datasets/pub/Genomes/
	Brassica nigra	*2*	http://brassicadb.org/brad/datasets/pub/Genomes/
	Brassica oleracea	*2*	plants.ensembl.org
	Brassica rapa	*2*	http://brassicadb.org/brad/datasets/pub/Genomes/
	Brassica cretica	*2*	https://www.ncbi.nlm.nih.gov/genome/70253?genome_assembly_id=384099
	Boechera retrofracta	*2*	http://public.dobzhanskycenter.ru/ad89dedc8b4674276c9b0760f29b07af/
	Boechera stricta	*2*	JGI
	Barbarea vulgaris	*2*	185.45.23.197:5080/Barbarea_data/
	Capsella grandiflora	*1*	JGI_13
	Cardamine hirsuta	*2*	http://chi.mpipz.mpg.de/
	Conringia planisiliqua	*1*	JGI_13
	Capsella rubella	*2*	JGI_13
	Camelina sativa	*6*	ftp://ftp.ncbi.nlm.nih.gov/genomes/Camelina_sativa/
	Eutrema heterophyllum	*2*	PKMM00000000
	Eutrema salsugineum	*2*	JGI_13
	Euclidium syriacum	*2*	JGI_13
	Eutrema yunnanense	*2*	JGI_13
	Leavenworthia alabamica	*1*	http://brassicadb.org/brad/datasets/pub/BrassicaceaeGenome/
	Lepidium meyenii	*6*	http://maca.eplant.org/index.html
	Raphanus raphanistrum	*2*	https://www.ncbi.nlm.nih.gov/genome/34361?genome_assembly_id=212223
	Raphanus sativus	*2*	https://www.ncbi.nlm.nih.gov/genome/12929?genome_assembly_id=249276
	Sisymbrium irio	*2*	http://brassicadb.org/brad/datasets/pub/BrassicaceaeGenome/
	Schrenkiella parvula	*2*	http://brassicadb.org/brad/datasets/pub/BrassicaceaeGenome/
	Thlaspi arvense	*2*	http://pennycress.umn.edu/download.html

Supplementary Table S2. Reciprocal crossing of mutants with WT to confirm zygosity of mutation causing phenotype

Genotype	Number of seeds	% Normal seeds	% Aborted ovules	% Abnormal seeds
WT	745	94.6%	3.2%	2.1%
β*knl2-1* ✕ β*knl2-1*	788	85.0%	0.8%	14.2%
β*knl2-2* ✕ β*knl2-2*	565	87.3%	1.2%	11.5%
β*knl2-1* ✕ WT	795	96.1%	2.6%	1.3%
WT ✕ β*knl2-1*	567	93.3%	1.7%	2.8%
β*knl2-2* ✕ WT	819	95.5%	1.7%	2.8%
WT ✕ β*knl2-2*	646	92.4%	5.3%	2.3%

Supplementary Table S3. Single silique genotyping of heterozygous β*knl2* mutants

	βknl2-1 (n)	Percentage	βknl2-2 (n)	Percentage
homozygous	29	16.0221	51	24.63768
heterozygous	94	51.9337	106	51.20773
wildtype	41	22.65193	35	16.90821
Missing data	17	9.392265	15	7.246377
Total (n)	181		207	

Supplementary Table S4. List of primers used in this study

Primer name	Primer sequence
**Cloning**	
EMD1674_fw	ATGACGACGACGAGGGCGAAGTC
EMD1674_rev	CCAACCGAAACTTCTTCTCCTATTCTTCTTC
EMD1674-attB1	GGGGACAAGTTTGTACAAAAAAGCAGGCTTCATGAC GACGACGAGGGCGAAGTC
EMD1674-attB2	GGGGACCACTTTGTACAAGAAAGCTGGGTCCCAACC GAAACTTCTTCTCCTA
**Analysis of T-DNA insertion mutants**	
LB Primer	ATT TTG CCG ATT TCG GAA C
SALKseq_135778.1-LP	CAT TCC TTT AGC TAA CGT GCG
SALKseq_135778.1-RP	CCA ACA ACA TCG TCC AAA ATC
SALKseq_091054.2-LP	TGACAACATCATCCAAAGCC
SALKseq_091054.2-RP	TGA TTC GGT TCC ATT TGA AAC
SALKseq_135778.2-LP	ACGCCAAGAATCTTGTCCAG
SALKseq_135778.2-RP	TTGCTTTCACCATATGCCAG
SALKseq_135778.0-LP	ATAGGCACGTGGATTTGTCC
SALKseq_135778.0-RP	ACCAGGCGTAAGGTTGATTG
