# Supplementary material for: Recurrent Plant-Specific Duplications of KNL2 and its Conserved Function as a Kinetochore Assembly Factor
Source: Mol Biol Evol. 2022 Jun 7;39(6):msac123. doi: 10.1093/molbev/msac123 (PMC9210943; doi:10.1093/molbev/msac123)
Supplement: msac123_Supplementary_Data [file msac123_supplementary_data.zip › Supplementary_File_2_Selaginella_KNL2_protein_cDNA_genomic.pdf]

Selaginella moellendorffii KNL2 Protein, cDNA, and genomic DNA

SANTA domain conserved hydrophobic motifs CENPC-k motif

critical residues for binding CENP-C (SANTA) or CENH3 (CENPC-k) are in **BOLD**

```
                                                M  I  P  S  R  R  S  M  D  P  R
XM_024672414.1      -AGAGCAGGGGCGGGCGCGCGCGCGCTATGATCCCGTCGCGGAGATCCATGGATCCGCGA
scaffold_9          AAGAGCAGGGGCGGGCGCGCGCGCGCTATGATCCCGTCGCGGAGATCCATGGATCCGCGA
                     ***********************************************************

                     Q  Q  Q  A  C  S  C  H  S  C  A  T  K  H  E  Y  R  G  K  S
XM_024672414.1      CAGCAGCAGGCTTGCTCCTGCCACTCGTGCGTCACGAAGCACGAGTACAGGGGCAAATCG
scaffold_9          CAGCAGCAGGCTTGCTCCTGCCACTCGTGCGTCACGAAGCACGAGTACAGGGGCAAATCG
                    ************************************************************

                     H  K  F  F  C  E  A  G  G  A  A  A  E  S  G  H  W  S  G  C
XM_024672414.1      CACAAATTCTTTTGCGAGGCGGGAGGAGCGGCGGCGGAATCTGGGCACTGGAGTGGCTGC
scaffold_9          CACAAATTCTTTTGCGAGGCGGGAGGAGCGGCGGCGGAATCTGGGCACTGGAGTGGCTGC
                    ************************************************************

                     G  G  G  G  M  L  F  P  A  T  P  L  K  R  E  M  T  L  R  R
XM_024672414.1      GGCGGTGGTGGGATGCTCTTCCCGGCCACGCCGCTCAAGCGGGAGATGACGCTGCGGAGG
scaffold_9          GGCGGTGGTGGGATGCTCTTCCCGGCCACGCCGCTCAAGCGGGAGATGACGCTGCGGAGG
                    ************************************************************

                     D  I  A  V  E  S  P  P  C  S  T  G  D  R  A  S  L  W  R  W
XM_024672414.1      GACATCGCAGTGGAGTCTCCTCCTTGTAGTACCGGCGACAGGGCTTCTTTGTGGCGATGG
scaffold_9          GACATCGCAGTGGAGTCTCCTCCTTGTAGTACCGGCGACAGGGCTTCTTTGTGGCGATGG
                    ************************************************************

                     P  C  S  M  P  R  Y  R  P  E  N  Q
XM_024672414.1      CCGTGCTCCATGCCGAGATATCGACCGGAGAACCAG------------------------
scaffold_9          CCGTGCTCCATGCCGAGATATCGACCGGAGAACCAGGTTTGGTCTGGGAGATTTTGCTTG
                    ************************************

XM_024672414.1      ------------------------------------------------------------
scaffold_9          TTTGAGTGGGTTTGTTTAAGAAGAGAGGAGATAGGAGGGGTTCTAAAAAATTCCAGGGAG

XM_024672414.1      ------------------------------------------------------------
scaffold_9          AGTTTGGTTGTGGAAAGCTCTGGTTTCCTCTTTTTTGTCGTCCGGTTTTCTCTTTGCTTG

                                                  I  V  L  H  K  W  F  L  I  K
XM_024672414.1      -----------------------------ATCGTTCTGCACAAATGGTTTCTTATCAAGC
scaffold_9          TTTTTGAGTTGTTCTATCGTTCTTTCCAGATCGTTCTGCACAAATGGTTTCTTATCAAGC
                                                 *******************************

                    L  E  E  S  D  T  T  S  G  R  L  Q  V  A  L  G  G  F  Q  Y
XM_024672414.1      TCGAAGAATCCGACACTACGTCCGGAAGGTTGCAAGTAGCCTTGGGTGGCTTTCAGTACG
scaffold_9          TCGAAGAATCCGACACTACGTCCGGAAGGTTGCAAGTAGCCTTGGGTGGCTTTCAGTACG
                    ************************************************************

                    G                                                     M  D
XM_024672414.1      G---------------------------------------------------GATGGACT
scaffold_9          GGTAAGTTCTTCATTTGCTATCGGGATACTTGTTGAGCATTCTATGCTTTAGGATGGACT
                    *                                                   ********

                    S  E  F  V  K  T  G  P  I  A  Q  R  T  D  K  S  R  L  K  T
XM_024672414.1      CGGAATTCGTAAAGACTGGCCCGATTGCCAAGCGCACTGACAAGAGCAGACTGAAAACTT
scaffold_9          CGGAATTCGTAAAGACTGGCCCGATTGCCAAGCGCACTGACAAGAGCAGACTGAAAACTT
                    ************************************************************
```

```
                    C  D  G  V  E  V  R  L  M  G  S  M  D  E  E  T  T  I  A  N
XM_024672414.1      GTGATGGAGTTGAGGTTCGTTTGATGGGGTCCATGGACGAGGAAACAACTATTGCCAATG
scaffold_9          GTGATGGAGTTGAGGTTCGTTTGATGGGGTCCATGGACGAGGAAACAACTATTGCCAATG
                    ************************************************************

                    G  F  S  A  W
XM_024672414.1      GGTTTTCAGCCTGG----------------------------------------------
scaffold_9          GGTTTTCAGCCTGGGTAAGCTGGTCTAGTTTGGCTGTTTCTTCACTTGGTTCTTACTGCG
                    **************

                             V  A  D  I  F  Y  S  G  F  P  C  T  W  E  R  V  L
XM_024672414.1      --------GTCGCTGATATATTCTATTCTGGTTTCCCGTGTACGTGGGAGAGAGTTCTCC
scaffold_9          AATTTCAGGTCGCTGATATATTCTATTCTGGTTTCCCGTGTACGTGGGAGAGAGTTCTCC
                            ****************************************************

                    Q  D  D  T  E  V  S  R  M  T  K  K  V  G  D  T  K  R  S  L
XM_024672414.1      AAGACGACACCGAAGTCTCGAGAATGACCAAGAAAGTCGGCGACACTAAGCGCTCGCTCG
scaffold_9          AAGACGACACCGAAGTCTCGAGAATGACCAAGAAAGTCGGCGACACTAAGCGCTCGCTCG
                    ************************************************************

                    G  G  S  S  P  C  S  S  K  G  A  A  A  S  E  S  Q  A  A  S
XM_024672414.1      GGGGATCAAGTCCCTGTAGCAGCAAAGGGGCAGCTGCGAGTGAGAGTCAAGCCGCATCAC
scaffold_9          GGGGATCAAGTCCCTGTAGCAGCAAAGGGGCAGCTGCGAGTGAGAGTCAAGCCGCATCAC
                    ************************************************************

                    P  T  T  E  T  Q  V  V  S  K  E  P  S  V  E  T  K  T  T  V
XM_024672414.1      CAACAACAGAAACACAAGTGGTCAGCAAAGAACCAAGTGTGGAGACGAAGACTACCGTTG
scaffold_9          CAACAACAGAAACACAAGTGGTCAGCAAAGAACCAAGTGTGGAGACGAAGACTACCGTTG
                    ************************************************************

                    A  H  G  D  T  E  P  I  I  V  V  T  E  V  Q  N  E  V  A  T
XM_024672414.1      CTCATGGCGACACAGAACCTATTATAGTAGTGACTGAGGTCCAGAATGAGGTGGCTACTG
scaffold_9          CTCATGGCGACACAGAACCTATTATAGTAGTGACTGAGGTCCAGAATGAGGTGGCTACTG
                    ************************************************************

                    V  A  E  I  T  C  K  E  P  V  A  E  G  E  A  D  V  A  A  E
XM_024672414.1      TTGCAGAGATTACTTGCAAAGAACCGGTTGCCGAAAGTGAGGCTGATGTTGCTGCCGAAG
scaffold_9          TTGCAGAGATTACTTGCAAAGAACCGGTTGCCGAAAGTGAGGCTGATGTTGCTGCCGAAG
                    ************************************************************

                    A  I  V  E  E  L  F  G  T  E  R  E  Q  D  V  G  T  E  T  R
XM_024672414.1      CCATCGTGGAGGAACTTTTCGGGACTGAGCGAGAACAAGACGTGGGAACAGAAACTCGAG
scaffold_9          CCATCGTGGAGGAACTTTTCGGGACTGAGCGAGAACAAGACGTGGGAACAGAAACTCGAG
                    ************************************************************

                    E  R  T  V  A  E  L  T  L  K  Q  A  G  A  G  S  P  E  H  T
XM_024672414.1      AACGTACAGTGGCAGAACTTACTCTGAAACAAGCAGGTGCCGGATCTCCGGAACATACCG
scaffold_9          AACGTACAGTGGCAGAACTTACTCTGAAACAAGCAGGTGCCGGATCTCCGGAACATACCG
                    ************************************************************

                    V  A  A  E  V  H  E  R  P  G  S  E  K  P  P  S  P  K  P  R
XM_024672414.1      TGGCTGCTGAAGTCCACGAAGGGCCAGGTTCCGAGAAGCCGCCGAGCCCAAAGCCGAGAG
scaffold_9          TGGCTGCTGAAGTCCACGAAGGGCCAGGTTCCGAGAAGCCGCCGAGCCCAAAGCCGAGAG
                    ************************************************************

                    D  T  V  E  A  R  A  E  S  V  V  E  N  N  D  T  I  A  E  Q
XM_024672414.1      ATACCATCGAGGCCCGGGCCGAGTCCGTGGTTGAAAACAACGACACCACTGCCGAGCAGC
scaffold_9          ATACCATCGAGGCCCGGGCCGAGTCCGTGGTTGAAAACAACGACACCACTGCCGAGCAGC
                    ************************************************************
```

```
                    R  K  S  I  K  P  S  P  A  R  G  R  K  R  P  L  S  K  P  S
XM_024672414.1      GGAAGAGCATAAAGCCATCACCGGCGAGGGGACGGAAGAGGCCCTTGTCGAAGCCGTCAG
scaffold_9          GGAAGAGCATAAAGCCATCACCGGCGAGGGGACGGAAGAGGCCCTTGTCGAAGCCGTCAG
                    ************************************************************

                    G  K  R  G  R  P  R  K  N  K  L  P  Q  E  E  A  E  A  P  A
XM_024672414.1      GAAAGCGTGGCCGGCCTAGGAAGAACAAGCTTCCACAGGAGGAAGCGGAAGCTCCGCCAC
scaffold_9          GAAAGCGTGGCCGGCCTAGGAAGAACAAGCTTCCACAGGAGGAAGCGGAAGCTCCGCCAC
                    ************************************************************

                    P  P  P  V  D  S  G  E  P  S  S  I  E  E  D  K  E  A  R  E
XM_024672414.1      CACCTCCAGTGGACTCGGGAGAGCCGAGCTCTATCGAGGAAGATAAAGAGGCTCGTGAGC
scaffold_9          CACCTCCAGTGGACTCGGGAGAGCCGAGCTCTATCGAGGAAGATAAAGAGGCTCGTGAGC
                    ************************************************************

                    Q  T  E  Q  A  G  S  E  L  L  A  T  P  Q  E  V  G  Q  D  S
XM_024672414.1      AAACTGAGCAACCAGGATCAGAGTTTCTTGCTACTCCTCAAGAAGTGGGACAGGATAGTA
scaffold_9          AAACTGAGCAACCAGGATCAGAGTTTCTTGCTACTCCTCAAGAAGTGGGACAGGATAGTA
                    ************************************************************

                    M  K  Q  Q  V  V  S  G  T  N  N  Q  I  E  A  G  A  S  T  A
XM_024672414.1      TGAAACAGCAGGTGGTCTCTGGCACTAACAACCAGATTGAGGCCGGGGCGTCTACTGCCC
scaffold_9          TGAAACAGCAGGTGGTCTCTGGCACTAACAACCAGATTGAGGCCGGGGCGTCTACTGCCC
                    ************************************************************

                    Q  S  K  R  T  K  R  R  P  K  R  S  S
XM_024672414.1      AGTCGAAACGTACCAAGCGGAGACCAAAGCGCTCCTCC----------------------
scaffold_9          AGTCGAAACGTACCAAGCGGAGACCAAAGCGCTCCTCCGTAAGTCCAATCTTTTCCTTGT
                    **************************************

                                                 N  V  P  P  P  L  P  A  S  R  V
XM_024672414.1      ----------------------------AATGTGCCCCCGCCGCTGCCAGCGTCCCGTGT
scaffold_9          GCATGTCCCTGACTTGATGTGTTCGCAGAATGTGCCCCCGCCGCTGCCAGCGTCCCGTGT
                                                ********************************

                      K  Q  D  E  V  I  E  A  Y  G  L  K  T  S  R  S
XM_024672414.1      GAAGCAAGACGAGGTGATCGAGGCTTATGGTTTGAAGACTTCCAGGAGTG----------
scaffold_9          GAAGCAAGACGAGGTGATCGAGGCTTATGGTTTGAAGACTTCCAGGAGTGGTAAGCGCTC
                    *************************************************

                                                                G  R  L  L
XM_024672414.1      ------------------------------------------------GAAGGCTGCTTG
scaffold_9          TTGACTAAAAGCTGGATCATTTCGGTGACGGCTCTAGAACATTTACAGGAAGGCTGCTTG
                                                                ************

                    V  P  P  L  A  Y  W  R  N  Q  T  I  A  H  D
XM_024672414.1      TACCTCCACTGGCGTACTGGAGAAATCAGACCATAGCTCACGACA---------------
scaffold_9          TACCTCCACTGGCGTACTGGAGAAATCAGACCATAGCTCACGACAAGGTAAGGCTGCGTC
                    *********************************************

                                                          K  D  G  G  I  I
XM_024672414.1      ------------------------------------------AGGACGGAGGCATCATTG
scaffold_9          TTGATCCTCTTGGTTCCCGAACTGGCTTCTCCTCTTCGTTGCAGGACGGAGGCATCATTG
                                                              ******************

                    A  I  L  D  G  F  K  E  T  P  S  D
XM_024672414.1      CAATCCTTGATGGTTTTAAGGAGACTCCCTCGGACA------------------------
scaffold_9          CAATCCTTGATGGTTTTAAGGAGACTCCCTCGGACACAGGTGATCACAGTAAAAGCTTGG
                    ************************************
                                                                 T  G  C  F
XM_024672414.1      -------------------------------------------------CAGGCTGTTTC
scaffold_9          AGATTCATCCATCTTGTTCTCACTCTTTTGTGGTTTGATGGCTTCCTTCCAGGCTGTTTC
                                                                     ***********
```

```
                    N  F  K  P  P  T  E  K  R  Q  L  Q  Q  K  L  C  S  A  A  W
XM_024672414.1     AACTTCAAGCCTCCCACGGAGAAGCGGCAATTGCAGCAGAAGCTGTGCTCTGCTGCTTGG
scaffold_9         AACTTCAAGCCTCCCACGGAGAAGCGGCAATTGCAGCAGAAGCTGTGCTCTGCTGCTTGG
                   ************************************************************

                    D  V  T  E  K  G  K  K  K  A  R  K  *
XM_024672414.1     GATGTCACGGAGAAGGGAAAGAAGAAGGCTCGAAAGTAGTATAACCAGAAGGCTCTTGAC
scaffold_9         GATGTCACGGAGAAGGGAAAGAAGAAGGCTCGAAAGTAGTATAACCAGAAGGCTCTTGAC
                   ************************************************************

XM_024672414.1     AGTGTACAAAGACTGCCACCCTCACAAACCTCTTACCTTCTCAACAAGTAGAATGCAGAT
scaffold_9         AGTGTACAAAGACTGCCACCCTCACAAACCTCTTACCTTCTCAACAAGTAGAATGCAGAT
                   ************************************************************

XM_024672414.1     AACACTAGGAGAAGAAGATCCTCGTTTGACAGATGCAATTTACATTTATAGTGTCCCTCT
scaffold_9         AACACTAGGAGAAGAAGATCCTCGTTTGACAGATGCAATTTACATTTATAGTGTCCCTCT
                   ************************************************************

XM_024672414.1     TGTGCCAAATGTAAGAAATGAAACATTGCTGCTGCAGCTAGGTGCTGTAGGAAAAAA
scaffold_9         TGTGCCAAATGTAAGAAATGAAACATTGCTGCTGCA---------------------
                   ************************************
```
