# Supplementary material for: Recurrent Plant-Specific Duplications of KNL2 and its Conserved Function as a Kinetochore Assembly Factor
Source: Mol Biol Evol. 2022 Jun 7;39(6):msac123. doi: 10.1093/molbev/msac123 (PMC9210943; doi:10.1093/molbev/msac123)
Supplement: msac123_Supplementary_Data [file msac123_supplementary_data.zip › Supplementary_File_3_Phylogenetic_KNL2_align.pdf]

>Colocasia_esculenta_MQL87308.1

----MGNSSS--SVPPR--------
----------------ASEA--
D---------------------------------ERSHREA--------------------------GGVA--
----
--ASPPS----------

---SSTTSC------
------------ASSFEKT--
--VLLHDWWLIK-AEDE--------CSR-
ARLAVG--GLATT--------GFQFFE-------------------------PVSSP---------LCSR---
------------------------------------LDKK-AV-RIF--------------------------
----
KSALIVKRYDGYTIETADGITVRIQGLI-------NASRTHQN-GFPSE--------ACNH-FRIGF--
PYGW-------------KDYA--
-------DRYFQTDTKGNGAPTCASD------IHDCSMDSANTSS-----------YCFPACSKE--------
--------------------------------------FPVGRIVDCFNSICKEIVDGAE-------------
--RSTDT------------------
-----AQNLC---------------KNNDCK--
--ENTNGIVAQT----------NGSTVVSDGNSNVAEENAYMATNN--DTLGGLKQVSSHVSNDSL-------
-LC-------------GNMNNDSLL--------------------------------------
EEVTGFQRESLAT---------VVAGPDNEKQQLGHASI-----DVDGLMKDIDGNTCCINAPSA--------
--VDSGA-------
KVLLSKSLPENWEESVSIEPNENHPSNEAEQVVHLSVTTL---SHESDVE---------HMNSP--------
RVQLNPVSSDTACN----AKRACNS--
---------------------------FNNNYVRY--------------------------------------
---------RHLSVSQN----------FAGINHNQ------------GESRGFSHLDG----------
AKVGFTN----------------AMHLEV---------SDVTGNSSHMAS-----------------------
-------------------------DHTLSRHSDENTVG--------------------------
SVASMMAPSNRTPA-------------------------------KQVAIIDRG--------
RYEKISPSSCMKDL--KGKFGGYLNSATKVPSSMFVKDVNHCPDNWEMRLIDSNTM--DL-GSEEVLAE----
---C--FYNEANDLQPESSKKLPSHTSDSAYGPSNIDN---------
NPEENSTDLPDVGMTVSESRNKQFFLR--
---RSERLSKLKIPAEDAERSL---------------EH-NKGNLHETKC------------G--
RPSNEDRMHIDL--------------EGNNISSKKHDLGASQEELM---------------------------
-------------------KSQDRCVSHSQEKKK-NGRNK-NQSVADTGKNR---VLSFSDGYSFMNS--
ENIVRDTHDLMRNR--------IEADAQNLDMPTGEQSNSRVGISC---------------------------
----
VAERAKQVRQL-------------------I----------------------------EAD-----------
--------------------------AQNL---
---DIPTGEQSNSGVGI--------
---SCVA--
EYAKEVCQPDV--

-----------HVNAQPNDGKSPARPARRS---
---RKKSH---------------
------------------F------------ASHVGKT-----------------------------------
---PL----------------------
-----TRQ-RT---KELSL------------
---------------ASPALLNF--------------------------KRSR-SG----------------
RLLVPTLAN-W--------CQQILYDVVLFLWMF----------------------------------
KFSQDGSVAGILT-----------GV-DAIKCDRRVGGQGDAGARALGR--GRR-RGGGVRARATP-------
-------------------------TRSAATPRLAS-------------------------------------
--------------DGDGRTRRLAGER---DG---

AALVGGRGGG---
--------------------------------GAGGRAPG---------------------------------
----
RPAMARG--
--------------------RAAERLRA---

----------IGFGFGEGEL---GFL---

--------------------V--------------------
>Phoenix_dactylifera_XP_008806422.1
---METEEKTTPR--------------

KRDEAATAALGSS--VTPMG---
---------------------RSSS----------STTPGPGGSNP---------------------------
----
TPG--
--TP-----

FTRF---
SSS------------------SSSDKNS---
---VLLSDWWLVK-AESG--------
AGG-KRLAVS---------------GFT-------------------------------S-------------
R---QQ-AV-RIF----------------------

SSAPVVKRYDAYTLETEDGITVIIQGMI-------NKIRTRDN-GFPPE--------VCNR-FLVGF--
PYNW-------------DYYA--
-------D-EYLRKSSTCSSNSSSSAGSGE--QSKDSAVGAGGTS--------------PVCVKE--------
--------------------------------------FPVRRICDIL-------------------------
----------------------------ISSGDTLSRNFT----------ENLKKFFRDPSL-
NQILQKPFFAGEKCSEHEM----------------DKSDQK--------------------------------
-----------YENDKGFA---------------TAI--------------------------
IHDAEGHVTSHA----------------------
EEGGRRNDLHAGNVLEENSDFNHDISYDSLMHESRETDSQPAVETTCQTETGTAMQREMLL-----------
TKTREVEKDNDLASSVPMDCDNVDHVPDCIEGK---ESVESR-------------------LDSCNV------
------------------------------GM-------NCLLKGSLPKES--
ILSKHEHEEEPGDDAVRRQHH-------------VE-----I----LRIP---NV--------GVSDDLLVP-
-----KDCDPKNAN--------SLNVKMSVGSE--
--------------NYLVS--------------DVT-------------------------------
KSLKAP---------------MTQDD------------RTS------------------
SKISSQNEVMVHSTVLV---------FQG----------DADSH--HVEG-----------------------
------------------------------RGRDLN-----------------------------NL----
KSPNVGI--
------TRISKDSSSN----------------EIDNMCH---------------------------------
SIFEKIERCKFPAARDPT--------------------------SPIGSAADMK---------
STRASEVKKSP---------------------------------INKHLTK---VVKKAGRKK----------
-----ESIQKKDQKCAEC----------------INSKVCLH-----------------------------
GTEELSST---------------GNVQDDVPLITLRTRNVHRNSSASSEGK-------PVESSTNVKAN-
ALSTR-MK----------------------------SSKKKGYDNQMAG---------------
CSPEVEKIGVGSALMDHNIVNAANDTKHASRHKHTQG------------------------------------
-VMHRQ---------------------------GHLDNSA----KEQSSSNKTL-------------------
-----------------------------AMS-------------------------------------GEN-
----
---------------------ILKI--
---------------------------------TNDQNAKISEV-----------------------------
----

---LIMEQPSGEKTLHTTRRK-----------
--TAWRP-------------------
---ISSAYQT--

---------PN--------------------------TRG-KA-----------------------------
----------------RQLSM--------------------------ASPESLN------------------
--------LKRSR-SG---------------RLIVPPLAN---------WCQQIIYNA--------------
------------------------------DGSIAGIRGA-------------HVLN---------------
------------------SP-KIG-----------------------------SQSEPV-------------
---KKR------------------------

--KKFT----------------------------

>Ceratopteris_richardii_gene01725

----MATHGLRLDA-------------SARNRFCSDVTHTEN-----YCNHCCG-HHSLSSPTRSK------
---------------FSSPEISWVH-QQK-QFARDPDDA-------------FRRGF---------------
---------------------------------CSA-----------------------------RYFV---
----
--QSSVQPP-----------
---TPHQDYE------FLYPASTSMSE-----
---------------GTPGGASRSFE-----------------PCSSSAWL---------
SSARGFNLERNSEREDLCTIGYETCSVQSN--
------------------Q-------------------------------VTLYSWYLIK-CEGA-------
-SDS-VGVSVG---------------GFL------------------------------G------------
TG---GI-EKF---------------------

ESASIVQRMEKCILMAQDGVKIRLAGLI-------NGSTSVAN-GFSQN-------LVES-FLSGF--
PYTWNHLL-------YSELRA--------------SKMKQPDHQ----------G-----------------
--F----HEGLSETVEVSQLSFKGSH--------------------------------ADQLKEIP------
-------------------------------------F----------------------------------
--------------SNQ-------VKDHHGYPEVLAEIELYAK------QTSRKIQIE--------------
-----DKNV--------------EFSNQTVS--
TDNDDFKQGLSKN----------DVVMHS-----------EM--------
GASLQQCSLDASLTNIEMKGKNEELNNQAITTDHGNSEIGTFKDDL--------------------------
-----------------------------------LITSDTVTPLE---------------------
KDILNSSHSI-------------------EKDLEAS-------------------------------
RYNGQFTDHVRSNSRRTSKAQSPHT------------RKKGGTVNNQSCV----------------------
----------------GSSPAIQTN----QCTRMTRSM----------------------------------
----
--HKVSMHQDHSKNNDSLLPDRVDNG--------------GNAGSYSG--
KNVAGDCHRQASFTMSQKNNKG--KSDHTKTLNTTSCA------------SKDGDE----------DITQE-
---TESKLNVDANLDPK-----
--------PV----------CLEKVVTISSDKN---------------------------------------
--------------DDDKDSDR----QDMPMLARKSETNANVE-----------------------------
-----------------CSTLKANIKKHRKSNKE--------------------------------
SDVAVKDPGQITR---------HLEKEVG---

```
TASSDENDDDK---------------DH---------------------------------------------
-------DRQDTPMSVRKSEANANMEG----------------------------------------------
-STVKVNTKKHRKSN-------KE-------Y---------------------------DVAV----------
-------------------------------------------------------------------------
---------------------------------------------------KDPGQATRHL------------
------------------------------------KKE----------------------------------
---VV--------------------------------------------------------------------
----------------------------TVAS-DK--------------------------------------
------------------NDDD---------------------------------------------------
-------------------------------------------------------------------------
-------------------------------------------------------------------------
-------------------------------------------------------------------------
------------------------------------------------------KDPDRQDMRTSVRKS----
--------------------------------------------------------EANANME----------
--------------------------------------------------------------------
CSTVKVNTKKH--------------------------------------------------------------
--------------------------------------------------RKSNKE-----------------
----------------------------CGVAVKDPGQSTRHLDILPALPSPR-----VPVPKQLVSE-----
-----------------AFGLKTSR-SG----------------RLLVPPLAH-W-------CGQSLLRDK--
-------------------------------------------DGGLLQYLM---------------------
-------------------------------------------------------------------------
-------------------------------------------------------VQKTRK------------
-------------------------------------------------------------------------
-------------------------------------------------------------------------
-------------------------------------------------------------------------
-------------------------------------------------------------------------
-------------------------------------------------------------------------
-------------------------------------------------------------------------
-------------------------------------------------------------------------
-------------------------------------------------------------------------
-------------------------------------------------------------------------
-------------------------------------------------------------------------
---------------------IIL-------------------------------------------------
-------------------------------------------------------------------------
-----------------------------------------------------------------------
>Anthoceros_angustus_AANG012187
-------------------------------------------------------------------------
-------------------------------------------------------------------------
-------------------------------------------------------------------------
----MLERGGDGVPPC-----------GPRGRQWSSRLPL------F-------------
TKPSSRCAGLRPHAPAQHDRRR---DACSREFDCGEDSYDAFRQQQLEPQPEHQQHQQ-------NQHCGF--
------LEQSGYDEAEGMHGS-------------------------ARAP----------------H------
------SPFERR-GEGC--RCWSC---SSRVRFRS--------------------------------------
------------------------------------------------------------------
EDSEGKSPE---------------------------EHRSW-R--RDAG--------------
RRPKECEHDGCRCRAGLRGRGSVSPCQSDSFRHFSRLG-P-----NTPSDTSTS-------------------
----GSGRQGLDELRARDVPLMAWH------------GRQRPAPAEKQ-------------------------
---------------------------------------------------------------------
VSLRRWFLVR-CDDKEAEDV-VDATN-PRVAVG---------------GMH----------------------
--------TS-------------------------------------------------------------
ESF------------------------------------------------------------------
ELQTVRSTMIVERIEKSRLRTKDGVEIRLMGCI-------DAETTIAN-GFSVK--------VVHH-FLTGF-
-PFVW-------------KHFL---------------------------------------------------
--------ELFLSNDTTPKS--------------------------------------DIPLYLPETPVT---
--------------------------------PDAAATGFKFGGR-------EGVSCRGVG------------
---------------SNEV--------ESPKTVAILSPLPATPTPATQTCVESTETQELD-------------
------NLNI-----------VAAMLSPAPAT--------------------------------------
PTTCLESTEKEELENP----------NVVATSS------PL--------------------
LGAQVLEDLGCHDAH----------LDRQEETGACTSQL--------------------KGKRSRKGRVPPV-
-------RVYKLRSSAGELEHGLPDRLGTTRKRGVKRQ-----------------------RDIDTATGYR--
```

----------------IMDDNDEG--------------------------------PAGGSST----
NGKEKEDQVERSGN--------------NSCGGLERQMEAPAGASEGSDLE----------------KN----
---IGPSSTALEKDGRERVVRPAEGELSNCDIL---------IHVNEV------------------EV-----
------------EQPPKA----------RGNCMGG------------VELQIEAHM-------VH--------
---------TEVSTNLETL------GPSIIASSKDEIGLEDHLVTQLEDEAQLEEM----------
LLDRELQEER---------------TGDGEEEDRT------ELVGV---------------------------
----------------------------HPDIEQGDSR-----------------------------
ELEEVHPGRQEEDG----------------------RVL---------------------------
EEVPRDMQQDAGRGLDEVPLDIQRDATSEVEDLHV-HIQQNAG-TELKDL----HL-
DIAQYAGIELKELHFDVAH---------DADRELKDV------------------------------
CLDRLDKVGRELDEVRP----------DRGIEDGI---EFEELHP-----------------------
DRQGASGEELGEVRTDLEQ---------------ED-----------------------G----
SHLEEVHPDNGWEGGLALVVFHDRGSEGGAKPEEHPHAGKITSSNE------------LG-------------
-----------------DSHPEVGGRKGVKRPREETFDAPKSAPT----------------------------
--EDID-----G-------------VDSAGSDKVSRTG-----------------------------------
----
NRYTRRGSK---KRNCK----------
------------------------VTPKNP---
--TIER-------------------

-----KSDMQKQLAS--

---SGCQSRR---
---SKTSM-----------IPPPLPTPC---
--VSVTTDLVSQ----------------------AYGLKISR-SG----------------RLLVPPLAY-W-
------RNQTTARDK---DGGIIAIMD----
-------GF-DENE------------------------TPDTGCFNFEPPKEKRAV-----KLQRQL------
---------QQAAEERQ---
QVTRAKASKKRRQAVAK--

>Azolla_filiculoides0019.g015039

----MEALLTTI----------------------------------FQHHQ---QNHIPSGSPNFL-------
----------------KPTEIH--------QSFRQRGPSDL---------C------FS-------------
STPSYPSEERRR------------------RISSGRSSEPSF-------------------------
SKFSASEG---
---ITAYN--------------VSENAYIPR----
---TPQTED----------
FGMASSSPY-------------------VQPASNAKFI-PSAYTQPRNCE-----------------RQDF-
RMASAVNG--------------SDHHMTAPQKQ--
--VKLYRWYLIK-CGTSS---

--------VEIAVG---------------GLL-------------------------------SPGT------
-VGSR---RQ-------------------------

EMAAITKRNDKCRLMTKDGVEVRLTGLI-------DDCTTIAN-GFSVE--------IVHL-FLSGF--
PYTW-------------NHLL--
-------QNEFQTCTIKNPICLGSGT-------------------------------DALNSIETSELN----
--------------------------------------FE-------------------G-------C-----
--------------ANKE-KCLPLLYNSQMDTSAPSGKKVDATAYQ----ENNTKLPLDEIL-------
QLSASSAMGGQDLQKYGT-----------HTAPST--------------------------------------
---HNTMELHETTSSS----------QKSKDE-------------------------LHENAPVEKTDNV---
----------LGVPREIGEAHVLDSPV----------------IPKENVRKTRDG------------------
REKS------------TQKSQLPKRVT----------------------RSMQRASRHM--------------
-----DGNENKI---------------------------------MNGA------------------------
---TN-------CADASENIQRTN--
LEAKDVNASF---------------KVITDT---------------------------------------
PIKE------------------------------EAV-------------------------------
AHLKKSMR------------KSGKNN------------RQQNDMKED----------FVDSAQNLNS------
-------FVDKDVDI--------IMDTIEH---
----------LEGDEHTPQESE-----------------------------HAAEVLIDSSNGTI--------
--DQGKGTVKNSAQH----------
SINIESMPDG--
EMEGKIHSRSEGR--------------RL-------------SSAEVIDFPNCFQEICT--------------
--ES---
-----QSCKEKQT------------EIFPPIKRKMQ----------------------SRPGEKRLSSSL---
---YFP------------------------------------NSSQETETERHGEKE-------AEMIPSL--
--KGKAQLRSGERTSCNSESDL-------

--------------------TNSLEDEKTG---
--TG-SQS-------------------------------------HEDN------------------------
----
EMHG---

------------SC--RL---------
----------------------G--
----------L---------------ISLTKGKIQSISEER--------------------------------
----------------------------RLSSSEVIDL-----------PDC---------------------
--LQEIGDESQSH----------------------

-----------------AREMRTP--
CRTGM-----------IPPPLPSPS-----VPVSKNLISA----------------------AFGLKTSR-
SG----------------RLLVPPLAH-W-------CSQSIVRDK----------------------------
-----------------DGGIIAISNGSMNMDTS-ESGY----------------------------------
-SKFRPPAETNLQ-----NLQKRL-H----------EAAVEGLKKKT--------------------------
-----------------------------MNKKKK--------------------------------------
----

-------------------------VKRN--

>Azolla_filiculoides0032.g024707

----MQTQKLIQISK-------P--------------------------------
FDQSRSQPSFLRLLLPLLLKTKQDCDHLHCDHNNRSQKNK-------NHSNRAPSRN----------------
-----------MDSAGNSSPSTTKNKAHC----------------------CHSTPIST-----HSM--AAH-
-----------TPWCQNRGQNC---
----------------------------A-------------QSTDI------------------------
NTGDGIEPP------------------------------------YRSL--------------LRPNQSS---
-----------------------------------QSPFAD--------------------------------
---YI--LSPQYDH-----------------HHQHRQS-----------------------------------
----
SWGDISPPARER-------FASRDSNAVGALGFASHECDDLGSRGFA--------------------------
---PSSKH--------LSRD-FDDLGACGFAPTRKGFSCEINDSGSFGFTPGSKIFS----RELGP-
RGFMSHEFNGLGPHGFASRDIDNLGFRGFASPAVEEIYEGICAENYLYILESNLVLEKANYASNLYRAIDNET
FETTAIIKRLEERKLLSKNGIVVNLVGTI-------NDCATIAN-GFPHQ--------MVQL-FLSGF--
PNIWNLLVGMAFPV---KQLG--ATSC--
-------SEFLGGGVMPESLTLE--

--RDTKH------------------
----ARQSL-----------FSECKEKLPSSCLDFG-------------------------------------
--NRAQADTEKNSLV-------DGN--
------------------------------------I------------------------------------
------------NIQITPKRRE-----------------------SNLDSETTRD------------------
--PNEGI----------------------------

--------MQDNPLHI-----RMECYNENKEEPVDADIAININTLEEKFLSKNS---------------
KLDVDK------C---EAVTTCADIGAAADSCG-----VLNQSLK------------VAC-------------
-IESPIRDACETEQ----------------------------DMGAVASVDGSRKR-----------------
------------------------------------KVRFRSKN----------ESVAGSSSQTQK-------
---SSETHSKRV-----------------
-------------CQEPFDKLDQEPEST---
------------------PAQNMDMETQLL------------SK-----------------------G-I--
SHKKGIS--------------------------KKRGKPKKKVRL------------------------CL--
----------SKPRRSNKCMISKQKQGMVKNPVSLHEE-EK--------------------------------
--MDT----------------SENPKVASGEQEDCRGEMQQ--------------------------------
----
TKNIDDDRNNGNDP--ASC------------
-------------------------NEEN--
--YMEA-DQ----------------

---GSQWENKNCREMAHQ---
-----KVDTKARIE---
------------------SCVSRCSLST---------------------------------------IN----
-------------------------------------SPE-----------------------DECSVKD---
---TPTDISACKVARNR--LPPPLPMPT---
--VSVSNQLVSK----------------------AFGLKTSR-SG----------------RLLVPPLAH-W-
------RNQSIVRDS---DGGIIAIED----
-------GSADTMG-------------------------VDTGRFNFKPPLEARAR-----KLQKWL-S---
VTASKCF---MQKLNSR--
ANKKKR---

---------NVT---

>Marchantia_polymorpha_0034s0011.1.p
---MDNIQRSHALG-------------

RREGSCKQRATLMAMHGG---------------LHSRRDSG-----------------------
TLGRSEPCC---------------------EATFSRNNAGVTA----NSVATP--------------------
-F--------------HLPNGRHGSHSSFN--------------------CRSSCHFHERFSVHD----EH--
---AESARRAAKFAQT----------SC--------HRRTATQSSCDCGHCQGRDLRED--------------
----------------------SEAQVS-------------------------------------
EENVRYRRGASVPD------------------------------------TQAG--------------
TGPSHAEE-GCGC--CHPSGSVLGRNDGGDDFD-DRIR----ALGGTPCFSLSDDLPYRATANAF--
MSKPSSVSRKGSPDLSR------IESMHR------------------ALAATKASVK----------------
--Q------------------------------
--VILYGWFLKRPSSSNGSAS----CSAVPQVVIG---------------GYK--------------------
-----------SAGM---------SES--
DYF--
-ESGLIVQRLERWRLQTSHGLEVGLMGMI-------NVSKSVAN-GFPEA--------LVQC-FMIGF--
PSAWSYMI---------SNFG---------MGSLSDDKTDLPS------------------------------
-------SSAVPSNATGQAQDPRNYR--------------------------------REVLLNTAPLE----
---G-------------
----------------------------DPVRIILSSSLFIAP-------DEARMIALRRL------------
---SSSNNL-----------QGGVESDATSAPQSVNT----------------Q---------------
EGLDRARNDRNSCYGA------INTGNHSASH-D-----PM----------------PSRLVERAVTQEV---
-------------LPEGGEEHS---------------------VRLGDHNKTRNGSDSCY--------
DAINTRNQVSSHD--------PRPSRLSERRATQVVLV-----EGDEIGDGL--EDVVSSPTPK--
DEVSVEGAVAVDLDVSERTPNEEAQPNIDWGEFLGDGNSRDQE-------------YEAGL-------
KLCDEANVTQEGTVHVAVVGAVDTDCNCPGVSYLKLTCASCG-CTGIDLD-----V-----------------
--TTGASQDGESKAD-LNIKVSSIEFPNRG---
-----------NEPAAG--------------------------C--AELS-----------------------
-----------------------------EDSSDFTTK-RPFQGPVSEEGEEKP-------------
SKMLCETH---------------ASSLEI----------RLPAT-----------------------------
-----------------------AAIGNDANGSDSGQPIV-VGTEAGLMQDSIPC---------
TPLQESKKRSRNSTE-------------------------------------TPG--------
PTEETILMCNILSH--AGPSVLPSQSRTNAPVSEI--------------LLNDIDRE-SV-
DTAVEPCQEEVIVS------
AKELAVDESTTRKKRKKSVGAALGRPSIHSVDSDPGLPRDDGLLATEVFSSNSQMTSFVSLRRS---------
--RRRSVAESSSRSQSGKESSEVLVVG------
---DE----------------------------GGKESIHV------------L------------
KSNREPKGGADV-----TYFHKDVK--EFRVPVVAC---------------------
RNEAGTESNHAMKSDQKLETDEENGCKE---
-----NKQVVTPGNNKGSRKSRKYY--
--KGRRKKSTKRV------------
---------------GFV------------------EKD----------------------------------
---TGMNH--------HS---G-------------GSRPE---------------------------------
----------------------------ELKS---
---------K------------------------------LHKDSLGTSACG---------------------
--ETHIEGGTDH-------
---C-------------------------------
------------------L--
--QVPREIPAPISQKHQRSSSRRNYR-

---------M----------------RRKDHDR-----------------------ETDLQTSGEGQGD----
---AADG--GL------
NQGLEDADKAAKST--------------------------KRLKNTAERSFSGSRR-----------------
----------------------ASPKVL------NRV----------IQKSQPQRR-KNK-------------
-------------------------------FSASI-----------VPPPPPAPRVVD--KTKVPEAFGL--
------------------------KKSR-HG----------------RVLVPRLAT-W-------
RNQSIKYDM---DGSIIAIAE----------
-GF-EGAE-----------------------GESGTGCYNFKPPTDK--------------------------
----
EARTMQRKLQKAA--

ELART--

>Physcomitrella_patens00338G00350

----MSCQ-----------------LQPGAEGSLSESVHFPPTSSFLEHQGYQGDVRMGMAPPLS--------
------------------------------SFSRAPPPRSPFLHD---------------------------
DGRAGSRYVH--------SGGFDRQ--G-YDYAVHRHSCGDH--
LELHSGSLLEHLLLQENQKALRIASLVQSQASGCLSGCLSCGLGEKRLRYKDSEERMVADQRHCCPRSVGEEP
SRSVNLGAEADPVECADEWFRQPRPRESVGGVGERSQAKISFFEDELGRSLCQERRTVFPVSRPVLSHSQASH
HIHDHQDHLVGAQESSVPI------------------YGFSDGDG-RVRQ-----RWPS--------------
QRPAYSGN-GVEVDGWQRATRSVSRVGSVTATEQDRLSLPDIAAGSLPTVLVGS--PHRPVGDA----
SRPTQSARTTSQNLLRMS--NSVEPSSRD-------------KPLQEASTQRVVT------------------
---QGP-----------------------------
-VSLTGWYIMK-VQTADVGRVVE-----TKVAVG---------------GRL---------------------
-------------V---------QG---GE-HV--

KTSPIVNRLDFHKVVTEDGVEVSLEGSM-------DLETSTAN-GFSPG--------IVQC-LCNGF--
PYMWKQLLRVR-PVGMSSSLA----------VSES-VGGLHP-------------------------------
-----VASKCVSEDESQGVVDPNICKTIPKG-IPGGSRDCEGNSTVGDVGSDAVAAVDEPSVIQR--------
----
-----------------------IASDAVGSKRVETEAVVTAGA------ETSTAVETEPVDPG--------
GVERCEPPNAFEIE--AMILGGGTTVRDEPPN---
---ADGKGTVEPE---GEGIGRANTSNA---IDEGCPLDVPSQQACDPCPTNFDPVPVSNPLDTDSL------
-----------GKEAESGPTVDTTF---------------VTMRRGRKNKKGPPQPV----------------
-------------RSSARLQQRRTKSAESM----------------PNLFKSSGPAQVEQVTVSG--------
-EVTRDNSGE-------------------------------QELGEILPDVQRAANGREKITRTSD-------
-----------------------------------I-------------------IGEVLKDLQEE----
DMEAANQGFV--------------GIVVDP--------------------------------------
QPIQV------------------------------EIS-------------------------------
VQVQDSGKNLLKDRDDVEVPKLNENDATE----DRMEIEENKEMAPA----------
ASSDFENEKSEETAQAAPAATFKLVNEEMEEAAPVTPP---ELIQR---------------------------
-----------------------------KVQSRVDSRYKP--------------------------
SSEALPQASEERVS--
PELNARMTRSLKRR--LRLSAISEPVADVAPVT--
----------------RSSKRLRRP------------------------------
VPESNSEPSISHQVGDPLVQPVSNPDLSHQVEGP----------------------------------
SMEPISN-PVTPHHGEVNG----------FHARVRDLSTRRAKAVNCSNCKKPC----------

```
SSQEVIKYKNR--------------------------------------------------------------
-----------------IASIGATPEHAVERTMKTEQE---------EY------------------------
----------------------PPLEPQRTESTPGTSSLPKK-------------------------------
--------------------------------G-----------------------------------
ETNTSSAKRR---------------------------------------------SGRPKK------------
-------------------------TKSA--------------------------------------------
--------------------------------------------------NLPA-------------------
-------------------------------------------------------------------------
-------------------------------------------------------------------------
-------------------------------------------------------------------------
-------------------------------------------------------------------------
-------------------------------------------------------------------------
-----------ERRTTRR-------------L-----------------------------------------
-----KLDDSGEGPSEG--------------------------------------------------------
-------------------------------------------------------------------------
------------------------------------HNPVP--------------------------SKQ---
-------------------------------------------SSRRTSI-----------
MPPPLPSPRVQGGLKGKVPEAFGL--------------------------KTSR-SG----------------
RLLVPALAY-W-------RSQSIEYDK---------------------------------------------
DGGIIAIFD-----------GF-QATP-------------------------SDTGCFNFTPPQEK-------
---------------------HAKKIQEKLCK-----------------------------------------
---------AAATVKKRK-------------------------------------------------------
-------------------------------------------------------------------------
-------------------------------------------------------------------------
-------------------------------------------------------------------------
-------------------------------------------------------------------------
-------------------------------------------------------------------------
-------------------------------------------------------------------------
-------------------------------------------------------------------------
-------------------------------------------------------------------------
-------------------------------------------------------------------------
-------------------------------------------------------------------------
-------------------------------------------------------------------------
-------------------------------------------------------------------------
----------------------------
>Picea_abies00016756
-------------------------------------------------------------------------
-------------------------------------------------------------------------
-------------------------------------------------------------------------
----MEDPF-AF---------------QAENSAHSGVTCG------------CSD-EAEYCTQND--------
----------------RRFHNGFPG-----NSIQRPYSSPF-------------RTLF--------------
NSPVHHR------------------------------------------------------------NQL---
-------------------------------------------------------------------------
--------------------------------------------------------FRRSTR-----------
---------------------------------------------GSNLA-----------------------
-------------------------------T----------------GGS-----------DYVH-------
------------SVSSQKR------------------------------------------------------
----------------------------------------VNLYKWYLIR-VVKDCPQND-MINDE-
AQVAVG---------------GYT-------------------------------T------------PD---
LL---------------------------------------DC-EKF--------------------------
---------------------------------------------
RTAPIESRIEKCKLRTVDGLDVLLMGTI-------DELCTNDN-GFPFQ--------VIHL-FLHGF--
PFNW-------------N-HV----------------------------------------------------
-------------------------------------------------------------------------
-------------------------------------------------------------------------
--------------INI---------------------------------SNGKP------------------
-----SLNMHI-------------------------------------------------------------
LPSSKMKVCASKE----------K---------------------------LEKPDSECPVVQVDDT------
------------VTDKTSLLEPEQ-----------------SLYKAGSPR-----------------------
-------------ESSVGCSKRVRHK--------------------PQPARLSKRS-----------------
```

--ICGKLSTGGKPEK--------------------------LFTVV-------GSQEKSDILKESE-------
--TNVS-----SGAPNKSVKQE-------

--AEITPK-----
--------RRRD--
KDPVKT-EM-------NVSQR--Y-----KRTK--
----ESKKQIDSGS----------------------------SLDEAKLVQSTGT------------------
--VNIKDCNAIP---------
---TIGYFPDSEGDAENRA----------
----------------DNQEIEQSSQ---
--------NKVHLEES--------------------------IDN---------CTPV-EQ------------
-TVEIHISVS-------------EFAVTGSK--
----------------RRRER-PSKSNTKRNRNSRNAYSQDTR------------------------------
--DI--ICDEATVMDGIK------NNSDAKDMTHCEELHQEITLTSPL--DKACK------------------
---GTDERVHAEEETL----ADVGVVHFQKI-F---------SSGSAENMPFSSGDSHINNDVIRV-
GKDVNKSKT---KRE------------
-------------------------GKGK-----------------------------SRTG--------
DTNSICDVSKGLVCNDITMK---------------------------DGL---
KLNPDDINEIENGDLNPEAKLVQSTGTLNIEDC---------NANSTIGRF------------PDSE------
-----------GDADPRADNQEI----------------
EQSSQNKVHLEKLIDNCTPVEQTVEIQIPISEFAVTGSKRRRGRSS-------KSNTKRNINSRNAYSQES--
--------RDILC------------------D------------E----ATV---------------
MDGIKNNSDVKDMTHCEELHQEITPISPLD-K---------------------------------A---
CKGTDKRVHAEEENLADVG----------L-VHFQKNVSSGSAENMPFSSGDSHI------------
NNDVIRVRKDVNKSKTKRKGKGKSRTVDANPICD----VSKGLVCNEMTMKDGLKL---NLDDINEI-
ANGDPNPVSGEIEHDMSAE-----------------------HDTQIGDNFLKE-------------------
----------N--------------VDKNKDS-----------------------------
LQTQKNDTERTMG---TPI----------------
----------GKRK---NLHLKIPPPLP-
------PRRVQ----------TTPEEVSK----------------------AFGLKTSR-SG-----------
------------------------------EI---
----SGTCLAILN-----------YM---

>Picea_glauca00028859

----MEDPF-AF---------------QAENSAHSGVTCG------------CSD-EAEYCTQND--------
----------------RRFHNGFPG-----NSIQRPYSSPF-------------RTLF--------------
NSPVHHR--NQL---

--FRRSTR-----------
---GSNLA-----------------------
-------------------------------T----------------GGS-----------DYVH-------
------------SVSSQKR--
--VNLYKWYLIR-VVKDCPQND-MINDE-

AQVAVG---------------GYT-------------------------------T------------PD---
LL---------------------------------------DC-EKF--------------------------
----
RTAPIESRIEKCKLRTVDGLDVLLMGTI-------DELCTNDN-GFPFQ--------VIHL-FLHGF--
PFNW-------------N-HV--

--------------INI---------------------------------SNGKP------------------
-----SLNMHI---
LPSSKMKVCASKE----------K---------------------------LEKPDSECPVVQVDDT------
------------VTDKTSLLEPEQ-----------------SLYKAGSPR-----------------------
-------------ESSVGCSKRVRHK--------------------PQPA-----------------------
----

>Picea_sitchensis00004746

----MEDPF-AF---------------QAENSAHSGVTCG------------CSD-EAEYCTQND--------
----------------RRFHNGFPG-----NSIQRPYSSPF-------------RTLF--------------
NSPVHHR--NQL---

--FRRSTR-----------
---GSNLA-----------------------
-------------------------------T----------------GGS-----------DYVH-------
------------SVSSQKR--
--VNLYKWYLIR-VVKDCPQND-MINDE-
AQVAVG---------------GYT-------------------------------T------------PD---
LL---------------------------------------DC-EKF--------------------------
----
RTAPIESRIEKCKLRTVDGLDVLLMGTI-------DELCTNDN-GFPFQ--------VIHL-FLHGF--
PFNW-------------N-HV--

--------------INI---------------------------------SNGKS------------------
-----SLNMHI---
LPSSKMKVCAKR---


```
-------------------------------------------------------------------------
-------------------------------------------------------------------------
-------------------------------------------------------------------------
-------------------------------------------------------------------------
-------------------------------------------------------------------------
-------------------------------------------------------------------------
-------------------------------------------------------------------------
-------------------------------------------------------------------------
-------------------------------------------------------------------------
------
>Pinus_pinaster00061713
-------------------------------------------------------------------------
-------------------------------------------------------------------------
-------------------------------------------------------------------------
----MEDPF-AF---------------QSENSAHSGITCG------------CSD-EAEYCTYND--------
----------------RRIHNGFTR-----SSIQRPYTSSF-------------RNLF--------------
NSPVRHR------------------------------------------------------------NQF---
-------------------------------------------------------------------------
--------------------------------------------------------FHGSTR-----------
---------------------------------------------DPKPV-----------------------
-------------------------------T----------------GGS-----------DYVH-------
------------SASSQKQ------------------------------------------------------
----------------------------------------VTLYKWYLIR-VVKDCPQSD-IIDDE-
EQVAVG---------------GYT-------------------------------T------------PD---
LL---------------------------------------DC-EKF--------------------------
---------------------------------------------
RTAPIESRLEKCKLRTVDGLDVLLMGTI-------DEQCTNDN-GFPFQ--------VIHL-FLHGF--
PFNW-------------N-HF----------------------------------------------------
-------------------------------------------------------------------------
-------------------------------------------------------------------------
--------------ISI---------------------------------SNRKP------------------
-----SLNMHI-------------------------------------------------------------
LPASNTEDCASKE----------K---------------------------VEKPDSECPVVQVDGI------
------------ETGKSSLLEQEQ-----------------SLSRTDSPR-----------------------
-------------ESSLRRSKRVRHD--------------------SQPAKLSKKS-----------------
--ICCKLSTEDKPEK--------------------------IFTVV-------ESQEKSNILKESE-------
----------------------------------------------ANLS-----SGAPTKSL----------
-------------------------------------------------------------------------
-------------------------------------------------------------------------
-------------------------------------------------------------------------
-------------------------------------------------------------------------
-------------------------------------------------------------------------
-------------------------------------------------------------------------
-------------------------------------------------------------------------
-------------------------------------------------------------------------
-------------------------------------------------------------------------
-------------------------------------------------------------------------
-------------------------------------------------------------------------
----------------------NFKSSDM--------------------------------------------
----------------------------------------------------DSHV---------
QIVQEDKL-------------------------------------------------TAS-------------
------------------------GK-----------------------------------------------
-------------------------------------------------------------------------
-------------------------------------------------------------------------
----------------------------------------------EF-------------------------
-------VGSQDN----------PNIL----------------------------------------------
-------------------------------------------------------------------------
------KGTEA--------------------------------------------------------------
-------------------------------------------------------------------------
-------------------------------------------------------------------------
```


>Pinus_sylvestris00003294

----MEDPF-AF---------------QSENSAHSGVTCG------------CSD-EAEYCTYND--------
----------------RRIHNGFTR-----SSIQRPYTSSF-------------RNLF--------------
NSPVRHR--NQF---

--FHGSTR-----------
---DPKLV-----------------------
-------------------------------T----------------GGS-----------DYVH-------
------------SASSQKQ--
--VYLYKWFLIR-VVKDCPENG-TIGDG-
EQVAVG---------------GYT-------------------------------A------------SD---
LL---------------------------------------DV-DRY--------------------------
----
ITSPIESRIERCKLRTVDGLDILLMGTI-------DDQCTYEN-GFPFQ--------VIHL-FLHGF--
PFNW-------------N-HI--

--------------VNI---------------------------------CKGQP------------------
-----NSNIAP---
LLARKTEESVSK---ECPVVQVDDT------
------------GAGKLGSLEPEE-----------------YFSRVVSPR-----------------------
-------------ESSVRSTKRVRHD--------------------SQLYRSSKES-----------------
--KCGKLSTESKPKM--------------------------HFTGV-------GSQENSNILKESE-------
--ANLC-----LGASSKSIEKE-------

--GLMPHL-----
--------RSSDMDIHG-------QINHKDKLTD-----------LRSELGKTKPLNFSVSHIV------
KEPVQN-DN-------KIMKG--L---

-------------------------LFTKN---

>Pinus_taeda00042871
---MNISSQR-------------

SEQDSDTENLSEMGNPFRVF---------------QSSNPVNSAGACG------------CLR-
QTECCTPKG------------------------RQFHS----------------------------------
NRF--------------NSPAHSR---
-----------NQF---

SLESTP--CSKLV------
--T----------------GSG-----
------YGIH-------------------STCSQKQ-------------------------------------
---VYLYKWFLIR-
VVKDCPENC-TIGDG-EQVAVG---------------GYT-------------------------------A-
-----------SD---LL---------------------------------------DV-DRY----------
----
ITSPIESRIEKCKLRTVDGLEILLMGTI-------DDQCTYEN-GFPFQ--------VIHL-FMHGF--
PFNW-------------N-HI--

--------------VNI---------------------------------CKGQP------------------
-----NSNIAP---
LLARKTDESVSK---ECPVVQVDDT------
------------GAGKLGSLEPEE-----------------YFSRVVSPR-----------------------
-------------ESSVRSTKRVRCD--------------------SQLYRSSKES-----------------
--KYGKLSTESKPKM--------------------------HFTGV-------GSQENSNILKESE-------
--ANLC-----LGASSKSIEKE-------

```
--------------------------------------------------------------GLMPHP-----
--------RLSDMDIHG-------QINHKDKLTD-----------LSSELGKTKPLNFSVSHIV------
KKPVQN-DN-------KIMKG--YNLRRTRGTE----------------------------------------
----ELKKQMDSGT----------------------------SLDEAKFI--IEN------------------
------------------------------------------------------MKFEDTNE-N---------
-----------------------------------------------TSARFTNSEDYADCRL----------
----------------HNQEVEQSPL-----------------------------------------------
--------TNEHLEQS--------------------------SNI---------CSRLGEQ------------
-TQEIQTPIS-------------ECA-----------------------------------------------
------------------------KLNTKKT-SSKNVFSQASR------------------------------
--NL--LCDDGTVKDGIE------SNSDGKDTTYSKKSHQKIIPTRPL--
HEVSKIYSQFPLDPDFLEFGSKKTRTGTDGKAQAEEDSL----ADVGFVQLQKN-V---------
SFHSAESKSLLPESSHN-NKIIGS-EKDVNKSKT---------------------------------------
----------KRK-------------------------------------GRGK-------------------
----------RRTTI------RHTNPFPAVVEGLVCGE-KMK---------------------------DDV-
--NLKFDDIDATSNGDPNLVACEIDN-DTLAEHDTEIGDNCLKENVDGDKGRLQTRKHGTLENTGTDGK----
-------------AQAEEDVNKSKT----------------KRKGRGKRR--------TTIRHT-------
NPFPAVLEGLVCGEKM-------KDDVNLKFDDIDATSNGD----------PNLVDCE---------------
ID------------N----DTLAEHDTEIGDNCLKENVDGDKGRLQTRKHGTLEN------------------
----------------------------------TGTDGKAQAEEDNLTDVG----------F-
VQLQKNVSFHSGQTKPFLPGNSHI------------NNNVMGSEKDVNTSKTKRQGR----------------
-VLEGFSWGE-KMRDDVNL---KFDDIDAT-SNGDPNLVACEIDHDMLGE-----------------------
HDTVTGDNCLKK-----------------------------N--------------VDRDKGS----------
-------------------LQTQKHGTLRTVG-----------------------------------------
SPT--------------------------GKKQ----------------------------------------
-------YSHARIPPPL--------PRRVQ----------TTPEEVSK----------------------
AFGLKTSR-----------------------------------------------------------------
-------------------------------------------------------------------------
-------------------------------------------------------------------------
-------------------------------------------------------------------------
-------------------------------------------------------------------------
-------------------------------------------------------------------------
-------------------------------------------------------------------------
-------------------------------------------------------------------------
-------------------------------------------------------------------------
-------------------------------------------------------------------------
-------------------------------------------------------------------------
-------------------------------------------------------------------------
-------------------------------------------------------------------------
-------------------------------------------------------------------------
-------------------------------------------------------------------------
-------------------------------------------------------------------------
------------------------------------------------------
>Pseudotsuga_menziesii00073202
-----------------------------------------------------MNISSQW-------------
-------------------------------------------------------------------------
-----------------------------------------------------------------
NEQISDPENLS-MGDPFGVF---------------QSSNSVNSRVTCG------------CLR-
QTDCCTPKG------------------------IPFH----------------------------------
RNRF--------------NSPMHHG------------------------------------------------
------------NEF----------------------------------------------------------
-------------------------------------------------------------------------
-SPGSTP--------------------------------------------------------CSKLA-----
-------------------------------------------------T----------------CSS----
-------DVIH-------------------STSSQKQ------------------------------------
----------------------------------------------------------AYLFKWFLIR-
VVKDCPENG-IIDDG-VQIAVG---------------GYR-------------------------------A-
-----------SD---LL---------------------------------------DS-HKF----------
-------------------------------------------------------------
RTAPIESRTEKCKLRTVDGLDVLLMGTI-------DEQCTYDN-GFPFQ--------VIHL-FMHGF--
```

PFNW-------------N-HI--

--------------VNI---------------------------------CKERP------------------
-----SSNLAP---
LPVSKIEENVCK---ECPVVQVDDT------
------------ETGKLSSLEPEE-----------------NISKVVSKR-----------------------
-------------ESSVSCSKRVRCE--------------------SQISRSSKGS-----------------
--KRGKLSTESKPKM--------------------------HYTCV-------GSQENYNISKESE-------
--ANMC-----LGASNKSIEKE-------

--RSITRI-----
--------RSSDKDIHV-------QINQQDKLTV-----------LGSELGRKKPLNFSVSHTV------
KRPVEN-EK-------KIMEG--YHLRRAKTTE--
----EFKKQIDSGT----------------------------SLDEAKLI--TAT------------------
--MNIEDASENI---------
---MSDRFTESAADAHHRL----------
----------------DIPEVEQSVM---
--------SNEHLKQS--------------------------KDI---------CSPLEEQ------------
-TQVIEIPFS-------------EFADSCYK--
----------------MRRGRISGKSNTKTN-RSRNAFSQASS------------------------------
--DL--LCDDATVKDAIE------NNSEGKDMTYSKELHQEIIPTRPL--
HEVSKVHSQFPLDSGFLEFGRKKTRIGTIGKAHTEEDNS----ADVGFIELQKN-V---------
SFHSAQTKSFPPGNSHINNNVTRS-EKDVNKSKT---------------------------------------
----------KRQ-------------------------------------GRGK-------------------
----------RRAT-------RHT--MPVVLEGLVCSEKMVK---------------------------DNV-
--NLKLDDIDAT---

---------------------NGDPHLVGCEVDNDMPAE-----------------------
HETEIGDNCQKE-----------------------------N--------------IDRDKGS----------
-------------------LQTEKHSTLKTAD---
TPT--------------------------SKRQ--
-------HSHANIPPPL--------PRRVQ----------ITPEEVSK----------------------
AFGLKTSR-SG----------------RLLVPPLAH-W-------CNQKIAYDL-------------------
--------------------------DGGIIAIFD-----------GISEQKEDNGGYN--------------
------------FKPPTEA----------------------------EAKKMQRKL---CKAANDVIKT----
--------------------------------------PKSGKK-----------------------------
----

-----------------------------------IRKG----------------------------------
----

>Salvinia_cucullata0025.g009355
---MHNHHHP---------------

----LAQPPSISPLPLYHGSPAPDRCPASADRPFPERISPGCGCN-L-HH-----LHASSGSPHFL-------
----------------RTMETA--------HACCQPGTSEL-------------------------------

```
RRPSSAEESRSV--FRDP----------------CRSSELS--------------------------RKFS--
-------------------------------------------------------------------------
------------------------------VAECLFPSHN--------------LSENAYVPR----------
---------------------------------------------TPQIND----------FGATPVMQ----
----------------YEPTSTAQFF-SWAYKQNN--------------------------FGMVSE------
--------PGHCMMRQQNQ------------------------------------------------------
----------------------------------------VKLYGWYLIN-SSNNT-----------
AEVAVG---------------GSL-------------------------------T------------PG---
-----------------------------------------AV------------------------------
----------------------------------------
SNGRKETTTITKRLEKCRLLTKDGVEVRLMGLI-------DDSTTISN-GFS----------IVHL-FLSGF-
-PYTW-------------ERLL---------------------------------------------------
--------ENEFRMYKTKITAGPGPGP---------------------------------SQVLEN-------
-------------------------------------------------------------------------
---------------VNLR----------DSEISKLSAEHV----------ENSRK-----------------
NASSLNGNQVDKFILDGQTMDVTQCKQNSSET--------MP-
DVKLQSIAGDEQGRVNVGDIGMPMNIMEKTQFTEESHEIIHSA----------QATKDKPD---GMPF--L--
--------------------QDDTSGLQ-------------KETEKLKELDSPV----------------
LRMEHMRKSIDS------------------LEKT------------DQMMRASRRIT----------------
------RSMQRSSKHM-------------------GATENET-------------------------------
--INGV-------DKFPQLSVSQEPE------------------------------------------I----
------ENLTKELRGINASSESITDTF--LKQRGIHSMRSTRK------------------------------
--------------------------PVKD-------------------------------------------
---------------------NRCRSST-------------KESSADDPI-----NIKCSDNKEMEEV-----
----VIVDDIEVEETALES-----------KQEAES--------LTETIEK---------NCSQR--------
------------------------------IEEEGEIESQNTNGVG--------------------------
TFPQLGVSHESEAE-----------------------------------------------------
SLTKEQNG-------------ITTSPES---------------------------------------------
-----------TTDTSLKATD------------------------VHLMRSIRKPVKINRHQSCTK-------
-------------------------------------------------------------------------
------ES----------------------------SVDDPINIKCS----------------------
DNKGMEEKLVTVDDIE-----------------------------------------VEQTAVESKQNAENL-
------ARTIAKT----------------------------------------------------
CSQRIEERAVQDPRNV---------------------------------------------------------
-------------------------------------------ESFARHDADM--------------------
----------------------------EEK-------------------------------------TESA-
-------------------------------------------------------------------------
--------------------SIES-------------------------------------------------
-------------------------------------------------------------------------
-------------------------------------------------------------------------
-------------------------------------------------------------------------
----------L--------------------------------------------------------------
-------------------------------------------------------------------------
-------------------------------------------------------------------------
------------------------------------------------------------LSDVGAMNTTI--
-------------------------------------------------------------------------
----------------------------------GNEFREGKET-------------H---------------
-------------TSCRAGM-----------IPPPLPCPR-----VPVSKRLIST------------------
----AFGLKTSR-SG----------------RLLVPPLAH-W-------CSQSLIRDK---------------
------------------------------DGGIIAISDCSMNTSAL-DDGS---------------------
--------------LKFKPPAGANLQ-----KLQKRL-R----------EAAVEGLKTKT-------------
------------------------------------------TNKKKKST-----------------------
-------------------------------------------------------------------------
-------------------------------------------------------------------------
-------------------------------------------------------------------------
-------------------------------------------------------------------------
-------------------------------------------------------------------------
-------------------------------------------------------------------------
-------------------------------------------------------------------------
-------------------------------------------------------------------------
-------------------------------------------------------------------------
```

```
--------------------------------------------------------------------------------
--------------------------------------------------------------------------------
--------------------------------------------------------------------------------
--------------------------------------------------------------------------------
----------------------------------------------------------------
>Salvinia_cucullata0183.g024952
--------------------------------------------------------------------------------
--------------------------------------------------------------------------------
--------------------------------------------------------------------------------
----MSQ-------------------------------------------------------NSHSEGSLLA------
-----------------RSFQMG---A--------------------------------------------------
DTELQ-RHPH---------------------------RVS----------YQ--------------------SRVQ--
--------------------------------------------------------------------------------
------------------------------------------------------------------YPE----------
-------------------------------------------------------------------EQI---------
-------------------------------------------------------------------VTHIGV------
-----------------HQM-----------------------------------------------------------
--------------------------------------------R----WYLTR--------PV-GC-QT-
SDIGVG----------------GFL----------------------------------S------------------
-----------------------------------------------TRAV----------------------------
--------------------------------------------
TSERVETAAIIKRLEERKLLTQSGVVINLIGTI-------DDCATLTN-GFSIQ--------MVHL-FLSGF-
-PHVWSLLV----------------------------------------------------------------------
---------------GTMSGSVSIEKNS--------------------------------------------------
--------------------------------------------------------------------------------
--------------------------------------------------------NTSGKVEE---------------
------CQSL-----------FFDSKEESPSNFHAVG---------------------------------------
---NRDQVDAEHGPHL-------DGS---------------------------------------------------
--------------------------------------------------------------------------------
--------------------------------------------------KNMHFT-----------------------
---------------------------------------------------------------STTRKFPE-------
--------------------------------------------------------------------------------
------------------------------------------------------------------I---I--------
--------------------------------------------------------------------------------
------------------------------ETHLEPC-----------EKRVLDDDA----------------
KCSIEK---------KGGV----DVADMTTGTG-----IHTETLD------------------------------
----------DAKL------------------------------SQGEVLATTARRKM------------------
---------------------------------------EVAVNVVN-------------------------------
--------------------------------------------------------------------------------
--------------------------------------------------------------------------------
--------------------------------------------------------------------------------
--------------------------------------GKNDN------------------------------------
---------------------GKDQLALLEN-IMEHNE-KN------------------------------------
-ND----------------------ENESGNDQEAPSSLNE------------------------------------
-----------------------------------------------------------
CVGTDQKSILDDEIQQIDKAEYGGAET------------------------------------------------
SVS----------------------------------------SEEN-------------------------------
--------------------------------------------------------------------------------
--------------------------------------------------------------------------------
--------------------------------------------------------------------------------
--------------------------------------------------------------------------------
--------------------------------------------------------------------------------
--------------------------------------------------------------------------------
-----------------------EDKESNENSEI--------------------------------------------
---------------------ECDLKARME------------------------------------------------
------------------------------------SCISRNSGST---------------------------------
---------IN---------------------------------------------SPE-------------------
-----DACSVRD----------------------------------------------------
PPTDASTKKMMKHI--LPPPLPSPT-----VSVSNQLVSK-----------------------AFGLKTSR-
SG-----------------RILVPPLAH-W-------RNQSIIRDS------------------------------
```

-----------------DGGIIAIQD----------GSEDTMR-------------------------
VDTGIFNFKPPAEARAR-----KLQKWL-S---
VTAADCFTKRLKSLHARSVKPGGPAEPHVGPSSVFSRHLRAMGISLSALPLQPLTKPPLSSPLLSNKDTLSPA
SAKKRQRGIDSSTDDGTPSPSKSCRRSPHTPEIQERPSRLRPRKLGFDASVQQTDVQSSLPSIAPSENDAIKQ
IAEKSSGQFSVLNQNSALSPSQHTDKGTAILRTPTRRASPRLLEKLSTKDAATSVVKPRSLNFSPVTPSRKSV
DKRLPPPSPNVTKTKVPTTPKRNFLDHVTVDRVKFSVGDDVYVKRTDDDIDEEAEGCLICGKAGKLIECDNCS
RGVHLKCTDPPLKKVPDGEWLCPKCELPSKASDSKQNGFQKNGTEALKTARELLLSCKLWAARIERIWKNEDG
SLWFQGRWYLIPEETSIGRQPHNLRRELFLTNDIDENEVVSILRKCYVMGPEEFRNSGRDGDDVFLCEYEYDT
QFHTFKRISDMDADNHSENDLSEEDEDEDSDGEESLRYGKKSHSQTPPKAANSRGSLLKIGTKSIPHNARIKP
ATVFERAKAALRLTATPGSLPCREREMTEISSFVTDAVVSGNKGLGHCLYISGVPGTGKTATVMEVMQKLKRR
YEEQNANPYRFVKINGLRLTSPEHLYTVLYEALTGHHVGWKKALQLLDERFSNPNPSRRADARPCILFVDELD
LLVTRNQSVLYNIFDWPTRPHSRLFVIGIANTIDLPERLLPRIASRMGLQRLSFSPYSHEQLQTVISSRLEAI
NAFEKQAVEFASRKVAAVSGDARRALELCRRAVEVAESRCTGDSNRGNSAVKGNLVSIKDVEEAIKEMFQAPH
IKMMGRCPKQAKIFLVSMVYEQHKTGMAETIFEKVASTYTFLCRNNNEMSSDWDTLLSVGCALGACRLILCEP
GSYHRIQKLQLNFPTDDVSFALKQDPEIPWIGKAREYFFLLVVFLELPPAAMVVAKKTKKAQESINNRLALVM
KSGKYTLGYKTTLESLRSGKGKLVIISNNCPPLRKSEIEYYAMLSKTGVHHYTGNNVDLGTACGKYFRVCCLS
ITDPGDSDIIRTMPTE
>Aegilops_tauschii2Gv20621700.1

----MAKK--TPSPPP--------
----------------RARSRR--------GAA----PT---------------------------------
----

--SPTPA-----------
--------------------------AA--------------LSPPFS------------------------
---------------PAPLRT-----------------------RLGAA-----------AVAAAA------
----AAAAAASSSPVEHPC---
--------------------------------------VTLCEWWPVR-VEGEE-----------RKLAVS-
--------------GFT------------------------------E------------RN----------
------------------------------------DAF---------------------------------
-------------------------------------TSAPIAHRYEPLTLQDEGGVVVLLHGSI-------
NLLRMREN-GFSVQ--------ICEQ-FMIGF--PFWW------------ETW-------------------
--DSHMESYPNCFIDPREGSA-------------
--------------------QFYLEK---
FQLGNFIQ--
KFGPSFIEDLL-----------NNAKNFPI--------------------DHLDAFT-------
ESSRFQEYICG--NDASTKENSAASD--------
--DAR---------------------------PATVA----NVEIGL-------------------------
----
TASSISQERDHVDIE-----------------CNVSLAPAET-------------------YTGDET-----
----

---CKEA---------------
GNQNDT----------MHPDAREED-----------AGSHLFNSDW--------------TCTMCP------
----DHMPN--DSEG---GNEN--
SVEL--------------------------LAKYPLAIVPPENA----------------------------
-------------------------NCCSEIPG----ASQSVEPSSM-------------------------
----

>Aegilops_tauschii2Gv20622700.1
---M-----------------------

RTRSMASKP--EPVPSTHG------
------------------------------TAARAPAPASV-------------------------------
SASTHGTAARAA-----ARASVSASTHGKAARAPARASVSAST----HGT------------
AARAPAPASVS--
--ASTHG--------------
TAARAPAPA------------------------------------SVSASTHGTAARAPAPVSVSAPTHGT--
-----------------------------------AAPAPPPASV----------------------------
-------RAPTYC-------------------ATVQRC-----------------------------------
---VALLDWWLVR----
---------GQG-GKIRVA---------------GYI---------------------------------
DNV---------E---KNRAG-RVF----------

SSGSITVRHADGTLETADNKIVLTRGPL-------NIEQMHWN-GFSRE--------VSEQ-FRLGF--
PIQW-------------EKYA--
-------NSNMKQA-NEHILSPAKST---------------------------------EYCVEK--------
--------------------------------------FLRSSF-----------------------------
--ANSMEHTLT--------------
-----GFDF--
--RTSKESTGNT----------DGPGLPNYVK---PR-----------IQEPSGNSVGYDNSVSNM-------
------------AASEGLCNDRM---------------GTPDESFED--------------------------
----PG----PGETCNGQASRADNSH-------------------EDIQTDASGQ------------------
----

------------RIVTH--
--------------------------------------SADSTLVNNDIDKI---------------------
----

```
-------------------------------------------------------------------------
-------------------------------------------------------------------------
--------------------------------------EE---------------------------------
-----------------------------ERGSSKLGNSS-----------VCPG------------------
-----------TEHVSEALNQGASPEHGSVQCS----------------------------------------
-------------------------------------------------------------------------
----------------------------------------------------------------
RRLRSGKVY------------------------------------------------GM--------------
-------------------------SNGA--------------------------------------------
-------------------------------------------------SLKR--------------------
-------------------------------------------------------------------------
-------------------------------------------------------------------------
-------------------------------------------------------------------------
-----------------------------------------------------------------------R-
-------------------------------------------------------------------------
-------------YSKRK-------------------------------------------------------
----TMQHGTLSMK-----------------------------------------------------------
-------------------VIPTEETT----------------------------------------------
------------------------------------PPAGPT------------------------CHK-
KVG----------------------------------------------------------------------
-------------------------------------------------------------------------
-------------------------------------------------------------------------
-------------------------------------------------------------------------
-------------------------------------------------------------------------
-------------------------------------------------------------------------
-------------------------------------------------------------------------
-------------------------------------------------------------------------
-------------------------------------------------------------------------
-------------------------------------------------------------------------
-------------------------------------------------------------------------
-------------------------------------------------------------------------
-------------------------------------------------------------------------
-------------------------------------------------------------------------
-------------------------------------------------------------------------
-------------------------------------------------------------------------
-------------------------------------------------------------------------
-------------------------------------------------------------------------
-----------------
>Amaranthus_hypochondriacus008009-RA
-------------------------------------------------------------------------
-------------------------------------------------------------------------
-------------------------------------------------------------------------
-------------------------------------------------------------------------
-------------------------------------------------------------------------
-------------------------------------------------------------------------
-------------------------------------------------------------------------
-------------------------------------------------------------------------
-------------------------------------------------------------------------
-------------------------------------------------------------------------
-------------------------------------------------------------------------
----------------------------------------------MVKISWSDAYRC----NDAQRKFHSA-
--------------PAR--------------------------------------------------------
-------------------------------RRYAS-REF---------------------------------
--------------------------------------HSTKIVKRHGCTTLETSDGFIVSLCGFI-------
NKSRSLEN-GYSTE--------VCRD-FIYGF--PYNW-------------EHYA-----------------
ASVP---------------------------------------------------------------------
-------------------------------------------------------------------------
-------------------------------------------------------------------------
------------EESSQ------------------------VNL----------------TDSTKC---F---
-------------------------------------------------------------------------
```

------------PSADLDAYNGAQLHEL---
---SFSLSDPERE----------------

-------------------------L---

------FCNKVYD----------------------------------DLM-----------------------
----
ELSSLI---

DE---
---NE--

-------------------------AQPSKPTRS---------------------------------------
----

------------------------------QVDVDV-------------------------------------
---LTSESRN---------------------

TKV--FTFQAGK-CE-
CI------------TEVL---LEH---
------E-------DS---

>Amborella_trichopoda_00137.13

----MGLT--ALSSPKF--------

--F----

---------------EKK---

```
--------P-----------------------------VVLKNWYLMK-VPSE--------TSG-KRLAVG-
--------------GFH------------------------------S------------K-----------
------------------------------------THF---------------------------------
-------------------------------------HSCPIEERISAFRLRTSDGAILLLEGCI-------
DEQWTLEN-GFPFE--------ISGL-FRIGF--PYTW------------KILV------------------
----------------------------------------SQFLSEDSSGFSIREVET--------------
------------------------------------------------------------------------
------------------------------------------------------------------------
-----------NNGSSTFLP-------------ITHLFGRNLFP--------------SETERF--------
--------------------------------HKQNEEERKACES----------ERQTT------------
------------------------------------------------------------------------
---------------------------------------------KSKSSSVARI-----------------
------QQYEVCLGQA--------------------------------------------------------
------------------------------------------------------------------------
------------------------------------------------------------------------
------------------------------------------------------------------------
----------------------------------------NVNVND------------SSSSKVR-------
-----------------------------FGSINE----------ETISK--GIDNEQPKCAKG--------
----------------------------FGSLYSNS-----------------------------------
KVDVVITPEGVKYH----------------------------------------------------------
-GTK-------LSDWDVPSPSGV-----------------------SL-----------KVL----------
-----ESNKKASQV--------------------------------AGSKVRRKRTAAIL------------
------------------------------------------------------------------------
DP-------ELNC-----------------------------------------------------------
-----------------------------------------------------SVNLVKKS-----------
--------R---------------------------------------------
TSSSKELKALQTPLSREFKTL---------------------------------------------------
------------------------------------------YSPSKELKALRIPV----------------
---------------------------SKE-------------------------------------FK---
------------------------------------------------------------------------
--------------------TLHF------------------------------------------------
------------------------------------------------------------------------
------------------------------------------------------------------------
------------------------------------------------------------------------
------------------------------------------------------------------------
------------------------------------------------------------------------
------------------------------------------------------------------------
--------------------------------------------------------------PSKESKAL--
------------------------------------------------------------------------
------RIPV--------------------------SKELNVL-----------------------------
-------------QTPSKGL-----------------------TALQTPSKESK------------------
-------LSISR-SG---------------RIIVRPLAY-W-------CNERIVYGK---------------
-----------------------------DGSITSILDG-------------DKYD----------------
--------EQCTRGSKSAPPKRRHTR------------------------NQNSSKSKQ-------------
---------------------------------------GERPSERKLTRNE--------------------
------------------------------------------------------------------------
------------------------------------------------------------------------
------------------------------------------------------------------------
------------------------------------------------------------------------
------------------------------------------------------------------------
-----------------KPSKPKGSPM---------------------------------------------
------------------------------------KLRRR-------------------------------
------------------------------------------------------------------------
------------------------------------------------------------------------
------------------------------------------------------------------------
------------------------------------------------------------------------
------------------------------------------------------------------------
---------------------------------------------------------
```
>Ananas_comosus021414.1

---MGANASPR--------------

RSQRGEEEELVAS--PTP-----
---------------------SPSP----------TPTPTPAPKSP---------------------------
----
TPF--
--SPS----
---LPPPPK----------
RSFPTTTRFP----------------FSPSPVAVSS------------------------AAA----------
-AAADA------------------SSTDKAC--
--VSLFDWWLLG-VERE------
--CGG-KKLAVG---------------GFT-------------------------------T-----------
--R---KQ-AT-RIF--------------------

TSAPIVKRHDAYTLETEDGIIVIIQGMI-------NRERMQSN-GFPPE--------VSKC-FLIGF--
PYNW-------------DQYA--
-------N-EYLQKNPASTNSLGSFFSVSE--QHKDSIKGG--------------------------------
----
-----------------------------DFGAKITRNFI----------DSIKNLFQLTSV-DSIVQR----
-SQC---
--------------------------L--------------------------PNEAETH-------------
----
--------------KYKDANK-------------NVDLMPHSVEGN---LSAKT-------------------
-IESSEIS-----------------------------------GM-------KSVL-----------------
---ISSSLNV-------
EDVIP--------------------GEQ---
---------NH--SGN-----
----------LRTDD------------R---------------------NVLDNTSVQKGATII---------
----
---DAGL--------------------
--LKVRMNVART-----------
-----LLDS-CG---------------------------------STSRKI----------------------
-----------------------------------STRKAGARTKS---------------------------
------QVSSLNR---EVEPSDLSQ---------------EN----------------------------
TGSYCVK-----------------------------G------------------------------------
-----------------------ESTSLINVE-AINSE-AP----------------------------
VVEKRSKDEVLS-----------------TPRKRKI-------------------------------------
--------------------------------KQ----------------------PTSSVIHLEASP----
VEKKTKEKVL--RTT------------

----------------KR---
-----TTKTP---
--------------------TSQVYQA--
--------------------------------------PI---------------------------TRQ-
HA---KQLSF---------------------
------ASPESLN--------------------------LRRTR-SG----------------RIIVPPLAK-
--------GSQQIIYDA---VCEIFSLL---


```
------------------------------------------------------------------------
------------------------------------------------------------------------
------------------------------------------------------------------------
------------------------------------------------------------------------
------------------------------------------------------------------------
------------------------------------------------------------------------
------------------------------------------------------------------------
------------------------------------------------------------------------
------------------------------------------------------------------------
-----------------
>Asparagus_officinalis_02.1557
------------------------------------------------------------------------
------------------------------------------------------------------------
------------------------------------------------------------------------
----MSQK---------------------------------------------------ANPSTQPPQVP--
---------------SVSSP----------ATAPPPSGS---------------------------------
-----------------------------------------------------------------TPF----
------------------------------------------------------------------------
----------------------------------------------------------SP------------
------------------------------------------------------------------------
------------------------------------------------------------------------
-----------SSSFSKC------------------------------------------------------
--------------------------------------VLLLEWWLLK-SDTD--------SGG-KRLAVG-
--------------GLN------------------------------A------Y-----------------
---------------------G---------KQ-VT-RVF--------------------------------
-------------------------------------TSAPIVTRFDAYTVETADGITVRIQGFI-------
NKARTHLN-GVPPE--------VSQC-FLIGF--PYNW------------DKYA------------------
----------------------------------------NGDFWEKPDSPAVSMRSSG-------------
-------------------ISRQLEQ-----------GS---------------------------------
------------------------------------------------------------------------
-----------SDISAVERD------------------SSNLLPI-------------EREDPH--------
------------------------------------------------------------------------
------------------------------------------HKLERGLGGY--------------------
---------------------------------------------ELRSRDRSRKTGM--------------
------KNTVSSESSI--------------------------------------------------------
----GR------------------------------------------------------------------
------KDC------DEGDANSLKMKRS--------------------------------------------
------------------------------------------------------------------------
---------------------MAFEPSAK--------APNERGNSRDSRL-------VIQNECRSVTRK---
---HTIHSGIVKHCGIK------------HANSVAA---------DVLQE----------------------
---------------------------------------------------------GHP------------
-----------------------------------------------------------------
HFSPEEPG----------SEHQQETPVSRD--------------VINKKCAG--------------------
---------HSEHDDSLRKRS-------------------------------NSVSVKTVIQCSV-------
----------------------------------------------------KRTNHSGSTAQLHL------
------DR--------------------------------------------------------
ASDLTRKSTGELPGI---------------------------------------------
CDSGALTDEELTRRK---------------------------------------------------------
------------------------------------------------------------------------
--------------------------------------------------KEHASTRKTI------------
---------------------W--------------KPS---------------------------Y-----
------------------------------------------------------------------------
----------------------------SLES----------------------------------------
------------------------------------------------------------------------
------------------------------------------------------------------------
------------------------------------------------------------------------
------------------------------------------------------------------------
------------------------------------------------------------------------
------------------------------------------------------------------------
------------------------------------------------------------------------
```

----------------PL---------------------------TRD-KA----------------------
-----------------------KKLSV---------------------------TSPELLDL----------
----------------KRSR-SG----------------RLLVPALAR-W--------CQKIIYDT-------
--------------------------------------DGSITGLSGL-------------DELN--------
-------------------------AP-CKG------------------------------SRSE--------
--QPKRRK-----------------

--------------------------------PL---------------------------------------
----

>Brachypodium_distachyon_3g30800.1.p

----MAPEP--QPGPSRHG------
------------------------------TTIQAPPPASV-------------------------------
D--

--APIYR------
-------------ATIQKC--
--VALLDWWLVR-------------GQD-
DKIRVA---------------GYT---E---
--------------------------------------RNRAA-RVF--------------------------
----
TSDFITMGHADGTLETADHKIVLTRGPL-------NIKQMHRN-GFPYE--------VSKH-FQLGF--
PAQW-------------EKYA--
-------NSNMKQS-KHQAQSPSKSI---------------------------------DNI-----------
---WKF-----------------------------
--LSFMKYNFE--------------
-----ETDF--
--NSSKGSTGNT----------DGTPI--
---------------QGLSNLSN---------------GTP--RFQDSSG-----------------------
----PG----PGETCS--SAQGDNQH-------------------QDMHLDA---------------------
----

------------EDAGQVPAKGMSPEFGVVQGS-------------------------------------
EDST---

GRRLRSGKVY--GMS------------
-------------------------TSSSA---
--SLKR-------------------

G--
---------------RSKRK---
------TIQHDTPNRK---
----------------------ILNEETI--
--------------------------------------LPVDPT------------------------NHG-
N--GGSPVT--------------------
----RSIAAAKLQSP-------------------------HPFQ-KGM-------------------------
----

>Brachypodium_distachyon_4g44060.1.p

----MAS--
----------------------------------PPEPA----------------------------------
----

---IP------------
----------------------------V--------------LS----------------------------
-----------------PLSS------------------------RLSAA-----------ADSSS-------
--------VSSSAPVEHPC--
---------------------------------------FTLQNWWLVR-VEGEE-----------RKIAVS-
--------------GFT-------------------------------Q------------RG----------
-------------------------------------DAF---------------------------------
--------------------------------------TSAPIAKRHESLVLEDEDGVVVRIDGLM-------
SLCRMRRN-GFSLQ--------ICES-FLIGF--PSWW-------------ESW-------------------
---DSHFESQPTSSSNSQEDSS-------------
--------------------QIYLKI--
FQLGNVVQ---
KSVASFIKNPL-----------HDAKIFRR---------------------YVADAFT-------
QCSRFDEYSFD---NDTSTKGKTVASN--------
--DASEG--------------------------PAAVANEVDNMEIDL-------------------------
----
IVSSTSQERGHVDIS------------------CNASFAPTEK-------------------CTSDET-----
----

--YKEA---------------

ENQNDS----------MHPDVTEQE------------AGNHSVNSDL----------------ICNRSR------
----DRMPS--DLED--
GNTNAGNSTD----------------------------VALCHLATAQPERV-----------------------
---------------------------------NCCSEIPG----ALQNIQPLSNQRNPVA--------------
------------SL------------------------------KNQSHPKRTEDIS------------------
------------LNQKAVPIEDTS---
--------------TSIRSHVLS----------------SE----------------------------------
-----------------------------KTVGPSKKQRSA----------------------------------
------------QDKLLSPAR--

--LRG------------------------------

-----TRNP--

----------ISYVHHS--
-----------------------------PH----------------------------TRG-KAQ---------
-------------------------------------SLSI----------------------------
STPESLEM---------------------------TRTK-SG-----------------RVVVPPLDL-GC----
----ERILYGN---NHLVLGVAPV-------
---KLHSP-----------------------------
----PIKGSKPET---
PARKRR---

--AR-

>Brachypodium_distachyon_5g25291.1.p

----MAAKTP--QTPDPPF--------
----------------GARSRP---------SVPRPPGSA-----------------------------------
----

---IP------------
-----------------------------V--------------LSPSFS-------------------------
---------------RAPLRS-------------------------RLAAA------------ADTNA-------
--------AASSSSVEHPC--
--VTLWDWWLVR-VEGEQ------------RKIAVS-
--------------GFT--------------------------------Q-------------RD----------
--------------------------------------DAF----------------------------------
---------------------------------------TSAPIAKCHGPLTLEDEDGVVVLIYGSV--------
SLSRMREN-GFSPQ---------ICEK-FMIGF--PYWW-------------ESW--------------------
--DSHIESQPTSFSNLQEGSS--------------

--------------------QFNSEM--
CQLGKFLE---
KVEPSFIKNLL-----------NDAKNFPR---------------------DYEDAFT-------
ECPRFEEYTFD---NDISTKEKSGVSN--------
--DASEG--------------------------PAGVANEVDNMEIDL-------------------------
----
IVSSTSQERGHVDIS------------------CNVSFASTEK-------------------CTSDET-----
----

--YKEA---------------
ENQNDT----------VHPDAREQE-----------AGSRPVNSDL---------------ICNRSS------
----YRMPN--DLED--
GNTNAGNSTD---------------------------VPLCHLAAVPLGRA----------------------
--------------------------------NCCLEISG----ALQNIQLLSYQRNPVA-------------
------------SL-----------------------------KNQGHLQRTEDIS-----------------
------------LNQKAVPSEDTS---
--------------TSICSRVQT---------------QE---------------------------------
----------------------------KTVGPSKKQRSA---------------------------------
------------RDILLSPARLPVTRS-PMSSK-KQIFAH-------------------------------
DK--------------------LSSPTILPVTRSPMP------------------------------------
----
SKKQRSAQD---KLS------------
-------------------------SPA---
--TLPV-------------------

---------------TRSP--

--------------------ISSVYHS--
--------------------------------------PL---------------------------TRG-
RAQ--SSISI---------------------
------STPESLNM--------------------------KRTK-SG----------------RLVVPPLDP-
GC--------ERILYDN---NHLLLGVAPV-
--ELHSP----------------------
-----------LKGNKAGT--
-PAKKKS--

AR---

>Brachypodium_stacei_03G173200.1.p

----MASEP--QAGPSNHG------
------------------------------TTIQAPPPVSV-------------------------------
N--

--APIYR------
-------------ATIQKC--
--AALLDWWLVR-------------GQD-
DKIRVA---------------GYT---E---
--------------------------------------RNRAA-RVF--------------------------
----
TSDFITIRHADDTLETADHKIVLTRGPL-------NIEQMHRN-GFPYE--------VSKH-FQLGF--
PAQW-------------GKYA--
-------NSNMKQS-KQQAQSPSDK--
--SF-----------------------------
--VKSMDYNLK--------------
-----ETDF--
--NSSKGSTGNT----------DGTPM--
---------------QGLSNLSN---------------GTP--RFQDSSG-----------------------
----SG----PGETCS--SDQGDNQH-------------------QDMHLDA---------------------
----

------------EDAGQVRAKGMSPEFGVVQGS-------------------------------------
EDST---

LSRLRSGKVY--QKS------------
-------------------------TSGSA---
--SLKQ-------------------

G--
---------------HSKRK---
------TIQHNTPDRK---
---------------------MILNEETP--
--------------------------------------PPVDPT------------------------NHG-
N--GGSPVT--------------------
----PSTAAAKLQSP-------------------------HPCQ-KGIFTK------------RL--------
----

KTVDRTRET--

>Brachypodium_stacei_05G288200.1.p

----MAS--
----------------------------------PPDPA----------------------------------
----

---IP------------
----------------------------V--------------PS----------------------------
-----------------PLSS------------------------RLGAA-----------A-----------
--------------VEHPC--
---------------------------------------FTLQNWWLVR-VEGTE-----------RKIAVS-
--------------GFT-------------------------------Q------------RD----------
-------------------------------------DAF---------------------------------
--------------------------------------TSAPIAKRHEPLVLEDEDGVVVRIDGLM-------
SLCRMRQN-GFSLQ--------ICEN-FLTGF--PSWW-------------ESW-------------------
---DSHFESQPTSSSNSQEDST-------------
--------------------QMYLKF--
FQLGSVVK---
KSVASFIKNPL-----------HDAKDFRR---------------------YVADAFA-------
QYSKFDEYSID---NDTSTKEKTAASN--------
--DASEG--------------------------PAAVANAVDNMEIDL-------------------------
----
IVSSTSQERGHVDIS------------------CNAS-APTEN-------------------CTSDET-----
----

--YNEA---------------
ENQNDT----------MHPDATEKE-----------AGSHPVNSDL---------------MCNRSP------
----DCMPS--DLED--
GNTNAGNSTD---------------------------VALCHLATVQPERA----------------------
--------------------------------NCCSEIAG----GLQNIQPLSYERNTAA-------------
------------SL-----------------------------KNQGHLKRTEGIS-----------------
------------LNHKAVPSEDTS---
--------------TSVSSHVQS---------------LE---------------------------------
----------------------------KAVDPSKKERSA---------------------------------
------------RNILLSPTR--

---LPG-----------------------------

-----TRIL--

----------ISYAHDS--
----------------------------PL---------------------------TRR-RAQ---------
------------------------------------SLSI---------------------------
STPESLKM--------------------------KRTK-SG----------------RVVVPPLDL-GC----
----DRILYDN---NHLVLGVAPV-------
--ELHSP----------------------------

----LIKGSKPGT---
PARKRR---

--AR-

>Brachypodium_stacei_09G247600.1.p

----MATKTP--QTPDPPL--------
----------------RSGSRR--------SVPPPP-PR----------------------------------
----

---SVIP------------
----------------------------V--------------LSPSFS------------------------
---------------RVPLSS------------------------RLAAA-----------A-----------
--------SSSSSSVEHPC--
---------------------------------------VTLWNWWLVR-VEGEE-----------RKIAVS-
--------------GFT-------------------------------E------------RD----------
-------------------------------------DLF---------------------------------
--------------------------------------TSAPIAKCHGPLALEDEDGVGVLIYGSM-------
NCSRMGEN-GFSRQ--------ICDK-FMIGF--PYWW-------------ESW-------------------
---HSRIESQPTSFSNSQEGSS-------------
--------------------Q--STF--
QQLGKFLN---R-
ESSFIKNLL-----------NDAKNFPR---------------------DYEDAFM-------
ECPRFEEYTFD---DDISTKEKSAVPN--------
--DASEG--------------------------PAAVVNELDNMGIDL-------------------------
----
IVSSTSQERGHVDNS------------------RTV-FAPTEN-------------------CTSDET-----
----

--YKEA---------------
EKQNDT----------VHPDAREQE-----------AG-----------------------------------
----

---YQRNPVA---------------------
----SL-----------------------------KNQGHLQRTEDIS-------------------------
----LNQKAVPSEDTS---
------TSIGSHVQS---------------RE---
--------------------KAVGPSKEQRSA---
----QDILLSPTRLPVTRS-PMPSK-KQRSAQ-------------------------------DK--------
------------LSSPTRLPVTRSPMP--
---SKKQISAQD---------
--KLS------------------------------
-------SPT---
--------------------------------TLPVTRSPLPSK-----------------------------
------KQR-------------SAQD------------------KLSSPTRSPMP------------------
----

```
-------------------------------------------------------------------------
-------------------------------------------------------------------------
--------------------------------------------------------
SNKQRSAQDKLSSPTRSP-------------------------------------------------------
-------------------------------------------------------------------------
-------------------MPSVHHS-----------------------------------------------
-------------------------------------PL---------------------------TRG-
RAQ--------------------------------------------SLISI---------------------
------STPESLNM--------------------------KRTK-SE----------------SFALRRGSC-
GVAFTSQRKQDRDSYEE---------------------------------------------EKGSLSDFQT-
------------QD--------------------------------AAAQP----------------------
-----------EDADNLTT------------------------------------------------------
-AACCRC------------------------------------------------------------------
-------------------------------------------------------------------------
-------------------------------------------------------------------------
-------------------------------------------------------------------------
-------------------------------------------------------------------------
-------------------------------------------------------------------------
-----------------------------------------------------------------------
TRNSEGS------------------------------------------------------------------
-------------------------------------------------------------------------
-------------------------------------------------------------------------
--------------------------------------------------------------GCYAS------
---------------------------------------------LYMQ------------------------
-------------------------------------------------------------------------
------PGV----------
>Citrus_sinensis_1g023837m
-------------------------------------------------------------------------
-------------------------------------------------------------------------
-------------------------------------------------------------------------
----------------------------------MDPT----------------------KTPRF--------
-------------------------------------------------------------------------
-------------------------------------------------------------------------
-------------------------------------------------------------------------
-------------------------------------------------------------------------
-------------------------------------------------------------------------
-------------------------------------------------------------TKLS--------
-----------SHS-FKS-------------------------------------------------------
---------------------------------------VTLHDWWLVR-AE-G------------NGLAVG-
--------------GIA-------------------------------S------------RE----------
-------------------------------SQ-EV-RAF---------------------------------
--------------------------------------CSAAIAKRHNTTTLETADGITITLSNLI-------
NRSRTNQN-GFPSQ--------VCNR-FLHGF--PFYW-------------EEYA------------------
-----------------------------------------DQCCG---------------------------
-------------------------------------------------------------------------
-------------------------------------------------------------------------
-----------QEYTN------------------------RGS------------------------PF----
------------------------------------GAANIS-------------------------------
------------------LPLVSLDDI----------------------------------------------
---------------------------------------------------PATRL-----------------
------RDLLISPRGH---------------------------------------------------------
-------------------------------------------------------------------------
-------------------------------------------------------------------------
-------------------------------------------------------------------------
-------------------------------------------------------------AD--N-------
-------GALQS----------------------------------EIL------------------------
----------------------------------------D-------------------------------
VLRPCGC------------------------------------------------------------------
----------------------------------------------------SSVRDK-------------
TPSN-H-QS-KAASGRKYRA--------------------------------AV-------------------
```


KRPPSR---GVVTRSMAKLQ-
TLGSQ--

---------------------------------------QNKDS-----------------------------
--------------------AE---

-----------------------------------FSTQIEGMVTGNG-------------------------
----

------------------------------SQ-GC--------------------------------------
-------HAATFSDNDKEPPK-------------------SPGNSAV--------------------------
RRSI---
-----------------------------RLKNQAK-------------------------------------
----

>Citrus_sinensis_1g045557m
--HFDHFPSG-------------

--ELWS-----
----MISLINRLL---SPKSSTPAPS-------
----------------RASE----------NSNKSDDDDNG-------------------------------
D---------------------------------VLAS-----------------------------SRF---
----

----------------QKT--
--VILYNWWLVK-ANTD--------HEG-
NRLAIA---------------GFT-------------------------------S-----------RE---
--------------------------------------LQ-AK-RVF--------------------------
----
TSAPIAKSYDPFSLETSDGIYIIIRGLI-------NKSRTLEN-GFSSD--------VFSH-FNLGF--
HYDW-------------KAYA--
-------EKCFMKEVEAAL---------------------------DPI------------------------
--------------------------------------FA---------------------------------
--ADAGT------------------
------ANL--
----------------------DSNAIP--------------------------------VDHKKV-------
---KGKR------------------------

------------KRNAGDINKSFEYFS------------------ENTLASASKS------------------
----

-------------------------SDSSRHAANHK-------------------------------------
------------DTVNT--
--N--
--NHASTNFPQLM------------
---KSEKD-------------------------
------------NVSSLEDNLAVA---
--------------------------------------DT---------------------------------
----
------------AEPSGYCVRGSSKRM-------AN-------------------------------------
----

STSRRKGNTGTAAK--SKT------------
-------------------------EERS--

-----KLDGNTMKN---
--------------------VALGTSPV---
--------------------------------------PEG-------------------------SDTD-
NAN--------------PSN-------------------------LDPAVSLGDSEVAL--------------
---GIPRTSEKHKEN-------------------------DRSK-DG----------------------LDY-
---QNINSSPASLPQDF---DTNI-------
--RCPGEKS---------------S--

GIASS--

>Cucumis_sativus_094610.1

-------P--KTPATCS------

---TSII--------
-----------PSS-LKS---
---------------------------------------IFLYDWWLVK-ANDG------------EGLAIG-

--------------GFA-------------------------------S------------RE----------
-------------------------------RS-GI-RAF---------------------------------
--------------------------------------YSAAISKRHETTILEATDGIIISISGFI-------
NRPRTHEN-GFPPK--------VYNH-FLLGF--PFNW-------------KDYM------------------
---SS------------------------------

-----------GSIRK------------------------STV------------------------EF----
------------------------------------FKASTSRSND----------QGTSH------------
------------------YLEPDLDNL--
---AVTRL-----------------
------RDLCLSTYGE---

---S-----------
--------SHGH----------------------------------DLFMKN---------------------
---------------------------------------SNSSCCPTQ------------------------
SFSNEGKNDDVIK--
------------DSL--
-----HARQEAKKLDIDL--------------------------------QI---------------------
----

RRGQ--GVCTRSMTKLK-
NTRNRSKESLI--

-----------------------------------SDSRKKKKS-----------------------------
--------------------RK---

>Daucus_carota_025649


```
-------------------------------------------------------------------------
----MFTPPASS---------------ARKHQNNNSNSN---------------------SPPTL--------
-------------------------------------------------------------------------
-------------------------------------------------------------------------
-------------------------------------------------------------------------
-----------------------------------------------------------TPI-----------
-------------------------FNSL--------------LKTNWR-----------KRCNTTRR-----
----------------TPYRF--------------------------ASN--------NA-TPLASA------
----PPEQPHFVST-SKN-------------------------------------------------------
---------------------------------------VVLLDWWLTK-LQIE---GS-TSPKA-FKFGVG-
--------------GKA-----------------------------FDG------------RE----------
----------------------------------ST-HFF---------------------------------
---------------------------------------SGEIVKRQDEITLENVEGITIRLDSLL-------
NRFRTLEN-GFTSQ--------VCDH-FFLGF--PFDW-------------EEFG------------------
-----------------------------------------AQYFGGQSIHGA--------------------
-------------------------------------------------------------------------
-------------------------------------------------------------------------
-----------RSSKG-----------------------TRTSS---------------DDIDER--------
---------------------------R---------------------------------------------
------------------CLLSSFDDI----------------------------------------------
---------------------------------------------------PVTRL-----------------
------FDHMMVTSGD-------------------YNECSLT-------------------------------
-------------RSIFDHILSEYGS-----------------------------------------------
------------------SSAELKEE----DTDRIPAEDFLVD------------------------------
-------------------------------------------------------------------------
----------------------ETKTPL-------------DVSGENNKP-------LVAGCSSL--------
-------DKASR-------------------DELEHKDD-------NVILD--DVSMGTTNNL----------
-------------------------------LTVHSQSKMDEDVR---------------------------
ALSKFVPTRNKTRL----------------------------------------------------
ETLTSQQQE----GL----PSNTTTNPDT--------------------------------------------
------------TSRTSTRQYADTTVTHVASV----------------------SQSAIPNAANIK-------
----VDRVL----------------------------------------------------------------
-------DS----------------------------------------------------
SSSKNCTTCNHLGKNDS--------------------------------------------------
AKRGTMSEMLMNSQ-ELNLF-AATL---------------------------------DP-----------
GVTAPIPAPEMSRNRIKVPQVDME-------------------------------------------------
----------------------------------------------------SRTLNKSGS------------
-------------------------------------KKL---------------------------------
----RR-------------------------------------------------------------------
-----------------------------NLNT----------------------------------------
-------------------------------------------------------------------------
-------------------------------------------------------------------------
-------------------------------------------------------------------------
-------------------------------------------------------------------------
-------------------------------------------------GSGLLTRSQARGVLTRCRAKLK--
---------------------------------------------------------KLRTDSK---------
------D-----------------------------------------------------------------
ITAEHDV------------------------------------------------------------------
---------------------------------------------TAEED-AAH--------FA---------
-------------------------QTTKISDSCP---------------------ISEAEKLEK--------
------------------DESR-RHV-----------------------------------------------
----------------------------------------PDKVLEVNNRHL---------------------
--------------------------DRP--------------------------------EVRRS-------
------------------------------------------------------GRRR---------------
-------------------------------------------------------------------------
-------------------------------------------------------------NVVNY-------
-------------------------------------------------------------------------
-------------------------------------------------------------------------
-------------------------------------------------------------------------
-------------------------------------------------------------------------
```

---RER---------------------

>Elaeis_guineensis_00052_p0043.1
---MGAGEKTTPG--------------

NQDEPATAVLGSS--VTPMA---
---------------------RSSS----------STTPDPGGSNP---------------------------
----
TPC--
--TP-----

FTRS---
SSS------------------SSSEKNT---
---VLLSDWWLVK-AERG--------
AGG-KRLAVS---------------GFT-------------------------------T-------------
R---QQ-AI-RVF----------------------

TSAPIAKRYDAYTLETEDGITVIIQGMI-------NKALAHDN-GFPPE--------VCNR-FLVGF--
PYNW-------------DYYA--
-------D-EYFRRSSTCSSNSSSSAGFGE--LSKDSADGAGHTS--------------SVCVKE--------
--------------------------------------SPIGRICDIL-------------------------
----------------------------ISSGVTLSSNFT----------ETLKKFFRDPSL-
NPICQKPLLAVEKCGEHEM----------------DKSDQK--------------------------------
-----------HENDKGCA---------------TTI--------------------------
NHDAEGHVTSHA----------------------
EEGGHRNDLHAGDVPEENSKLNHDVSYDSLMHESRETDSQPAVEITSQTETGTAMRRETLL-----------
TKTRDVEKDNDLVVSVAMDCDNVDHVAACIDGK---ESAESR-------------------LESCKV------
------------------------------GM-------KYLLKDSLPKES--
NLTNHEHDEELGHDAVKRRHF-------------VE-----I----LRVP---NV--------GVSDDLLV--
----

S---------------MAQDD------------KAR------------------SKISSQNQVMVHSTILV--
-------AQD----------DADSH--PVEG--
-----------RGMDLN-----------------------------NL----KSPNVNI--------------
--TGISKDFSSN-----
-----------EIDDMCR---------------------------------RIFKKIESCKFPAARDSI----
----------------------PQIGSAVDMK---------STRASEVKESP---------------------
------------TNKHLTD---VV-KGGRKK---------------ENIQKEDQKCAEC--------------
--INSKVCLHFSMP-------AAQKSANEEKLTGKFSLCGTEELSST---------------
GDVQDDVSLIVLRTRNVPRNSSASSEGK-------PVKSSTNVKAD-ALSTR-MK------------------
----------SSKKKGDDNQMAR---------------
CSPEVEKIGVSSVPMDHNIVNAANDAKRESRHKHTQAVAKVGKVSLESLPTEHSSEDVNYGIQPCIISTPAAT
VVMHRQGDRHTNDCIGMNDDNENVTLKFPVKTTGHLANSV----KEQSSNDKTL-------------------
-----------------------------AMT-------------------------------------GEN-
----
---------------------IPNG--
---------------------------------PNDQN-----------------------------------
----

---------------------------------------EG-EG-----------------------------
----------------HPLAS---------------------------AALRSLGALT---------------
-----HRPLESSR-QH----------------R------------------CPS-------------------
-----------------------------------RGLRGP-------------LVIR---------------
----

>Eucalyptus_grandis_A00550.1.p

----MASS--SDPSR--------
----------------------------------SPDDG---------------------------------
D---------------------------------DFSA-----------------------------SRF---
----

----------------RKT--
--VCLHDWWLTQ-AADDG-------SQG-
RTLAVS---------------GFA-------------------------------SP-----------KQ---
-----------------------------------QSVQQ-VV-RVF--------------------------
----
KSAPITKRYDFSTLETADGVCVVVSGLI-------NKSRTTES-GFSLE--------IFKH-FVFGF--
PPNW-------------EDCA--
-------SKCLEKEAKIVTRPGKASFD--

--KGGKRI-----------------
------GKVD---------------EDNSLR--
----------------------NAT--

------------IVTSRRVTRSGS---------------------KSFALSSSRD------------------
----
---SSAMMEPN--------

LPNAEV----------EVTQD---------SCC--

--SAAV-----------------------------
--DE-----------------------------

--------------------------------------SK---------------------------------
----

VYSPQKTGGK---GKG-----------

--------------------------NVN--

------KNNFCGE--
----------PIVF-------KTPAGSQG--

--SSL----------------------
-----ISPDSF----------------------------KRSR-SG----------------RILLPRLEF-
W-------RNQVAVYAQ---DRGVAGVQE--
---------GP-SLILDDG-----------------------------LSP----------------------
----

>Eucalyptus_grandis_I00627.1.p

----MDRSG-------------------RRQEEGQVHKVG------------SMM-K---SLPSQ--------
----
--TQV----

---PPS-----------
---LPPNNR------------------------

---------------------------------------VFLNEWWLVN-------------ANG-KGLAIG-
--------------GFA-------------------------------H------------RD----------
-------------------------------SE-RV-SLF---------------------------------
--------------------------------------CSAPIVKRHDITTFETADGMAIMVGGLI-------
NRLRTLEN-GFSTK--------VCNK-FLLGF--PYDW-------------AEYS------------------
---TSSCGEYSSVQAGSVKAA--------------

-----------ASEKTDFW--SRSTTN--------
Y--
------------------FEATSLDDV--
---TVPKL-----------------
------RDL--

--FSLGD------------LDDSFLM-------
-------KKIYD----------------------------------DVV------------------------

---------------------------------GMSGSGAEHD------------------------------
----
-----GEFL--RSCRKSSPST--
----AAESQTNEA------------------------------------QINDKKS-----------------
--K-------------------------
ED--------VLC-----------------KHKEDI-------------------------------------
--------W--STRKIVKKV-N---------
-----H---

-----------------------------KSGRQL--------------------------------------
----------RAS-------------------------------------YV---------------------
--FR-------------

-------------------RRE-----------G---------------------------------------
-----------------------------VSTRSMTR------------------------------------
----

--FKGL---

>Fragaria_vesca_18236-v2.0.a2-hybrid.t2

----MASP--------------------PSY-----------------------------IQTL---------
--------------------------------------D---------------------------------
D------------------------------------R-----------------------------SHF---
----

----------------KPT--
--VSLYDWWLVK-SEDN--------DGK-
SKLAVA---------------GVS-------------------------------S------------AP---
--------------------------------------QEIPR-RVF--------------------------
----
NSAPISKKHDVFTLETTDGVTVILDGII-------NKQQTIAN-GFSDE--------IAKR-FQLGF--
YPGW-------------EK--
---------GVAEFLAGDC-NTTVV--------SGRISDSGSEGEGK--------
KDSSPTSVHFSQEETRTPYEHVGSTYFQWNGTSKNLEGNQRNPMSGCSMKHRSSKLLPLSDAFENVGEASGVA

VQSGGKMSEAERCPSNSARWVTRFLSEFWSKWTNQQMVGNSLNSEKKTTDSVLTVS---------
KTVINSSQ------------------------TDE----------------SEVGEK----------------
----
---EEGSHE---QVSCEEI--
HDS-------------------------------------EFSKQLSSR------------------------
LSLNNSINDM-------------------VNHAVEEETGTDDMV-----
NQAVEEETGTDDMVNQAVEEETGTY--DFISTALQSIGIPKTLE-ENEKNPESGCSIKHNSTNFSPSVDV---
---GVVVS---------GVTVPLE------------GETNLSKI----SPRNPGGRVF---------------
--GD---LSIIT---------
------------------------RSKGKTKQIVGS----GLDSE---------------QNTVD--------
----SVPAVE---------------S-VSNT-----------------SENVDY----------EVGGR--
QMFELFLSCCERAEGLINSSQTSVHFAHETPNKKVHCEDRLDVEDPTQIT--LSSDSTIDDSK----------
--------------------VDNLMNHAAEVEL------------------DAE-------------------
-----------NIGLTEFQSF----GRPQT-RDESEKNPASE------PAAETLE-ILDEAL-------
DVNA------------------QSG-E-KRDISVGSSSQFAGRVSLL-----------------------
FSGIIQRKEK--------------KKQTVGNGLNSETKTIDSVPVVSGSVSN---PLENDSFET-AGNWVSH-
--PSDSELQEPVDVNVVRKLDFDFVEDD----------MQQTSNAEEGQDFGNVKAQVREGKSG---------
-------AKGEIKSR-KRNMRQVTVDT---
QEIHSIPEVNKKNNRTPS-TLEVK-KKK----------------------------------D----------
---------GTPKANTKKRQVSASLE---
---GSKMRT-----STPSTLEVN----------
---------------------------------------NKE-------------------------------
------KV---
-------------------------------SLST--------------------------------------
--TKVYTRK--------------------

--RKVSPSTSDVSK-
---K----NK-------

-SSPLTLE---
--MNN-KKE---ATLP---S---PK--
------------------------VTAYTSL---------------------------ISPESLSA-------
-------------------GRSR-SG----------------RLRLRPLEF-W-------RNQSAVYDK----
---DHGVIGIQE-----------EI-PR-------
---------------------------VTP-SRG------------------------------SFTEPR---
--KRK--------------

--GN---------------------

-------------------------RG--

>Fragaria_vesca_21890-v2.0.a2-hybrid.t7

----MASP--------------------PSS-----------------------------TET----------
----
D------------------------------------R-----------------------------PHF---
----

```
-------------------------------------------------------------------------
-------------------------------------------------------------------------
----------------KPT------------------------------------------------------
----------------------------------------VSLYDWWLVK-SQDN--------HGN-
NRLAVA---------------GVS-------------------------------S------------AP---
--------------------------------------QE----RVF--------------------------
---------------------------------------------
YSAPICKKHDVFTLETTDGVIISLNGTF-------NKQQAIAS-GFPDE--------VAMH-FQLGF--
YTGW-------------EK------------------------------------------------------
---------GVAELLEQDF-DTTGV--------SGRISDSGSEG---------------
PTSVRFSQEETRTPYEHVGSTYFQWIGRSKNLEVNQKNPMSVCSMKHKSAKLLPSLDAFENVGEALGAAVQSG
GGNSEAKRCLSNYIHRVSRFLSEFWSKWMNQQMVGNGLNSENKTTDSVLTVS---------KTVNNSLQ----
--------------------TDE----------------CEVGEKG---------------------------
--------------VESSCLTS---------------------------------LHL----SLEEGSHE---
QVSCEEM----------------------------------------------------CDS-----------
--------------------------EFPEQLSSR------------------------QSLNNSPNDM----
---------------VNHAVEEETDTE-----------------------------------
DFISTALQSIGIPRTLE-ENEKNPEYGCSIKLSSTSFSASVDV------GVVAS---------GVTIPLA---
---------GEINVSKT----CPRNPGGRVS-----------------GD-----------------------
--------------------------------LSIIT---------------------------------
RSKGETKQIVGN----GLNSE---------------QNTVD------------SVPAVE--------------
-S-VSTT-----------------SQSVDY----------EVGGR--
QMFESFLSCCQRAEGLINSSPTFVHFARETPNEKVHCEDRLDVEGPKQITTNLSSDSTIDDSK----------
--------------------VDDLMNHAAEVEL------------------DIE-------------------
-----------NIGLTEFQSF----GRPQT-REENET------------ATEALE-VQEE----------
VNS------------------------KKEVSLSA--------------------------------------
LKANTK--------------KKH----------------------VSA---SLEGSKKRT------ST---
PSTLEVNN------------------------------------------------KEKVSL-----------
-----STTKVNTK-KRKASP----------------------------------------------------
STSKVSKKNKLSPS-TLEVN-KK--------------------------------------------------
-------------------------------------------------------------------------
-------------------------------------------RT-----STPLTLEVN--------------
-----------------------------------NKE-----------------------------------
--KV---------------------------------------------------------------------
---------------------------SLST------------------------------------------
------------------------------------------TKVNTKK------------------------
-------------------------------------------------------------------------
-------------------------------------------------------------------------
-------------------------------------------------------------------------
--------------------------------------------------------RKASPSTSEVSK-----
-------------------------------------------------------K----NK-----------
----------------------------------------------------------------------
LSPSILE------------------------------------------------------------------
-----------------------------------------------L-------------------------
---------------------------YSI---------------------------ISPESLST--------
------------------GRSR-SG----------------RLLLRPLEF-W-------RNQSPVYDK-----
----------------------------------------DHGVIGIQE-----------EV-PR--------
--------------------------VTR-SRG------------------------------SLSKPR----
-------------------------------------------------------KRK---------------
-------------------------------------------------------------------------
-------------------------------------------------------------------------
-------------------------------------------------------------------------
-------------------------------------------------------------------------
-------------------------------------------------------------------------
-------------------------------------------------------------------------
-------------------------------------------------GNGDFHKFAGRHR--KFRGL----
-------LYDESSEH----------------------------------------------------------
--------------------------------IAPL-------------------------------------
-----------------------FRGCSQH-------------------------------------------
----------------REIYFCKSNYRTA--------------------------------------------
```

```
---------------------HFQKRRK---------------------------------------------
------------RKEE----------------------------------------------------
>Fragaria_vesca_22410-v2.0.a2-hybrid.t1
-------------------------------------------------------------------------
-------------------------------------------------------------------------
-------------------------------------------------------------------------
----MTST----------------------------------------------------PPPPFS-------
-------------------------------------------------------------------------
-------------------------------------------------------------------------
-------------------------------------------------------------------------
-----------------------------------------------------------APR-----------
-------------------------------------------------------------------------
------------------------------T------------------------------------------
-----------VSASLKS-------------------------------------------------------
---------------------------------------VLLDDWWLVK-AQGN------------NALAVE-
--------------GFA------------------------------RS--L---------RP----------
----------------------------------AI-RTF---------------------------------
--------------------------------------SSAAISKRHTATTLETIDGIIVTLCGLL-------
NISRTSQN-GFPPE--------IYDR-YLLGF--PFDW-------------EEHA------------------
-----------------------------------------AALLGQGSTGKSASAR----------------
-------------------------------------------------------------------------
-------------------------------------------------------------------------
-----------NSSQK-----------------------YMSL----------------EHSESN--------
-------------------------------------------------------------------------
------------------QLPFSISDV----------------------------------------------
---------------------------------------------------PATAT-----------------
------RDFIMSCVGD---------------------------------------------------------
-------------------------------------------------------------------------
-------------------------------------------------------------------------
-----------------------------------------------------------SEHL----------
-------------------------------------------------------------------------
-------KTILD----------------------------------DILRT----------------------
-------------------------------------------------------------------------
-------------------------------------L-----------------------------------
-----GD----NVFGDTTPQINS-----------------------NM-------------------------
----EDSDPVEKV------------------------------------------------------------
------------------------------------------------------------------------
ES---------GC------------------------------------------------------------
-------------------------------------------------------------------------
-------------------------------------------------------------------------
-------------------------------------------------------------------------
-------------------------NETLTMAKKV--------------------------------------
-------------------------------------------------------------------------
-------------------------------------------------------------------------
--HIDG-------------------------------------------------------------------
-------------------------------------------------------------------------
-------------------------------------------------------------------------
-------------------------------------------------------------------------
-------------------------------------------------------------------------
-------------------------------------------------------------------------
-------------------------------------------------------------------------
-------------------------------------------------------------------------
-------------------------------------------------------------------------
-------------------DRN---------------------------------------------------
-----------------------------KSANIHSK------------------------GSQKSK-
KGMYSG-------RNLTGKISVPRRSS------ARLKNKNEMQDS----------------------------
-------------SMTLDSEL-------------------EVMH-----------------------------
-------------------------------------------------------------------------
-------------------------------------------------------------------------
-------------------------------------------------------------------------
```


>Glycine_max_09G003000.1.p

----MAAS--KCNSATPLI--------

--PVP------------

---------------PKSL--
---------------------------------------IFLHEWWLVK-------QR--------KGLAVG-
--------------GLA-------------------------------S------------VE----------
----------------------------IADRE----RVF---------------------------------
--------------------------------------LSSVIVGREETNVLHSEDGITILFRGFI-------
NTSRSSQN-GVPFQ--------VCQH-FLVGF--PHDW-------------KKYS------------------
---AYSFGDA-------------------------

F--
------------GDSSV---------------------C-SKNI-----------------------------
----

---CH--
-----GL--

--------------------G--
---ISSGNH------------------------
--------SKQSQS-----------------TDNMEC--EC--------------------------------
----
---------------------------N---

-----NTASQLQVG---

EK---
----------------G------------------------------ISDVAVESQAGNKKS-----------
----

------------------------FMSMFDSNKCT--------------------------------------
----------STS-------------------------------------GKG--------------------
----
--TVKP---

--LTSKEKMD---------------------

--------------------RKE-KQQ---
IRQKI--------------------------VLDASNSCNR-------------------------MVTRSI-
SK-----------------R-----------------SHTMPKEDE-----------------------------
------------------KATIATVVSP---
-VRRSP---
------------------------------RLYSRR---------------------------------------
----

>Glycine_max_10G043400.3.p

----MADS---------------------TPT------------------------------ATPS---------
---------------------------------------N----------------------------------
DT-----------------------------------VS------------------------------SYF---
----

----------------QRT--
---VTLYDWWLIK-AKND---------FQG-
KRLAVA----------------GVS--------------------------------S------------RK---
---------------------------------------DE-AM-RVF---------------------------
----
VSAAVIKRYDVFSLETADGICVMISGFI--------NEQRTLEN-GFAAE---------VFNR-FLFGF--
PPDW--------------ESYA---
-------LDCFREESTTGT-DLGSAVPDNVPASSLEILSDGVE--------------------------------
----
-----------------------------NSIPTSLASP-------------KEAPG------------------
------DHE-----------------KSFPG--
--NECNVS---KEM-------
--G----------------------------

-----------------------GVNVACS---

--------------------------------------SGG-K-RRSARLHD-----------------------
-----------------IKVHQQK-------------KKP-----------------------AS----------
-----GGSLKN---PNNENSTL---
VAL-----------------ENCDVEGL-KSPVTP--
-----------------------IQSQS-SRQLS-TS--------------------------------------

-----------------------SGQLVKKSAS---
--RISRT-----LSPKTEGCY-
---KKK----------------------
---------------RV---
--TVE-------------------------------
--RKVGRPKG----------

KLNKSASAVKNPQE--
-K-----D--
----------------LSHLTKG---
--SQQ-K-----
--IST---------------------------
VTPESPSF--------------------------RKSR-SG-----------------RLLLPPLEF-W-----
--RNQIPIYNA--DHEITEIQD--------
---GA-SL-----------------------------------ISP-CKGF-----------------------
------SPSLSRF--
SNQKRG--

---A--

>Glycine_max_13G131100.2.p

----MADS--------------------TPT------------------------------ATPS---------
--------------------------------------N----------------------------------
DI----------------------------------VS------------------------------SSF---
----

----------------RRT---
--VTLYDWWLVI-AKND---------FQG-
KRLAVA---------------GVS--------------------------------S------------RK---
--------------------------------------DE-AT-RVF---------------------------
----
VSAAVIKRYDVFSLETADGICVIIRGFI-------NEQRTLEN-GFSAE---------VFHH-FLFGF--
PPDW-------------ERYA---
-------LDCFKEEPTTDA-DLGSVVPDNAPASCPKILSDGVE-------------------------------
----
----------------------------KSIPTCLVSP------------EEASG-------------------
------DHE----------------MSFPE--
--NECNVS--KEM-------
--GG--------------------------
------------VHVACSSGG-------------------------KSHSFKLHNI------------------
-KVCQQKKQPASECLP-----NHPDNE----------------------
NSSSVALENCNVERLESPTTPIQPQL-------WSEYSNDACV------ENA----------------------

```
---------IPTSLASE----EAPGDHEK--------------------------------------------
-------------------------------------------------------------------------
--------------------------------------------------SFPENE---------------
SNVS---------------------------------------K--EINGVNVACS-----------------
-------------------------------------------------------------------------
-------------------------------------------------------------------------
----------------------------------------------------------------SGG-K-
SRSARLHD--------------------------------------IKVYQQ--------------KKP----
------------------AS---------------GGSLKH---PNNENSTS---------------------
------------------------------VAL----------------ENCDVKGL-KSPATP---------
----------------------------------------------------IQSQS-SRQLS-TS-------
-----------------------------------------------------PGQVIKKSAS----------
-------------------------------------------------------------------------
----------KISRT-----LSPKTEGCY--------------------------------------------
-----KKK-------------------------------------RV--------------------------
----------------------------------------------------------------------
TVE----------------------------------------------------------------------
---------------TKVVMPKG--------------------------------------------------
-------------------------------------------------------------------------
-------------------------------------------------------------------------
-------------------------------------------------------------------------
---------------------------KLNKSASALKNPRE--------------------------------
----------------------------K-----D--------------------------------------
-------------------------------------------LSPLAKG-----------------------
-------------------------------------------------------------------------
-----------------SQQ-K------------------------------------------------
IST---------------------------FTPESLSF--------------------------RKSR-SG--
--------------RLLLPPLEF-W-------RNQIPIYNA--------------------------------
-------------DHEITEIRD-----------GA-SL----------------------------------
ISP-CRGF-----------------------------SPSLSRF-----------------------------
--------------------------SNQKRG-----------------------------------------
-------------------------------------------------------------------------
-------------------------------------------------------------------------
-------------------------------------------------------------------------
-------------------------------------------------------------------------
-------------------------------------------------------------------------
-------------------------------------------------------------------------
-----------------------A-------------------------------------------------
-------------------------------------------------------------------------
-------------------------------------------------------------------------
-------------------------------------------------------------------------
-------------------------------------------------------------------------
-------------------------------------------------------------------------
------------------------------------------
>Gossypium_raimondii_004G087600.1
-------------------------------------------------------------------------
-------------------------------------------------------------------------
-------------------------------------------------------------------------
----------------------------------MGKR----------------------NRERR--------
----------------KSEK-----------------------------------------------------
-PC-------------------------------KS------------------------------DQL----
-------------------------------------------------------------------------
-----------------------------------------------------------VP------------
----------------------V--------------------------------------------------
----------------PI-----------------------------GSP-----------TPLA--------
-----------NLS-LNS-------------------------------------------------------
---------------------------------------VLLHDWWLCM-VQ-P------------KGLAVG-
--------------GFE-------------------------------C------------RG----------
-------------------------------RQ-GQ-RVL---------------------------------
--------------------------------------CSAAIAKRHDATTLETADGITVAISGFI-------
```

```
NTSRTLQN-GFSPK--------VCSH-FLFGF--PYDW-------------EEYA------------------
-----------------------------------------SHSNE---------------------------
-------------------------------------------------------------------------
-------------------------------------------------------------------------
-----------QSFCS------------------------STE------------------------------
------------------------------------ASITTSLLHS----------NAMS-------------
------------------LPLPSLDNL----------------------------------------------
-------------------------------------------------HVPAARL-----------------
------RDLLMLSAGD---------------------------------------------------------
-------------------------------------------------------------------------
-------------------------------------------------------------------------
-------------------------------------------------------------------------
-------------------------------------------------------------SP--N-------
-------SVFDH----------------------------------MLL------------------------
----------------------------------------Q-------------------------------
KLSTHDSQNAAIT------------------------------------------------------
TADSNM---------------GNKDPKV----------------------------CPYSVADG---------
----ENSNCH-KE-KVIQNHVDDN--------------------------------NIL-C------------
---------------------------------------SR---------------SKTTVGSR---------
---N--ED-------------------------------VGIL----------------------------
TPTNTR-----------------------------------------------------GVITRSMTRLK-
RRTSE-RL--------------------------------------------------------------
KRLRLS-------------------------------------------------------------------
--------------------------------------SSELKEKVL--------------------------
-----------------------RR------------------------------------------------
-------------------------------------------------------------------------
-------------------------------------------------------------------------
-------------------------------------------------------------------------
-------------------------------------------------------------------------
-------------------------------------------------------------------------
-------------------------------------------------------------------------
-------------------------------------------------------------------------
-------------------------------------------------------------------------
-------------------------------------------------------------------------
-------------------------------------------------------------------------
-------------------------------------------------------------------------
-------------------------------------------------------------------------
-------------------------------------------------------------------------
-------------------------------------------------------------------------
-------------------------------------------------------------------------
-------------------------------------------------------------------------
-------------------------------------------------------------------------
-------------------------------------------------------------------------
-------------------------------------------------------------------------
-------------------------------------------------------------------------
-------------------------------------------------------------------------
-------------------------------------------------------------------------
-------------------------------------------------------------------------
-------------------------------------------------------------------------
-------------------------------------------------------------------------
-------------------------------------------------------------------------
-------------------------------------------------------------------------
-------------------------------------------------------------------------
-----------------------------------------------------
>Gossypium_raimondii_006G174400.1
-------------------------------MFLFLFLKEKLGK--------IYHQQPTV-------------
-------------------------------------------------------------------------
-------------------------------------------------------------------------
-STAMAPK--------------------TRD-----------------------------KNDGN---AIQ--
-----------------------------------NDSN---------------------------------
```

```
DG----------------------------------SS-----------------------------SYF---
-------------------------------------------------------------------------
-------------------------------------------------------------------------
-------------------------------------------------------------------------
-------------------------------------------------------------------------
----------------QKT------------------------------------------------------
----------------------------------------VCLHDWWLIK-AEKE--------FEG-
KRLSVA---------------GST-------------------------------S------------IE---
--------------------------------------SK-AF-RLF--------------------------
---------------------------------------------
TSAPIVKRHDALTLQTADGICVCIRGFI-------NKQLTIEN-GFSSE--------VFTH-FFIGF--
PPYW-------------EKYA----------------------------------------------------
-------KECLGETIMADV---------------------GLE------------------------------
-------------------------------------------------------------------------
------------------VVPNSSNAARDSGPSLISTP-----------CNN---------------------
--------A----------------FNLPTM------------------------------------------
--VEERTN---------------------------------------------------LDSSQQK-------
---------------------------------------------GE--------------------------
-------------------------------------------------------------------------
-------------------------------------------------------------------------
----------------------------------------------------------ASTIKVQ----
DEQNLNRK-----------------------------------------------------------------
-------------------------------------------------------------------------
-------------------------------------------------------------------------
----------------------------TLSCL----------------------------------------
-------------------------------------------------------------------------
-------------------------------------------------------------------------
-------------------------------------------------------------------------
-------------------------------------------------------------------------
-------------------------------------------------------------------------
---------------------------TSKL-NH---------------------------------------
---------------------------VKES-SLE--------------------------------------
-------------------------------------------------------------------------
-------------------------------------------------------------------------
-------------------------------------------------------------------------
-------------------------------------------------------------------------
-------------------------------------------------------------------------
-------------------------------------------------------------------------
-------------------------------------------------------------------------
-------------------------------------------------------------------------
-------------------------------------------------------------------------
-------------------------------------------------------------------------
-------KETRK------------------------------------------------------------
KLDFEEV------------------------------------------------------------------
---------------ASSVSRE---------------------------------------------------
--------------------------------------------------------------RKG-N------
------------------------------------------KSI---------------------------
ISPESLNF--------------------------KRSR-SG----------------RVLLPRMEF-W-----
--RNQIPVYDQ---------------------------------------------DRRITGIKE--------
---EV-DD-----------------------------------VN-SSG------------------------
------SRSNPK---------------------------------------------------------
YQKR---------------------------------------------------------------------
-------------------------------------------------------------------------
-------------------------------------------------------------------------
-------------------------------------------------------------------------
-------------------------------------------------------------------------
-------------------------------------------------------------------------
-------------------------------------------------------------------------
-------------------------------------------------------------------------
-------------------------------------------------------------------------
```


>Hordeum_vulgare_2Hr1G067110.2
--SLRSAP---------------

---NMARK---TRNPPS--------
----------------RARSRR--------GAPPPPSPS----------------------------------
----

---SSTAA-----------
---------------------------LA--------------LSPPFS------------------------
---------------PVRFCG------------------------RLGAA-----------EAAAA-------
-------------DVEHPH--
---------------------------------------VTLWEWWTVR-LKGED-----------RKLAVS-
--------------SFT-------------------------------E------------KN----------
-------------------------------------DLF---------------------------------
--------------------------------------TSAPIAQRYESLTLQYEDGVVVLLYGSF-------
NSSRMREN-GFSMQ--------ICER-FMIGF--PYWW-------------ETW-------------------
---DSHMESYPNLFIHPQEDSI-------------
--------------------QFYLEK--
FQLANFIQ---KFAPFLIKD-
L-----------NDAKKLPI---------------------NDLYAFI-------GGSRFQEHNCG-------
----------------------------------NDVSTKESSAASE----------DAR-------------
---------------PAVVA----YVEIGL---
---NPSSTSHERDHVNIE-----------
-------GNVSLAPTET-------------------YSGDET-------------------------------
----

------------------------CKEA---------------GNQNDT----------MHPDAREDD-----
------VGSHLFNSDW---------------TCTMPP----------NHMPN--DSEG---------------
-----------------------------------GNAT--NAEL---------------------------
LALDPLARVQPKSS--
NCCSEIAG----AFQSVEPLSYQSAPVA-------------------------PL------------------
-----------KNQQYLERNEHIT-----------------------------LTQKAVSNKTVP--------
---SSIHSDVQS---------
------PK---
KTVGSAKKQRSA---KQGLGRPTR-------

---LNSAPYA-----------

RL---------------------------TRS-RVR-------------------------------------
--------ALSI---------------------------STPESLKM--------------------------
RRTR-SG----------------RVIVPQLD-------SVR--SWVVYDR-----------------------
----------------------SKIKL--

>Hordeum_vulgare_2Hr1G067130.1
---LRSAP---------------

---NMARK---TRNPPS--------
----------------RAKSRR--------GAPPPP-PS----------------------------------
----

---SSTAA-----------
---------------------------PT--------------LSPPFS------------------------
---------------PVWFCG------------------------RLGAA-----------EAAAA-------
-------------DVEHPH--
---------------------------------------VTLWEWWTVR-LKGED-----------RKLAVS-
--------------SFT-------------------------------E------------KN----------
-------------------------------------DLF---------------------------------
--------------------------------------TSAPIAQRYESLTLQYEDG---
GLWCSFMVHLTHRECVRM----GFSMQ--------ICER-FMIGF--PYWW-------------ETW------
----
DSHMESYPNSFIHPQEDSA---------------------------------QFYLEK---------------
-------------------------------FQLGNFIQ----------------------------------
---------------------KFAPFLIKV-L-----------NDAKKLPI---------------------
NDLDAFI-------GGSRFQEHNCG---
NDVSTKENSAASD----------DAR----------------------------PAAVA----NVEIRL----
----
-------------NPSSTSHERDHANIE------------------GNVSLAPTET-----------------
--YTGDETCKE--AGNQNDTMHPDAR-
EDDAGSHHDLDAFTGGSRFQEHNC-------GNDVSTKENSAASDDARPAALANVEI----CLNSSSTSQER-
---DHVNIEGNVSLAPTK---------TYIGDE--

SCKEA---------------GNQNDT----------MHPDAREDD-----------AGSHLFNSDW-------
--------TCTMFP----------NHMPN--DSEG--------------------------------------
------------GNAT--NAEL---------------------------LALDPLARVPPKSS----------
--NCCSEIAG----AFQSVEPLSYQSAPVA-
------------------------SL-----------------------------KNQQYLERNEHIT-----
------------------------LTQKAVSNKNVP-------------------------------------
--------------------------SSIHSDVQS---------------PK---------------------
--KTVGSAKKQRSA---------------------
------------------------K--

>Hordeum_vulgare_2Hr1G073590.2
---M-----------------------

RTRSMASQP--EPGPSTHG------
------------------------------TAAPAPPPASV-------------------------------
SASTHGTAA--

---PAPP----------
--------------------------TASA---------------SASIHGT---------------------
----------------AAPA--RPVHH------
-------------ATLQRC--
--VTLIDWWLLR-------------GQG-
GKICVT---------------GYI---------------------------------DPI--------FR---
--------------------------------------RKRSV-RLF--------------------------
----
ISSPITVRHAEGTLETADHRFVLTRGPL-------NIKKMDCN-GFPSE--------VSNQ-FRLGF--
PIQW-------------EKYV--
-------NSNMKQA-NEHTLSPEKST---------------------------------EYSVEE--------
--------------------------------------FLCGSF-----------------------------
--ANSLEHTLP--------------
-----GFDF--
--RTSKESTGNA----------DGPGLPNYVK---PR-----------VQEPFGNSGDYDNSMSNM-------
------------AASEGLCNDRV---------------GMPDESFED--------------------------
----PG----PGDTCNGQASRADNSH-------------------EDTQTDASGK------------------
----

------------RIVSH--
--------------------------------------SVDSALVSNDIDKI---------------------
----

--------------------------------------EE---------------------------------
-----------------------------EHGPSKLGNNS-----------VCPG------------------
-----------TEHVLEVLNKVVSTEHGSVQCP--

KKLRSGKVY--EM--------------
-------------------------SDGA--
---SSKR--------------------

---G-

-------------YSKRK---
----AMQHEALSMK---
-------------------VIPNEETA--
------------------------------------PPAGPT------------------------SRK-K--
---GASVA-----------------------
-QITALDKLQSH-------------------------GPGR-KGN----------------------------
----

>Lactuca_sativa_2_36541.2

--MPNLS------------------------
------------------------------S----------------ASK-----------TPIS--------
-----------SSNYNKS---
---------------------------------------VLLHKWWLIK-V-----------ESE-SKLGIG-
--------------GYV-------------------------------N------------RE---TLGSRG-
MRLLGSASASG----------GKRCNNNNNLNT-ES-KVF---------------------------------
--------------------------------------YSAAISKRLDNNTLETVDGFTITIIGSI-------
HSSRTLSY-GFSPQ--------VCDH-FLSGF--PFNW-------------EGYF------------------
----

--GNF----------------------
-----------SHEKC-----------------------ERSL-P----------------------------
---------------------------------LSFN------------------------------------
-----------------DLPVTRARDI--

```
-----LMSTA------------------------------------QESEACA--------------------
-------------------------------------------------------------------------
--FT----------SMILKDILKQTE-----------------------------------------------
------LD-----------------------------------------------------------------
-------------------------------------------------------------------------
-----------------------------------------------------------HKQD----------
------GSSL------------------------------------KIMEE----------------------
----------------------------------------DCGN----------------------------
S------------------------------------------------------------------------
-------------------------------------------------------------------------
--------RETEAA-----------------------------------------------------------
-------------------------------------------------------------------------
-------------------------------------------------------------------------
---------------------------------------------------RVSSRAETLPE-
SPSDTFSSLS--------------------------------------------------------
DIKGEKCNKTRVLGSKWMPLR---------THEGYPLRS----------------------------------
-------------------------------------------KRLKKSN-----------------------
-------------------------------------------------------------------------
-------------------------------------------------------------------------
-------------------------------------------------------------------------
-------------------------------------------------------------------------
-------------------------------------------------------------------------
-------------------------------------------------------------------------
-------------------------------------------------------------------------
-------------------------------------------------------------------------
-----------------------------------------------ETD-----------------------
-------------------------------------------------------------------------
-------------------------------------------------------------------------
-------------------------------------------------------------------------
-------------------------------------------------------------------------
-------------------------------------------------------------------------
-------------------------------------------------------------------------
-------------------------------------------------------------------------
-------------------------------------------------------------------------
-------------------------------------------------------------------------
-------------------------------------------------------------------------
-------------------------------------------------------------------------
-------------------------------------------------------------------------
-------------------------------------------------------------------------
-------------------------------------------------------------------------
-------------------------------------------------------------------------
-------------------------------------------------------------------------
-------------------------------------------------------------------------
-------------------------------------------------------------------------
-------------------------------------------------------------------------
-------------------------------------------------------------------------
---------------------------------------------------------
>Lactuca_sativa_6_35500.1
-------------------------------------------------------------------------
-------------------------------------------------------------------------
-------------------------------------------------------------------------
----MAFD------------------------------------------------AADNSPPSR--------
-------------------------------------------------------------------------
------------------------------------------------------------------SHF----
-------------------------------------------------------------------------
-------------------------------------------------------------------------
-------------------------------------------------------------------------
-------------------------------------------------------------------------
---------------KKS-------------------------------------------------------
---------------------------------------VSLRDWWLTKPQSDDDR----------NTIGVS-
```

--------------GLT-------------------------------ST-----------SQ----------
----------------L--------------NR-AE-RCF---------------------------------
--------------------------------------SSAPIVKAYDFFELETVDGVCVILQGYI-------
NKEKTLGS-GFSSE--------VFDH-FVIGF--PSYW-------------EEFS------------------
---TSCPQRRSSDEC--------------------

-----------VSTAH------------------------EDE------------------------------
----

------------------------------KHSIEQHGKFH-----DMNTR----------------------
----

---L-------------------------

ED----------C--

----------KDE--

--VLSS---

-----------------------KS---
TSL-----------------SPH-----GPTETECYSG--------------------------KRSR-SG--
--------------RVLLPSLEF-W-------RNQTVVYDAGL------------------------------
-------------DRQVTGVSVA-------WDHGI--------------------------------------
---SRG---
-------------------------RVRGKN--

--------------------I--------------------
>Manihot_esculenta_08G078100.1.p

----MAAS--------------------ASL-----------------------------SNPSN---I----
--------------------------------------D---------------------------------
NT----------------------------------CS-----------------------------SYF---
----

----------------QKT--
--VSLCDWWLIK-ADED--------FQG-
KRLAVA---------------GVT-------------------------------S------------RE---
--------------------------------------QR-AV-RVF--------------------------
----
HSAPITKRHDVFTLETADGIVVIFQGFI-------NRTRTIEN-GFPSE--------VFSH-FLFGF--
PPYW-------------EEYA--
-------DNCFKQAVNSDV-NSQNTLDVDEP-----ITDQGLED-------------
VRPTPCKNKDTSDNECYIKVCS-----------LDVSKKYIVDASKDSDLDKDAIELMSSVKNISTYSGV---
------------------------------LNDKLIAKLSISQNESHPSIFVCPR-----DNDDTVDNMEH--
----------------------DAA----------------KNLPPS--------------------------
------------------TSCLTNVV------------DSMND-------------NIAV----EAR------
--LLSYHSI--GSSGMI------
-------------YDTRS---------GKESQKMCTR------------------------KSKNKESVSL--
-----------------ESNTIKENASEEC--------------------------------------
SVSNSAYMDNLIV-----------------SEFDNTETA------GAEGL---------VLTL----------
-----CPKSLKKE----N-QNKGKKG---

---------GMKSGS-------------KNFSTG------------SMPRDL---------------------
---EAVLGCIGEGEVICEES-------------
-------------------LLRSTTAVNS---------------------------------DILKQPSN---
---NF-QVTVC----
STVQTVAKENEDAPS-----------------MVESS-------PVNAIP-S-------------DSGS-R-
KCSTPLRD--------------------------------------LKDNKK--------------QKS----
------------------IS---------------GCSVNH---KSKELISE---------------------
------------------------------VD-----------------EHLDGSKL-KK-------------
---RSHS-TFQAE-KE-------
---ISQKM-LSAA----------

----------RILRN-----LSGTVGSKE--
-----KSE-------------------------------------QV--------------------------
--SV-

--------------------------NKGGSSMKKARR-----------------------------------
-------------------------KIIFDAQ---
--ATPLTGE--------------------------

--------------RKE-K--TCV---
------------------------FSPESLSL--------------------------KRSR-AG--------
--------RLLLPTLEF-W-------RNQIPVYDA--------------------------------------
-------DRNITGIQE-----------EF--------------------------------------RA-
SRG------------------------------CNSEPQM---------------------------------
----------------------RSSKRK---


```
-------------------------------------------------------------------------
-------------------GK----------------------------------------------------
-------------------------------------------------------------------------
---SP--------------------------------------------------------------------
-------------------------------------------------------------------------
-------------------------------------------------------------------KRH---
-------------------------------------------------------------------------
--------------------------------------
>Musa_acuminata_06_p22880.1
-------------------------------------------------------------------------
-------------------------------------------------------------------------
-------------------------------------------------------------------------
----MGPE-------------------------------------------EDVRSSVFLAKPSV--------
----------------------------------APSSS------------------F---------------
----------------------------------ASA-----------------------------TPF----
-------------------------------------------------------------------------
-----------------------------------------------------------PPS-----------
-------------------------------------------------------------------------
-------------------------------------------------------------------------
-----------FPADKKS-------------------------------------------------------
---------------------------------------VFLFDWWLIK-AESE--------VEG-KRLAVG-
--------------GFT-------------------------------T------------------------
-------------------------------RQHAT-RLF---------------------------------
--------------------------------------SSAPIIKRNDAYTLETADGVTVVVQGMI-------
NKERTQDN-GFPLE--------VCNH-FLIGF--PYNW-------------DHYA------------------
-----------------------------------------DEYSNKRSTSTSPGKLSSLDE----
AFKDSTNRTAST--------------FPVLLDE----------------------------------------
------FPICRVLNFF-----------------------------------------------------
TSGGDNLTTNFS----------DHLKNLSMSAA-----------------ESEM----------------
QKSSSY-----------------------------------------LMEGLGKHNMDNN----------
DGNIVG--------------------SASSVEHHAEVLTCSKEV------------
EYSRKNLNEGGGYRITV--------------------------------------SKQELVDMRK--------
------KASSNNSRKMETCSNLFSFISVNNNST------NHLQVSVDQK-------------------
LDAEGRNNP------------------------------VEEGR-------SSISNSPLLQDVS---------
--------------------------------HL---------------------DYSEKLGDDTA-------
---------------------------------------------------------G---------------
-------------------------------------------------------------------------
------RLCHSDKSI--------INDTDGLPIP------------TNLDNKDAYSPHG----------
KIDVKL-------KISDVIGTL-KAGTWQEGSVG--------------------------------------
TRYVEGELNGFDHS-----------------------------KISKHCSTSNEGES------------SS--
-------------------------------------------------------------------------
-------DEINGMCL----------------------------------NTLEKIKNCNIPS------------
--------------VENPTTQMCSTVDIGL---------TGDSEMDMCC------------------------
---------QKEPLVK---VIEEKEIKK---------------DSIQKQNQDCTEC----------------
IGEKDFVHFSASVE-------MMPLAEDKLTGKTTSTGTEEQSSAVKGHNYPYVSGTCKRGGVTLGIPA----
-------SVSRSEGL---KGKSVFSSKMKVKRV-
SKNYEVASTKLETGETKPNHLNSEEKGFKHTSEYHASVKGDNKDASTGSP----
AQDPTDVSQICTFDSFKQEVGADPLKC----------------------------------------------
--------------------------------------------RLQSSL----LSSHAATKEL---------
----------------E----------------------KQN-----------------------
GLAVDHIA------MENNS--------HSNG---------------SPRYST------KY-------------
---------------------------------------AVQS-DIPVMNY-------------
SSEDKSLEMI------------------YK---------------------------TLMDKMNNQDVPKV--
-------------------------------------------------------------------------
-------------------------------------------------------------------------
-------------------------------------------------------------------------
-----------------------------------------------------------------------
SVAEESNKIKGSHTSRRRK-------M----------------------------------------------
-------------------------------------------------------------------------
-----------------LRQVSYVHQS----------------------------------------------
```

--------------------------------------PL---------------------------TRD-
KA---KKSFV---------------------
------ALSESLNF--------------------------RRSR-SG----------------RLIVPPLDN-
--------GCQQIIYDA---
DGSITGIMSAR------------NVLN---------------------------------SP-SQG-------
-----------------------SKSE--
------------PAKRRK---

---KL----

>Nelumbo_nucifera_010241510.1

----MATT---SPTT--------
----------------ASNQIL--
D---------------------------------TRTM-----------------------------SSA---
----

--------------------S--

----------------LRM--
--VCLQDWWLVK-SEHN--------FHG-
KRLAVG---------------GFA-------------------------------T------------RQ---
RL---------------------------------------AG-RVF--------------------------
----
CSTPIIKRHDSFTVETADGVVVTVKGLI-------NNARTCQN-GFSTE--------ICSH-FLFGF--
PFDW-------------EDYT--SYFFG-
-------NECTTDS---
--GGAL-----------------
--TNIKGF-----------------
-------------------------DETEVH--
---SYNSTNHDMPF-----

------------HLDKYPVTEI-----------------------RDCLISITGN------------------
----

-------------------------SGNSALP--------------SSIFA----------------------
------------DIIEK--CTNSISEKAGE--
LVHMFINGSNTST----------------------------SLSSLSA-------------------------
----
--EASRECKKANLRQNEIDNDIF------
-----PATN-GRLECENVKLEDQGNLN---
HDGCNET---KSETLKD-KFTVQSDDVLE-----------NAPNDSLFR----------------------
VK---TELVRLS---
---------------------KVELVESRSTSNRN-GVR---GSGL---------------------------
------DV---

LKRKKRSFS--

--TVES-NY----------------

--------------------LTRSKRLQ---
---NRE-E-

----LSENPHGKE-----------------GEEQMVCSQVSR-EV----------------SVLLK-----W-
---CMK----DKVKSNVGTR---
------HGKVD--

>Nelumbo_nucifera_010253233.1

----MTTD--------------------YAS-----------------------------SQSH---------
------------------------------------VNK---------------------------------
TI----------------------------------SS-----------------------------S-F---
----

----------------LRT--
--VCLQDWWLIK-AEKD--------FDG-
KRLAIG---------------GLT-------------------------------S------------KQ---
--------------------------------------CQ-VG-RVF--------------------------
----
SSAPIIKRYDIFTLETADGINVMIKGFI-------NRSRTHQH-GFPDE--------VCNH-FLIGF--
PYDW-------------EAYA--
-------GEYFGNESNIGG-IP-----------------SGECS-----------------------------
----
--------------VNVEAPACPNMKSSSSSNWLATFSE------------ETPN------------------
------KNK----------------KAKSKQ--
--EECRNKQLNG----------NFNND-------------------------------TGPSSDKV-------
---DGVN------------------------

--GIPGQ------------SESRRSCY----
RARSLKIKF--
--KEGQ-------------TTN---

------------SSQEV------------S--------------------DFVKSV-----------------
VNNIDC----------KSGGK--SA--

--FDWPDAGPQ----------
---DTN-S-ESLLKDHDSG----------------
--------------------CHKHVS--------------KNCL---------------------LS------
-----ST-TTDARQE---PTNENSMGH--
-----------------------GEAKSSNL-NQSFR------------------------------------
-----------------------SLIGSADC-TNELK-NT---------------------------------
----

---VGNEKC------

NFHDISMSGRKTKR---
-KL--
----------------VSTFS-Q--
---ERK-H-----
---SSI---------------------------
VSPECLSF--------------------------RRSR-SG----------------RLLVPTLEF-W-----
--HNEQVVYDA---DGQITGIQK--------
---GL-PT----------------------------------IET-SAG------------------------
--------SRS-H---
TQEKKK---

KRV--

>Oryza_sativa_01g34610.1

----MATQ--PRA--------
-------------------------G--
DGAAA--
AAAAKE---
--AP-----

------------------AVSYLQAC---
--VELDDWWLER-VEGE--------E---
GKVRVV---------------GSN-------------------------------T------------TT---
--------------------------------------SR-AG-RRF--------------------------
----
TSASIKTRHASGDLETEDGIIIMIARPP-------NISKMHLN-GFPDE--------VSKH-FSLGF--
PVQW-------------ENII--

-------NANMAEMNKQPQSPLKST----------------------------------EYYIEK--------
--------------------------------------FL---------------------------------
----R---GNLKY------------------
-----SMGL-------------------------FSW------------------------------------
----------------------DGLNIY---

------------QGSRSDADRFPS---------------------ERLSNSSNGR------------------
----

--------------------PTVEDP---

--------TANTDCNV---NFMGTLATSEEFCTG--------RM-----------------------------
------------DMPEE---
PRATPSETCGNDQENNQ--------------------------HLCMLMNTCENGNK----------------
---VQHGTSSVGPSVV-------
--PAEKYVRSK----------------

-------------------AEQDALLVN---------------DSTSHVSSVLGDCATPKC------------
---G-----------------------------
-----------------KSLTHLGTKDALETN---
----------------------EGMNPQFGVPQGSEGST----------------------------------
----
VRRLRNGKVIVIST--SAS------------
-------------------------TK--
--KVYK-------------------

------------RA---
-----RMQDNTFSEN--
--------------------VIPNKNVT---
-------------------------------------CPTGL-------------------I-----SQE---
---NVGSVAV-----------------------
----TAAAKLQIHD----------------------TPRKGR-RG----------------RL----------
----
---R-------------------

KSEKRKRS---

>Oryza_sativa_04g28040.1

----MGTEPS--QTPTPPP--------
----------------------------------PPPPA----------------------------------
--SPRRR--

```
-------------------------------------------------------------------------
---------------------------------------------------------APQPA-----------
---------------------------TI--------------LSPSFS------------------------
---------------RNPL--------------------------RATAA----------SASASAG------
---PVPSPSSSSDCEEHPC------------------------------------------------------
---------------------------------------VELFDWWLKR-VEGDD-----------RKVRIA-
--------------GHT-------------------------------E------------RN-H--------
-------------------------------KP----HLF---------------------------------
--------------------------------------TSAPIVKRHKACMLEAEDSIIVLIDGPL-------
DLSQMENN-GYSLE--------VCEK-FMTGF--PCLW-------------ESY-------------------
-----------------------------------------NLGSQQSCSYTSISRDGRT-------------
--------------------KFYLER----------------------------------------------
FQIGNFID-------------------------------------------------------
KVGSSFLANLL-----------NNSRS---------------------
SSGNDADSFEKGSYLSNKKPRFEEYTCD-----------------------------------------
LDISAKEKTTAFN----------EGSTG--------------------------SLAVCNKVGNQQIDL----
-------------------------------------------------------------------------
-------------VVKSFSKERGHGNID------------------LSASLTSIEE-----------------
--TTRDKT-----------------------------------------------------------------
-------------------------------------------------------------------------
-------------------------------------------------------------------------
---------------------------------------------------------------SEDA------
---------GNQNEF----------IHSDAEYQE-----------AGSHLVNSDSIY-------------
GMSTESGNQN------EFIHA--DAEH----------------------------------------------
QEVGSHVVNSDSNFD--------------------------MSTDNMICEMGDGSA-----------------
------------------------------------NAGSAVSQGSKEVLATVLPERANLSPDSCLD-NI---
------LPISTCNSNNCL-----------------------------ENQGFPEIAQHMT-------------
----------------LNEEVVPNEDIS---------------------------------------------
------------------TSVHSDVES---------------------------------------LG-----
--------------------------------NPVGPAEVQRSE-----------------------------
----------------CDILQGAPRSPKQNVGSAQEQ--------------------------R---------
-------------------PEQSMSQGAARSPMIRTPIPDGAPSLR---------------------------
-------------------------------------------------------------------
NQHLGSAQE-------------------------------------------------QRS------------
---------------------------------------------------------------------EHF-
--------------------------------------------------MLKG-------------------
-------------------------------------------------------------------------
-------------------------------------------------------------------------
-------------------------------------------------------------------------
-------------------------------------------------------------------------
-------------------------------------------------------------------------
--------------ATRSP-----------MIRTP--------------------------------------
-------------------------------------------------------------------------
--------------------IPYGHYS----------------------------------------------
--------------------------------------PL---------------------------TRG-
KAK---------------------------------------------SSSV---------------------
------STPESLKL--------------------------RRTR-SG----------------RVVVPTLDP-
GC--------QRIVYDR---------------------------------------------DGLVSGVAGL-
----------------------------------------------EFESP----------------------
----------PLKGNESRT------------------------------------------------------
-PESKRR------------------------------------------------------------------
-------------------------------------------------------------------------
-------------------------------------------------------------------------
-------------------------------------------------------------------------
-------------------------------------------------------------------------
-------------------------------------------------------------------------
-----------------------------------------------------------------------
VR-----------------------------------------------------------------------
-------------------------------------------------------------------------
-------------------------------------------------------------------------
```


>Populus_trichocarpa04G07690
----------------------------------MIVIK----------------RNPN--------------
----

-SILIMST--------------------ISL-----------------------------SS-----------
----
--F----

---------------S---
---TVT-
--------------SLL-------------------------------S------------------------
-------------------------------KK-PV-RVF---------------------------------
--------------------------------------HSAAIPERYDVFTLQTADGVNVLLQGYI-------
NKTLTIEN-GFSSQ--------VFRH-FCFGF--PPDW-------------EE--------------------
---CGTKLLNSNC-ESA----------------

--AEPPVSQNECRPIFLLLP-
------VDDGVNNLKN------------------------EDS----------------KNLSPL--------
------------------------------------SSCRVNDV------------NWVKD------------
-SVVV----PVKPSGHHVDVALSSEKI--
------GS--------------------------------------KKSKTRSFG------------------
------KLMAKRSSSL-------------------ERISIKNDASGEC-------------------------
-------------SALTDYNVGNITK-----------------SNFDQTRSV------RTSGV---------
VLAVSLE------------VSSSLKKK----KRENEGNKD---------------------------------
----
-----------------------GLKSGS--------------DFSMP------------SLPQGP-------
---QVMLRCI-ENNTT---


```
-------------------------------------------------------------------------
-------------------------------------------------------------------------
-------------------------------------------------------------------------
-------------------------------------------------------------------------
-------------------------------------------------------------------------
-------------------------------------------------------------------------
-------------------------------------------------------------------------
-------------------------------------------------------------------------
-------------------------------------------------------------------------
-------------------------------------------------------------------------
-------------------------------------------------------------------------
-------------------------------------------------------------------------
-------------------------------------------------------------------------
----------------------------------------
>Populus_trichocarpa04G07740
-------------------------------------------------------------------------
-------------------------------------------------------------------------
-------------------------------------------------------------------------
----MTTE--------------------VAT-----------------------------SS-----------
-------------------------------------------------------------------------
--------------------------------------------------------------------F----
-------------------------------------------------------------------------
-------------------------------------------------------------------------
-------------------------------------------------------------------------
-------------------------------------------------------------------------
---------------QKT-------------------------------------------------------
---------------------------------------VLLHDWWLIK-AEQE--------FQG-KKIAVA-
--------------GLT-------------------------------S------------TE----------
-------------------------------KK-PV-RVF---------------------------------
--------------------------------------HSAAITKRYDVFTLQTADGVNVLLQGYI-------
NRTLTVEN-GFSSQ--------VFRH-FCFGF--PPDW-------------EE--------------------
-------------------------------------------CGTKFLNSNC-ESA----------------
-------------------------------------------------------------------------
------------------------------------------------------AEPPVSQNECRPIFLSLP-
------VDDGVNNLKN------------------------DDS----------------KNLSPL--------
------------------------------------SSCRVNDV------------SWVKD------------
-SVVV----PAKPSGHHVDVALSFEKI----------------------------------------------
------GS--------------------------------------KKSKTRSFG------------------
------KLMAKRSSSF-----------------------IKNDASGEC-------------------------
-------------SALTDYNVGNITK-----------------SNFDQTRSV------RTSGV---------
VLAVSLE------------VSSSLKKK----KRENEGNKD---------------------------------
-------------------------------------------------------------------------
-----------------------GLKSGS--------------DFSMP------------SLPQGP-------
---------------------------------------------------------QVMLRCI-
ENNTTQGPS--------------------------------LSRSPTRGDS----------------------
-----------DILNQSEK------------------------------------------------------
--CLVENSVF----SSMRSGAEANIISPS-----------------MEETE-------TLDANL-I-------
------DSGH-R-KSTASVIN--------------------------------------LIDIPK--------
------QKS----------------------VS---------------GHSVRY---KAKRLIS---------
----------------------------------------------------------------VEG-KP-
KR------------------------------------------------------------------NSGT-
TL-----D------------------------------------------------------------
IAEAM-LRAS---------------------------------------------------------------
------------------------------RKMNT-----LSGNSKSKE------------------------
-------------------------KKE-------------------------------------TI------
-------------------------------------------------------------------------
-----------------AV------------------------------------------------------
-------------------------------------------------------------------------
-------------------------------------------------------------------------
-------------------------------------------------------------------------
```

--EKGGLSMKQARR---------------
---KVIFDAD---------------------
--IREMT--------

--------------TCI---------------------------HSPESLNL---------------------
-----KRSR-SG----------------RLLLPTLDF-W-------RNQIPVYDE------------------
---------------------------NRNITGVQE-----------EL-GV---------------------
-------------VKP-SRG------------------------------VESKTQ-----------------
--KSKRE----------------------------

>Populus_trichocarpa07G12430

----MSTD--------------------PK-RKGSSSA----------------------TTPAS--------
----------------LTKI---
-LH-------------------------------RSL-----------------------------SHL----
----
---KS------------
----------------------R--
----------------TP-----------------------------NNI-----------TPVS--------
-----------SLS-LKS---
---------------------------------------VWLYDWWLAK-AE-G------------DGLAVS-
--------------GFT-------------------------------F------------RE----------
-------------------------------FVFGH-KLFY--------------------------------
-------------------------------KLGSIFLLMITIVRRHYATILESKDGITVTISGFI-------
NRDRTREN-GFSFQ--------ICEH-FQLGF--PYSW-------------LELA------------------
---TRLGG---------------------------

-----------EESAN------------------------RGS-------------------PPGKSGF----
------------------------------------DEPKMSSGTS----------AS---------------
------------------AASVSFDDL--
---PVTRI-----------------
------RDIATHPLGD---

---S---K-------
-------DCALA----------------------------------DIL------------------------
--D-------------------------------
HFCSNDAKL---S--
PLATSP---------------GSKNPVT----------------------------VANAVLDE---------
----TPRK-N-KR-RADRKYKDGG--------------------------------IIP--------------
----
-----RRV-------------------------------DIVTG--------------------------

```
EHITPSR-----------------------------------------------------GIVTRSMSRRR-
NFREI-R----------------------------------------------------------------
EESPS--------------------------------------------------------------------
-------------------------------------SNPTVSCKA---------------------------
----------------------SRN-------------------------------------YP---------
----------------------EKV------------------------------------------------
-------------------------------------------------------------------------
-------------------------------------------------------------------------
--------------------------------------------LPSSA------------------------
-------------------------------------------------------------------------
-------------------------------------------------------------------------
-------------------------------------FLSTSETTKNLAG--G--------------------
------------------------------------------TMST---------------------------
-------------------------------------------------------------------------
-------------------------------------------------------------------------
-----------------------------SARRD-SW------------------------------------
---------ATTSTEKIME-----------------------VPDVPLV------------------------
--RRSS-S-----------------------------------------------------------------
-------------------------------R-PNIRKDYR--------------------------------
-------------------------------------------------------------------------
----------------------------------------EK-------------------------------
-------------------------------------------------------------------------
-------------------------------------------------------------------------
-------------------------------------------------------------------------
-------------------------------------------------------------------------
-------------------------------------------------------------------------
-------------------------------------------------------------------------
-------------------------------------------------------------------------
-------------------------------------------------------------------------
-------------------------------------------------------------------------
-------------------------------------------------------------------------
-------------------------------------------------------------------------
-------------------------------------------------------------------------
----------------------------------------------------
>Populus_trichocarpa17G03610
-----------------------------------------------------MADVPTH-------------
------------LSING--------------------------------------------------------
--------------------------------------------------AK-----------------
KEKTRPGKMSSE--------------------AKEREGFSSS----------------------TTPAS----
--------------------LTKI-------------------------------------------------
-----LI-------------------------------RSL-----------------------------
SRL----------------------------------------------------------------------
------------------------------------------------------------------NP-----
-----------------------------R-------------------------------------------
-----------------------TP-----------------------------SNT-----------VPLS-
------------------SLS-LNS------------------------------------------------
----------------------------------------------VWLHDWWLVK-VE-G------------
NGLAVS---------------GFT-------------------------------S------------RE---
--------------------------------------GV-GT-RLF--------------------------
---------------------------------------------
CSAAIVKRHYTTILEAKDGITVTLSGFI-------NRDRAHEN-GFSFQ--------ICDR-FQLGF--
PYSW-------------EEIA----------------------------------------------------
-------AKLRG-------------------------------------------------------------
-------------------------------------------------------------------------
--------------------------------------------------EESAN------------------
------GGS-------------------PRGKSGL--------------------------------------
--VELNMSSGIS----------TN---------------------------------TASVSFDDI-------
-------------------------------------------------------------------------
-----------------PVTRI-----------------------RDILMHPLGD------------------
-------------------------------------------------------------------------
```

```
-------------------------------------------------------------------------
-------------------------------------------------------------------------
-------------------------------------------------------------------------
---------------------------P---K--------------DCALE----------------------
------------DIL----------------------------------------------------------
------G-------------------------------SFCSNTMEH---T----------------------
--------------------------------PMLTDPF-------------SNSKSPVT-------------
---------------VA---------------------RK-R-KRTKADQKHRDGG-----------------
---------------KIT-------------------------------------------------------
-------------------------------------HTD-------------------------------
DTVMG--------------------------ECITPRR-----------------------------------
------------------GVVTRSMSRLR-NL-----------------------------------------
-------------------------------------------------------------------------
-------------------------------------------------------------------------
--------------------------------------------------AKN--------------------
-----------------NP-------------------------------G----------------------
-------------------------------------------------------------------------
-------------------------------------------------------------------------
-------------------------------------------------------------------------
-------------------------------------------------------------------------
-------------------------------------------------------------------------
-------------------------------------------------------------------------
-------------------------------------------------------------------------
-------------------------------------------------------------------------
-------------------------------------------------------------------------
-------------------------------------------------------------------------
-------------------------------------------------------------------------
-------------------------------------------------------------------------
-------------------------------------------------------------------------
-------------------------------------------------------------------------
-------------------------------------------------------------------------
-------------------------------------------------------------------------
-------------------------------------------------------------------------
-------------------------------------------------------------------------
-------------------------------------------------------------------------
-------------------------------------------------------------------------
-------------------------------------------------------------------------
-------------------------------------------------------------------------
-------------------------------------------------------------------------
-------------------------------------------------------------------------
-------------------------------------------------------------------------
-------------------------------------------------------------------------
-------------------------------------------------------------------------
-------------------------------------------------------------------------
-------
>Setaria_italica_K96788
-------------------------------------------------------------------------
-------------------------------------------------------------------------
-------------------------------------------------------------------------
----MASE----------------------------------------------------PPPGSTG------
------------------------------EATRTP-------------------------------------
-------------------------------------------------------------------------
-------------------------------------------------------------------------
----------------------------------------------------------PAPS-----------
-------------------------------------------------------------------------
------------------------------------------------------------AAAAPR-------
-----------VSYVQQC-------------------------------------------------------
---------------------------------------VVLVDWWLER-VEGEE-----------GKIRVA-
--------------GIASTAQMRH---------------LLLPKGASSST-----------GN----------
-------------------------------RNVAG-RVF---------------------------------
```

--------------------------------------RSAAIGRRHDQHAIETEDGYKIQIGRLL-------
NVPRTRDN-GFPEK--------VCKC-FEFGF--PIQW-------------LKLV------------------
---NPKMEQQ-NEQAQSESTAD--APR--------
-----------------HSVKYWMEE--
FLSDDL---
------------TNLKKYASE-------------------ENDS-----------------------------
-------------------------------------YSSAGYTSNT----------DGPAIQS---------
----------------LSNLPDGNAGNM-------------------AASGGLYGGRT---------------
NMPGKPLARPR------------------------------------ETSCSGQESDQH--------------
-------ESMQIDTSEQ--

---GLDNH---------------------

SISSVSVNQNTGSF---

-----CPNSKVDDS------------------IL-------------ATSKIMSVEKESY-----------
RRRVGSSK---ADEDADIQH-------------
--EN-------MQSCS----------------NEHEIVTLPID-SA------
IVNENPNSTSSDLEKPGTPKCGKAS-----------MNLG-----------------------------
STDALELPTERMTPQFGAVQGS-------------------------------------EDSP----------
----
--VRRLRSGKVFG----------

----MPSG--------------G--
-----------------------------LMKS--

---G---------------------
---HKKR--
---KIQHEASSQN------

MIPNEGDT---
-----------------STADLT------------------------SHE-NDS-------------------
-------------------------SAAGG---------------------------VTKDKQES--------
------------------HDSH-RGISA---

--KKAKKKRESSK------------

------------------------------------LFW------NW--------------------------
----

---C-----------------------

>Solanum_lycopersicum_04g015710.3.1

----MAEA---

```
-------------------------------------------------------------------------
------------------------------------------------------------------NNFT---
-------------------------------------------------------------------------
-------------------------------------------------------------------------
-----L-------------------------------------------------------------------
-------------------------------------------------------------------------
-----------SSSFLKQ-------------------------------------------------------
---------------------------------------VYLYDWWLIK-VETG--------DGS-KRLGVG-
--------------GFT-------------------------------A------------KE----------
------------------RPDG---------------RVF---------------------------------
--------------------------------------HSTTIAKRHDTTTLVTEDGITILLSGFI-------
NRCRTLQN-GFSSE--------VCKQ-FLLGF--PYNW-------------EESA------------------
-----------------------------------------AVSFGESTNENAASRISDF-------------
-------------------------------------------------------------------------
-------------------------------------------------------------------------
-----------SESANASAD-----------------CTSSSF------------------------------
-------------------------------------------------------------------------
--------------------TLSVDHL----------------------------------------------
--------------SPNVL------------------------------------------------------
------RDLLISAAGD---------------------------------------------------------
-PEGGM-------------------------------------------------------------------
-------------------------------------------------------------------------
-------------------------------------------------------------------------
-------------------------------------------------------------------------
-----LRKSIFN----------------------------------EIVQK----------------------
------------------------------------------------YGNNA------------------
FNVDEASSLNQKSG-----------------------------------------------------------
-------------NQVTPQSPSLNG-----------------------SP-----------------------
------SQKKKAKTNRKQE------------------------------------------------------
-------------------------------------------------------------------------
-DS----------CIADAKS------GK---------------------------------------------
------------------------------------------------EKLPEATPKDMPEKR----------
-----------VLD------------RLLHRSGDDVPIVGE-----------NSCLNQKSGNQVSSRGPSLD-
-------------------------------------------------------------------------
--------------------------ETPYKKKKTR-------------------------------------
--------ANLRKE-----------------------------------------------------------
----------------------------------------------------------------------
DDK---HVPN---------------------------------------------------------------
-------------------------------------------------------------------------
-------------------------------------------------------------------------
-------------------------------------------------------------------------
-------------------------------------------------------------------------
------------------------------------------AQCRKE-----------VLP-----------
----------------------------------KCNDESGTD------------------------------
-------------------------------------------------IDKNSSSS----------------
-------------------------------------------------------------------------
---------------SAL-----TRD-KAS------------------------------------------
LYKKTKI-------------------------YLTQEEKRDVH-------------------------KVSG-
QG-----------------------------------------------------------------------
-----------------DFGIVNITN-----------------------------------------------
-SSNGPLTRS---------------------------------------------------------------
-----------------------------RAKMKR--------------------------------------
-------------------------------------------------------------------------
-------------------------------------------------------------------------
-------------------------------------------------------------------------
-------------------------------------------------------------------------
-------------------------------------------------------------------------
-------------------------------------------------------------------------
-------------------------VKEQGQE-------------------------------GH--------
-------------------------------------------------------------------------
```


RYL--

>Solanum_lycopersicum_09g010550.3.1

----MASP--------------------STCQTQRVEEK---------------------KEPKS--------
----
---------------------------------SNSSK----------------------------SCF----
----

---------------QPT---
---------------------------------------VSLKDWWLIR-AERD--------SQG-RTLAVA-
--------------GRT-------------------------------S------------RE----------
-------------------------------GQ-AL-RGF---------------------------------
--------------------------------------TSAPIHKIYDVFNLETIDGICVVLKGFI-------
NRSRSEEN-GFPSE--------VIEQ-FLFGF--PPQW-------------ETFN------------------
---EKFLG---------------------------

-----------RDSKG---

---KASASY--------
------------------------------ALGFEK-------------------------------------
----

------------------PSG--

-----------C---

---------SEK-------------------------------------VK----------------------
----
-DLKNLD--

------------------QND--
-------------------------YVETTGETIQD--------------------------HNGR-KD----
----

>Sorghum_bicolor_KXG20573

----MKPL--PVPEA--------

---------------------------G--------------------------------------SPHRRG-
----
---MPP-----------
---------------------------SL--------------LSPSSRS-----------------------
---------------AVP-----------------------------AAAD----------------------
-------GDHDAAVSEHAC--
---------------------------------------VTLSEWWLAT-AEGDD-----------QKIAVA-
--------------GTF-------------------------------E------------RN-Q--------
----------------------------------TV-QEY---------------------------------
--------------------------------------SPAPIAKRHTSSVLETEEGTVLRLHGLH-------
NVLRTYHN-GYSAK--------VYSE-FLNGF--PDWW-------------QSCK------------------
--PCNP--------------------------------KLMNSHTECCSSNASNSGVDST-------------
--------------------QFYLER--
YMQGRRLDSY-----------G-----------------TYLI--------------------
SKFPDILASFL-----------HNDAVFQK--------------------SSHL---------
LNGKPRFEEYTCD---GDITTNENAAASS------
----EAATGD--------------------------------QRIPEVSL-----------------------
-------------------------EVRGCRKE----------------------------------
TQHMSLTDKAAVDEEMP----------------ASVYLDMQNS------------------------------
---------------------LC--
--LSNGTPILEEYTCD-----------------
--GYI------------------------
--PPNEDA---------------
AASNDDNE-------------------------RYIATSKEVNNMEKIVLVTG--------SPSRERGHD---
----DIATD--
-----------------------------VAVSELVHSTPATGT-----------------------------
----------------Y----------------------------RKKTPVA---------------------
----SL-----------------------------KSQGSWKENQPVA-------------------------
----SNKKMK--------------------------------LI-----------------------------
------------------------------DP----------C-----------LGK----------------
--------------------QHVGRPKKRISP---
----HAKCQSATRSPGTRN--

--PAS----------------------------Y-

--------------------------------VLWS-------------------------------------
----

--------------------PL--------------------------TRD-KA------------------
---------------------------TSLSM--------------------------STPEDLEL------
--------------------KRSR-SG---------------RVIVPKLDN-WC--------QTIVYGR---
---DGLIAAVIGL---------------------
----------------------------DSP------------------------------ALPKWSESKT-
---D-----RRKKRKTK-----------

>Sorghum_bicolor_KXG30438
--MDSQIWPIPPD-------------
------------TSRSG-------------------------------------AISI--------------
----
VHIMKSQKSRSRGMPSQ---
PPPGSTD-----------------------------------RSPRTP------------------------
----
------APF---

QPAPD-----------------------------------STGG---------------SP-----------
--------LGSRR------------------RSPPRPA----------------------------------
----PATSR----------------------DMQ--------------------------------------
--VTLVEWWLER-VESKE--
---------GKIGVA--------------GEACVPQMRHQSKEGKIGVAGEAYVPQMSEGAFSSK-------
----PN--K--AR-RLF-----------------

RSCAIVKRLGYCTIESEDGYHIRIDGPL------NISKTREN-GFSEK--------VCEC-FKWDF--
PEQW-------------QGLV---
-------NPKMVPDYNEHARSPAETTTAAP--

--SPHEDVDMS------------------II-
------------A---

---------------------------------G---------------------------------------
--FFSS-----------------------------

>Sorghum_bicolor_OQU89324

----MPSQ--PAPDSTG------
------------------------------GSPRIPVASAEAVRR--------
VVDFFKTNGKLRSVTGAAVSSPLLPRGAATP-----PLAGMPSQ-------PPQGSTGGSP------------
----------RISAPF---

----QPAPD------------------------------------YTGG---------------SHRTPAP-
G-----------SIGVVP------------------RIPELSA------------------------------
---------QAVDR------------------IA--RKC----------------------------------
--IILVDWWLER-
VEGEE-----------GKIRVA---------------GTTFTPRMA----------------EQMRKGASSS-
------------N---MRMAV-RVF----------

RSSAIVKRHDYTSIESEDGYQIEIGHCL-------NIPKTREN-GFSEE--------VCES-FDFGF--
PDLW-------------QRLV--
-------NPKMVPD-DEHALSPSETTTGPPS-------------------------PSVEDYMAK--------
--------------------------------------FLSDSF-----------------------------
--SSKIGFDFT--------------
-----END---

---FDS---------------------

---GSI---------------------

---SSDSKVDGN------------------IL-
------------APSKISSVVNEGY-----------RSTVGCGQ-----------------------------
--------------AKKDANIQQ---------------EN-------MPSCS----------------
SEHAMVT-------------------------------PKFGKDS-----------VNLG-------------
----------------TTDALELPTEGMTPKFGAIRGS-----------------------------------
--EDSI---

GRRLRSGKVL---
--------------------------PIG--------------G-----------------------------
--PMK--------------------

----------------KQK--
-----KIQ-----QQ--
--------------------MV-NQGAT---
-------------------------------------PAADLT------------------------SHE-
NDF--SAAEV---------------------
------VVKENLGSDDSC---------------------GKVTG-QG----------------RIAE------
----

GKGKRKRKR--

--VW---

C--RFSYYP----------------------

>Spirodela_polyrhiza_6G0070900

----MGNS--PSRG-------
---------------FRMSEV---
DK-------------------------------LGRVED----------------------------AAVA--
----
--GTPPS----------

---ISSASC------
------------VSSMEKT--
--VLLHDWWLIK-VRDE--------CDR-

ERLAVG---------------GLT------------------------------A----------F------
-------------------------------------GK-AA-RIF--------------------------
----
NSAPVAKRYDAYTLETTDGITVRIQGLINIL------------GPNPF--------LIDH-
FFRAVENPRRW-------------HQMK--

--ISLTK-----------
-------------RHI--

--M---

-------------------ELILH--

---RKGEKPH--
---------------------------------------GCLRVTL--------------------------
--DRLL------------

>Triticum_urartu_13040-P1

```
-------------------------------------------------------------------------
-------------------------------------------------------------------------
-------------------------------------------------------------------------
----MSRL-----------------------------------------------------------------
-------------------------------------------------------------------------
-------------------------------------------------------------------------
-------------------------------------------------------------------------
-------------------------------------------------------------------------
-------------------------------------------------------------------------
-------------------------------------------------------------------------
-------------------------------------------------------------------------
-------------------------------------------------------------------------
-------------------------------------------------------------KK----------
-------------------------------RNRAG-RVF---------------------------------
--------------------------------------SSGSITVRHADGTLETADNKIVLTRGPL-------
NIEQMHWN-GFSRE--------VSEQ-FRLGF--PIQW-------------EKYA------------------
-----------------------------------------NSNMKQA-NEHTLSPAKST-------------
--------------------EYCVEK----------------------------------------------
FLRSSF-------------------------------------------------------------------
------------ANSMEHTLT-------------------EFDF-----------------------------
-------------------------------------RTSKESTGNT----------DGPGLPNYVK---PR-
----------IQEPSGNSGGYDISVSNM-------------------AASEGLCNDRM---------------
GMPDESFED------------------------------PG----PGETCNGQASRADNSH------------
-------EDIETDASGQ--------------------------------------------------------
-------------------------------------------------------------------------
-------------------------------------------------------------------------
-------------------------------------------------------------------------
-------------------------------------------------------------------------
-----------------------------------------------RIVTH---------------------
-------------------------------------------------------------------------
SMDSTLVNNDIYKI-----------------------------------------------------------
-------------------------------------------------------------------------
-------------------------------------------------------------------------
-------------------------------------------------------------------------
EE--------------------------------------------------------------
EHGSSKLGNSS-----------VCPG-----------------------------
TEHVLEALNQGASPENGSVQCS---------------------------------------------------
-------------------------------------------------------------------------
-----------------------------------------------------RRLRSGKVY-----------
-------------------------------------GM----------------------------------
-----SNGA----------------------------------------------------------------
-----------------------------SLKR----------------------------------------
-------------------------------------------------------------------------
-------------------------------------------------------------------------
-------------------------------------------------------------------------
---------------------------------------------------R---------------------
------------------------------------------------------------------YSKRK--
---------------------------------------------------------TMQHETLCMK------
------------------------------------------------------------------------
VIPTEETT-----------------------------------------------------------------
-----------------PPAGPT------------------------CHK-KLL-------------------
------------------------LGGSDI-------------------------------------------
----------------------TKACY----------------------------------------------
-------------------------------------------------------------------------
-------------------------------------------------------------------------
-------------------------------------------------------------------------
-------------------------------------------------------------------------
-------------------------------------------------------------------------
-------------------------------------------------------------------------
-------------------------------------------------------------------------
```


>Vitis_vinifera_205s0124g00540.1

----MGKG--ESSNTKA------

---TIIS--------
-----------ASSFLRS---
---------------------------------------VTLHDWWLLK-TN-A------------NRLAVG-
--------------GFA-------------------------------T------------RE----------
-------------------------------RQ-GI-RVF---------------------------------
--------------------------------------SSGAIAKRHDATTLETADGITITIVGFL-------
NKSRTHQN-GFPSE--------VCKH-FLFGF--PYHW-------------EEYA------------------
---VQCFV---------------------------

-----------GESTK------------------------SGV----------------SKKPSGCEEF----
------------------------------------NLPSTSSENN---------------------------
------------------LLPASLDEL--
---PVTRV-----------------
------RDLLMSTLGD---

---SKTLT-------
-------NSIFS----------------------------------DIVGRV---------------------
---------------------------------------SN--------------------------------
LSNMKGNSPTTV---
-----------DTELDRTPRN--
----HKKAEVEKFEDDN--------------------------------SILDVSDMRT--------------
----
EE----------C-------------------KKNICQSSGV---------------------MNDSTPSRR-
--VSTRTMTRLK-
NLKSQLEWNLS--

-----------------------------------SSTSKKQKT-----------------------------
--------------------REN-------------------------------------SEKRL--------
-----------------------------L---
------------TNSSNDV--

-----------------------------------LRRSSGNDFKHARKDMKTNGPIK---------------
--TMDSDLD--------------------------
---KFIKHKMAKV----------

---------------------------EQKRTDNC--------------------------------------
---------------------------------------VSMDTMDIRK-----------------
EECQKYFCRSE-VGM---------------HALSP--------------------------------------
-----------------------------DRGVATRSMARLKN------------------------------
-------------VKNNP---

>Vitis_vinifera_208s0007g00050.1

----MASD--SDPS---------
------------------------------------INS---------------------------------
ND----------------------------------SS-----------------------------SYF---
----

----------------QQT--
--VCLQDWWLIK-SKKG--------FEG-
KRLAVA---------------GLS-------------------------------S-----------KE---
--------------------------------------QR-AV-RVF--------------------------
----
SSAPIIKRYDVFTLETADGITVIIKGFI-------NKLRTNEN-GFPSE--------VFNH-FLFGF--
PPHW-------------EEYA--
-------EKCFEGGSTSGF-VSR--------------SNPGVVS-----------------------------
----
--------------ISTHDLDKLMMDSEKTNQCKKAINEAR--HENSEDVVEMKR------------------
------KNN----------------ERSSPF--
--EACENQDV-G----------NLNRS-------------SLT--------------ASAQSGKKM-------
---DISE------------------------

--DIPCQ------------SEGQVSKC----
QSRKSNRKW--
--QEGQKAEI-------GLKTK---
------------RKSFI------------SVPVGF---------------DHVNNT-----------------
VGNISR----------EIGGE--SI--

--SNLSDVGPQ----------
---EHH-N-ITVEKLDDEC----------------
--------------------LKDGSQ--------------KNSE---------------------FP------
-----PA-YTNAKCK---QNHENIMIQ--
-----------------------AKIQSKNT-NESVRP-----------------------------------
----------------------STRPGQANQ-KIDLK-TP---------------------------------
----

---VGERA-------

---SSSGKKTKR--
KLDYNIQ--
---------------VTHPSIK---
--ERT-E------
--VSI---------------------------
VSPELLSL--------------------------KRSR-SG----------------RLLLPSLDF-W-----
--RNQKAVYDA---DRRITGIQE--------
---VL-HV----------------------------------VEP-SRA------------------------
--------NRPLH---
LVGRE--

>Zea_mays_001145421.1

----MPSQ--PQPSSIG------
------------------------------GTSQTP-------------------------------------
--APF----

---QTAPV-----------
-------------------------FTGG---------------SPRTLAT----------------------
--LSAQSVPR-------
-----------VT--RRS---
---------------------------------------IALVDWCLER-VEGEE-----------GKIRVA-
--------------GTTYTPQTS----------------PQTRREASSSK-----------GS----------
-------------------------------RKVAG-RVF---------------------------------
--------------------------------------RSSAIVRRHDHFRIMSEDGYLIRIGCLL-------
NIPKTRNN-GFSEE--------VCEC-FEFGF--PIQW-------------HRLV------------------
---NPKMVPD-NKHALSPSETTASAPS--------
-----------------HSVGYYMEK--
FLSDSF---
------------SNSNRYSFT-------------------ENDS-----------------------------
-------------------------------------YTSVVCTGSR----------NGLTTQT---------
----------------LSNLPDDNAGNI-------------------TVSWGLYGLGM---------------
DMSEKPWTPPA------------------------------------EACNNRQESDQH--------------
-------ESMQIDACKQ--

---ELVNR---------------------

SMSSVSVKQSISSI---

-----SPNSKVDGN------------------RI-------------VPSKIMSVVNESY-----------
RSTVGCGQ---AEEDVGIQQ-------------
--EK-------KHSCS----------------SEHGMVTLSINC----------------
TSSQLGAPGIPKFGKDS-----------VNLW-----------------------------
TTDALELSTEGMTPKI--GADST----------

--DRRLRSGKILG----------

----MPSG--------------G--
-----------------------------LMNR--

---G---------------------
---HKLK--
---KIKQEASSKQ------

MV-NQGAT---
-----------------CTVDLT------------------------SHE-NDF-------------------
-------------------------SAAEI---------------------------VVEEKLESHSSC----
------------------LKGR-GG--------------------P---------------------------
----

--AKGKRKRER--------------

--------------------------------------W----------------------------------
----

>Zostera_marina_30g00570.1
--MVYDSPI--------------

----MVKNATFFC---KGAADSPQT--------

--ST-NKM-

---TPS-----------

---------------RRS---
---------------------------------------VFLSDWWLVD-TKDN--------DGG-RKLAVG-
--------------GKN---Y----------
-------------------------------NRLNMVREF---------------------------------
--------------------------------------TSAFICKRVDAFTLETIDGFTIVIDGPV-------
NQNRSKLN-GFSVE--------VCSQ-FLMGF--PVNW-------------ETYT----VDFF----------
--P--------------------------------------KESISE------------F-------------
----

-----------ANSSG------------------------GNI----------------LHRPSS--------
IP------------Q---DGSPAS-----------
----------------KIAAFIKLAEY--
--DIYQRSRKRER----------------
------ENCDTCIGNT---

--VKIELNDG---------SVSPTSELNFP-----
-----RSTRLMK------------------IEKIETPTGN------EIHLQ----------------------
-------------------------------------TPLDSP----------------------------
VSCDKLQFSSEPKRK--
RGRPSKTGNEIH-----------LQTSLDSPVSCD-----------------------KL-------------
-QF-------------SSEPKRKRG---------------------------------
RPSKAGNEIHLQNPL--
--------------------------DS-------PVSC----------------------------------
----
-DKLQFFSKPKRKRG--

--RPSKASKLS------------
------------------------------------NHS----------------------------------
---SKEGC-------------GQ--
----------------------------KFKK---
--------AK---

--ELDSPDQGQF-------

---TRD-------------------------
-----------------------RDPSL-----------------------F---SSPELLNL----------
----------------RRSR-SG----------------RLLVPMLDV----------QQRLIHDG-------
--------------------------------------DGSIIGIMKS-------------------------
---------------Y------QNEDP--
--PSSSRT-----------------

-----------------GN-------------------------Q----------------------------
----

>Aethionema_arabicum_maker9882.1


```
-------------------------------------------------------------------------
-------------------------------------------------------------------------
----------------------------------------MLKDWWLVK-CTNE--------FEG-KRFGVA-
--------------GIEIT----------------------------AS------------AE----------
-------------------------------RR-AM-RVF---------------------------------
--------------------------------------TSSPIIKAYDVFTLETSDGNCIIIRGFL-------
NKERVIQS-GFNPEVSIELRIRISRH-FVFGF--PPCW-------------EQIC------------------
-----------------------------------------NDCFEGR------DINTE--------------
--------------------TLKID------------------------------------------------
--------------------------------------------------------------KAFCSVLSPC-
-----------KNKK-------------------------TNI----------------EDTPVQ---------
------------------------------------NKDKSTVSEM----------K----------------
-----------------------TAEF-------------------NGKDTRS--------------------
------NGSR------------------------------------TGDKQIVGR------------------
------KSQRLQSK-----------------------------------------------------------
-------------------------------------------------------------------------
-------------------SRRVE-------------------------------------------------
-------------------------------------------IP----------------------------
------------------------------------------TTNGD------------HSSEGF--------
-------KE-SK------------------RGVEEK-HES------VAIEN--DVTSPV--------------
--------------------------------------GTD-S------------------------------
VHKVTSKSAT---------------------------------------------------------------
--------------------------------------------------------K-------------
RLQS-K-ASRVVI------------------------------------------------------------
--------------------------------PTTN-GEH-TSEGFEE----SKRGVEEK-------------
--HE----------SVANEN---------------DVT------------------SPV---------GTD--
--------------------------------------------------SVHKVTSKSATRES-S-----
PS--------------------------------------------------------
TEQQKGKRKVTSAFPRA--------------------------------------------------------
-------------------------------LLKDLDKR-----SKSRKKGKS--------------------
-------------------------------------------------------------------------
-------------------------------------------------------------------------
---------------------AVKS-DI---------------------------------------------
-------------------------------------NVEA--------------------------------
-------------------------------------------------------------------------
-------------------------------------------------------------------------
-------------------------------------------------------------------------
-------------------------------------------------SAEENLSMGKTKR-----------
-------------------------------------------------KIAFDME-----------------
----------------------------------------------------------------VTPVKEA--
-------------------------------------------------------------------------
--------------------------------------KKQ-D------------------------------
------------------NNL---------------------------VSPESVGQ-----------------
---------RRSR-SG----------------RVLVSSLEF-W-------RNQIPVYDM--------------
-------------------------------DRKLIRVQE-----------GH-DV-----------------
-----------------TTP-SKG-------------------------------------------------
---------------------------------------------FS--------------------------
-------------------------------------------------------------------------
-------------------------------------------------------------------------
-------------------------------------------------------------------------
-------------------------------------------------------------------------
-------------------------------------------------------------------------
-------------------------------------------------------------------------
-------------------------------------------------------------------------
-------------------------------------------------------------------------
-------------------------------------------------------------------------
-------------------------------------------------------------------------
-------------------------------------------------------------------------
-------------------------------------------------------------------------
-----------------------------------------------------------
```

```
>Arabidopsis_halleri_0330s0008.1.p
-------------------------------------------------------------------------
-------------------------------------------------------------------------
-------------------------------------------------------------------------
----MAE-------------------------------------------------------PNP--------
-------------------------------------DD---------------------------------
D---G-------------------------------SK-----------------------------SYF---
-------------------------------------------------------------------------
-------------------------------------------------------------------------
-------------------------------------------------------------------------
-------------------------------------------------------------------------
----------------QKT------------------------------------------------------
----------------------------------------VVLRDWWLIQ-CPKE--------FEG-
KRFGVA---------------GFE------------------------------DS------------VE---
--------------------------------------TR-AM-RVF--------------------------
---------------------------------------------
KSSPIIRALDVFTLLASDGIYITLRGFL-------NKERVVNN-GFTPE--------ISRE-FIFGF--
PPCW-------------ERFC----------------------------------------------------
-------SSCFVGD-SFGT-DINTV----------------------------------PSTID---------
-------------------------------------------------------------------------
----------------------------KAFPPILSPC------------KYSN-------------------
------GNV----------------EDNPAE------------------------------------------
--SRDKSSVTET----------D---------------------------------------IAEI-------
------------NDKDG------------------------------SR------------------------
------------ARAKKTARR------------------------KSLHLS----------------------
----EEEE--------------------------------------------RKLESSYVQNTTN--------
---EGDHGSECLSKAK------------------------------------------SGDVEKD----
GCEAI--------------------------------------------------------------------
-------------------------------------------------NNEDNEWKLDGS----ELQNR---
------------TNDGV------------HGSEGL---------------IK-AK------------------
SSDVEK-DEC------EVIDN--NVKSPAVGCG----------------------------------------
----IKYTDAD-N------------------------------VDKVTSASATGE------------------
------------------------------------SLTPEQRK----GV----L-GTTASPQC---------
-------LLKDL-------DKSSKSEK-------------KGIS-K-K-------------------------
-------------------------------------------------------------------------
-------------------------------------------------------------------------
-------------------------------------------------------------------------
-----------------------SKNATKES-L-----PS---------------------------------
------------------------EQRKGRVKVTNASQDP---------------------------------
------------------------------------------------------LSKDLINS-----
SKPGKKGKS----------------------------------------------------------------
-------------------------------------------------------------------------
-------------------------------------------------------------------------
-------------------------------------------------------------------------
-------------------------------------------------------------------------
-------------------------------------------------------------------------
-------------------------------------------------------------------------
-------------------------------------------------------------------------
-------------------------------------------------------------------------
-------------------------------------------------------------------------
-------------------------------------------------------------------------
-------------------------------------------------------------------------
-------------------------------------------------------------------------
-------------------------------------------------------------------------
-------------------------------------------------------------------------
-------------------------------------------------------------------------
-------------------------------------------------------------------------
-------------------------------------------------------------------------
-------------------------------------------------------------------------
-------------------------------------------------------------------------
```


>Arabidopsis_halleri_39738s0001.1.p

---TLEAIDGITISINGFI-------
NRSRSLEN-GVSNE--------VCNR-FRLGF--PHDW------------EDYD------------------
--EE------------------------------

-----------EEEKK-----------------------NVV----------------D-------------
----
---------------------VSFDDI---
--PVNRY-----------------
------QD-LYCL---

--EGCLK-------
-------DKILD---------------------------------DVV------------------------
---------------------------------------G-------------------------------
SLRDLV--

--CQ-KSDKECEKS-----------------------------------RIGGD------------------
----
DG------------------------------E-------------------------------SL------
--VVGVKTRGMLRRR-EEC-E-A---
--SIGKRVATVS------

------------GERAVT----------RTDME---------------------------------------
----
----------EKV--------EYT--
LKIIDEDGDTFAKRAEMYYRKRPEIVNFVEEAFRSYRALAERYDHLSR-ELQSANRTIA-------------
-TAFPEHVQ----------------------FPLE---DDSD-
ENEDYEGNPRKPPKHLHLIPKGINIPEVPDIPKKKDFRSQSMMLSRKGPAGLK-------RTVSSA----
QAKREAAIVRSGLSKEEGLEEIDMLQKGILALQTEKEFVRSSYEQSYERYWDLENEVTEMQKSVCNLQDEFGL
GASIDDSDARTLMASTALSSCKDTLAKLEEKQRKSV--EEAEIEKGRITTAK----ERFYA--------
LRNKFEKPENDVLDEVIRTDEEEEKEA-------
DDVVQESSYESEREDSNENLTVVKLAEKIDDLVHRVVSLETNASSHTALVKT------LR--

SETDELHEHIRGLEEDKAALVSDSTVMKQR-------ITVLEEELRNVKKLFQ----
KVEDQNKNLQNQFKVANRTVDDLSGKIQDVKMDEDVEGAGIFQELAVVSGSEDSRDDLKSISTETEMRSNVEK
TKKDVIVVKESEDSERAQEEKSEMKDSFALSETASTCFGTEAEDLVTEDEDEETPNWRQLLPDGMEDREKVLL
DEYTSVLRDYREVKRKLGDVEKKNREGFFELALQLRELKNAVAYKDVEIQSLRQKLSSPGKDSPHQVEGNNQ-
-------------LEHEQVHHE-SV--
SISPTSNFSVSTTPHHQLGDMKRTPGRTKSTEVRVKFADVDDSPRTKIPTVEDKVRA-
DIDAVLEENLEFWLRFSTSVHQIQKYQTTVQDLKSELSKLRIESKQQHESPR-SS-SNTAVASEAKPIYRHL-
-REIRTELQL-WL------ENSAVLKDE-----
LQGRYASLANIQEEIARVTAQSGGNKVSDSEISGYQAAKFHGEILNMKQENKRVSTELQSGL-DRVRALK---
---TEVEKILSKLEEDLGISSATEARTTP-SKST-----------------------------SSGRPRI---
------------------------------------PLRSFLFG----VKLKKNRQQKQSSSS----------
----

--------------------------------LFSCV------------------------------------
----
---------------------------SPS---
--PGLQKQS--------------------

---------------------SYNRPP---
GKL---P-----------E
>Arabidopsis_lyrata_2G18170.t1

----MTTTR-------------------SKFQSLSARR----------------------FTP----------
--------------------------------LPEPNPS----------------------------------
-P--------------------------------RTF-----------------------------SKT----
----
---LP------------
--EPNSS-----------PGTNGTFR-----
----------------TPFPL--------------------------SLI-----------TPI---------
------------KT-LKS---
---------------------------------------ITLSDWWLKK-KS--------------KGLSIT-
--------------GFE-------------------------------S------------NG----------
-------------------------------GS-GV-RLF---------------------------------
--------------------------------------SSGTISKRHESTTLEAIDGITISINGFI-------
NRSRSLEN-GVSNE--------VCNR-FRLGF--PYDW-------------EDYN------------------
---VEE-----------------------------

-----------EEEKK------------------------NVV----------------D-------------
----
---------------------ISFDDI--
---PVNRY-----------------
------QD-LYCL--

---EGCLK-------
-------DKILD----------------------------------DVV------------------------
--S-------------------------------
SLRDLV---

--CQ-IFDKECEKS------------------------------------RIG--------------------
---------------------------------GD--------------------------------------
DG-------------------------------E-------------------------------SLVSR---

```
-------------------------------------------------VVGVKTRGMLRRR-EEY-E-A---
---------------------------------------------------------SIGKGVATIS------
-------------------------------------------------------------------------
------------GERAVT-----------TSKKK---------------------------------------
----------KR-------------------------------------------------------------
-------------------------------------------------------------------------
-------------------------------------------------------------------------
-------------------------------------------------------------------------
-------------------------------------------------------------------------
-------------------------------------------------------------------------
-------------------------------------------------------------------------
-------------------------------------------------------------------------
-------------------------------------------------------------------------
-------------------------------------------------------------------------
-------------------------------------------------------------------------
-------------------------------------------------------------------------
-------------------------------------------------------------------------
-------------------------------------------------------------------------
-------------------------------------------------------------------------
-------------------------------------------------------------------------
-------------------------------------------------------------------------
-------------------------------------------------------------------------
-------------------------------------------------------------------------
-------------------------------------------------------------------------
-------------------------------------------------------------------------
-------------------------------------------------------------------------
-------------------------------------------------------------------------
-------------------------------------------------------------------------
-------------------------------------------------------------------------
-------------------------------------------------------------------------
-------------------------------------------------------------------------
-------------------------------------------------------------------------
-------------------------------------------------------------------------
----------------------------------------
>Arabidopsis_lyrata_6G11780.t1
-------------------------------------------------------------------------
-------------------------------------------------------------------------
-------------------------------------------------------------------------
----MAE-------------------------------------------------------PNP--------
-------------------------------------DD---------------------------------
D---G-------------------------------SK-----------------------------SYF---
-------------------------------------------------------------------------
-------------------------------------------------------------------------
-------------------------------------------------------------------------
-------------------------------------------------------------------------
----------------QKT------------------------------------------------------
----------------------------------------VVLRDWWLVK-CPKE--------FDG-
KRFGVA---------------GFE------------------------------DS------------VE---
--------------------------------------TR-AM-RVF--------------------------
---------------------------------------------
KSSPIIRALDVFTLLASDGIYITLRGFL-------NKERVVNN-GFTPE--------ISRE-FIFGF--
PPCW-------------ERFC----------------------------------------------------
-------NSCFLGD-SCGT-DINTV----------------------------------PSTID---------
-------------------------------------------------------------------------
----------------------------KACPPILSPC------------KYSN-------------------
------GNV----------------EDYPSE------------------------------------------
--SRDKSSVTET----------D---------------------------------------ITEI-------
------------NDKDG------------------------------SR------------------------
------------ARAKKTARR------------------------KSLHLS----------------------
----EEEE--------------------------------------------RKLESSNVQNTT---------
```

```
---------------K------------------------------------------SGDVEKD----
GCVAI--------------------------------------------------------------------
-------------------------------------------------NNEDNEWKLDGS----ELQNC---
------------TNDGV------------HGSERL---------------IK-AK------------------
SSDVEK-DEC------EVIDN--NVISPAVGCG----------------------------------------
----IKYTGAD-N------------------------------VDKVTSASATGE------------------
------------------------------------SLTPEQRK----GV----L-GTTASPQC---------
-------LLKDL-------DKSSKSEK-------------KGIS-K-K-------------------------
-------------------------------------------------------------------------
-------------------------------------------------------------------------
-------------------------------------------------------------------------
-----------------------SKNATKES-L-----PS---------------------------------
------------------------EQRKGRVKVTKASQDP---------------------------------
------------------------------------------------------LSKDLINS-----
SKPGKKGKS-------------------------------------------------RKS------------
-------------------------EK----------------------------------------------
--------------------------------------------------TLQS-DS----------------
------------------------------------------------------------------
NVVEPMN------------------------------------------------------------------
--------------QSRSQ------------------------------------------------------
-------------------------------------------------------------------------
-------------------------------------------------------------------------
----------VEEAEENLSWEKIKR------------------------------------------------
------------KIDFDVE------------------------------------------------------
---------------------------VTPEKEV---------------------------------------
-------------------------------------------------------------------------
-KKQ-K------------------------------------------------TNA----------------
-----------ASTDSVGQ--------------------------KRSR-SG----------------
RVLVSSLEF-W-------RNQIPVYDM---------------------------------------------
DRNLIQVKD-----------GS-ET-N--------------------------------STP-SKG-------
-----------------------KGS-----------------------------------------------
--------------NSQK-------------------------------------------------------
-------------------------------------------------------------------------
-------------------------------------------------------------------------
-------------------------------------------------------------------------
-------------------------------------------------------------------------
-------------------------------------------------------------------------
-------------------------------------------------------------------------
---------PRS-------------------------------------------------------------
-------------------------------------------------------------------------
-------------------------------------------------------------------------
-------------------------------------------------------------------------
-------------------------------------------------------------------------
-------------------------------------------------------------------------
----------------------------
>Arabidopsis_thaliana_1G58210_new
-------------------------------------------------------------------------
-------------------------------------------------------------------------
-------------------------------------------------------------------------
----MTTTRA-----------------KSKFQSLSACR----------------------FTP----------
--------------------------------LPEPNTS----------------------------------
-P--------------------------------STY-----------------------------SKT----
-------------------------------------------------------------------------
-----------------------------------------------------------LP------------
--------------------------------------------KPNSS-----------PGTDGTFP-----
----------------TPFPL--------------------------AVI-----------TPI---------
------------KT-LKS-------------------------------------------------------
---------------------------------------VTLSDWWLTK-KG--------------KDLCIK-
--------------GFE-------------------------------S------------NG----------
-------------------------------AS-GV-RLF---------------------------------
```

```
--------------------------------------SSGTISKRHESTTLEAIDGITISINGFI-------
NRSRCLEN-GISIE--------VCNR-FRLGF--PYDW-------------EDYN------------------
-----------------------------------------EEE-----------------------------
-------------------------------------------------------------------------
-------------------------------------------------------------------------
-----------EEKKK------------------------KNV----------------D-------------
-------------------------------------------------------------------------
---------------------ISFDDI----------------------------------------------
---------------------------------------------------PVNRY-----------------
------QD-LYSL------------------------------------------------------------
-------------------------------------------------------------------------
-------------------------------------------------------------------------
-------------------------------------------------------------------------
-------------------------------------------------------------EGCLK-------
-------DKILD----------------------------------DVV------------------------
----------------------------------------G-------------------------------
SLRDLV-------------------------------------------------------------------
-------------------------------------------------------------------------
--CQ-KSDKACEKS------------------------------------RVGDVDD----------------
------------------------------DDDDD--------------------------------------
DD-------------------------------K-------------------------------SLVSR---
-------------------------------------------------VVGVKTRGMLRRR-EEY-E-A---
---------------------------------------------------------SIGKRVATMS------
-------------------------------------------------------------------------
------------GKRVVT-----------VSKKK---------------------------------------
----------NRR------------------------------------------------------------
-------------------------------------------------------------------------
-------------------------------------------------------------------------
-------------------------------------------------------------------------
----------------------------------RS-------------------------------------
-------------------------------------------------------------------------
-------------------------------------------------------------------------
-------------------------------------------------------------------------
-------------------------------------------------------------------------
-------------------------------------FG----------------------------------
-------------------------------------------------------------------------
-------------------------------------------------------------------------
-------------------------------------------------------------------------
-----------------------W-------------------------------------------------
-------------------------------------------------------------------------
-------------------------------------------------------------------------
-------------------------------------------------------------------------
-------------------------------------------------------------------------
-------------------------------------------------------------------------
-------------------------------------------------------------------------
-------------------------------------------------------------------------
-------------------------------------------------------------------------
-------------------------------------------------------------------------
-------------------------------------------------------------------------
-------------------------------------------------------------------------
-------------------------------------------------------------------------
-------------------------------------------------------------------------
-------------------------------------------------------------------------
-------------------------------------------------------------------------
----------------------------------------
>Arabidopsis_thaliana_5G02520.1
-------------------------------------------------------------------------
-------------------------------------------------------------------------
-------------------------------------------------------------------------
----MTE-------------------------------------------------------PNL--------
```

-------------------------------------DE---------------------------------
D---G-------------------------------SK-----------------------------SSF---
----

----------------QKT--
--VVLRDWWLIK-CPKE--------FEG-
KQFGVA---------------GFE------------------------------ES------------VE---
--------------------------------------TR-AM-RVF--------------------------
----
TSSPITKALDVFTLLASDGIYITLRGFL-------NKERVLKN-GFNPE--------ISRE-FIFGF--
PPCW-------------ERVC--
-------NSCFEGD-SFGT-DVNTV----------------------------------PSTIE---------
----
----------------------------KACPPILSPC------------KYSN-------------------
------RNL----------------KDNPAE--
--SREKSNVTET----------D---------------------------------------IAEI-------
------------NDKGG------------------------------SG------------------------
------------ARDIKTARR------------------------RSLHL-----------------------
-----QIK--RILESSKVRKTAN--------
---DGDHGSEFLNTAK--RGDVERD----
GCEVI--
---NNEDSEWKLDES----EVQNL---
------------CNDGD------------NGSEGF---------------IK-AK------------------
SSDVEK-DKS------EAIDN--DVISPAVGSG--
----IKHTGAD-N------------------------------VDKVTSASATGE------------------
------------------------------------SLTSEQQN----GL----L-VTTASPHS---------
-------LLKDL-------AKSSKPEK-------------KGIS-K-KSGKILRSDDNVVDPMNY--------
----------------SGTKVKSAEN--------------KRKI--------------------DASKLQ-
SPTSNVAEH-SKEGLNN----AKSNDVEK---------------DV----------CVAINN-----------
----EVI------------------SPVKGFGK-RLSGTD---------------------------------
--------------------VERLTSKNATKES-L-----TS-------------------------------
--------------------------VQRKGRVKVSKAFQDP-------------------------------
--LS---------------
-KGKS---KKS----------------
---------------------EK--
--TLQS-NS--------------------
--NVVEPMN----

---HFRSE---

AEEAEENLSWEKIKR--
--KIDFDVE--
-----------------VTPEKKV---
--KQQ-K----
--TNA--------------------------
-ASTDSLGQ--------------------------KRSR-SG----------------RVLVSSLEF-W----
---RNQIPVYDM---DRNLIQVKD-------
----GS-ET-N--------------------------------SAP-SKG-----------------------
-------KGS---
DSRK---

RRNLKIK--

>Arabis_alpina_G506860.h1.t1

----MVEH--------------------R--------------------------------APNL--------
-------------------------------------DG---------------------------------
DSENR-------------------------------SS-----------------------------SSF---
----

----------------QRT--
--VILRDWWLVK-CSKE--------VEG-
KRFGVA---------------GTEITA--------------------------S-V------------VE---
--------------------------------------KR-AM-RVF--------------------------
----
TSSPIIKAFDIFTLQASDGICITLQGFL-------DKKRVVES-GFIPE--------ISRE-FIFGF--
PPCW-------------EQIC--
-------NKCYGGC-PLGT-DFKSV----------------------------------HSIKE---------
----
----------------------------EACSPILSPC------------KNTK-------------------
------GNV----------------ENSPAE--
--SRDESRVSEK----------N---------------------------------------MG---------
----
----------------QQARR------------------------KSPRRQ--PG------------------
-GKRAEDV--RKLEL----------------

--S----KVKNM-------
--------INDGD------------HGSEDL---------------GK-AK------------------
RSVVEK-DEC------EAIDN--EVASLDDGCG--
----KRHTRAY-S------------------------------VDKTTS------------------------
----

------------------------KN---
--------GREGLDK----GKSSDVIE---------------NE----------CEAIKD-------------
-REVI------------------LPEDGCGR-KHTGAD-----------------------------------
-----------------NVDKLTSMSAGGES-L-----TS---------------------------------
------------------------EQRKGKLKVTKTSLPS---------------------------------
--LSKDLNNS-----
KKPGKKERS---KKS------------
-------------------------EK--
--TLKG-DC----------------

NVVEPLS--
--------------HSGSK--

----------VKEAEETMSWEKAKR--
------------KIDFDLE--
---------------------------VTPVKKP---------------------------------------
----
-KKQ-N--TDA----------------
-----------DSTGSVGQ--------------------------KRSR-SG----------------

RKLVSPLEF-W-------RNQVPVYDV---
GRTLIQVNV-----------GH-ET-N--------------------------------STP-SKG-------
-----------------------KGS---
--------------DSRK---

---------ARR---

>Arabis_alpina_G656800.h1.t1

----MTNK--------------------SKSQSLSTRR----------------------TSPRI--------
----------------FS------------RSLPEPNSS----------------------------------
-P--------------------------------RTL-----------------------------SRA----
----
---LP------------
--KRNFS-----------PGTDGIPR-----
----------------TPFSL--------------------------GAI-----------TPV---------
-----------IGT-LKS---
---------------------------------------VSLSDWWLTK-KT-----------KE-NGLGIE-
--------------GFE-------------------------------S------------KS----------
-------------------------------GS-VARRLF---------------------------------
--------------------------------------SSGAITKRHDSTNLETFDGITVCISGFI-------
NQSRTLQN-GISLD--------VCNR-FLLGF--PYNW----------------K------------------
---EES-----------------------------

-----------VETKK------------------------A------------------D-------------
----
------------------LG-ISFDDI--
---PVSRL-----------------
------QDLLVTTC---

---STCLK-------
-------SKILD----------------------------------HVV------------------------
--D-------------------------------
SLRDLTCPTNN--

--TQ-KSDKKCEEK---

--LLPM---
---VVGVKTRGMLRQR-EDN-E-AS--
---SSSIGKLVL---------

-----------------------------SKSKK---------------------------------------
----------KR---

>Barbarea_vulgaris-Contig192-snap0.61

----MSDH--------------------E--------------------------------DPNV--------
-------------------------------------DG---------------------------------
DGANS-------------------------------YS-----------------------------SSF---
----

----------------QRT--
--VVLRDWWLIK-CSNE--------FEG-
KRFGVA---------------GTE------------------------------AT------------VE---
--------------------------------------SR-AM-RIF--------------------------
----
TSSPIIRALDVFTLKASDGLCITLRGFL-------NKERLVKN-GFKPE--------ICRE-FIFGF--
PPCW-------------ERIC--
-------NDWFQGD----S-DINTI----------------------------------D-------------
----
----------------------------KASSPILSSC------------KYSK-------------------
------GTL----------------EDNPAE--
--SRD------T----------N---------------------------------------TAEI-------
------------DNKDG------------------------------SR------------------------
------------AR-----DQ------------------------KTARRKAKSG------------------
-AKSAEDE--RKLESSKVQNTTN--------
---DGDLVSEGLNKAK--SSDVEKD----
ECEAI--
---NNEENY----DS----KDQNC---
------------TSVED------------HGSEGV---------------DK-AK------------------
SFDVEE-DEC------GAIND--EVISPADGCG--
----RKQTGAD-N------------------------------VDKVTSTTATGE------------------
------------------------------------SLTSEQRK----GE----LKVTTASPLS---------

```
-------LFKDF-------DKSSKPGK-------------KRIS-K-KSKKTLESDGNAVEPKNH--------
----------------SRTKVKSAEK--------------KRNL--------------------DVSKIQ-
GPTTNDGDH-GSECLNK----AKSNDVEK---------------DE----------CVAIND-----------
----EVI------------------LPVDGCGK-KHSGTD---------------------------------
-------------------GVEKLTSKNATRES-L-----TS-------------------------------
--------------------------EQRKGKRKGTKTCLHS-------------------------------
--------------------------------------------------------LSKDLDKS-----
SKPGKNRKS-------------------------------------------------KKS------------
-------------------------EK----------------------------------------------
--------------------------------------------------TLKG-DS----------------
------------------------------------------------------------------
NVVEPMN------------------------------------------------------------------
--------------HLGC-------------------------------------------------------
-------------------------------------------------------------------------
-------------------------------------------------------------------------
------------EVEEDLSWGKTKR------------------------------------------------
------------KIDFDVE------------------------------------------------------
---------------------------VTPTKES---------------------------------------
-------------------------------------------------------------------------
-KKQ-K------------------------------------------------TIV----------------
-----------VSTDSVGE--------------------------KRSR-SG----------------
RLLVSSLEF-W-------RNQIPVYDM---------------------------------------------
DRNLIQVKE-----------GS-ET-N--------------------------------SAP-SKE-------
-----------------------RASL--YIYLCARLL------------LDRKRIEIS--------------
------------KAKKMK-------------------------------------------------------
-------------------------------------------------------------------------
-------------------------------------------------------------------------
-------------------------------------------------------------------------
-------------------------------------------------------------------------
-------------------------------------------------------------------------
-------------------------------------------------------------------------
---------IK--------------------------------------------------------------
-------------------------------------------------------------------------
-------------------------------------------------------------------------
-------------------------------------------------------------------------
-------------------------------------------------------------------------
-------------------------------------------------------------------------
----------------------------
>Barbarea_vulgaris-Contig491-snap0.32
-------------------------------------------------------------------------
-------------------------------------------------------------------------
-------------------------------------------------------------------------
--MATATK--------------------SKLQSLSVHR----------------------SSPRT--------
----------------RS------------KSVPESNPS----------------------------------
-P--------------------------------LTF-----------------------------SRA----
-------------------------------------------------------------------------
-----------------------------------------------------------SM------------
--------------------------------------------KPNSS-----------PGTNSIPP-----
---------------RTPFSL--------------------------GAI-----------TP----------
-----------IGN-LKS-------------------------------------------------------
---------------------------------------VTLSDWWLIK-KG-----------KE-KNLCIS-
--------------GFE-------------------------------S------------KG----------
-------------------------------GS-EV-RLF---------------------------------
--------------------------------------SSGTITKRHDSITLEAVDGITICISGFI-------
NRSRSLQN-GVSNE--------VCNR-FLLGF--PYNW----------------N------------------
-----------------------------------------EE---E--------------------------
-------------------------------------------------------------------------
-------------------------------------------------------------------------
-----------EEDKK------------------------KKV----------------E-------------
-------------------------------------------------------------------------
```

---------------------ILLDDI--
---PVNRL-----------------
------QD-LCFV--

---EGCVK-------
-------DKILD----------------------------------DVV------------------------
--S-------------------------------
SLRDLV---

--CP-KSDKKCEKS------------------------------------RIGGDESL---------------
--------------------------------DSDDVVDSVRGLVC---SKSDKGCEK----------
FRIGGDD-------------------------------E-------------------------------
SLVSK--VVGVKTRGMLRRR-
QDE-D--
SIGKRRA--
--------------------------------ASNAYSWWWASHIRTKQ------------------------
--------------------------SKW-------------------------------------
LEHNLQDME------------------------EKV--------EYT--
LKIIDEDGDTFAKRAEMYYRKRPEIVNFVEEAFRSYRALAERYDHLSR-ELQSANRTIA--------------
-TAFPEHVQ-----------------------FPLE---DDSD---EDYEGKPHK---
HLHLIPKGTNIPEVPEIPKKKDFRSQSMMLSRKGPGSLK--------GAVASA----
LAKREAAIVSSGLTKEEGLMEIDKLQKGILALQTEKEFVRSSYEESYERYWDLENEVAEMQKRVCSLQDEFGL
GASIDDSDARTLMASTALISCKDTLARLEEKQKQSV--EDAEIEKERIITAK----ERFDA---------
IRNRFEKPESDDHDDVIRTEDEEVEEADVEEVEEADVVQESSYESEREDSNENLTVVKLAEKIDDLVHRVVSL
ETNASSHTALVKT-------LR--TETDDLHEHIRGLEEEKASLISDSTDMKQR-------
ITVLEDELRNVRKLFQ----KVEDQNKNLQKQFKEANLTVDDLSGKLQDVKMDEDVEGGGIFQELPVVSGS--
--DDLKSFSKETE-
RSSVEERKNKAIVGKESEDDEGAQEEKPEMKDSFALSETASTCFGTEAEDLVTEDEDGETPNWRQLLPDGMED
REKVLLDEYTSVLRDYREVKRKLGDVEKKNREGFFELALQLRELKNAVAYKDVEIQTLRGKLDTPMKGSPHQV
EGNNQ--------------LEHDQGQRE-SV--
SISPTSNFSVATTPHHQGLDMKRTPGRAKTNEVRVKFADVDDSPRTNIPTVEDKVRA-
DIDAVLEENLEFWLRFSTSVHQIQKYQTTVQDLKSELSKLRIESKQHQESPRSSS-SNSAVASEAKPIYRHL-
-REIRTELQL-WL------ENSAVLKDE-----
LQGRYASLANIQEEIARVTAHSGGNKVSESEISGYQAAKFHGEILNMKQENKRVSTELQSGL-DRVRALK---
---TEAERILNKLEEDLGITSATEARATP-SKSS-----------------------------SSGRPRI---
------------------------------------PLRSFLFG----VKLKKNRQQKQSASS----------
----

--------------------------------LFSCV------------------------------------
----
---------------------------SPN---
--PGLQKQS--------------------

---------------------SYVKQP---
GKL---P----------E
>Boechera_retrofracta_RETT00022998

----MAEH--------------------R--------------------------------EPNL--------
-------------------------------------DD---------------------------------
D---G-------------------------------SK-----------------------------SSF---
----

----------------QKT--
--VVLRDWWLVK-CPKE--------FEG-
KRFGVA---------------GIE------------------------------DS------------GE---
--------------------------------------TR-AM-RVF--------------------------
----
TSSPIIKALDVFTLLASDGICITLRGFL-------NKERVVKN-GFTHE--------ISRE-FIFGF--
PPCW-------------ERIC--
-------YNCFGGGVSLGT-DTNTV----------------------------------PSTIA---------
----
----------------------------KASYPILSPC------------KNNK-------------------
------ENL----------------EDTSAK--
--ERDENTVPDM----------D---------------------------------------TAEI-------
-----------NNTKDG------------------------------SR------------------------
------------ARAKKTARR------------------------KSLHLQTKSG------------------
-GKSAEDE--RILESSKVHNSTN--------
---DGDHGSEGLHKGK--SGEVEKD----
ECEAI--
---NNEDNEWRLDES----KLQNMT--
------------TNDGD------------HGSEGL---------------DK-AK------------------
SGGVEK-DEC------EAINN--GVISPADGCG--
----RNHTAAD-N------------------------------VDKVTSTSTTPE------------------
------------------------------------SLTSEQRK----GE----LKVTTASPHS---------
-------LLKDL-------HKNSKPRK-------------KGKS-K-KSEKTLKNDGNVVERSNC--------
----------------SETKVKSAEN--------------KR-------------------------KPQ-
NPTTNDRDL-GKEGLNN----AKSDDVEI---------------DE----------CVAIND-----------
----EVI------------------SPVDGCGK-RHSGTD---------------------------------
-------------------GVEKLTSKNATIES-L-----TS-------------------------------
--------------------------EQRKGRVKETKTSLHS-------------------------------
--LSKDLNNS-----
SKPGKKGKS---KKS------------
-------------------------EK--
--TLKR-DW----------------
--HATE---

---------YNF---

---SWESEENLLWGNTKR---
-----KIDFDVE---
--------------------VTPDNKA--
---KKQ-K-
---TNA-----------------------
----TSTDSLGQ--------------------------KRSR-SG----------------RVLVSSLEF-W-
------RNQIPVYDV---ARNLIEVKD----
-------GH-ET-N--------------------------------SSP-SKG--------------------
----------KGS--
-DSRK--

---RRS-

>Boechera_retrofracta_RETT00031638

---MATTK--------------------SKSQSLSARR----------------------SSPRT--------
----------------RS------------KSLPEHNPS----------------------------------
-P--------------------------------RTH-----------------------------SGA----
----
---LP------------
--KPNSS-----------PVTNVILQ-----
----------------SPFSL--------------------------GAI-----------TP----------
-----------VRT-LKS---
---------------------------------------ITLSDWWLTK-KG-----------KEKKGLSIT-
--------------GFE-------------------------------S------------KG----------
-------------------------------GS-EV-RLF---------------------------------
--------------------------------------SSGTICKRHNSTTLEASDGITICISGFI-------
NRSRTLEN-GVSNE--------VCHR-FLLGF--PYNW-------------EDYN------------------
---EE------------------------------

-----------EEEEK------------------------KNV----------------DT------------
----
-----------------DFG-VSFDDV--
---PVSRL-----------------
------QD-LFSL--

---EGCLK-------
-------SKILD----------------------------------DVV------------------------
--G-------------------------------
SLRDLA---

--CS-KSDKECDKS------------------------------------RIGDD------------------
----
---SLVSR---
---VVGVKTRGMHRRR-EEY-E-A---
---SIGKRV----------

-----------------------------RH--

TKRCCREQRAMHIHGGGPATSVQSNPNGSNT-IFKV--------EYT--
LKIIDEDGDTFAKRAEMYYRKRPEIVNFVEEAFRSYRALAERYDHLSR-ELQSANRTIA--------------
-TAFPEHVQ-----------------------FPLE---DDSD-
ENEDYEGNPRKPPKDLHLIPKGSNIPEVPEIPMKKDFRSQSMMLSRKGPAGLK--------RTVSSA----
LAKREAAIVSSGLSKEEGLAEIDKLQKGILALQTEKEFVRSSYEQSYERYWDLESEVTEMQKRVCNLQDEFGL
GAAIDDNDARTLMASTALSSCKDTLAKLEEKQKQSV--EEAEIEKGRIKTAK----ERFDA---------
LRNKFEKSESDDHDEAIKTEVEEEE----------
GDVVQESSYESEREESNENLTVVKLAEKIDDLVHRVVSLETNASSHTALVKT-------LR--
SETDELHEHIRGLEEDKASLVSDSTDMKQR-------ITVLEDELRNVRKLFQ----
KVEDQNKNLQNQFKVANRTARDLSGKLQDVKMDEDVEGAGIFQELLVVSGSEDSRHDLKSILTETETRSSLEE
TKKDATVVRESENGGRSQEEKSEIKDSFALSETASTCFGTEAEDLVTEDEDEETPNWRQLLPDGMEDREKVLL
DEYTSVLRDYREVKRKLGEVEKKNREGFFELALQLRELKNAVAYKDVEIQSLRQKLNSPGKDSPHQVEGNNQ-
-------------LEHDQGQRE-SV--
SISPTSNFSVSTTPHHQGGDVKRTPGRTKSNEVRVKFADVDDSRRTKIPTVEDKVRA-
DIDAVLEENLEFWLRFSTSVHQIQKYQTTVQDLKSELSKLRIESKQQLESPR-SS-SNPAVASEAKPIYRHL-
-REIRTELQL-WL------ENSAVLEDE-----
LQGRYASLANIQEEIARVTAQSGGSKVSDSEISGYQAAKFHGEILNMKQENKRVSTELQSGL-DRVRALK---

```
---TEVERILSKLEEDLGISSATEARTTP-SKSS-----------------------------SSGRPRI---
------------------------------------PLRSFLFG----VKLKKHRQQKQSSSS----------
-------------------------------------------------------------------------
-------------------------------------------------------------------------
-------------------------------------------------------------------------
--------------------------------LFSCV------------------------------------
-------------------------------------------------------------------------
---------------------------SPS-------------------------------------------
----------------------------------------------PGLQKPS--------------------
-------------------------------------------------------------------------
-------------------------------------------------------------------------
-------------------------------------------------------------------------
-------------------------------------------------------------------------
---------------------SYNRPP---------------------------------------------
GKL-----------------------------------------------------P-----------E
>Boechera_stricta_30057s0098.1.p
-------------------------------------------------------------------------
-------------------------------------------------------------------------
-------------------------------------------------------------------------
---MATTK--------------------PKLQSLSARR----------------------SSPRT--------
----------------RS------------KSLSEHNPS----------------------------------
-L--------------------------------RTH-----------------------------SGA----
-------------------------------------------------------------------------
-----------------------------------------------------------LP------------
--------------------------------------------KPNSS-----------PVTNVILQ-----
----------------SPFSL--------------------------GAI-----------TP----------
-----------IRT-LKS-------------------------------------------------------
---------------------------------------ITLSDWWLTK-KG-----------KEKKGLSIT-
--------------GFE-------------------------------T------------KG----------
-------------------------------GS-EV-RLF---------------------------------
--------------------------------------SSGTICKRHNSTTLEAIDGITICISGFI-------
NRSRTLEN-GVSNE--------VCNR-FLLGF--PYNW-------------EDYN------------------
-----------------------------------------EE------------------------------
-------------------------------------------------------------------------
-------------------------------------------------------------------------
-----------EEEEK------------------------KNV----------------DT------------
-------------------------------------------------------------------------
-----------------DFG-VSFDDV----------------------------------------------
---------------------------------------------------PVSRL-----------------
------QD-LFSL------------------------------------------------------------
-------------------------------------------------------------------------
-------------------------------------------------------------------------
-------------------------------------------------------------------------
-------------------------------------------------------------EGYLK-------
-------SKILD----------------------------------DVV------------------------
----------------------------------------G-------------------------------
SLRDFA-------------------------------------------------------------------
-------------------------------------------------------------------------
--CS-KSHKECDKS------------------------------------RIGDD------------------
-------------------------------------------------------------------------
-----------------------------------------------------------------SLVSR---
-------------------------------------------------VVGVKTRGMHRRR-EEY-V-A---
---------------------------------------------------------SIGKRV----------
-------------------------------------------------------------------------
-----------------------------RH------------------------------------------
--------------------------------------------------
TKRCCREQRAMHIHGGGPATSVQSNPNGSNT-IFKV--------EYT--
LKIIDEDGDTFAKRAEMYYRKRPEIVNFVEEAFRSYRALAERYDHLSR-ELQSANRTIA--------------
-TAFPEHVQ-----------------------FPLE---DDSD-
ENEDYEGNPRKPPKHLHLIPKGSNIPEVPEIPMKKDFRSQSMMLSRKGPAGLK--------RTISSA----
```

LAKREAAIVSSGLSKEEGLEEIDKLQKGILALQTEKEFVRSSYEQSYERYWDLESEVTEMQKRVCNLQDEFGL
GAAIDDSDARTLMASTALSSCKDTLAKLEEKQKQSV--EEAEIEKGRIKTAK----ERFDA---------
LRNKFEKSESDHHDEAIKTEVEEEE---------
GDVVQESSYESEREESNENLTVVKLAEKIDDLVHRVVSLETNASSHTALVKT-------LR--
SETDELHEHIRGLEEDKASLVSDSTDMKQR-------ITVLEDELRTVRKLFQ----
KVEDQNKNLQNQFKVANRTARDLSGKLQDVKMDEDVEGAGIFQELPVVSGSEDSRDELKSILTETETRSSLEE
TKKDATVVRESEDGGRSQEEKSEIKDSFALSETASTCFGTEAEDLVTEDEDEETPNWRQLLPDGMEDREKVLL
DEYTSVLRDYREVKRKLGEVEKKNREGFFELALQLRELKNAVAYKDVEIQSLRQKLNSPGKDSPHQVEGNNQ-
-------------LEHDQGQRE-SV--
SISPTSNFSVSTTPHHQGGDVKRTPGRTKSNEVRVKFADVDDSPRTKIPTVEDKVRA-
DIDAVLEENLEFWLRFSTSVHQIQKYQTTVQDLKSELSKLRIESKQQLESPR-SS-SNLAVASEAKPIYRHL-
-REIRTELQL-WL------ENSAVLEDE-----
LQGRYASLANIQEEIARVTAQSGGSKVSDSEISGYQAAKFHGEILNMKQENKRVSTELQSGL-DRVRALK---
---TEVERILSKLEEDLGISSATEARTTP-SKSS-----------------------------SSGRPRI---
------------------------------------PLRSFLFG----VKLKKHRQQKQSSSS----------
----

--------------------------------LFSCV------------------------------------
----
---------------------------SPS---
--PGLQKPS--------------------

---------------------SYNRPP---
GKL---P----------E
>Boechera_stricta_6251s0018.1.p

----MAEH--------------------R--------------------------------EPNL--------
-------------------------------------DD---------------------------------
D---G-------------------------------SK-----------------------------SSF---
----

----------------QKT--
--VVLRDWWLVK-CPKE--------FEG-
KRFGVA---------------GIE------------------------------DS------------GE---
--------------------------------------TR-AM-RVF--------------------------
----
TSSPIIKALDVFTLLASDGIYITLRGFL-------NKERVVKN-GFTPE--------ISRE-FIFGF--
PPCW-------------ERIC--
-------YNCFGGGVSLGT-DTNTV----------------------------------PSTIA---------
----
----------------------------KASYPILSPC------------KNNK-------------------
------ENL----------------QDTSAE--
--ERGENTVPDM----------D---------------------------------------TAEI-------
-----------NNTKDG------------------------------CR------------------------
------------ARAKKIARR------------------------KSLHLQTKSG------------------
-GKSAEDE--RILESSKVHNTTN--------
---DGDHGSEGLHKGK--SGEVEKD----
ECEAI--
---NNEDNEWRLDES----KLQNMT--
------------TNDGD------------HGSEGL---------------DK-AK------------------
SGGVEK-DEC------EAINN--GVISPADGCG--
----RNHTAAD-N------------------------------VDKVTSTSTTPE------------------

```
------------------------------------SLTSEQRK----GE----LKVTTASPHS---------
-------LLKDL-------HKNSKPRK-------------KGKS-K-KSEKTLKNDGNVVEPSNC--------
----------------SETKVKSAEN--------------KR-------------------------KPQ-
NPTTNDRDR-GKEGLNN----AKSDDVEI---------------DE----------CVAIND-----------
----EVI------------------SPADGCGK-RHSGTD---------------------------------
-------------------GVEKLASKNATIES-L-----TS-------------------------------
--------------------------EQRKGRVKETKTSLHS-------------------------------
--------------------------------------------------------LSKDLNNS-----
SKPGKKGKS-------------------------------------------------KKS------------
-------------------------EK----------------------------------------------
--------------------------------------------------TLKR-DW----------------
------------------------------------------------------------------HATE---
-------------------------------------------------------------------------
---------YNF-------------------------------------------------------------
-------------------------------------------------------------------------
-------------------------------------------------------------------------
---SWESEENLSWGNTKR-------------------------------------------------------
-----KIDFDVE-------------------------------------------------------------
--------------------VTPDNKV----------------------------------------------
-------------------------------------------------------------------KKQ-K-
-----------------------------------------------TNA-----------------------
----TSTDSLGQ--------------------------KRSR-SG----------------RVLVSSLEF-W-
-------RNQIPVYDV--------------------------------------------ARNLIEVKD----
-------GH-ET-N--------------------------------STP-SKG--------------------
----------KGS------------------------------------------------------------
-DSRK--------------------------------------------------------------------
-------------------------------------------------------------------------
-------------------------------------------------------------------------
-------------------------------------------------------------------------
-------------------------------------------------------------------------
-------------------------------------------------------------------------
---------------------------------------------------------------------RRS-
-------------------------------------------------------------------------
-------------------------------------------------------------------------
-------------------------------------------------------------------------
-------------------------------------------------------------------------
-------------------------------------------------------------------------
-------------------------------------------------------------------------
---------------
>Brassica_cretica_RQL77229.1
-------------------------------------------------------------------------
-------------------------------------------------------------------------
-------------------------------------------------------------------------
----MADN--------------------PN------------------------------PNPNP--------
------------------------------------DDE---------------------------------
D------------------------------------V-----------------------------SYY---
-------------------------------------------------------------------------
-------------------------------------------------------------------------
-------------------------------------------------------------------------
-------------------------------------------------------------------------
----------------QKT------------------------------------------------------
----------------------------------------VVLRDWWLIK-CPIE--------FEG-
KRFGVA---------------GTQI-------------------------------------------AE---
--------------------------------------TG-AV-RVF--------------------------
---------------------------------------------
TSSPIVKAFDVFTLEASDGVCIVLRGFL-------NKQRLVLS-GFLPQ--------ICSE-FILGF--
PPCW-------------ESKC----------------------------------------------------
-------NLSFVG-LPS---AIN--------------------------------------------------
-------------------------------------------------------------------------
----------------------------KASGAILSPC------------NNDKK------------------
```

------RNL----------------EDT---
------KTTVTA----------K---------------------------------------KKKK-------
-----------KN----------------------------------TV------------------------
------------EISDKPSRK------------------------KSIRLQSKS-------------------
----
---VE----------

---------LM---S----KVQTT-------
--------SSTND-------------VSDGL---------------DKRGES-----------------
SDDVEKTDEC------EVINN--QVD---

--GNVVERVNHQ-------
----------------SGTKVK-------------------RKL--------------------
DVSQVQKNPTTND-------------------GVER---------------DE----------SM-VNE----
-----------E-ISP----------------SPVDGC------GTN--------------------------
--------------------------S-KTITSKNA---T-L-----TS------------------------
---------------------------------EERNGKLKVTKTSL--------------------------
----
-------KNG---KKS---------
----------------------------EK---
---ILEG-DLD------------
--DV--
VVEPMM---
-------------TTHSR---

---------SSKVKHNLSVGKTIR---
-----------KIDFDAE---
--------------------------LTPEKDA--

KKQ-K--TNS-----------------
----------MSADSLGQ--------------------------KRSR-SG----------------
RVLVSPLEY-W-------RNQLPVYDK---
DRNLIQVNE-----------GH-QTNN--------------------------------ST-PSK--------
----

>Brassica_cretica_RQL80743.1

----MATK--------------------SKLQSLSARR----------------------SSPRT--------
----------------RS------------GAVREPIST----------------------------------
-RA-------------------------------ASC-----------------------------SRF----
----
---VP------------
--KPNS---------------DEIPR-----

----------------TPFSF--------------------------KSI-----------TPI---------
-----------GGT-LKS---
---------------------------------------VSLSDWWLTK-KA-----------NE-KGLGVA-
--------------GFE-------------------------------S------------KG----------
-------------------------------GP-EV-RLF---------------------------------
--------------------------------------SSATISTRHDSTTLETSDGLTVSISGFI-------
NRSRSLQN-GLSSE--------VCNR-FLLGF--PYHW-------------RDYT------------------
---EQGFLEE-------------------------

-----------EEEKD---

------------------YG-FSFDDI--
---PVNRL-----------------
------QDVLFTA--

---SPRFQ-------
-------DKMLD----------------------------------DAV------------------------
--D-------------------------------
SLRDLL--RST--

--TE-KPDKECRTP------------------------------------RMDGG------------------
----
DK-------------------------------E-------------------------------SLVLS---
---VVGVKTRGMVRRR-EEGGE-A---
---SIGERVL---------

-----------------------------RSSKK---------------------------------------
----------KRD--

---Q-------


```
>Brassica_juncea_A040432
-------------------------------------------------------------------------
-------------------------------------------------------------------------
-------------------------------------------------------------------------
----MATK--------------------SKLQSLSARR----------------------SSPRT--------
----------------RS------------GAVREPIST----------------------------------
-PA-------------------------------APC-----------------------------SRF----
-------------------------------------------------------------------------
-----------------------------------------------------------VP------------
--------------------------------------------KPNS---------------DEIPP-----
---------------RTPFSF--------------------------KSI-----------TP----------
------------TT-LKS-------------------------------------------------------
---------------------------------------VSLSDWWLTK-KA-----------NE-KGLGVS-
--------------GFE-------------------------------S------------KG----------
-------------------------------GP-EV-RLF---------------------------------
--------------------------------------SSAAISTRHDSTTLETSDGLTVSISGFI-------
NRSRSFQN-GFSSE--------DCNR-FLLGF--PYHW-------------KDYT------------------
-----------------------------------------EERFV---------------------------
-------------------------------------------------------------------------
-------------------------------------------------------------------------
-----------EEEKD---------------------------------------------------------
-------------------------------------------------------------------------
------------------HC-VSFDDI----------------------------------------------
---------------------------------------------------PVNRL-----------------
------QDVLFTA------------------------------------------------------------
-------------------------------------------------------------------------
-------------------------------------------------------------------------
-------------------------------------------------------------------------
-------------------------------------------------------------SPRFQ-------
-------AKILD----------------------------------DAV------------------------
----------------------------------------D-------------------------------
SLRDLL--RSS--------------------------------------------------------------
-------------------------------------------------------------------------
--TE-KPDKECRTP------------------------------------RMDGG------------------
-------------------------------------------------------------------------
NE-------------------------------E-------------------------------SLVLS---
-------------------------------------------------VVGVKTRGMVRRR-EEG-E-A---
---------------------------------------------------------SIGERVL---------
-------------------------------------------------------------------------
-----------------------------RSSKK---------------------------------------
----------NKF------------------------------------------------------------
-------------------------------------------------------------------------
-------------------------------------------------------------------------
-------------------------------------------------------------------------
-------------------------------------------------------------------------
-------------------------------------------------------------------------
-------------------------------------------------------------------------
-------------------------------------------------------------------------
-------------------------------------------------------------------------
-------------------------------------------------------------------------
-------------------------------------------------------------------------
-------------------------------------------------------------------------
-------------------------------------------------------------------------
-------------------------------------------------------------------------
---------------LLN-------------------------------------------------------
-------------------------------------------------------------------------
-------------------------------------------------------------------------
-------------------------------------------------------------------------
-------------------------------------------------------------------------
-------------------------------------------------------------------------
```


>Brassica_juncea_A040823

----MAD--NPNP--------
------------------------------------DEE---------------------------------
D------------------------------------V-----------------------------SYY---
----

----------------EKT--
--VVLRDWWLIK-CPIE--------FQG-
KRFGVA---------------GTQI---AE---
--------------------------------------TG-AV-RVF--------------------------
----
TSSPILKAFDVFTLEASDGVCIVLRGFL-------NKPRLVQS-GFLPQ--------ICSE-FILGF--
PPYW-------------ESKC--
-------NLSFVG-LPSGSASIN--

----------------------------KASGTILSPC------------NNDKK------------------
------RNL----------------EDTPAR--
--RRVVKTTVTA--KKKK-------
-----------QN----------------------------------TV------------------------
------------EISDRPSRK------------------------KSLRLQSKS-------------------
----
---VE----------

---------LM---S----KVQTT-------
--------SSTND----------------GL---------------DKSAKC-----------------
SDDVEKTDES------EVINH--QVDG----CV--
----KKRNGAD-N------------------------------VVKVTRMSATEA------------------
----
--ANVVERVNHQ-------
----------------SRTKVK-------------------RKL--------------------
DVSELQKNLTTND-------------------GVER---------------DE----------PM-DNE----
-----------EEISP----------------SPVDGC------GTD--------------------------
--------------------------T-KKITSKNA---T-L-----TS------------------------
---------------------------------EERNDKLKVTKTSL--------------------------
----
-------KNG---KKS---------
----------------------------EK---
---ILEG-DLD------------
--DV--
VVEPMT---
-------------TTHSR---

---------SSKLKHNLSVGKTIR---
-----------KIDFDQE---

--------------------------VTPEKDA--

TKQKK--TNS-----------------
----------MSADSLGQ--------------------------KRSR-SG----------------
RVLVSPLEY-W-------RNQLPVYDK---
DRNLIQVNE-----------GR-QT-N--------------------------------TT-SSKG-------
-----------------------KG--
------------GSVSRK---

---------PRR---

>Brassica_juncea_B007540

---MATTK--------------------SKPHSLSARR----------------------SPPRT--------
----------------RS------------GALPKPLST----------------------------------
-P--------------------------------ATR-----------------------------SRF----
----
---LP------------
--KPNS---------------DEITP-----
---------------RTPFSS--------------------------KSI-----------TPII--------
-----------GGT-PKS---
---------------------------------------VSLSDWWLTN-DG--------------KGLGVA-
--------------GFE-------------------------------S------------------------
----------------------------------EA-RLF---------------------------------
--------------------------------------SSATISTRHDSTTLETSDGITVSVSGFI-------
NRSRSLEN-GFSSE--------VCNR-FLLGF--PYHW-------------KDYT------------------
---EEGFVE--------------------------

-----------EEDEE------------------------EEK----------------D-------------
----
------------------YG-VSFDDI--
---PVDRF-----------------
------EDVLFTA--

---SPRFQ-------
-------DKILG----------------------------------DAI------------------------
--D-------------------------------
SLRDLL--RS---

GNEECQ-EAEKECEERSDKTP------------------------------IRMDGG----------------
----
--DE-------------------------------EE------------------------------GLVLS-
---DEGVKTRGMLRRR-EEG-E-A-
---SIGERLH-------

-------------------------------RSSKK-------------------------------------
------------KRD--
---------------------------------QEKR------------------------------------
----

>Brassica_juncea_B012077

----MAEN--------------------H--------------------------------NPNP--------
-------------------------------------EE---------------------------------
D------------------------------------V-----------------------------SYY---
----

----------------QTR--
--VVLRDWWLIK-CSNE--------FQG-
KRFGVA---------------GTQI---AE---
--------------------------------------TG-AA-RFF--------------------------
----
TSSPIIKAYDVFTLEASDGVCISLRGFL-------NKQRLSQS-GFLPE--------ISRE-FIFGF--
PPCW-------------ESKC--
-------NHFFLGEVPS-EAGTN--

----------------------------KASPTIISPC------------NNTK-------------------
-------------------------WNSPAE--
--RRGKTTAVEN----------N---------------------------------------TEEE-------
-----------TNDKDGLG--------------------------GGST------------------------
------------ARDDKPARK------------------------KSLRLQSKS-------------------
----
---VE----------

---------LM---N----KVKNK-------

--------TTNHG--------------SEGL---------------SKEAK------------------
SGGVAK-DEC------EVISN--EVISP---AG--
----KKHTSAD-D------------------------------VDKVTCMSASGE------------------
------------------------------------S----QGK----GE----LEATKACPHS---------
-------LSEDL-------EKSGEPGK-------------KTVS-E-KSGRILRSDGNVVEHVNH--------
----------------SGTKAK-------------------RKL--------------------
DVSQVQKNPATNDVGN-GSEGLNK----AKGNGVEG---------------DE----------CM-GNE----
-----------E-I------------------SRVDGCGKMQHSGTN--------------------------
--------------------------S-KKVTSKNANKES-L-----TS------------------------
---------------------------------EQRNGKLKVTKTSLS-------------------------
--DSS--
---SKHGKNGKS---KKG---------
----------------------------EQ---
---ILRG-DL-------------
--DV--
VVEPM--
--------------THSR---

----------SKVKNNLSVGKTIR---
-----------KIDFDVE---
--------------------------VTPEKDA--

KKQ-K--TNA-----------------
---------VLSADSLGQ--------------------------KRSR-SG----------------
RVLVSPLEF-W-------RNQVPVYDV---
DRNLIQVKE-----------GH-QT-N--------------------------------STPPSKG---SFF-
----------------------FKA--
------------LYIYRR---

---------GLH---

>Brassica_napus_GSBRNA2T00016820001

----MAD--NPNP--------
------------------------------------DDD---------------------------------
D------------------------------------V-----------------------------SYY---
----

----------------QKT--
--VVLRDWWLIK-CPIE--------FDG-
KRFGVA---------------GTQI---AE---
--------------------------------------TG-AV-RVF--------------------------
----
ASSPIVKAFDVFTLEASDGVCIVLRGFL-------NKQRLVLS-GFLPQ--------ICSE-FILGF--
PPCW-------------ESKC--

-------NLSFVG-LPSGSASIN--

----------------------------KASGAILSPC------------NNDKK------------------
------RNL----------------EDT---
------KSTVTA--KKKK-------
-----------KN----------------------------------TV------------------------
------------EISDKPSRK------------------------KSIRLQSKS-------------------
----
---VE----------

---------LM---S----KVQTT-------
--------SSTND-------------VSDGL---------------DKRGKS-----------------
SDDVEKTDEC------EVINN--QVD---

--GNVVELVNHQ-------
----------------SGTKVK-------------------RKL--------------------
DVSQVQKNPTTND-------------------GVER---------------DE----------SM-VNE----
-----------E-ISP----------------SPVDGC------GTN--------------------------
--------------------------S-KKITSKNA---T-L-----TS------------------------
---------------------------------EERNGKLKVTKTSL--------------------------
----
-------KNG---KKS---------
----------------------------EK---
---ILEG-DLD------------
--DV--
VVEPMM---
-------------TTHSR---

---------SSKVIHNLSVGKTIR---
-----------KIDFDAE---
--------------------------VTPEKDA--

TKQ-K--TNS-----------------
----------MSADSLGQ--------------------------KRSR-SG----------------
RVLVSPLEY-W-------RNQLPVYDK---
DRNLIQVNE-----------GH-QT-N--------------------------------ST-SSKG-------
-----------------------KGS---
--------------VSRK---

---------PRR---

>Brassica_napus_GSBRNA2T00024219001

----MATK--------------------SKLQSLSARR----------------------SSPRT--------
----------------RS------------GAVREPIST----------------------------------
-PA-------------------------------APC-----------------------------SRF----

----
---VP------------
--KPNS---------------DEIPP-----
---------------RTPFSF--------------------------KSI-----------TP----------
------------TT-LKS---
---------------------------------------VSLSDWWLTK-KA-----------NE-TGLGVS-
--------------GFE-------------------------------S------------KG----------
-------------------------------GP-EV-RLF---------------------------------
--------------------------------------SSAAISTRHDSTTLETSDGLTVSISGFI-------
NRSRSFQN-GFSSE--------DCNR-FLLGF--PYHW-------------KDYT------------------
---EERFV---------------------------

-----------EEEKD---

------------------HC-VSFDDI--
---PVNRL-----------------
------QDVLFTA--

---SPRFQ-------
-------AKILD----------------------------------DAV------------------------
--D-------------------------------
SLRDLL--RSS--

--TE-KPDKECRTP------------------------------------RMDGG------------------
----
NE-------------------------------E-------------------------------SLVLS---
---VVGVKTRGMVRRR-EEG-E-A---
---SIGERVL---------

-----------------------------RSSKK---------------------------------------
----
NKLIKGSHQNTRTKTISLFLSDQCFVFYASIYAHHHIRPLTKTCCREQRAMRIHGGGPATSVQSNPNGSNT-
IFKVPSIFHHVFEYT--LKIIDEDGDTFARRAEMYYRKRPEIVSFVEEAFRSYRALAERYDHLSR-
ELQSANRTIA---------------TAFPEHVQ-----------------------FPLE---DD---
ETEDFEGNPRKQP-HLHLIPKGSNIPQ--
-
REAAVVSSGLSKEEGLEEIDNLQKGILALQTEKEFVRSSYEESYERYWDLENEVAEMQKRVCSLQDEFGLGAA
IDDSDARTLMASTALSSCKDTLAKLEEKQKQSV--EEAEIEKERITTAK----ERFYA---------
LRNKFEKPESDDHDKFIKTEAK------------
VDVVQESSYESEREDSNENLTVVKLAEKIDDLVQKIVSLESNASSHTALVKT-------LR--
SETDGLHEHIRGLEEDKAALVSDSTDMKQR-------IAVLEKELSEVRKLFQ----
KVEDQNKSLQKQFKEANWTADDLSGKLQDVKMDEDVEGAGIFQELPAVSGSEDY---LKSITKETER------
----
EKDEDEETPNWRQLLPDGMEDREKVLLDDYTSVLRDYRGVKRKLGEVEKKNREGFFELALQLRELKNAVAYKD
VEIQSLRQKLGTLEKDSPHQVEGNNQ--------------MEHDQGQRE-SV--SISPTSNFSV---------
--------------RVKFADVDDSPRTKIPAVEDKVRA-
DIDAVLEENLEFWLRFSTSVHQIQKFQTTVQDLKSELTKLKIQSKQQQES---SR-SKHAAASEAKPIYRHL-
-REIRTELQL-WL------ETSAVLKDE-----
LQGRFASLANIQEEIGRVTAHSGGSKVSDSEISSYQAAKFHGEILNMKQENKRVSSELQSGL-DRVRVLK---
---TDVERILSKLEEDIGISSATEARTTP-SKSS-----------------------------SSGKARI---
------------------------------------PLRSFLFG----VKLKKQTKQKQASAS----------
----

--------------------------------LFSCV------------------------------------
----
---------------------------SPF---

--PAPQQES--------------------

---------------------S---

>Brassica_napus_GSBRNA2T00086338001

----MATK--------------------SKLQSLSARR----------------------SSPRT--------
----------------RS------------GAVREPIST----------------------------------
-RA-------------------------------ASC-----------------------------SRF----
----
---VP------------
--KPNS---------------DEIPR-----
----------------TPFSF--------------------------KSI-----------TPI---------
-----------GGT-LKS---
---------------------------------------VSLSDWWLTK-KA-----------NE-KGLGVT-
--------------GFE-------------------------------S------------KG----------
-------------------------------GP-EV-RLF---------------------------------
--------------------------------------SSATISTRHDSTTLETSDGLTVSISGFI-------
NRSRSLQN-GLSSE--------VCNR-FLLGF--PYHW-------------RDYT------------------
---EEGFLEE-------------------------

-----------EEEKD---

------------------YG-VSFDDI--
---PVNRL-----------------
------QDVLFTA--

---SPRFQ-------
-------DKMLD----------------------------------DAV------------------------
--D-------------------------------
SLRDLL--RST--

--TE-KPDKECRTP------------------------------------RMDGG------------------
----
DK-------------------------------E-------------------------------SLVLS---
---VVGVKTRGMVRRR-EEGCE-A---
---SIGERVL---------

-----------------------------RSSKK---------------------------------------
----------KRD--

---Q-------

>Brassica_napus_GSBRNA2T00119574001

----MADN--------------------P-------------------------------NPNP--------
------------------------------------DEE--------------------------------
D------------------------------------V----------------------------SYY---
----

----------------EKT---
---------------------------------------VVLRDWWLIK-CPIE--------FQG-
KRFGVA---------------GTQI--AE---
-------------------------------------TG-AV-RVF--------------------------
----
TSSPILKAFDVFTLEASDGVCIVLRGFL-------NKPRLVHS-GFLPQ-------ICSE-FILGF--
PPYW-------------ESKC---
-------NLSFVG-LPSGSASIN---

----------------------------KASGTILSPC-----------NNDKK------------------
------RNL----------------EDTPAR---
--RRVVKTIVTA---KKKK-------
-----------QN---------------------------------TV------------------------
------------EISDRPSRK-----------------------KSLRLQSKS-------------------
----
--VE----------

---------LM--S----KLQTT-------
--------SSTND----------------GL--------------DKSAKC-----------------
SDDVEKTDES------EVTNN--QVDG----CG---------------------------------------
----KK--

--RVNHQ-------
----------------SGTKVK------------------RKL--------------------
DVSELQKNPTTN--------------------------------------DE----------SM-DNE----
-----------E-ISP----------------SPVDGC------GTN-------------------------
--------------------------S-KKITSKNA---T-L-----TS-----------------------
---------------------------------EERNGKLKVTKTSL-------------------------
----
-------KNG--KKS---------
----------------------------EK--
--ILEG-DLD------------

--DV--
VVEPMT---
-------------TTHSR---

---------SSKVKHNLSVGKTIR---
-----------KIDFDLE---
--------------------------VTPEKDA--

TKHNK--TNS-----------------
----------MSADSLGQ--------------------------K----------------------
RVLVSPLEY-W-------RNQLPVYDK---
DRNLIQVNE-----------GR-QT-N--------------------------------ST-SSKG---LFF-
----------------------FS---

>Brassica_nigra_B045991-PA

---MATTK--------------------SKPHSLSARR----------------------SPPRT--------
----------------RS------------GALPKPLST----------------------------------
-P--------------------------------ATR-----------------------------SRF----
----
---LP------------
--KPNS---------------DEITP-----
---------------RTPFSF--------------------------KSI-----------TPTI--------
-----------GGT-PKS---
---------------------------------------VSLSDWWLTN-DG--------------KGLGVA-
--------------GFE-------------------------------S------------------------
----------------------------------EA-RLF---------------------------------
--------------------------------------SSAAISTRHDSTTLETSDGITVSISGFI-------
NRSRSLEN-GFSSE--------VCNR-FLLGF--PYHW-------------KDYT------------------
---EEGFVE--------------------------

-----------EEDEE------------------------EED----------------D-------------
----
------------------YG-VSFDDI--
---PVDRF-----------------
------EDVLFTA--

---SPRFQ-------
-------DKILG----------------------------------DAI------------------------
--D-------------------------------
SLRDLL--RGR--

PDQECQKKPEKECEERSDKTP-----------------------------IRMDGG----------------
----
--DE-------------------------------EE-----------------------------GLVLS-
--DEGVKTRGMLRRR-EEG-E-L-
--SIGERLH-------

-------------------------------RSSKK------------------------------------
------------KKG---
---------------------------------EKGENFLLRV-----------------------------
----

--------------------------------------CF--------------------------------
--RNIVV---------------------------

>Brassica_nigra_B033030-PA

---MIK------------------------

---------------------------------LI------------------------------------
----

---AASPTIISPC-
-----------NNAK---WNSPAE--------
-----------------------------------RRDESTVVEN----------N----------------

-----------------------TEEE--
--EISDKLARK------------------
------KSLRLQSKS--

----------------------VE---
---LM----------------------------
-----------------S----KVKNK---------------TTNHG--------------SEGL--------
-------SKEAK------------------SGGVAK-DKC------EVISN--EVISP---AG----------
----------------------------------KKHTSAD-D------------------------------
VDNVTCMSASGE--S----
QGK----GE----LEATKACPHS----------------LSEDL-------EKSGEPGK-------------
KTVS-E-KSGRILRSDGNVVEHVNH------------------------SGSKAK------------------
-RKI--------------------DVSQVQKNPATNDVGN-GSEGLNK----AKGNGVEG-------------
--DE----------CM-GNE---------------E-I------------------SRVDGCGKMQHSGTN--
--S-KKVTSKNANKES-L-----
TS---
EQRNGKLKVTKTSLS--
-----------------------------------DSS-----SKHGKNGKS---------------------
----------------------------KKS-------------------------------------GQ---
----
--------------------ILKG-DL--
---------------------------------DV--SVEPM-------------------------------
---THSR------------------

---SKVKDNLSVGKTIR------------
--KIDFDVE------------------
---VTPEKDA---

-------------------------------------KKQ-K-------------------------------
-----------------TNA--------------------------VLSADSLGQ------------------
--------KRSR-SG----------------RVLVSPLEF-W-------RNQVPVYDVVC-------------
------------------------------DDVLL---------------------N----------------
----------------NT---

>Brassica_oleracea_1g087890.1

----MATK--------------------SKLQSLSARR----------------------SSPRT--------
----------------RS------------GAVREPIST----------------------------------
-RA-------------------------------ASC-----------------------------SRL----
----
---VP------------
--KPNS---------------DEIPR-----
----------------TPFSF--------------------------KSI-----------TPI---------
-----------GGT-LKS---

```
---------------------------------------VSLSDWWLTK-KA-----------NE-KGLGVA-
--------------GFE-------------------------------S------------KG----------
-------------------------------GP-EV-RLF---------------------------------
--------------------------------------SSATISTRHDSTTLETSDGLTVSISGFI-------
NRSRSLQN-GLSSE--------VCNR-FLLGF--PYHW-------------RDYT------------------
-----------------------------------------EEGFLEE-------------------------
-------------------------------------------------------------------------
-------------------------------------------------------------------------
-----------EEEKD---------------------------------------------------------
-------------------------------------------------------------------------
------------------YG-VSFDDI----------------------------------------------
---------------------------------------------------PVNRL-----------------
------QDVLFTA------------------------------------------------------------
-------------------------------------------------------------------------
-------------------------------------------------------------------------
-------------------------------------------------------------------------
-------------------------------------------------------------SPRFQ-------
-------DKMLD----------------------------------DAV------------------------
----------------------------------------D-------------------------------
SLRDLL--RST--------------------------------------------------------------
-------------------------------------------------------------------------
--TE-KPDKECRTP------------------------------------RMDGG------------------
-------------------------------------------------------------------------
DK-------------------------------E-------------------------------SLVLS---
-------------------------------------------------VVGVKTRGMVRRR-EEGGE-A---
---------------------------------------------------------SIGERVL---------
-------------------------------------------------------------------------
-----------------------------RSSKK---------------------------------------
----------KRD------------------------------------------------------------
-------------------------------------------------------------------------
-------------------------------------------------------------------------
-------------------------------------------------------------------------
-------------------------------------------------------------------------
-------------------------------------------------------------------------
-------------------------------------------------------------------------
-------------------------------------------------------------------------
-------------------------------------------------------------------------
-------------------------------------------------------------------------
-----------------------------------------------------------------Q-------
-------------------------------------------------------------------------
-------------------------------------------------------------------------
-------------------------------------------------------------------------
-------------------------------------------------------------------------
-------------------------------------------------------------------------
-------------------------------------------------------------------------
-------------------------------------------------------------------------
-------------------------------------------------------------------------
-------------------------------------------------------------------------
-------------------------------------------------------------------------
-------------------------------------------------------------------------
-------------------------------------------------------------------------
-------------------------------------------------------------------------
-------------------------------------------------------------------------
-------------------------------------------------------------------------
-------------------------------------------------------------------------
-------------------------------------------------------------------------
-------------------------------------------------------------------------
----------------------------------------
>Brassica_oleracea_2g002600.1
```

```
-------------------------------------------------------------------------
-------------------------------------------------------------------------
-------------------------------------------------------------------------
----MAD------------------------------------------------------NPNP--------
------------------------------------DDD---------------------------------
D------------------------------------V-----------------------------SYY---
-------------------------------------------------------------------------
-------------------------------------------------------------------------
-------------------------------------------------------------------------
-------------------------------------------------------------------------
----------------QKT------------------------------------------------------
----------------------------------------VVLRDWWLIK-CPIE--------FEG-
KRFGVA---------------GTQI-------------------------------------------AE---
--------------------------------------TG-AV-RVF--------------------------
---------------------------------------------
TSSPIVKAFDVFTLEASDGVCIVLRGFL-------NKQRLVLS-GFLPQ--------ICSE-FILGF--
PPYW-------------ESKC----------------------------------------------------
-------NLSFVG-LPSGSASIN--------------------------------------------------
-------------------------------------------------------------------------
----------------------------KASGAILSPC------------NNDKK------------------
------RNL----------------EDSPAR------------------------------------------
--RRVVKTTVTA----------K---------------------------------------KKKK-------
-----------KN----------------------------------TV------------------------
------------EISDKPSRK------------------------KSIRLQSKS-------------------
-------------------------------------------------------------------------
-------------------------------------------------------------VE----------
-------------------------------------------------------------------------
---------LM---------------------------------------------S----KFQNT-------
----------TND------------DVSEGL---------------DKRAKS-----------------
SDDVEKTDEC------EVINN--QVDG----CV----------------------------------------
----KKRTSAD-N------------------------------VDKVTIMRATEE------------------
------------------------------------SLTLKQGK----GE-----------------------
-------LEES--------EKSGKRGKK------------AAVS-K-KSG------GNAVEHVNHQ-------
----------------SGTKVK-------------------RKL--------------------
DVSQVQKNPTTND-------------------GVER---------------DE----------SM-VNE----
-----------E-ISP----------------SPVDGC------GTN--------------------------
--------------------------S-KKITSKNA---T-L-----TS------------------------
---------------------------------EERNGKLKVTKTSL--------------------------
-------------------------------------------------------------------------
-------KNG---------------------------------------------------KKS---------
----------------------------EK-------------------------------------------
-----------------------------------------------------ILEG-DLD------------
------------------------------------------------------------------DV--
VVEPMM-------------------------------------------------------------------
-------------TTHSR-------------------------------------------------------
-------------------------------------------------------------------------
-------------------------------------------------------------------------
---------PSKVIHNLSVGKTIR-------------------------------------------------
-----------KIDFDAE-------------------------------------------------------
--------------------------VTPEKDA----------------------------------------
-------------------------------------------------------------------------
TKQ-K------------------------------------------------TNS-----------------
----------MSADSLGQ--------------------------KRSR-SG----------------
RVLVSPLEY-W-------RNQLPVYDK---------------------------------------------
DRNLIQVNE-----------GH-QT-N--------------------------------ST-SSKG-------
-----------------------KGS-----------------------------------------------
--------------VSRK-------------------------------------------------------
-------------------------------------------------------------------------
-------------------------------------------------------------------------
-------------------------------------------------------------------------
```


---------PRR---

>Brassica_rapa_A01g027290.3C

----MATK--------------------SKLQSLSARR----------------------SSPRT--------
----------------RS------------GAVREPIST----------------------------------
-PA-------------------------------APC-----------------------------SRF----
----
---VP------------
--KPNS---------------DEIPP-----
---------------RTPFSFS------------------------LKSI-----------TPT---------
------------TT-LKS---
---------------------------------------VSLSDWWLTK-KA------------NE-TGLGVS-
--------------GFE-------------------------------S------------KG----------
-------------------------------GP-EV-RLF---------------------------------
--------------------------------------SSATISTRHDSTTLETSDGLTVSISGFI-------
NRSRSFQN-GFSSE--------DCNR-FLLGF--PYHW-------------KDYT------------------
---EERFV---------------------------

-----------EEEKD---

------------------HC-VSFDDI--
---PVNRL-----------------
------QDVLFTA--

---SPRFQ-------
-------AKILD----------------------------------DAV------------------------
--D-------------------------------
SLRDLL--RSC--

--TE-KPDKECRTP------------------------------------RMDGG------------------
----
NE-------------------------------E-------------------------------SLVLS---
---VVGVKTRGMVRRR-EEG-E-A---
---SVGERVL---------

-----------------------------RSSKK---------------------------------------
----------NKF--

---------------LLN---

>Brassica_rapa_A02g000520.3C

----MADN--------------------P--------------------------------NPNP--------
------------------------------------DEE---------------------------------
D------------------------------------V-----------------------------SYY---
----

----------------EKT--
--VVLRDWWLIK-CPIE--------SQG-
KRFGVA---------------GTQI---AQ---
--------------------------------------TG-AV-RVF--------------------------
----
TSSPILKAFDVFTLEASDGVCIVLRGFL-------NKPRLVQS-GFLPQ--------ICSE-FILGF--
PPYW-------------ESKC--
-------NLSFVG-LPSGSASIN--

----------------------------KASGTILSPC-------------NDKK------------------
------RNL----------------EDIPAQ--
--RRVVKTTVTA---NKK-------
-----------QN----------------------------------TV------------------------
------------EISDKPSRK------------------------KSLRLQSKS-------------------
----
---VE----------

---------LM---S----KVQTT-------
--------SSTND----------------GL---------------DKSAKC-----------------
SDDVEKTDES------EVTNN--QVDG----CG--
----KK---

---HVNHQ-------
----------------SGTKVE-------------------RKL--------------------
DVIELQKNPTTND-------------------GVER---------------DE----------PM-DNK----
-----------EISSP----------------SPVDGC------GTN--------------------------
--------------------------T-KKITSKNA---T-L-----TS------------------------
---------------------------------EERNGKLKVTKTSL--------------------------
----

-------KNG---KKS---------
----------------------------EK---
---ILQG-DLD------------
--DV--
VVEPMT---
-------------TTHSR---

---------SSKVKHNLSVGKTIR---
-----------KIDFDQE---
--------------------------VTPEKDA--

TKHNK--TNS-----------------
----------MSADSLGQ--------------------------KRSR-SG----------------
RVLVSPLEY-W-------RNQLPVYDK---
DRNLIQVNE-----------GR-QT-N--------------------------------TT-SSKG-------
-----------------------KG--
------------GSVSRK---

---------PRR---

>Camelina_sativa_XP_010414757.1

----MATK--------------------SKPQSLSARCS---------------------SPPRT--------
----------------RS------------KPLPETNPSPRTRSKPL--------------------PETN--
-P-------------------SP-----------RTH-----------------------------PEA----
----
---LP------------
--KPNFP-----------P----TPR-----
----------------TPASL--------------------------GAI-----------TPI---------
------------VK-TKS---
---------------------------------------VTLSDWWLTR-KG-K--------DKEKKALCIT-
--------------GFE--
--------------------------------S-DV-RLF---------------------------------
--------------------------------------SSGTILKRHNSVTLESVDGITISISGFI-------
NRARSMEN-GVSEE--------VCNR-FLLGF--PFNW-------------EDYN------------------
---EENVV---------------------------

-----------DEDR--
---G---------------
------------------FV-VSFNDV--
---PVNRI-----------------
------QD-LSFV--

---DGYLK-------

-------DRILV----------------------------------DVV------------------------
--A-------------------------------
SLRDMV---

--CP-KSDEKKKSV-----------------------------------V-----------------------
----
ED-------------------------------E-------------------------------SLVSSA--
--VVVGVKTRAM-RRR-DEF-E-SS--
---SSGKRPVCT-------

-----------------------------RSTKR---------------------------------------
----------KKK--

------------------------LA---

>Camelina_sativa_XP_010452097.1

----MAEH--------------------R--------------------------------EPNL--------
-------------------------------------DD---------------------------------
N---G-------------------------------SK-----------------------------SYF---
----

----------------QKR--
--VVLRDWWLIK-CPKE--------FQG-
KRFGVA---------------GIE------------------------------DF------------VE---
--------------------------------------KR-AK-RVL--------------------------
----
TSSPIIKALDVFTLLASDGIYITLRGFL-------NKERVVQN-GFTPE--------ISRE-FIFGF--
PPCW-------------EQIC--
-------NNCFGGGVSLGT-DTNTV----------------------------------SSTVA---------

----
----------------------------KASYPILSPC------------NNKK-------------------
------ENLE---------------EDSPAE--
--GRDESNVADT----------I---------------------------------------AAEIT------
-----------TNTEDG------------------------------SR------------------------
------------PSYKITARR------------------------KSLHLQTRFG------------------
-G---ESSEVHNTTT--------
---DGDHGGEGLDEAK--SGNVEND----
TCEAI--DKGVVSPADGSG---------
RNHTGADNAD--------KVTSTVTTA----VSLTSEQQKGELRDGCEVFNNEDGERKLDES----ILQNLT-
-------------TNDGD------------HGSEGL---------------DK-AK-----------------
-SGDVDK-DEF------EAINN--GVILPADGCG---------------------------------------
-----RKRTDAD-N------------------------------VDKVTSTSTNGE-----------------
-------------------------------------PLTSEELN----GE----LKVTTASPHS--------
--------LFKDF-------DKSSKPGR-------------KGIS-K-KSRKTLKKTGNVVEPSHC-------
-----------------SETKVKSATK--------------KR-------------------------KPQ-
NPTTNDKDR-GKEGLKN----VKSDDVER---------------DE----------CVGIND-----------
----EVI---------------------DGCGR-RHSGTD---------------------------------
-------------------SVGKLTSKNATKES-L-----TS-------------------------------
--------------------------EQRKGREKETKTTL---------------------------------
--LSKDLN-------
SKPGKKGSS---KKS------------
-------------------------EK--
--TPKR-DL----------------
--QAAE---

---------ENL---

---SCESEENLSWGNTKR---
-----KIDFDVE---
--------------------VTPDNKV--
---KKQ-K-
---TSV-----------------------
----VSTDSLGQ--------------------------KRSR-SG----------------RVFMSSLEY-W-
------RNQIPVYDM---DRRLIEVKG----
-------GH-ET-N--------------------------------PTP-SKG--------------------
----------KGS--
-YRRK--

---PRS-

>Camelina_sativa_XP_010470274.1

---MVATK--------------------SKPQSLSARRS---------------------SPPRT--------
----------------RS------------KPLPETNPSPRTLSKPLPETSPSPRTHSK------PQPETN--
-P-------------------SP-----------RTH-----------------------------PQA----
----

```
------------------------------------------------------------LP------------
---------------------------------------------KPDFP-----------P----TPR-----
----------------TPASL---------------------------GAI-----------TPI---------
------------GT-RKS--------------------------------------------------------
----------------------------------------VTLSDWWLTR-KG-K--------DKKKKALCII-
--------------GFE---------------------------------------------------------
--------------------------------S-DV-RLF----------------------------------
---------------------------------------SSGTILKRHNSVTLESVDGITISIGGFI-------
NRSLSIEN-GVSEE--------VCNR-FLLGF--PFNW--------------EDYN------------------
------------------------------------------EENVV---------------------------
--------------------------------------------------------------------------
--------------------------------------------------------------------------
-----------EEDR-----------------------------------------------------------
----------------------------------------------------------G---------------
------------------FV-VKFDDV-----------------------------------------------
----------------------------------------------------PVNRI-----------------
------ED-LSFV-------------------------------------------------------------
--------------------------------------------------------------------------
--------------------------------------------------------------------------
--------------------------------------------------------------------------
--------------------------------------------------------------DGCLK-------
-------DKILV-----------------------------------DVV------------------------
-----------------------------------------A-------------------------------
SLRDLVS-------------------------------------------------------------------
--------------------------------------------------------------------------
--CP-KSDEKKKKS------------------------------------VAVG--------------------
--------------------------------------------------------------------------
ED-------------------------------E--------------------------------SLVSSA--
-------------------------------------------------VVVGVKTRAM-RRR-DEF-E--S--
----------------------------------------------------------SSGKRPVCT-------
--------------------------------------------------------------------------
-----------------------------KSTK-----------------------------------------
----------KKK-------------------------------------------------------------
--------------------------------------------------------------------------
--------------------------------------------------------------------------
--------------------------------------------------------------------------
------------------------LA------------------------------------------------
--------------------------------------------------------------------------
--------------------------------------------------------------------------
--------------------------------------------------------------------------
--------------------------------------------------------------------------
--------------------------------------------------------------------------
--------------------------------------------------------------------------
--------------------------------------------------------------------------
--------------------------------------------------------------------------
--------------------------------------------------------------------------
--------------------------------------------------------------------------
--------------------------------------------------------------------------
--------------------------------------------------------------------------
--------------------------------------------------------------------------
--------------------------------------------------------------------------
--------------------------------------------------------------------------
--------------------------------------------------------------------------
--------------------------------------------------------------------------
--------------------------------------------------------------------------
--------------------------------------------------------------------------
--------------------------------------------------------------------------
--------------------------------------------------------------------------
--------------------------------------------------------------------------
--------------------------------------------------------------------------
--------------------------------------------------------------------------
```


>Camelina_sativa_XP_010490713.1

----MAEH--------------------R--------------------------------EPNF--------
-------------------------------------DD---------------------------------
D---G-------------------------------SK-----------------------------SYS---
----

----------------QKT--
--VVLRDWWLVK-CPKE--------FEG-
KRFGVA---------------GIE------------------------------DP------------VE---
--------------------------------------KR-AV-RVF--------------------------
----
TSSPIIKALDVFTLLASDGIYITLRGFI-------NKERLVKN-GFTPE--------ISRE-FIFGF--
PPCW-------------EQIC--
-------NNCFGGGVSLGTDDTNTV----------------------------------SSTVS---------
----
----------------------------KASYPILSPC------------KNNK-------------------
------ENLE---------------EDSLAE--
--GRDESNVTDT----------I---------------------------------------ATEIT------
-----------TNTEDV------------------------------SR------------------------
------------PRYKITARR------------------------KSLHLRTRFG------------------
-GKSVEDQ--RILESSEVHNTTN--------
---DGDLGSEGFDEAK--SGDVEND----
NCEGI--DNGVVSPADGSG---------
RNHTGADNVD--------KVTSTITTA----ESLTSEQL---LIDECEVINHEDGVKKLDDS-----------
-------------TNDGD------------RGSEGL---------------DK-AK-----------------
-SDSVDQ-DEF------EAINN--GVLLPADGCG---------------------------------------
-----RTRIDADNN------------------------------VDKVTSASATGE-----------------
-------------------------------------SLTSEEQK----GE----LKVTAASPHS--------
--------LFKDL-------DKSSKPGR-------------KGMS-K-KSRKTLKKAGNVVEPSHR-------
-----------------SETKVKSAAN--------------KR-------------------------KPQ-
KPTTNDKDR-GKEDVNN----AKSDDVER---------------DE----------CVGIND-----------
----EVI---------------------DGCGR-RHSGTD---------------------------------
-------------------GVGKLTSKNVTKES-P-----TS-------------------------------
--------------------------KQRKGREKETKTSL---------------------------------
--LSKDLN-------
SKPGKKKSS---KKS------------
-------------------------EK--
--TPKR-DL----------------
--QAAE---

---------ENL---

---SCESEENLSWGNTKR---
-----KIDFDVE---
--------------------VTPDNKV--
---KKQ-K-
---TNA-----------------------
----VSTNSVGQ--------------------------KRSR-SG----------------RVLVSSLEY-W-
------RNQIPVYDM---ERRLIEVKG----
-------GH-ES-N--------------------------------PTL-SKG--------------------
----------KVS--
-YRRK--

---PRS-

>Camelina_sativa_XP_019084578.1

----MAEH--------------------R--------------------------------EPNL--------
-------------------------------------ED---------------------------------
D---G-------------------------------SK-----------------------------SYS---
----

----------------QKR--
--VVLRDWWLVK-CPKE--------VEG-
KRFGVA---------------GIE------------------------------DP------------VE---
--------------------------------------KG-AV-RVF--------------------------
----
TSSPIIKALDVFTLLASDGIYITLRGFI-------NKERLVKN-GFTPE--------ISRE-FIFGF--
PPCW-------------EQFC--
-------NNCFGGGVSLGT-DXXTY----------------------------------SSHVVC--------
----
---------------------------FAASYPILSPC------------KNNK-------------------
------ENLE---------------EDSPAE--
--GRDESNVTDT----------N---------------------------------------AAEIT------
-----------TNTEDG------------------------------SR------------------------
------------PRYKITARR------------------------KSLHLQTRFG------------------
-GKSVEDQ--RILESSEVHNTTN--------
---DGDHGGEGLDEAK--SGNVEND----
TCEAI--DIGVVSPANGSG---------
RNHTGADIVD--------KVTSTITTA----ESLTSEQQKSELRDECEVFNHEDGERKLDDS-----------
-------------TNDGD------------RGSEGL---------------DE-AR-----------------
-SGDVEN-DNC------EAIDN--GVISPADGSG---------------------------------------
-----RNRTDADNN------------------------------VVKVTSTSATGE-----------------
-------------------------------------PLTSEELN----SE----LKVTKASPHS--------
--------LFKDL-------DKSSKPGR-------------KGIS-K-KSRKTLKKTSNVVEPSHC-------
-----------------SATKVKSAAN--------------KR-------------------------KPQ-
NPTTNDKDG-GKEGVNN----AKSDDVVR---------------DE----------CVGIND-----------
----DVI------------------SPVDGCGR-RHSGTD---------------------------------
-------------------GVGKLTSTDATKES-L-----TS-------------------------------
--------------------------EKRKGRVKETKTSL---------------------------------
--PSKDLNNS-----
SKPGKKANS---KKS------------
-------------------------ER--
--TLKT-DL----------------
--QAAE---

---------ENF---

---PCESEENLSWGNTKR---
-----KIDFDVE---
--------------------VTPDNKV--
---KKQ-K-
---TNA-----------------------
----VSTDSLGQ--------------------------KRSR-SG----------------RVLVSSLEY-W-
------RNQIPVYDL---SRKLIEVKG----
-------GH-ET-N--------------------------------PTP-SKG--------------------
----------KGS--
-YRRK--

---PRS-

>Camelina_sativa_XP_019101020.1

MKKKIAMTTK--------------------SKPQSLSTRR----------------------SPPRT------
------------------RS------------KPLPETNPSPRTRSKPQ--------------------
PEPN---P-------------------SP-----------RTH-----------------------------
PKA--
--LP-----
---KLNFS-----------P----
FPP---------------------TPASL--------------------------GAI-----------TPI-
--------------------GT-RKY---
---VTLSEWWLTR-KG-K--------
DKEKKALCIT---------------GFE---
---S-DV-RLF----------------------

SSGRILKRHNSVTLESVDGITISISGFI-------NRARSIEN-GVSEE--------VCNH-FLLGF--
PFNW-------------EDYN--
-------EEIVV---

--DEDR-------------------

-----------------------G---------------------------------FV-VSFDDV-------
----
-----------------PVNRI-----------------------QD-LSFV---------------------
----

---------------------------DGCLK--------------DKILV----------------------
------------DVV--
------S-------------------------------SLRDMV-----------------------------
----
--CP-KSDEKKKSA---------------------
--------------VVV--
--------------------------------------DD-------------------------------E-

-------------------------------SLVSSA--------------------------------------
-------------VVVGVKTRAM-RRR-DEF-E--S---------------------------------------
-----------------------CIEKRSVLH---
---KVYKE-
---EEE-----------------------

--ISL--------

---------------------------VVSFTLFFNSHFV-----------------------------------
----

>Capsella_grandiflora_2240s0051.1.p

----MAEH---------------------R---------------------------------EPNL--------
--------------------------------------DD----------------------------------
D---G--------------------------------SK------------------------------SYF---
----

----------------RRT--
---VVLRDWWLVK-CPVE---------FEG-
KRFGVA----------------GIE-------------------------------DS------------LE---
---------------------------------------SR-AV-RVF---------------------------
----
TSSPIIKALDVFTLLSSDGIYITLRGFL--------NKERVVKN-GFTPE---------ISRE-FVFGF--
PPCW-------------ERIV--
-------TNCSGGGASLGT-DTTTV------------------------------------PSTVA---------
----
-----------------------------RACYPILSPC------------KNNK--------------------
------QNI-----------------EDSSAE---
--GRDESIVPDM-----------NM-------------------------------------GHMAEI-------
-----------INTKDG-------------------------------SR-------------------------
------------AKDRNTARR-------------------------KSLHLQTKSG-------------------

-GKNAEQE--KILESSKVRNTTS--------
---DGNHGTEELHKGK--TGDVEKD----
ECETINNEDEERDLDEGKLQ-NLTTIDGDLGSECLDKAKSGDVEQDYCEAIDNGVISSADGCG---------
KNQTGADNVV--------RVTSTSISG----ASLTSEQQKGELRDECEAINNRDDKWKLEES----KLQNLT-
-------------TNDGD------------HGSEGL---------------DK-AK-----------------
-SCDVEK-DES------EAVNN--GVISLVDGCG---------------------------------------
-----RKRTDAD-N------------------------------VDKVTSTSATGE-----------------
-------------------------------------SLTSEQQE----AE----HEVTAASPHS--------
--------LFQDL-------DKSSKPGK-------------KGKSKK-SKRKTLKNTGN--------------
----

PAKKGNS---KKS--------------
-----------------------EK--
--TLKR-DL------------------
--HAAE-----

-------ENF---

-ACESEENLSWGNTKR---
---KIDFDVE---
------------------VTPENKV--
---KKQ-K---
---TKA-------------------------
--VSTDSLGQ--------------------------KRSR-SG----------------RVLVSPLEF-W---
----RNQIPVYDM---DRNLIQVKD------
-----GH-DS--------------------------------------SQG----------------------
--------KGS---
KSRK---

--PRS--

>Capsella_rubella_0002s0640.1.p

MTK---TTAK--------------------SKSQSLSAPRS---------------------SPPRT------
------------------RS------------KTLPESS---------------------------------
N---P-------------------SP-----------RTH-----------------------------PK--
----
---P---------
---KRNFS-----------P----LPR--
-------------------TPFSL--------------------------GAI-----------TPI------
---------------GT-RKD--
--VTLSDWWLTR-KG-K--------
EKKKGSLCIT---------------GFE------------------------------SS------------

KH---GS-EM-RLF---------------------

SSGTIVKRHNSITLEAIDGITISISGFI-------NRSRSLQN-GISNE--------VT--------------
----

-
>Capsella_rubella_0006s0132.1.p


```
-------------------------------------------------------------------------
----MAEH--------------------R--------------------------------EPNL--------
-------------------------------------DD---------------------------------
D---G-------------------------------SK-----------------------------SYF---
-------------------------------------------------------------------------
-------------------------------------------------------------------------
-------------------------------------------------------------------------
-------------------------------------------------------------------------
----------------RRT------------------------------------------------------
----------------------------------------VVLRDWWLVK-CPVE--------FEG-
KRFGVA---------------GIE------------------------------DS------------LE---
--------------------------------------SR-AV-RVF--------------------------
---------------------------------------------
TSSPIIKALDVFTLLSSDGIYITLRGFL-------NKERVVKN-GFTPE--------ISRE-FVFGF--
PPCW-------------ERIV----------------------------------------------------
-------TNCSGGGASLGT-DTSTV----------------------------------PSTVA---------
-------------------------------------------------------------------------
----------------------------RACYPILSPC------------KNNK-------------------
------QNI----------------EDSSAE------------------------------------------
--GRDESIVPDM----------NM------------------------------------GHMAEI-------
-----------INTKDV------------------------------SR------------------------
------------AKDRNTARR------------------------KSLHLQTKSG------------------
-GKNAEQE--------------------------------------------KILESSKVRNTTS--------
---DGDHGMEELHKGK------------------------------------------TGDVEKD----
ECETINNEDEERELDESKLQ-NLTTIDGDLGSEGLDKAKSGDVEQDYCEAIDNGVISSADGCG---------
KNQTGADNVV--------RVTSTSITG----ASLTSEQQKGELRDVCEAINNRDDKRKLEES----KLQNPT-
-------------TNDGD------------DGSEGL---------------DK-AK-----------------
-SCDVEK-DES------EAVNN--GVISLADGCG---------------------------------------
-----RKRTDAD-N------------------------------VDKVTSTSATGE-----------------
-------------------------------------SLTSEQQK----TE----HEVTAASPHS--------
--------LFQDL-------DKSSKPGK-------------RGKSKK-KSRKTLKNTGNVVEPSNC-------
-----------------LEAKVKSAEN--------------KR-------------------------KLQ-
NLTTNDKDR-GKKGLNN----AKSNDVER---------------DD----------CVAINE-----------
----EVI------------------SPVDGCAR-RHPGTN---------------------------------
-------------------RVGKLISKKATKES-L-----TS-------------------------------
--------------------------EQRKGRVKETKTSLRS-------------------------------
--------------------------------------------------------LSKDLNNS-----
SKPAKKGNS-------------------------------------------------KKS------------
-------------------------EK----------------------------------------------
--------------------------------------------------TLKR-DL----------------
------------------------------------------------------------------HAAE---
-------------------------------------------------------------------------
---------ENF-------------------------------------------------------------
-------------------------------------------------------------------------
-------------------------------------------------------------------------
---ACESEENLSWGNTKR-------------------------------------------------------
-----KIDFDVE-------------------------------------------------------------
--------------------VTPENKV----------------------------------------------
-------------------------------------------------------------------KKQ-K-
-----------------------------------------------TKA-----------------------
----VYTDSLGQ--------------------------KRSR-SG----------------RVLVSPLEF-W-
------RNQIPVYDM---------------------------------------------DRNLIQVKD----
-------GH-DS--------------------------------------SQG--------------------
----------KGS------------------------------------------------------------
-KSRK--------------------------------------------------------------------
-------------------------------------------------------------------------
-------------------------------------------------------------------------
-------------------------------------------------------------------------
-------------------------------------------------------------------------
-------------------------------------------------------------------------
```

---PRS-

>Cardamine_hirsuta_CARHR053810.1

----MATK--------------------SKIRSLSAHR----------------------TSPRS--------
----------------RF------------KSLPESNPS----------------------------------
-P--------------------------------FTFT----------------------------RAS----
----
---SP------------
--GTNG-------------------------
----------------TPFSL--------------------------GAI-----------TPI---------
------------GN-LKS---
---------------------------------------VTLSDWWLIK-KG-----------NE-KTLCIS-
--------------GFE-------------------------------S------------KG----------
-------------------------------GS-EV-RLF---------------------------------
--------------------------------------SSGTITKRHDSVTLEAIDGITICISGFI-------
DRSRSLQN-GISNK--------VCNR-FLLGF--PYDWNE-----------EEKE------------------
---EEGTV---------------------------

------------EEKK------------------------N-V----------------D-------------
----
---------------------VSFDDI--
---PVNRL-----------------
------QD-LCFL--

---DGCLK-------
-------DKILD----------------------------------DVV------------------------
--S-------------------------------
SLRDLV---

--CP-KLDKKCEKS------------------------------------RIGGG------------------
----------------------------------DESLDS--GM-----------------------------
DD-------------------------------E-------------------------------SLESQ---
---VVGVKTRGMLRQR-QD--E-D---
---SIGKIVRV--------

-----------------------------TTSKK---------------------------------------
----------RR---

>Cardamine_hirsuta_CARHR210840.1

----MSDH--------------------Q--------------------------------EPNL--------
-------------------------------------DG---------------------------------
DGANS------------------------------CSS-----------------------------SSF---
----

----------------QRT--
--VVLRDWWLIK-CSNE--------FEG-
KRFGVA---------------GTE------------------------------TS------------FE---
--------------------------------------SR-AM-RVF--------------------------
----
TSSPIIKALDVFTLQASDGLCITLRGFL-------NKERVFKN-GFKPE--------ICRE-FIFGF--
PPCW-------------ERIC--
-------NDCFQGD----S-DINTI----------------------------------D-------------
----
----------------------------KACSPILSPC------------KFNR-------------------
---------------------------NPAE--
--SRDHSTVTET----------N---------------------------------------IAEI-------
------------NSKDG------------------------------SR------------------------
------------AV-----RR------------------------KSLRLQPKSG------------------
-VNSAKGE--RKLESSKVQNSTN--------
---GGDHGSEGLSKAK--SSDVEED----
ECEAI--
---NKEDSY----DS----KVQNC---
------------TSDED------------HGGEGL---------------DE-AK------------------
SSDVEK-DEC------EAIND--EAISP----G--
----RKQNGAD-N------------------------------VDKVTSVSASG-------------------
----

-----------------------------ES-L-----TS---------------------------------
------------------------EQRKGKRKGTKTSLHS---------------------------------
--LSKEINNS-----
SKPGKNRKS---KKS------------

---DS----------------

NVVEPMN--
--------------HSESE--

------------EAEEDLSWGKTKR--
------------KIDFDVE--
---------------------------VTPEKES---------------------------------------
----
-KNN--V----------------
-----------VSTDSLGQ--------------------------KRSR-SG----------------
RLLVSSLEF-W-------RNEIPVYDT---
DRNLIQVRE-----------GS-DT-NSK------------------------------SAP-SKG-------
-----------------------KGS---
--------------NSLK---

---------PRN---

>Conringia_planisiliqua_CP11_g33626_DN3_SP0_c

--M----

---------------DRT---

--------------------------------R-AM-RVF---------------------------------
--------------------------------------TSSPIIKAFEVFTLQASDGVCIILRGFL-------
NKERVVQS-GFIPE--------ISRE-FIFGF--PPFW-------------EQIC------------------
---NNCFRGV-PDAT-GFNTL--------------
--------------------PSVIG--
--KASRPILSPC-
-----------NNTR-------------------------GNL----------------VDCSAE--------
------------------------------------SRDRSIVTEK----------N----------------
-----------------------TAEI------------------NNGRSG----------------------
-------GSR------------------------------------AIDKNTASK------------------
------KSLRLQSKSG-------------------GKSSQDE-------------------------------
-------------KKLEVSKVQNITN-----------VGDHVREGLNKAKG----------------------
------------------CNDDVEED----EREAI--------------------------------------
----
------GNEGNEKKLDES----EVHNG---------------TNDED------------HGSEGS--------
-------DK-AK------------------NNDVEK-DEC------EVIYN--DVTSLADGCG----------
----------------------------------KKHSGAD-N------------------------------

VDKVTSMSATGE--
LLTSEQGK----GE----LGVTRASP---

HSGTD--S-KKLKSKNATKES-
L-----TS---
EQRKGKLKVTKTTVHS---
------------------------------KSKDVSNS-----RKPGRKGKS---------------------
----------------------------KIS-------------------------------------EN---
----
--------------------TLKG-DC--
------------------------------------DVVEPMN------------------------------
--HSASK------------------

--VKEAEENMSGGKINR------------
--KIDFDEELM----------------
---PPLLCNA----KVTPDKDA---

-------------------------------------KKQ-K-------------------------------
-----------------TNA---------------------------VSADSLGQ------------------
--------KRSR-SG----------------RVLVSSLEY-W-------RNQIPVYDM---------------
------------------------------DRNLIQVNE-----------GH-ET-I----------------
----------------STP-SKGS---FF----------LKAVFDKRGLH--CKSN----TEFCLALV-----
-------LDRKGIEFS--------------------------KAKKMK-------------------------
----

---------------------------------------IKHTASNYFG------------------------
----

--------------NY---

>Euclidium_syriacum_0020s0140.1.p

----MVDP--------------------W--------------------------------EPNL--------
-------------------------------------DD---------------------------------
D-----------------------------------CS-----------------------------SYF---
----

----------------QKT--
--VIITDWWLIK-CSNE--------FNG-
KRFGVA---------------GTEIT---------------------------DSF------------DQ---
--------------------------------------KR-AI-RVF--------------------------
----
RSSPIIKAINVFSLETSNGVSIILRGVL-------NKERVVKS-GFNLE--------ISRE-FIFGF--
PPLW-------------EQIC--
-------NKWFEGI-SLSN-DIDTV----------------------------------PSSIII--------
----
---------------------------DKARYSVLSPCK----------SKNTK-------------------

```
------RNV----------------EDSVGK------------------------------------------
--NRDKNTVTET----------N---------------------------------------KVKV-------
------------NDKDGRS--------------------------VGSR------------------------
------------ARDENNARR------------------------KSFRLRSKP-------------------
-------------------------------------------------------------------------
-------------------------------------------------------------VEEE--------
-------------------------------------------------------------------------
-------------------------------------------------------------------------
-------------------------------------------------------------------------
---TEF------EVLDN--EVD---DGCG--------------------------------------------
IKHTDDA-------------------------------------------E----------------------
--------------------------------SMAFEQRK----DE----PKVTT------------------
----------------------R-------------AALR-K-KS----------------------------
-------------------------------------------------------------------------
---------------------EN---------------DQ----------SVVV-----------------
EPM---------------------------IHTTED-------------------------------------
---------------GREVEKSKNV-----------TT-----------------------------------
----------------------EQGKGEVKVTK----------------------------------------
----------------------------------------------------------EL-----
DKRSKSGKS-------------------------------------------------KR-------------
-------------------------------------------------------------------------
-------------------------------------------------------NV----------------
------------------------------------------------------------------
VVTEPMN------------------------------------------------------------------
--------------HSRPE------------------------------------------------------
-------------------------------------------------------------------------
-------------------------------------------------------------------------
----------VKQAEKILSMGETKR------------------------------------------------
------------KIDFDAE------------------------------------------------------
---------------------------LTPEKKS---------------------------------------
-------------------------------------------------------------------------
DKKQKK------------------------------------------------SDA----------------
------------SSSSIGS-------------------------FNRSR-SG----------------
RLLMSPLEF-W-------RNEIPVYDM---------------------------------------------
DRSCIKVKD-----------GD----N--------------------------------ETP-SKAG------
-----------------------KRS-----------------------------------------------
--------------DSRK-------------------------------------------------------
-------------------------------------------------------------------------
-------------------------------------------------------------------------
-------------------------------------------------------------------------
-------------------------------------------------------------------------
-------------------------------------------------------------------------
-------------------------------------------------------------------------
---------PRR-------------------------------------------------------------
-------------------------------------------------------------------------
-------------------------------------------------------------------------
-------------------------------------------------------------------------
-------------------------------------------------------------------------
-------------------------------------------------------------------------
----------------------------
>Euclidium_syriacum_0048s0039.1.p
-------------------------------------------------------------------------
-------------------------------------------------------------------------
-------------------------------------------------------------------------
----MATK--------------------SKSESVSM------------------------HPPRT--------
----------------RS------------GYVPEPNSS----------------------------------
-P--------------------------------RTR-----------------------------SGC----
-------------------------------------------------------------------------
-----------------------------------------------------------VP------------
--------------------------------------------ESKSA-----------PRTDRVAR-----
```

----------------TLFPP--------------------------DIV-----------TPV---------
------------GT-LKS---
---------------------------------------VVLDEWWL-K-KG-----------KE-KGLCVS-
--------------GFE-------------------------------I------------KG----------
-------------------------------GG-AI-RKF---------------------------------
--------------------------------------SSGAITKRHDSNTLETIDGITVTLSGFL-------
DRTRSLQN-GISFD--------LCNR-FNFGF--PYDWN------------EDED------------------
---ES------------------------------

-----------VETKN------------------------KAF----------------G-------------
----
------------------FD-FSFDDI--
---PVRNV-----------------
------NDLLLTS--

---NSSLR-------
-------SKILD----------------------------------DVV------------------------
--E-------------------------------
GLRGFA--Y--
---------------GS--
--TQ-ESEKECEKS------------------------------------GMD--------------------
--------------------------------DND-----------------------------------
YNGDD-------------------------------E-------------------------------
SLVPR--VVGAKTRSMLKRV-
HET--

---SKK-----------------------------
--------------------KRS--

>Eutrema_heterophyllum_scaffold130477_cov169.67_1

----MADH--P--------
-------------------------------------DG---------------------------------
D------------------------------------S-----------------------------SSF---
----

----------------QRTVS-----------------VSGL--------LMHFLVF----------------
-------------------DSISMLDLFFSSFWVY---VKVILRDWWLIK-CPKE--------FEG-
KRFGVA---------------GSEIA----------------------------ASPRLALSFRSGLLNR---
--------------------------------------TR-AM-RVF--------------------------
----
TSSPIIKAFDVFTLQASDGICITLRGFL-------NKERVVKS-GFIPE--------ISRE-FIFGF--
PPCW-------------EQIC--
-------NNCFGGV-PLGT-DVKTS----------------------------------PSVID---------
----
----------------------------KACCPILSPC------------KNSK-------------------
------GNL----------------EDSRAE--
--RRDKSTVTEK----------N---------------------------------------TRVI-------
------------NDKDGHS--------------------------GGST------------------------
------------AINKKTARK------------------------KSLRLQSKSG------------------
-GKSAEDE--RELELGNVQHTTN--------
---DGDHGSEGLNKAK--SRDVEKD----EF--

--KATDNEGNGRKLDES----KVQNR-------
--------TNDGD------------HGSGGL---------------DK-AK------------------
SSDVEK-DEC------EVINN--EVISPVDGCA--
----KKHTGAD-N---------------------------------VISMSATGE------------------
------------------------------------SLTSEQGK----GE----LKVTEASPHS---------
-------LFEDL-------DKSSKPGK--------------------KSRKTLKSDGNVVEDVNN--------
----------------SGTKVKSAKN--------------KRKL--------------------DLSNIQ-
HHTTNDGDH-GREGLNK----AKGNDVEK---------------DE----------CMAINN-----------
----EVI------------------SPVDGCGK-RHSSTD---------------------------------
-------------------S-KNLASKNATKKS-L-----TS-------------------------------
--------------------------EQRKGKLKVTKTSLHS-------------------------------
--ISKDLNNS-----
SKPGKKGKS---K--------------

--TLKG-DW----------------

NVVEPMN--
--------------HSGSK--

----------VKEAEENLSGRKTHR--
------------KIDFDEE--
---------------------------VTPDKEA---------------------------------------
----
-NKQ-K--TNA----------------
-----------VSADSLGQ--------------------------KRSR-SG----------------
RVLVSSLEY-W-------RNQIPVYDM---
DRNLIQVNE-----------GH-ET-N--------------------------------SAP-SKG-------
-----------------------KGS---
--------------DSRK---

---------PRS---

>Eutrema_heterophyllum_scaffold455_cov155.49_1

----MTTK--------------------SKSKSLSARR----------------------SSPRT--------
----------------RS------------GAVAEPISS----------------------------------
-P--------------------------------CIF-----------------------------SRF----
----
---VP------------
--EPNSS-----------PGTDEIPR-----
----------------TSFSF--------------------------GAV-----------TPV---------
-----------AGT-LKS---
---------------------------------------VSLSDWWLTK-KT-----------NQ-KGLCVT-
--------------GFE-------------------------------S------------KG----------
-------------------------------GS-EV-RLF---------------------------------
--------------------------------------SSAAISERHDSTTLETFDGITVSISGFF-------
DRSRTLQN-GFSSE--------VCNR-FLLGF--PYNW-------------KDHD------------------
---EE------------------------------

-----------AEEKK------------------------Q--------------------------------
----
------------------FS-VSFDEI--
---PVNRY-----------------
------QDLLFSS--

--YH-------
-------NEILA----------------------------------DVV------------------------
--S-------------------------------
SLRDLVCPS--

--TE-KSDKKCKKS------------------------------------RMSND------------------
----
DD-------------------------------DK------------------------------SVVPR---
---VVGVKTRGMLKRR-EDS-E-GE--
---AFSVVERVHT--------

-----------------------------TSNKK---------------------------------------
----------NRS--

-------------------REKTKR--

>Eutrema_salsugineum_v10015290m

----MADH--------------------R--------------------------------EPNS--------
-------------------------------------DG---------------------------------
D------------------------------------S-----------------------------SSF---
----

----------------QRT--
--VFLGDWWLIK-CSKE--------FEG-
KRFGVA---------------GYEIA----------------------------AS------------VE---
--------------------------------------TR-AM-RVF--------------------------
----
TSSPIIKAFDVFTLQASDGTCITLRGFL-------NKERVVKS-GFLPE--------ISRE-FIFGF--
PPCW-------------EQIC--
-------NNCFGGV-HLGT-DINNA----------------------------------SSLID---------
----
----------------------------KACSPVLSPC------------KNSK-------------------
------GNL----------------EDSRVE--
--TRAKSTVTEK----------N---------------------------------------TTVI-------
------------NDKDGHS--------------------------DGSI------------------------
------------ARYTKSARK------------------------KSLRLQSKPG------------------
-GKSAEEE--RELELSKIQNTTN--------
---DRDHGSEGLNKAK--SRDVEKD----EF--

--EATGNEGSEAKLDES----KVQNR-------
--------TNDGDH-----------HGSGGL---------------DK-AK------------------
SSDVEK-YEG------EVINN--EVISSADGCG--
----NKHACAD-N------------------------------VDKVTSMSATGE------------------
------------------------------------SLTSEQGN----GE----LKVTRASPQS---------
-------LFEDL-------DKSRKPAK--------------------KSRKTLKSDGNVVEDVNH--------
----------------SGTKVKSAKN--------------KRKL--------------------DVSKGQ-
HPTT------SSEGLNK----AKGNDVEK---------------DE----------GTAINN-----------
----EVV------------------SPVDGYG--RHSGTD---------------------------------
-------------------S-KKLTSENATKES-L-----TS-------------------------------
--------------------------EQQKGKLNVTKTSLHS-------------------------------

--MSKDLNNS-----
SKPGKRGKS---K--------------

--NLKG-DW----------------

NVVEPMN--
--------------HTGSK--

----------VKEAEENLSGGKTNR--
------------KIDFDEE--
---------------------------VTPDKEV---------------------------------------
----
-KRQ-K--TNA----------------
-----------VSADSVGQ--------------------------KRSR-SG----------------
RVLVSSLEY-W-------RNQIPVYDM---
DRNLIQVNE-----------GHDET-S--------------------------------STP-SK--------
----

>Eutrema_salsugineum_v10023956m

-----MTK--------------------SKSQSLSARR----------------------SSPRT--------
----------------RS------------GAMPEPISS----------------------------------
-P--------------------------------RTS-----------------------------SRF----
----
---VP------------
--KLNSS-----------PRTNEISR-----
----------------TPFSF--------------------------GAV-----------TPI---------
-----------AGK-LKS---
---------------------------------------VSLSDWWLTK-KT-----------NQ-KGLCVT-
--------------GFE-------------------------------L------------KG----------
-------------------------------GS-EA-RLF---------------------------------
--------------------------------------SSGAISRRHDSITLETFDGLTVCISGFI-------
NRSRTLQN-GFSSE--------VCNR-FLLGF--PYNW-------------EDNH------------------
---DE------------------------------

-----------EAEKK------------------------KQ-------------------------------
----
------------------F--


```
-------------------------------------------------------------------------
-------------------------------------------------------------------------
-------------------------------------------------------------------------
-------------------------------------------------------------------------
-------------------------------------------------------------------------
-RR-EEYKKCGKS------------------------------------RMDDD-------------------
-----------------------------------------------------------------------
DDD-------------------------------E-------------------------------CLVPR--
--------------------------------------------------VVVVKTRGMLRRR-EEN-E-T--
-------------------------------------------------------------------------
-------------------------------------------------------------------------
------------------------------RDKK---------------------------------------
-----------------------------------------------------
RCCREQRAMLIHGGGLATSVRSNQNGSSTIFRKV--------EYT--
LKIIDEDGDTFAKRAEMYYRKRPEIVNFVEEAFRSYRALAERYDHLSR-ELQSANRTIA--------------
-TAFPEHVQ-----------------------FPLE---DDDDNENED----
PQKPPKHLHLIPKGSNIPEVPEIP-KNEFRSQSMMLSRKGPAGLK--------RTVSSA----
QAKREVAIVSSGLSKEEGLEEIDKLQKGILALQTEKEFVRSSYEQSYERYWDLENEVTEMQKRVCSLQDEFGL
GAAIDDSEAKTLMASTALSSCKDTLAKLEEKQKQSV--EEAEIEKERIDTAK----ERFDA---------
LRNRFNKPEINDHGEVIKTEKE------------
KDVVQESSYESEREDSNENLTVVKLAEKIDDLVQRVVSLETDATSHTALVKT-------LR--
SETDDLHEHIRGLEEDKASLVSDSTDMKQR-------IIILEDELSKVRKLYQ----
KVEGQNKSLQNQFKEANRTAEDLSGKLQGVKMDEDVEGAGIFQELQVVSGSEDS----
KSISKETERRSSVEEQKKDDIVVKESE---
GAQEEKPEIKDSFALSETASTCFGTEGEELVTEDEDEETPNWRQLLPDGMEDREKVLLDEYTSVLRDYREVKR
KLGEVEKKNREGFFELALQLRELKNAVAYKDVEIHSLRQKLDTHGKDSPHQVEGSNQ--------------
LEQDQGQRE-SVSISISPTSNFSVSTTPHHQVGEMKRT----KSNEVRVKFADVDDSPRTNIPTVGDKVRA-
DIDAVLEENLEFWLRFSTSVHQIQKYQTTVQDLKSELLKLRIESKQQQESPRSSS-NNT---SEAKPIYRHL-
-REIRTELQL-WL------ENSAVLKDE-----
LQGRYASLANIQEEIARVTAQSGGTKISDSEISGYQAAKFHGEILNMKQENKRVSSELQSGL-DRVRALK---
---TDVERILSKLEDDLGISSASEARTTP-SRSS-----------------------------SSGRPRI---
------------------------------------PLRSFLFG----VKLKKHRQQKQTASS----------
-------------------------------------------------------------------------
-------------------------------------------------------------------------
-------------------------------------------------------------------------
--------------------------------LFSCV------------------------------------
-------------------------------------------------------------------------
---------------------------SPS-------------------------------------------
----------------------------------------------PALQKQS--------------------
-------------------------------------------------------------------------
-------------------------------------------------------------------------
-------------------------------------------------------------------------
-------------------------------------------------------------------------
---------------------SYVRQP---------------------------------------------
GKL-----------------------------------------------------P-----------E
>Eutrema_yunnanense_scaffold1887_cov115.11_1
-------------------------------------------------------------------------
-------------------------------------------------------------------------
-------------------------------------------------------------------------
----MA-------------------------------------------------------------------
-------------------------------------DG---------------------------------
D------------------------------------S-----------------------------SSF---
-------------------------------------------------------------------------
-------------------------------------------------------------------------
-------------------------------------------------------------------------
-------------------------------------------------------------------------
----------------QRT------------------------------------------------------
----------------------------------------VVLRDWWLIK-CSKE--------FEG-
KRFGVA---------------GSEIA----------------------------AS------------VE---
--------------------------------------TR-AM-RVF--------------------------
```

TSSPIIKAFDVFTLQASDGICITLRGFL-------NKERLVKN-GFIPQ--------IYRE-FVFGF--
PLCW-------------EQIC--
-------NNCFGGV-P----DIKTV----------------------------------PSVID---------
----
----------------------------KACCPILSPC------------KNSK-------------------
------GNL----------------EDSPAE--
--RRDQSTVTEK----------N---------------------------------------TRVI-------
------------NDKDGHS--------------------------GGST------------------------
------------ARNTKPARK------------------------KSLRLLSKSG------------------
-GKSAEDE--RELELGNVQHTTN--------
---DGDHGSEGLNKAK--SRDVEKN----
EFKATDNEANERKLDESKVQ-NRTN-
DGDHGSGGLDRAKSSYVEKDECEVINNEVISPARNKKTKRKKSLRLQSKSGGKSAEEERKLELGEVQNTTNDG
DHGSEGLNKAKSRDVEKNEFKATDNEGNERKLNES----KVQNR---------------TNDGD---------
---HGSGGL---------------DK-AK------------------NSDVEK-DEC------EVINN--
EVISPADGCD--KKHTGTD-N----------
--------------------VDKVTSMSATGE---
-------------SLTSEQGK----GE----LKVTRASPHS----------------LFEDL-------
DKSSKPGK--------------------KSRKTLKSDSNVVEDVNN------------------------
SGTKVKSAKN--------------KRKL--------------------DGSKVQ-HHTTNDGDH-EREGLNK-
---AKGNGVEN---------------DE----------CMAINN---------------EVI-----------
-------LPVDGCGK-RHSSTD---
-S-KKLASKNATKES-L-----TS---
--------EQRKGKLKVTKTSLHS---
--------------------------------------MSKDLNNS-----SKPGKKGKS-------------
------------------------------------K------------------------------------
----
----------------------------TLKG-DW--------------------------------------
--NVVEPMN----------------------
--HSGSK----------

--VKEAEENLSGGKTNR----
--KIDFDEE----------

VTPDKEA--
---KTQ-N---------------------
---------------------------TNA---------------------------VSTDSLGQ--------
------------------KRSR-SG----------------RVLVSSLEY-W-------RNQIPVYDM-----
--DRNLIQVNE-----------GH-ET-N------
--------------------------STP-SKG------------------------------RGS-------
---DDSRK---------------

---PRS---------------------

>Eutrema_yunnanense_scaffold81_cov138.25_1


```
----M---K-------------------SKSKSLSARR----------------------SSPRT--------
----------------RS------------GAVPEPISSPRTRSGAVPEPISSPRTRSG------AVPEPN-
SSP--------------------------------RTF-----------------------------SRF---
-------------------------------------------------------------------------
------------------------------------------------------------

VPQSNSSARTFSRFVPDSNSSPRT------------R--SRAV--------------PEPNSS----------
-PVPDEIPR---------------------TPFSF--------------------------GAV---------
--TPV--------------------AGT-LKS-----------------------------------------
-----------------------------------------------------VSLSDWWLTK-KT-------
----NQ-KGLCVA---------------GFE-------------------------------S----------
--KG-----------------------------------------GS-EV-RLF-------------------
----------------------------------------------------

SSAAISERHDSTTLETFDGITVCISGFI-------NRSRTLQNGGFSSE--------VCNR-FLLGF--
PYNW-------------KDHD----------------------------------------------------
-------EEE---------------------------------------------------------------
-------------------------------------------------------------------------
--------------------------------------------------AEEKK------------------
------K----------------------------------------------H-------------------
---------------------------------------------------------FS-VSFDEI-------
-------------------------------------------------------------------------
-----------------PVNRL-----------------------QDLLFTS---------------------
-------------------------------------------------------------------------
-------------------------------------------------------------------------
-------------------------------------------------------------------------
-------------------------------------------------------------------------
---------------------------SACLK--------------NEILD----------------------
------------DVV----------------------------------------------------------
------D-------------------------------SLRDLVCAS--------------------------
-------------------------------------------------------------------------
----------------------------------------TE-KSDKKCKKS---------------------
---------------GMGND-----------------------------------------------------
--------------------------------------DD-------------------------------E-
------------------------------SVVPR--------------------------------------
--------------VVGVKTRGMLKRR-EDN-E-GE-------------------------------------
--------------------A-SVVERVH--------------------------------------------
-------------------------------------------------------------------TTSKK-
------------------------------------------------KRS----------------------
-------------------------------------------------------------------------
-------------------------------------------------------------------------
-------------------------------------------------------------------------
-------------------------------------------------------------------------
-------------------------------------------------------------------------
-------------------------------------------------------------------------
-------------------------------------------------------------------------
-------------------------------------------------------------------------
-------------------------------------------------------------------------
-------------------------------------------------------------------------
-------------------------------------------------------------------------
-------------------------------------------------------------------------
-------------------------------------------------------------------------
---------------------------------------------------------RVKTKR----------
-------------------------------------------------------------------------
-------------------------------------------------------------------------
-------------------------------------------------------------------------
-------------------------------------------------------------------------
-------------------------------------------------------------------------
-------------------------------------------------------------------------
-------------------------------------------------------------------------
-------------------------------------------------------------------------
-------------------------------------------------------------------------
```

```
-------------------------------------------------------------------------
-------------------------------------------------------------------------
-------------------------------------------------------------------------
-------------------------------------------------------------------------
-------------------------------------------------------------------------
-------------------------------------------------------------------------
-----
>Leavenworthia_alabamica_maker14753.1
-------------------------------------------------------------------------
-------------------------------------------------------------------------
-------------------------------------------------------------------------
----MNDH--------------------Q--------------------------------ESNI--------
-------------------------------------DG---------------------------------
DGGYT-------------------------------YS-----------------------------SYF---
-------------------------------------------------------------------------
-------------------------------------------------------------------------
-------------------------------------------------------------------------
-------------------------------------------------------------------------
----------------QRT------------------------------------------------------
----------------------------------------VVLKDWWLIK-CSNV--------FEG-
KRFGVA---------------GLE------------------------------AS------------VE---
--------------------------------------SK-AK-RVF--------------------------
---------------------------------------------
TSSPIIKALDVFTLKASDGLCINLRGFL-------NKERVVQN-GFKTE--------ICRE-FIFGF--
PPCW-------------ERIC----------------------------------------------------
-------NDCFQGD----S-DLNTI----------------------------------H-------------
-------------------------------------------------------------------------
----------------------------KASSPILSPC------------KYCT-------------------
------ENL----------------DDIPAE------------------------------------------
--SRCKSTVTET----------N---------------------------------------ITEI-------
------------NSKDG------------------------------SR------------------------
------------P-------R------------------------KYLRQQSKSG------------------
-GKIA--------------------------------------------------QLSKVHNTTI--------
---DGDHGYKGFHKAK------------------------------------------SGNVDKD----
ECEVI--------------------------------------------------------------------
-------------------------------------------------NNQDKC----DS----EVQNC---
------------SNDED------------HGSEGL---------------VN-AK------------------
SSDVET-D-----------DN--EVISPADGCV----------------------------------------
----RKHSGAN-N------------------------------VDKVTSMSANGE------------------
------------------------------------SLTSDQHK----DE----LKLTTASPQS---------
-------LFKDL-------NKSSKP------------------R-K-KSDKTSKSGKNAVKPMNF--------
----------------SGTKVKSADK--------------KKKL--------------------DASKIQ-
NSTTNDGDH-GSECLIK----SKSNDVEK---------------DE----------CLAITE-----------
----------------------------DGSGN-KHSGTD---------------------------------
-------------------GVEKV-------------------------------------------------
-------------------------------KRKRPKPSLHS-------------------------------
--------------------------------------------------------LLKKINTS-----
SKPGKKKKS-------------------------------------------------MES------------
-------------------------EK----------------------------------------------
--------------------------------------------------T----------------------
-----------------------------------------------------------------------
MN-----------------------------------------------------------------------
---------HLES------------------------------------------------------------
-------------------------------------------------------------------------
-------------------------------------------------------------------------
-------E-EEDLSWGKTKR-----------------------------------------------------
-------NIEFDME-----------------------------------------------------------
----------------------VTPEKES--------------------------------------------
---------------------------------------------------------------------KMQ-
K------------------------------------------------TNV---------------------
```

------VSTDSLGQ--------------------------KRSR-SG----------------RLLVSTLEF-
W-------RNQIPVYDM---DRNLIQIKQ--
---------GG-DK---

>Lepidium_meyenii_scaffold1913.53

----MATKN-------------------SKSQSLSARR----------------------SPPRT--------
----------------RS------------KSLPEPNSS----------------------------------
-P--------------------------------CTC------------------------------------
----

-------------------------FRSS--------------LKPNFS-----------PGTDCILK-----
----------------TPYSV--------------------------GAS-----------TPI---------
------------GT-LKN---
---------------------------------------VSLSDWWLTK-KG-----------KE-KALSIT-
--------------GFE-------------------------------A------------KG----------
-------------------------------GT-EV-RLF---------------------------------
--------------------------------------SSVAISKRHDSTTLEAIDGITICISGFI-------
NRSQTLEK-GISNE--------VCDR-FLLGF--PYNW-------------KDYN------------------
---EEE-----------------------------

-----------EMEMN------------------------NHT----------------D-------------
----
------------------FS-ISFDDI--
---PVSRL-----------------
------QD-LSYL--

---EGSLR-------
-------KKILV----------------------------------DVV------------------------
--D-------------------------------
SLRDLV---

--FT-KADKQCEKS------------------------------------RI---------------------
----------------------------------D--------------------------------------
VD-------------------------------E-------------------------------SSVSM---
---VVGVKTRSMLRKR-EMN-E-A---
---SIVKRVQTM-------

------------------------------SKKK---------------------------------------
----------KR---

```
-------------------------------------------------------------------------
-------------------------------------------------------------------------
-------------------------------------------------------------------------
-------------------------------------------------------------------------
-------------------------------------------------------------------------
-------------------------------------------------------------------------
-------------------------------------------------------------------------
-------------------------------------------------------------------------
-------------------------------------------------------------------------
-------------------------------------------------------------------------
-------------------------------------------------------------------------
-------------------------------------------------------------------------
-------------------------------------------------------------------------
-------------------------------------------------------------------------
-------------------------------------------------------------------------
-------------------------------------------------------------------------
-------------------------------------------------------------------------
-------------------------------------------------------------------------
-------------------------------------------------------------------------
-------------------------------------------------------------------------
-------------------------------------------------------------------------
-------------------------------------------------------------------------
-------------------------------------------------------------------------
-------------------------------------------------------------------------
-------------------------------------------------------------------------
-------------------------------------------------------------------------
-------------------------------------------------------------------------
----------------------------------------
>Lepidium_meyenii_scaffold219.113
MSTGVCGKRVRYDDFF-GSSSSPTHKRFKSC--GLPISSSEIGSECDDPVGSLIR------------------
-------------------------------------------------------------------------
-------------------------------------------------------------------------
----MFPT--------------------M--------------------------------DPEFFFILVQ--
---------FFN-YFIRNVLR---------NTNNVFEET---------------------------------
KESLS-------------------------------SI-----------------------------SFN---
-------------------------------------------------------------------------
-------------------------------------------------------------------------
-------------------------------------------------------------------------
-------------------------------------------------------------------------
----------------
GESVGSWRDEEMTDGAKWVDRLVSEMSKAINIDDMRQRVVVILEALERIIKHNSDASKKRAIANQHRLSTENE
EKAKEVQHLKSLVGKYQEQVNKLEVVLRDWWLIK-CSNE--------FEG-KRFGVA---------------
GAE------------------------------ES------------VK------------------------
-----------------SR-AL-RVF-----------------------------------------------
------------------------KSAPIIKALDVFTLQASDGIFITLRGYL-------NKELVIKN-
GFKPE--------IARE-FIFGF--PPRW-------------ERIC---------------------------
--------------------------------NKCFEEV----------------------------------
-----------PLIIK---------------------------------------------------------
-----------------------------------------------------KASTPILSPC----------
--KYSK-------------------------ENL----------------EDNPAG-----------------
---------------------------SRDKTSLTET----------N-------------------------
--------------TAEI-------------------NKKDG------------------------------
SR------------------------------------ARDKITATR------------------------
KSLHLQSKSG-------------------GESTEDE-------------------------------------
-------RKLKSNEVKNTTN-----------DGDHGSIGMNKAK-----------------------------
-------------SGDVEED----EGNAI--------------------------------------------
-------------------------------------------------------------------------
RTEGNERKLGEC----KTKNR---------------SNDGD------------LGSEIL--------------
-DK-AK------------------SRDVKI-YEG------EVIDN--EVTLFKD-------------------
-------------------------------------------------------------------------
```

```
---SLT-------------------------------------------------------SLTSEQRT----
DE----LKVTRASPHS----------------LFKDS-------DKTSKPGK--------------QGTS-K-
KSAKILKSDSN------H------------------------SVNIVKSAGN---------------KRKL---
-----------------NASKVQ-NPTTNDGDH-DSKGLNK---------------------------------
-----------------------------------------------------------RYSGTD---------
-------------------------------------------SVGKLNS------------------------
--------------------------------------------------------------------------
--------------------------------------------------------------------------
--------------N-----SKPDKKGKS---------------------------------------------
-----KKS-------------------------------------VK---------------------------
-----------------------------------------------------------------------
NLKS-DG-------------------------------------------------------------------
----------------NVVTPMH---------------------------------------------------
------------------------------NSGSK---------------------------------------
--------------------------------------------------------------------------
--------------------------------------------------------------------------
--------------------------ANKAEGHLSWEKTNR---------------------------------
----------------------------KIDFDVE---------------------------------------
-------------------------------------------VTPDKP-------------------------
--------------------------------------------------------------------------
---------------------K-------------------------------------------------
ADA---------------------------ASTNSLGQ---------------------------KRSR-SG--
--------------RVLVATLEF-W-------RNQIPVYDR---------------------------------
-------------DRNIIQVKD-----------DL-ET-N---------------------------------
SIPSSKG------------------------------KVS----------------------------------
----------------------------DSRK------------------------------------------
--------------------------------------------------------------------------
--------------------------------------------------------------------------
--------------------------------------------------------------------------
--------------------------------------------------------------------------
--------------------------------------------------------------------------
--------------------------------------------------------------------------
-----------------------RRK------------------------------------------------
--------------------------------------------------------------------------
--------------------------------------------------------------------------
--------------------------------------------------------------------------
--------------------------------------------------------------------------
--------------------------------------------------------------------------
------------------------------------------
>Lepidium_meyenii_scaffold277.39
--------------------------------------------------------------------------
--------------------------------------------------------------------------
--------------------------------------------------------------------------
----MTTKN-------------------SMSQSLSARR-----------------------SPPRT--------
----------------RS------------KSLPEPNSSPRNRFKSL---------------------PEPN-
SSP---------------------------------CTR-----------------------------SKS---
--------------------------------------------------------------------------
-------------------------------------------------------------
LPEPNSSPRTRSKLLPEPNSSPRTCSKPLPEPSSLPRTYFRSS---------------LKPNSS----------
-PGTDGIFK---------------------TPYSL---------------------------GAV---------
--TPI---------------------GT-LKS------------------------------------------
------------------------------------------------------VSLSDWWLTK-KG-------
----KE-KALSIT---------------GLE--------------------------------S----------
--KG------------------------------------------GN-EV-RLF-------------------
-----------------------------------------------------
SSGAISKRHDSTTLEAIDGITVCISGFI--------NRSLTLQN-GISNE--------VCDR-FLLGF--
PYNW-------------KDYN-----------------------------------------------------
-------EEE----------------------------------------------------------------
--------------------------------------------------------------------------
---------------------------------------------------EMEKN------------------
```

------VHT----------------D---
---FS-LSFDDI-------

-----------------PVSRL-----------------------HD-LSYL---------------------
----

---------------------------EGSLK--------------KKILV----------------------
------------DVV--
------D-------------------------------SLRDLV-----------------------------
----
--FP-KADKECEKS---------------------
---------------RI---
D--------------------------------------VG-------------------------------
E-------------------------------SSASM------------------------------------
----------------VVGVKTRSMRRQG-EAN-E-A------------------------------------
------------------------SIVKRVKTM--

SKKK---KR------------------

>Lepidium_meyenii_scaffold60.13

----MAEH--------------------R--------------------------------EPNF--------
-------------------------------------DG---------------------------------
DGASS-------------------------------ST-----------------------------SYF---
----

----------------QKTV-------------------SEL--------IMHLLFLIRSAL-----------
---------------------IIALVVLLSKY------VKVVLRDWWLIK-CSNE--------FEG-
KRFGVA---------------GIE------------------------------ES------------VK---
--------------------------------------SR-AM-RVF--------------------------
----
NSSPIIKALDVFTLQASDGMFITLRGFL-------NKERLLEN-GFNPE--------ISRE-FIFGF--
PPCW-------------ERIC--
-------NKCFEEV---PSIIE---------

----------------------------KASSPILSPC------------KYSK-------------------
------KNL----------------EDNPAE--
--SRDKTSLTVT----------N---------------------------------------TAKI-------
------------NKNDG------------------------------SR------------------------
------------ARDKKTAFR------------------------KSLHLQSKSG------------------
-GESTEDE--SKLKLSEVKNTTN--------
---DEDHGSIGLNKAK--SGDVEED----
EGKAI--
---YSEDKEMKLRER----KTKNR---
------------SIDGD------------CGSEIL---------------DK-AK------------------
SRDVEI-YEG------EVIDK--EVTLPADGCG--
----RKR-GED-R------------------------------VGRVTTRNAARE------------------
------------------------------------SLTSKQRT----DE----LKLTRASPHS---------
-------LFKNS-------DKTSKPGK-------------QGTA-K-KSGKILKSANN------H--------
----------------SETIVKSAGN--------------KRKL--------------------NASKVQ-
NPTTTDGDH-DSEGLNK--
----------------------------------KYSGTG---------------------------------
-------------------SVGKLKS---

---N-----
SKPEKKGKS---KKS------------
-------------------------VK--
--NLKS-DC----------------

NIVTPMN--
--------------NSGSE--

----------ANRAEEHLSWEKTKR--
------------KIDFDVE--
---------------------------VTPDKP--

-----K--ANA----------------
-----------ASTDSLGQ--------------------------KRSR-SG----------------
RVLVASLEF-W-------RNQIPVYDL---
DRNLIEVKE-----------DC-DT-K--------------------------------STPPSKG-------
----

>Lepidium_meyenii_scaffold668.120

----MADN--------------------R--------------------------------EPNF--------
-------------------------------------EE---------------------------------
DVASS-------------------------------ST-----------------------------SFF---
----

----------------QKT--
--VVLRGWWLIK-CSNE--------FEG-
KRFGVA---------------GVE------------------------------ES------------VE---
--------------------------------------TK-AM-RSF--------------------------
----
KSSPIIKALDVFTLQASDGIFIILRGFL-------NKERLLEN-GFNRE--------IARE-FIFGF--
PPCW-------------ERIC--
-------NNCFKEA---PSLIE---------

----------------------------KASSPILSRC------------KYSK-------------------
------ENL----------------EDNPAE--
--SRDKTSLTET----------N---------------------------------------TAEI-------
------------NRIDG------------------------------SR------------------------
------------ARDKKTASR------------------------KPLPLQSKSG------------------
-RKSTEDE---RLGL----------------
--------HLDLNKA--

---TNR-------
--------SIDGD--
---------------------------RG--
RKR-GED-R------------------------------VDKVTTRSASSK----------------------
--------------------------------SLTSEQRT----DE----LKVARACPHS-------------
---LSKDS-------EKTSKPGK-------------QGTS-K-KSGKILKCDSN------H------------
------------SVNIVKSSGN--------------KRKL--------------------NASKVQ-
NPTTSDGDH-DSEGLNK--
----------------------------------RYSATD---------------------------------
-------------------SVRKQKS---

---Y-----
SKPEKKGKS---KKS------------
-------------------------VK--
--NLKS-DC----------------

NIVTPMN--
--------------NSGSE--

----------ANKAEEHLSWEKTKR--
------------KIDFDLE--
---------------------------VTPDKP--

-----K--ANA----------------
-----------ASTDSFGQ--------------------------KRSR-SG----------------
KVLMATLEF-W-------RNQIPVYDR---
DRNFIQVKE-----------DR-ET-N--------------------------------STPSSKG-------
-----------------------KVS---
--------------DSRK---

---------RRK---

>Lepidium_meyenii_scaffold96.171

-----------------------------MSQSLSARR----------------------SPPRT--------
----------------RS------------KSLPEPNSS----------------------------------
-P--------------------------------RTR-----------------------------SKP----
----

LPEPNSSPRTRSKPLPEPNSSLRTC--------------FRSS--------------LKPNSS----------
-PGIDGILK---------------------MPYSL--------------------------GAF---------
--TPI---------------------GT-LKT---
---VSLSDWWLRK-RG-------
----NE-KALSIT---------------GFE-------------------------------S----------
--KG---GT-EV-RLF-------------------

SSGAISKRHDSTTLEAIDGITICISGFI-------NRSQTLEN-GISNE--------VCYR-FRLGF--
PYNW-------------
KDYNGEEEIDKNVHIDFSISFDDIPVNRFQDRSQTLENGISNEVCDRFRLGFPCNWKDYNGEEEEE-------
----

---------------------------------EIEKN------------------------VHT--------
--------D--
--FS-ISFDDI------------------------

PVNRF-----------------------QD-LSFL--------------------------------------
----

----------EGSFK--------------KKMLV----------------------------------DVV--
--D----------
---------------------SLRDMV--

-----------------------CP-KADKECKKS------------------------------------
RI---D---------------
-----------------------VD-------------------------------E----------------
---------------SSVSM--
VVGVKTRGMIRQR-EAN-E-A--
--------SIGKIVPRM--
--SKKK---------------
----------------------------------KR-------------------------------------
----


```
-------------------------------------------------------------------------
-------------------------------------------------------------------------
-------------------------------------------------------------------------
-------------------------------------------------------------------------
-------------------------------------------------------------------------
-------------------------------------------------------------------------
-------------------------------------------------------------------------
-------------------------------------------------------------------------
-------------------------------------------------------------------------
-------------------------------------------------------------------------
-------------------------------------------------------------------------
-------------------------------------------------------------------------
-------------------------------------------------------------------------
-------------------------------------------------------------------------
-------------------------------------------------------------------------
-------------------------------------------------------------------------
-------------------------------------------------------------------------
-------------------------------------------------------------------------
-------------------------------------------------------------------------
-------------------------------------------------------------------------
-------------------------------------------------------------------------
-------------------------------------------------------------------------
-------------------------------------------------------------------------
----------------------------------------------------------------
>Moringa_oleifera_10005842
-------------------------------------------------------------------------
-------------------------------------------------------------------------
-------------------------------------------------------------------------
----MAKR--------------------AGRKSLIAKS-----------------------------------
-------------------------------------------------------------------------
DP-----------------------------------------------------------------------
-------------------------------------------------------------------------
-------------------------------------------------------------------------
--------------------------------------------EANQ-----------------ITR-----
----------------TPFTS--------------------------AAI-----------IPT---------
------------QS-IKY-------------------------------------------------------
---------------------------------------VLLHDWWLSR-AE-D------------KGLAVA-
--------------GFE-------------------------------S------------RG----------
-------------------------------KL-GV-RIF---------------------------------
--------------------------------------SSGIIAKRHDAVTLETVDGIRITISGLI-------
NRSRTHQN-GFPFE--------VCDI-FLFGF--PYDW-------------KDYA------------------
-----------------------------------------SQCYS---------------------------
-------------------------------------------------------------------------
-------------------------------------------------------------------------
-----------VESAE------------------------RDF----------------QLRKPGCFEF----
------------------------------------NMVLGSSGNT----------SGP--------------
---------------------GSLYDL----------------------------------------------
---------------------------------------------------PAAKI-----------------
------RDLLMSPDGG---------------------------------------------------------
-------------------------------------------------------------------------
-------------------------------------------------------------------------
-------------------------------------------------------------------------
------------------------------------------------------------WENCAE-------
-------SNLLD----------------------------------DV-------------------------
-------------------------------------------------------------------------
------------------------------------------------------------------SVHSNM-
--------------KEESPRT----------------------------DVNSILC-----------------
----KSAGNHKRA------------------------------------KVD-IKL-----------------
-----------------------------YRGSREIL-----------HTKHTATQE------------
LQNTG-------------------------------KSVLR-------------------------
```

```
ADILTPYA-----------------------------------------------------GVTTRNMTKLI-
KEQ-E-AN-----------------------------------LKNLLVE-----------
DENQLVTRGQRLAVR----------------------------------------------------------
-----------------------------------------------CNEWRGEIE-----------------
--------------------------------EEE-------------------------------------
TA-----------------------------------------------------------------------
-------------------------------------------------------------------------
-------------------------------------------------------------------------
-------------------------------------------------------------------------
-------------------------------------------------------------------------
-------------------------------------------------------------------------
-------------------------------------------------------------------------
-------------------------------------------------------------------------
-------------------------------------------------------------------------
-------------------------------------------------------------------------
-------------------------------------------------------------------------
----------------------------------------------------ETPG-----------------
-------------------------------------------------------------------------
------------------------------------------------------SGM-KHV------------
-------------------------------------------------------------------------
---------------------------------------------------EK--------------------
-------------------------------------------------------------------------
-------------------------------------------------------------------------
-------------------------------------------------------------------------
-------------------------------------------------------------------------
-------------------------------------------------------------------------
-------------------------------------------------------------------------
--------------------------------------------EA---------------------------
-------------------------------------------------------------------------
-------------------------------------------------------------------------
-------------------------------------------------------------------------
-------------------------------------------------------------------------
-------------------------------------------------------------------------
---------------------------------------------------------------
>Moringa_oleifera_10015199
-------------------------------------------------------------------------
-------------------------------------------------------------------------
-------------------------------------------------------------------------
----MAPT--------------------S--------------------------------APNC--------
--------------------------------HNNRDVG---------------------------------
D------------------------------------S-----------------------------SCF---
-------------------------------------------------------------------------
-------------------------------------------------------------------------
-------------------------------------------------------------------------
-------------------------------------------------------------------------
----------------QKT------------------------------------------------------
----------------------------------------VCLRDWWLIK-ADKD--------FEG-
KRLGIA---------------GVT------------------------------SA------------RD---
--------------------------------------LQ-PV-RLF--------------------------
---------------------------------------------
TSAPIVKRFDVFTLETADRICIVVNGCI-------NRARTSEN-GFSSK--------VFRH-FLFGF--
PPYW-------------EEYA----------------------------------------------------
-------AECLDGES--VP-DVNKE-----------KMDEGL-------------------------------
-------------------------------------------------------------------------
----------------------------EDPSPVSTPS------------KSVE-------------------
------ENE----------------EGNCVI------------------------------------------
--------------------------------------------------------------DVEI-------
-------------------------------------------------------------------------
------------NLCENMSQR------------------------TTIDAPK---------------------
-------------------------------------------------------------------------
```


--------------------------FSL--
--------LSENDY-----------HPSSTI---------------NPVINK-----------------
DDNAKN----------EVRKN--D---
---GSKDTSED--
--TPQS---------

---LGCEINDV--
RPLELEIQ----------------------------------LPEEP-------LFCYVAQS-----------
----
--SE-------------------------------

--RM-----
GDPSSSGKC---QE-------------

WILXXXXXX--
----------------XXXXX--

------------SVTTQNNASGESPIS--
--------------WSNKDVL--
------------------PV---------VVTNTEG-------------------------------------
----
---VRS-R--IHSVS--------------
-------------VTQD------------------------------KGQK-SG----------------
NITQTGMQNDY-------SNRLA--
RSPSGCDDVNHA------YGL-------------------------------------GTILEEG--------
---------------------QSCTKEDS--
-----------SFGERT--

--------KRR--

--INF---NVHVS------------
--FS-----------------

>Raphanus_raphanistrum_RrC12816_p1

----MAD--NPNP--------
-------------------------------------DD---------------------------------
D---CSCY---

----------------QKT--
--VVLRDWWLIK-CPIE--------FQG-
KRFGVA---------------GTQI---AD---
--------------------------------------TG-AV-RVF--------------------------

----
SSCPIIRAFDVFTLEASDGVCIILRGFL-------NRQRLLQS-GFLPQ--------ISRE-FIFGF--
PPRW-------------ESKC--
-------NLCFVGL-PSQS-PIN--------------------------------K-----------------
----
----------------------------QASRSIISPC------------NNAK-------------------
------SNL----------------EDK---
--RRDKITVVEK--ITEEEI-------
------------NDKLGRC----------------------------ST------------------------
------------SKDDNKPSK--

--------TNPGG------------EGGLSM---------------AAAAK----------------------
---DEC---G--EVFNN--QVISP---AG--
KKHTSAD----------------------------------EVTSIMRATE----------------------
-----------------------------------ESQGK----GE----LEGTNACPHSL------------
---------------SGSDKPGK-------------KAAS-K-KSGRILRSHGNVVEHANH------------
------------SGTKVR-------------------RKLLD------------------AVSGVQKNPTVN-
------EGLNK---EAKGNGVER---------------DD-------------------------------
EEV------------------ARVDGCGKMHPSGTN-------------------------------------
---------------S-KKIASK---KES-M-----TS-----------------------------------
----------------------EQRKGKLKVTKTSLSGST---------------------------------
----
NKHGKNGNS---KKS------------
-------------------------EQ--
--ILKGGDV----------------
---DVVVSVEPM-

--------TQSR---

----SKVKDNLSLGKTIR---
-----KIDFDEE---
--------------------VTPEKDA--
---KKH-K-
---TNNA----------------------
---VVSPDSLGQ--------------------------RRSR-SG----------------RVLVSPLEF-W-
------RNQVPVYDV---DRNLIQVNE----
-------GY-QT-N--------------------------------STPPSKGFL-
KFFSFYLWKNIPQGAATTCYVALHPSLKGVTGKYFADCNEVIPT---------KLAR----------------
---------------DDTLAQK---------------------------------------LWDFSV------
----
-------------------------KLINS---

------------VSKK---

---NY----------
---LGFEDTS---------

>Raphanus_raphanistrum_RrC35_p2


```
----MATK--------------------SKLKSVPARR----------------------PSPRT--------
----------------RS------------GAVPEPIST----------------------------------
-P--------------------------------ATR-----------------------------SRS----
-------------------------------------------------------------------------
-----------------------------------------------------------VP------------
--------------------------------------------KPNS---------------DSIPR-----
---------------TTPFSS--------------------------KFI-----------TPISG-------
----------GGAL-LKP-------------------------------------------------------
---------------------------------------VSLSDWWLTK-KA----------NNN-KGLGVS-
--------------GFE-------------------------------S------------KG----------
-------------------------------GS-KV-RLF---------------------------------
--------------------------------------SSATISTRHDSTTLQTSDGLTVSISGFI-------
NRSRSLQN-GFSSQ--------VCNR-FLLGF--PYHW-------------KDYT------------------
-----------------------------------------EEGFV---------------------------
-------------------------------------------------------------------------
-------------------------------------------------------------------------
-----------EEDKN------------------------G--------------------------------
-------------------------------------------------------------------------
------------------YGVVSFDDI----------------------------------------------
---------------------------------------------------PVNRL-----------------
------QDVLFTA------------------------------------------------------------
-------------------------------------------------------------------------
-------------------------------------------------------------------------
-------------------------------------------------------------------------
-------------------------------------------------------------SSCFQ-------
-------AKILD----------------------------------DAV------------------------
----------------------------------------D-------------------------------
SLRDLL--RSS--------------------------------------------------------------
-------------------------------------------------------------------------
--TE-KPDKECRTP------------------------------------RTDDG------------------
-------------------------------------------------------------------------
GE-------------------------------E-------------------------------SLVLS---
-------------------------------------------------VKGVETRGMLRRR-EEG-E-A---
---------------------------------------------------------SIGERLH---------
-------------------------------------------------------------------------
-----------------------------RSSKK---------------------------------------
----------KRD------------------------------------------------------------
-------------------------------------------------------------------------
-------------------------------------------------------------------------
-------------------------------------------------------------------------
-------------------------------------------------------------------------
-------------------------------------------------------------------------
-------------------------------------------------------------------------
-------------------------------------------------------------------------
-------------------------------------------------------------------------
-------------------------------------------------------------------------
-------------------------------------------------------------------------
-------------------------------------------------------------------------
-------------------------------------------------------------------------
-------------------------------------------------------------------------
-------------------------------------------------------------------------
-------------------------------------------------------------------------
-------------------------------------------------------------------------
-------------------------------------------------------------------------
-------------------------------------------------------------------------
-------------------------------------------------------------------------
-------------------------------------------------------------------------
-------------------------------------------------------------------------
-------------------------------------------------------------------------
---------------------Q---------------------------------------------------
```


>Raphanus_sativus_018437333.1

----MATK--------------------SKLKSVSAHR-----------------------SSPRT--------
----------------RS------------GAVSEPIST-----------------------------------
-P--------------------------------ATR------------------------------SRS----
----
--VP------------
---KPNS---------------DSIPR-----
---------------TTPFSS---------------------------KSI-----------TPISG-------
----------GGAL-LKP--
--VSLSDWWLTK-KA----------NNN-KGLGVS-
--------------GFE--------------------------------S------------KG----------
-------------------------------GS-KV-RLF----------------------------------
---------------------------------------SSATISTRHDSTTLETSDGLTVSISGFI-------
NRSRSLQN-GFSPQ--------LCNR-FLLGF--PYHW--------------KDYT------------------
--EEGFV---------------------------

-----------EEDKN-------------------------D--------------------------------
----
------------------YGVVSFDDI---
--PVNRL-----------------
------QDVLFTA---

--SSCFQ-------
-------AKILD-----------------------------------DSV------------------------
---D-------------------------------
SLRDLL--RSS---

--TE-K--KECRTP-------------------------------------RMDVG------------------
----
DG-------------------------------E--------------------------------SLVVS---
--VKGVETRGMLRRR-EEG-E-A---
--SIGERLH---------

-----------------------------RSSKK--
----------KRD---


```
-------------------------------------------------------------------------
-------------------------------------------------------------------------
-------------------------------------------------------------------------
-------------------------------------------------------------------------
-------------------------------------------------------------------------
-------------------------------------------------------------------------
-------------------------------------------------------------------------
-------------------------------------------------------------------------
-------------------------------------------------------------------------
---------------------Q---------------------------------------------------
-------------------------------------------------------------------------
-------------------------------------------------------------------------
-------------------------------------------------------------------------
-------------------------------------------------------------------------
-------------------------------------------------------------------------
----------------------------------------
>Raphanus_sativus_018452984.1
-------------------------------------------------------------------------
-------------------------------------------------------------------------
-------------------------------------------------------------------------
----MAD------------------------------------------------------NPNP--------
-------------------------------------DD---------------------------------
D-----------------------------------------------------------------ISYH---
-------------------------------------------------------------------------
-------------------------------------------------------------------------
-------------------------------------------------------------------------
-------------------------------------------------------------------------
----------------QKT------------------------------------------------------
----------------------------------------VILRDWWLIK-CSIE--------FQG-
KRFGVA---------------GTQI-------------------------------------------AE---
--------------------------------------TG-AV-RVF--------------------------
---------------------------------------------
TSCPIIRAFDVFTLEASDGVCIILRGFL-------NKQRLLQS-GFLPQ--------ISRE-FIFGF--
PPCW-------------ESKC----------------------------------------------------
-------NHCFVGV-PSGS-PIN--------------------------------K-----------------
-------------------------------------------------------------------------
----------------------------EASRTILSPC------------NNAK-------------------
------SNL----------------EDSSIP------------------------------------------
-------SVVEK---K------N--------------------------------------NTVET-------
------------NDTVG----------------------------GGSS------------------------
-------------------RK------------------------MSLRLHSKS-------------------
-------------------------------------------------------------------------
-------------------------------------------------------------LE----------
-------------------------------------------------------------------------
---------LMK---------------------------------------------------KNT-------
--------SNDGG-----------------------------------------------------------
VSK-DVC------EVINI--EV----DGCG-------------------------------------------
-ENHTGAD-N-----------------------------AVDKVTSASATEE---------------------
---------------------------------SL----------------LTTTT-----------------
-------------------------------------------------------------------------
-------------------------------------------------------------------------
-------------------------------------------------------------------------
-------------------------------------------------------------------------
-----------------------------------TS------------------------------------
---------------------EQRNGKLKVTKTSPS-------------------------------------
----------------------------------------------------------------
GGSSKRGKS-------------------------------------------------KKS------------
-------------------------EK----------------------------------------------
--------------------------------------------------ILIG-DSET--------------
-------------------------------------------------------------------------
```

-------MTRSR---

----SKVKDNLSVGNTIR---
-----KIDFDEE---
--------------------VTPEKDA--
---KKH-K-
---TNSA----------------------
---VVSPDSLGQ--------------------------KRSR-SG----------------RVLVSPLEF-W-
------RNEVPVYDL---DRNLIQVNE----
--GTPPSRG--------------------
----------KGS--
-VSRK--

---PRR-

>Schrenkiella_parvula_Tp2g05410

---MAATK--------------------STFQSLSACR----------------------SSPRT--------
----------------RS------------GALPQPNSSLRTRSGALPQPNSSLRTRSG------
AVPHPNSSTP--------------------------------RTC----------------------------
-SKS---
---VP----
--ELNVS-----------
PETEEIPP---------------------TPLSF--------------------------KAI----------
-TPV--------------------VGT-LKS--
--VSLSDWWLTK-KT--------
---KE-NALGVT---------------GFE-------------------------------S-----------
-KS---GS-EV-RLF--------------------

SSGAISTRHDSTTLETSDGITVCVSGFI-------NRSRTLQN-GFSSE--------VCNR-FLLGF--
PYHW-------------RDYN--
-------EEGFV---

--EEEKK------------------
------H--
---FT-VLFDDI-------

-----------------PVNRL-----------------------QDVLFTS---------------------
----

---------------------------SPCLK--------------SKILD----------------------
------------DVV--
------D-------------------------------SLRDLVCPRTVK-----------------------
----

-----------------------------------SDKKCE-KSDKRCEKS---------------------
--------------RTVDE---

-----------------------------SLVPS--------------------------------------
-------------VVGVKTRGMLRRR-EEN-E-T--------------------------------------
---------------------TIGERVHLT--
--SKKKRSREN-
---NKG----------------------
--------------TKRCCREQRAMRIHGGGQATSVQSNPNGSNTIFRKV--------EYT--
LKIIDEDGDTFAKRAEMYYRKRPEIVNFVEEAFRSYRALAERYDHLSR-ELQSANRTIA-------------
-TAFPEHVQ----------------------FPLE---DDDD-
ENEDHEGNPRKPPKHLHLIPKGSNIPEVPEIP-KKEFRSQSMMLSRKGPAGLK-------RTVSSA----
LAKREAAIVSSGLSKEEGLEEIDKLQKGILALQTEKEFVRSSYEQSYERYWDLENEVTEMQKRVCSLQDEFGL
GAAIDDSKARTLMASTALSSCKDTLAKLEEKQKQSV--EEAEIEKERITTAK----ERFDA---------
LRNKFENPESDGHDEVTKTEEKEKE--------
ADVVLESSYESEREDSNENLTVVKLAEKIDDLVQRVVSLETNASSHTALVKT------LR--
SETDELHEHIHGLEEDKASLVSDSTVMKER------ISVLEDELSKVRKLFQ----
KVEDQNKSLQNQFKEANWTAENLSGKLQDVKMDEDVEGAGIFQELPVVSGSEDSRDDLNSISKKTET-
SSVKERKNDAIVMKESEDTEGAQEEKSETKDSFALSETASTCFGTEGDELVTEDEDEETPNWRQLLPDGMEDR
EKVLLDEYTSVLRDYREVKRKLGEVEKKNREGFFELALQLRELKNAAAYKDVEIQSLRQKLDTPGKDSLHQVE
GNNQ--------------LEHDQGQRE-SV--
SISPTSNFSVSTTPHHQVGEIKRTSGRTKSNEVRVKFADVDDSPRTKIPTVEDKVRA-
DIDAVLEENLEFWLRFSTSVHQIQKYQTTVQDLKSELSKLRIESKQQQESPR-SS-SNHAAASEAKPIYRHL-
-REIRTELQL-WL------ENSAVLKDE-----
LQGRYASLANIQEEIARVTAQSGGSKVSDSEITGYQAAKFHGEILNMKQENKRVSSELQSGL-DRVRALK---
---TDVERILSKLEEDLGISSATEARTTP-SKSS----------------------------SSGRPRI---
-----------------------------------PLRSFLFG----VKLKKHKQQKQSSSS----------
----

--------------------------------LFSCV-----------------------------------
----
---------------------------SPS--
---PALLKQS--------------------

---------------------SYIRQP--
GKL--P-----------E
>Schrenkiella_parvula_Tp6g39990

----MTDH---P--------
------------------------------------DG---------------------------------
D-----------------------------------S-----------------------------SSF---
----

----------------QRT---
---------------------------------------VILRDWWLIK-CSKV--------FEG-
KRFGVA---------------GTEIA---------------------------AS------------VE---
-------------------------------------TR-AV-RVF--------------------------
----
TSSPIIKAFDVFTLQASDGICVILRGFL------NKERVVKS-GFTPE-------ISRE-FIFGF--
PPCW-------------EQVC---
-------NNCFGGV-PFGT-GINAI---------------------------------PSVID---------
----

----------------------------KASRTILSPC------------KNTQ-------------------
------GNL----------------ENTPAE--
--NRDKSTVTEK----------S---------------------------------------TADI-------
------------NDKDGHS--------------------------GRSR------------------------
------------ARERKTASK------------------------MSLRLQSISG------------------
-GKGAEHE--RKLKVTKIQNATD--------
---D---GSEALNKAK--SGDVE----------

--KAMSNEGN-----ES----EVQNR-------
--------IDDGY------------YGSEGL---------------DK-GK------------------
SSDVEK-DEC------EVTNN--ELISPADGCS--
----KKHTDA-----------------------------------YVTERSATGK------------------
------------------------------------SLTSEQGR----GE----LKITRASPHS---------
-------LFEDL-------DKSSKPGK-------------KGTS-K-KSGKTLKCKGNVEDSVNH--------
----------------SGTKVNSAKN--------------KRKL--------------------NVSQVQ-
NPTTNDGDH-GSEVLNK----ATGNDVEK---------------DE----------CIAINN-----------
----EVI------------------SPVDECGK-RNSVTY---------------------------------
-------------------S-KELTSKNATKES-L-----AS-------------------------------
--------------------------EQRKGKLKVTKTFLLS-------------------------------
--MSKDLNHS-----
TKPGRKGNS---KKS------------
-------------------------EK--
--TLMG-DL----------------

NVVEPMN--
--------------YSGSK--

----------VKDAEENLSGEKTNR--
------------KIDFDEE--
---------------------------VTPDKDA---------------------------------------
----
-KKQ-K--INA----------------
-----------VSPDSLGQ--------------------------RRSR-SG----------------
RVLVSSLEY-W-------RNQIPVYDV---
DRNLIQVNE-----------GH-ET-NST----------------------------PYSTP-SKG-------
-----------------------NGS---
--------------NSRK---

---------PRR---

>Sisymbrium_irio_maker2387.1

--MKMATK--------------------SKPHPLSARR----------------------SSPRT--------
----------------RS------------GAVPEPISS----------------------------------
-P--------------------------------RTC-----------------------------SRF----
----
---VP------------

--ELNST--------------DDIPS-----
---------------RTPFSS--------------------------KAI-----------TPI---------
-----------VGT-QKS---
---------------------------------------VSLSDWWLTK-KA-----------NE-KGLRVT-
--------------GFE-------------------------------S------------KG----------
-------------------------------GS-EV-RQF---------------------------------
--------------------------------------SSGPISIRHDSTTLETCDGITVCVSGLI-------
NRTRTLQN-GFASE--------VCNR-FLLGF--PYQW-------------KDCS------------------
---EEGFVE--------------------------

-----------EEEKK------------------------R--------------------------------
----
------------------FT-VSFDDI--
---PVNWL-----------------
------QDVLFTS--

---SSCFK-------
-------GDILH----------------------------------HVV------------------------
--D-------------------------------
SLRGFVCPMSEN---

SDKECD-KSDKECEKSDKNSEKSDKEC----------------------EKSRMDND----------------
----
--DE-------------------------------EE-----------------------------KSVVPS-
---VVGVKTRGM-RRR-EEN-ETA-
---TIGKRVHTL-----

----------------------------SKKRSREK-------------------------------------
------------SKDME--
------------EKV--------EYT--
LKIIDEDGDTFAKRAEMYYRKRPEIVNFVEEAFRSYRALAERYDHLSR-ELQSANRTIA--------------
-TAFPEHVQ-----------------------FPLE---DDDD-ANEDHEGNPRKP--
HLHLIPKGSNIPEVPEIP-KKEFRSQSMMLSRKGPAGLK--------RTVSSA----
LAKREAAIVSSGLSKEEGLEEIDNLQKGILALQTEKEFVRSSYEQSYERYWDLENEVTEMQKRVCNLQDEFGL
GAPIDDSDARTLMASTALSSCKDTLVKLEEKQKQSV--EEAEIEKERITTAK----QRFDA---------
LRNKFEKTESGDHDEFIKTKERQEENV-------
TDVVQGSSYESERDDSNENLTVVKLAEKIDDLVQRVVSLETNASSHTALVKT-------LR--
SETDDLHEHIRGLEEDKASLVSDSTDMKQR-------ITVLEDELSKVRKLFQ----
KVEDQNKSLQKQFKEANWTAEDLSGKLKDVKMDEDVEGAGIFQELPVVS-
SEDSRDDLKSISKETGTRSSVEGRKKDANAVKEIEDIEGAQEEKPEIKDSFALSETASTCFGTEGEELVTEDE
DEETPNWRQLLPDGMEDREKVLLDEYTSVLRDYREVKRKLGDVEKKNREGFFELALQLRELKNAVAYKDVEIQ
SLRQKLDTPGKDSPHQVEGNNQ--------------LEHDQGQRE-SV--
SISPTSNFSVSTTPHHQVGEMKRTPGRTKSNEVRVKFADVDDSPRTKIPTVEDKVRA-
DIDAVLEENLEFWLRFSTSVHQIQKYQTTVQDLKSELSKLRIESKQQQESPR-SS-SNHAAASEAKPIYRHL-
-REIRTELQL-WL------ENSAVLKDE-----
LQGRYASLANIQEEIARVTAQSGGSKVSDSEISGYQAAKFHGEILNMKQENKRVSSELQSGL-DRVRTLK---
---TDVERILSKLEEDLGISSATEARTTP-SKSS-----------------------------SSGRPRI---
------------------------------------PLRSFLFG----VKLKKHRQQKQSASS----------
----

--------------------------------LFSCV------------------------------------
----
---------------------------SPS---
--PALQKQS--------------------

---------------------SYVR-P---
GKL---P-----------E
>Sisymbrium_irio_maker4372.1
MSTGVCGKRVWYDDFFASSSSSPTNKRSKCSTFGSPIRSSELGSGSDDPVASLIHMFPTMDPEFVRNVLSNKN
NVFEEAEESLRSISFNGDLDRAEAAPFDGSAVDWRDEDMMDGAKWVDILVSEMAKAINIDDMRLRVKGILEAL
ERIIENNSNASKKLEYASLKENLQRLINDNQILKRVIANQHQRSSESEEKAKEVEHLKGVVGQYQEQIHKLEK
EVFSMAD--TP--------
-------------------------------------DD---------------------------------
D------------------------------------S-----------------------------SSF---
----

----------------QRT--
--VILRDWWLIK-CSKE--------FEG-
KRFGVA---------------GTE---

-----------IAVD-GFIPE--------ISRE-FIFGF--PPGW-------------DQIC-----------
--NNCFQGV-PFAT-DTNSV-------
---------------------------PLVMMD--

KASRLVLSPC------------KNAK-------------------------VNI----------------
VDSSAE--NGDKSTVTEK----------K--
------------------------------------NMAEI-------------------NDKDGRS------
--------------------GGSR------------------------------------AIGRKTASK----
--------------------KSLRLQSKSG-------------------GKSTEDG-----------------
---------------------------KKLELSKVQNTTN-----------DGNHSSQGLNEVK---------
---------------------------------SRDVEKN----ECEAM------------------------
----
--------------------GYEGSERKLDES----KVQNG---------------TSDGD------------
LGGEVL---------------DM-AK------------------NSHVEI-DEC------EVVNN--
KVISPADGCG--KKHIGAD-N----------
--------------------VDTVTSMMDTGE---
-------------SLTSEQGK----DE----LKVTRASPHS----------------LFEDL-------
DKSNSPGK-------------KGIS-K-KSGKTRRSDGNVVERVNR------------------------
SRTKVKSAKD--------------KRKL--------------------DVSEVQ-NRTTNEGGL-GSEGLNK-
---T-------------------------------------INY---------------EVI-----------
-------SPVHGCGK-SHSGTD---
-S-RKLTSKNATKES-L-----TS---
--------EQRKGKLKVTKTSLRS---
--------------------------------------KSKDVSNS-----SKPGKKGES-------------
------------------------------------KKS----------------------------------
---GK--
----------------------------TLKG-DR--------------------------------------
--DVVEPMN----------------------
--HSVSK----------

--VEEAE---TGGKTNR----
--KIDFDEE----------

VTPNKVA--
---KKQ-K---------------------
---------------------------TNA---------------------------MSSKSVGQ--------
------------------SRSR-SG----------------RLLVSPLEY-W-------RNQIPVYDM-----
--NRNLVQVNE-----------GH-ET-N------
--------------------------TTP-TKG------------------------------KGL-------

--DSQK---------------

---RRR---------------------

>Tarenaya_hassleriana_010524136.1

---MMGIP--------------------ARWKTTMAKKRK------------SS------SRPAP--------
----------------RS---
-PC---SRY----

---QTRP------------
--DPISS-----------PAHDTIAR-----
----------------TPFSA--------------------------GAS-----------TAV---------
------------QS-LNS---
---------------------------------------VFLSDWWLRR-VQ-G------------KGLSVA-
--------------GFE-------------------------------A------------KG----------
-------------------------------GS-GV-RLF---------------------------------
--------------------------------------SSGAISKRHDSTTLETVEGITVSISGLI-------
NRLRTLQN-GFSFE--------VCSH-FLMGF--PWYW-------------EHYA------------------
------------TLSCG-----------------------QEE------------------------------
----

-----------TGEKH------------------------DQT----------------NE------------
------------------------------------GRPASLKGES----------AS---------------
------------------CSTVSFDDI--
---PVNRF-----------------
------YELLMSPPNH---

--SKDCLG-------
-------NKALD----------------------------------EVL------------------------
--G-------------------------------
RLRHCISQG--
---------------SP--
--VKEQSDKRFETSAD----------------------------------SIN--------------------
---------------------------------SE------------------------------------
AGEE-------------------------------EEGAD-------------------------HDKKMAT-
---GMGVKTRGMMRLR-RTIKE-
EG-----------------------------------DLPF------------------GCAPGKRFQNA---
----
------------------RYMA-------SVEARNASR-----------------------------------
--------------NSS--
---------------------------------VEKNGQSLSSKA----------------------------
----

--------------------------------------SSAYD------------------------------
----

------------DYSEL--
-------------------------------TI--

>Tarenaya_hassleriana_010543322.1
--MVH------------------

----ILEM--------------------K--
-------------------------------------GD---------------------------------
D--AT------------------------------CNS-----------------------------SCF---
----

----------------QKT--
--VMLRDWWLIK-CPDE--------FEG-
KRFGVA---------------GTELM----------------------------AS------------TE---
--------------------------------------RR-AM-RVF--------------------------
----
TSAPIVKAVDVWTLETSDGICILLKGLI-------NKERSANG-GFSPE--------IFSH-FIFGF--
PPRW-------------EEIS--
-------NKCFKGESSSGA-GTDSV----------------------------------TLRIH---------
----
----------------------------TADRLMSTPC------------KNKE-------------------
------SPLESKIKIG-------IGENNTAK--
--ANDEDSTDDK----------SGSPEL------------KMAV----P----------REKSGDMI------
-----------RNTRTGNTAPP----------------------VTSEE------------------------
------------VKVEQNTVR------------------------KSSRLQLKSS------------------
-ENDTENG--RKSDTAKVSNPSY--------
---CGEH-SNSLPTVP---

-------TPIMA---
--------TSNGN-----------------F---------------DE-AK------------------
TVDAEN-DDR-------------------DG--
------------S------------------------------VDDVVSSEVA--------------------
----
---------------------TTVPGE-------------SSAS-----------------------------
---Q---

-------------------------VDGFVK-RHTDPA-----------------------------------
-----------------SKEKKLRKCSTEES-E-----SS---------------------------------
------------------------GQCRVKGKETRASPCL---------------------------------
--QSKENGRT-----
AEFAKRENP---NKA------------
-------------------------TK--
--SVKS-DG----------------

KVRALN---
-------------HSKRK---

----------GESKEKCVQDEDWR---
-----------RINFDVE---
--------------------------ATPGKQA--

RKE-K--TAF-----------------
----------VSPELSST--------------------------KRSR-SG----------------
RLLVSTLEF-W-------RNQVPVYDA---
GRNIIGIKE-----------RI-DD----------------------------------TSPVSRG-------
-----------------------KGS---
--------------ESGR---

---------RRR---

>Thlaspi_arvense_06963

----MAEA---NL--------
-------------------------------------DG---------------------------------
D------------------------------------S-----------------------------SAF---
----

----------------QKT--
--VILRDWWLIK-CSKE--------FEG-
KRFGVA---------------GTEIAA--------------------------S--------------VE---
--------------------------------------TR-AM-RVF--------------------------
----
TSSPIIKAFDVFTLQASDGICITLRGFL-------NKERVVKS-GFIPE--------ISRE-FIFGF--
PPSW-------------EQIC--
-------NNCFGGV----T-DVNTV----------------------------------PTVND---------
----
----------------------------KACYPFLSPC------------KNSR-------------------
------GRL----------------EDSPAE--
--SRAKSPVTEK----------N--I-------
------------NEKDTRS--------------------------GCSV------------------------
------------ARDIKNPP----------------------------RLQSKRG------------------

-GKKAEDE--RELGLSKLQSTAS--------
---DGDNGNEGLKKAK--SGDVEKD----EC--

--EAICNEGDEMKLDES----KVQNR-------
--------TNGGD------------HGCEGL---------------DE-AK------------------
SSDVEI-DEC------EVINN--EVISPADGCG--
----KKHTGAD-I------------------------------VDRVTSMSAAGE------------------
------------------------------------SLTSKQGK----GE----PKVTRASPHS---------
-------LFEDL-------DKSSKRGK-------------KGIS-K-KSGKTLKGDGNVVEHVDH--------
----------------SGTKVKRAKN--------------KKKL--------------------DASEVQ-
HPTANDEDH-GTEGLNK----AKGNDAEK---------------DE----------CIAIDN-----------
----------------------------DACGK-RHSGTD---------------------------------
-------------------S-EKLISKNATKES-L-----TS-------------------------------
--------------------------EQRKGKF--
--NS-----
SKPGKIGKS---KKR------------
-------------------------EK--
--TFKG-GL----------------

NVVEPMD--
--------------HSGSE--

----------VKEAEANLSREKTNR--
------------KIDFDEE--
---------------------------VTPDREA---------------------------------------
----
-K---K--TNA----------------
-----------VSADSLGQ--------------------------KRSR-SG----------------
RVLVSSLEY-W-------RNQIPVYDM---
DRNLIQVHE-----------GR-GT-Y--------------------------------STP-SQG-------
-----------------------KGS---
--------------DPRK---

---------PRR---

>Thlaspi_arvense_17942

----MATK--------------------SKPLFLSARRSSPPV-------------PELISSPRT--------
----------------RS------------GAVPELISS----------------------------------
-P--------------------------------RTF-----------------------------SKF----
----
---VP------------
--EPNSS-----------PRTDGIPR-----
----------------TPFSS--------------------------GAV-----------TPV---------
-----------VGA-LKS---
---------------------------------------VSLSDWWLTK-KA-----------NE-KALGVT-
--------------GFE-------------------------------S------------KG----------

-------------------------------GY-EV-RLF---------------------------------
--------------------------------------SSGTISIRHDSTTLETSDGIRVCISGFI-------
NRSRTLQN-GFSSE--------VCNR-FLLGF--PYSW-------------KDHD------------------
---ED--ET--------------------------

-----------VEEKK------------------------H--------------------------------
----
------------------FG-ISYEDL--

---------LFTS--

---SSCLK-------
-------SEILD----------------------------------DVV------------------------
--N-------------------------------
SLRDLVCPR--

--TE-KSNEECEKS------------------------------------RMITE------------------
----
DDDD-------------------------------E-------------------------------SVVPS-
---VVGVKTRGMLRRS-EEN-E-A-
---SIGKRMLQR-----

-----------------AASNAYSWWWASHIRTKQ--------------------------------------
------------SKW-------------------------------------LEHNLQDME------------
------------EKV--------EYT--
LKIIDEDGDTFAKRAEMYYRKRPEIVNFVEEAFRSYRALAERYDHLSR-ELQSANRTIA--------------
-TAFPEHVQ-----------------------FPLE---DDDN-ENVDFEGNAPK---
HLHLIPKGSNIPEVPEIP-KKEFRSQSMMLSRKGPAGLK--------RTVSSA----
LAKREAAIVSSGLSKEEGLEEIDKLQKGILALQTEKEFVRSSYEQSYERYWDLENEVTEMQKRVCSLQDEFGL
GAAIDDSEARTLMASTALSSCKDTLAKLEEKQKQSV--EDAEIEKERIITAK----ERFDA---------
LRNKFEKLESDDHDEVFKTEEEEE----------
ADVVQESSYESEREDSNESLTVVKLAEKIDDLVQRVVSLETNASSHTALVKT-------LR--
SETDDLHEHIRGLEEDKASLVSDSTDMKQR-------ITVLEDELSQVRKLFQ----
KVEDQNKNLQKQFKEANTTADDLSGKLQDVKMDEDVEGAGIFQELPVISGSE---------
TKEAETRSSVEERKKDDTVVKESEEIEGGEEEKPEIKDSFALSETASTCFGTEGEEMVTEDEDDETPNWRQLL
PDGMEDREKVLLDEYTSVLRDYREVKRKLGEVEKKNREGFFELAIQLRELKNAVAYKDVEIQTLRQKLDTHEK
ESPNQVEGSNQ--------------LDHDQGQRE-SV--
SISPSSNFSVSTTPHHQVGDMKRTPGRIKPNEVRVKFADVDDSPRTKIPTVEDKVRA-
DIDSVLEENLEFWLRFSTSVHQIQKYQTTVQDLKSELSKLRIESKQRLDSPRSSS-SNTAVASEAKPIYRHL-
-REIRTELQL-WL------ENSAVLKDE-----
LQGRYASLANIQEEIARVTSQSGGSKVSDSEISGYQAAKFHGEILNMKQENKRVSSELQSGL-DRVRALK---
---TEAERILSKLEEDLGISSATEARTTP-SKSS-----------------------------SSGRPRI---
------------------------------------PLRSFLFG----VKLKKHSKQKQSASS----------
----

--------------------------------LFSCV------------------------------------
----
---------------------------SPS---
--PALQKQS--------------------

---------------------SYVKQP---
GRL---P-----------E
