# Supplementary material for: Recurrent Plant-Specific Duplications of KNL2 and its Conserved Function as a Kinetochore Assembly Factor
Source: Mol Biol Evol. 2022 Jun 7;39(6):msac123. doi: 10.1093/molbev/msac123 (PMC9210943; doi:10.1093/molbev/msac123)
Supplement: msac123_Supplementary_Data [file msac123_supplementary_data.zip › Supplementary_File_5_gamma_KNL2_alignment.pdf]

γKNL2

SANTA domain   conserved hydrophobic motifs   CENPC-k   imperfect_duplication

```
Sorghum_bicolor_XP_021304566          --------MKPLPVPEAGSPHRRGM----PP-SLLSPS-SRSAVP-------------AA
Setaria_italica_XP_022683723          ---------MQPPPRTPVAPRASTPRRGMPP-SLLSPSYSRRAFP------ATAASAAAA
Oryza_brachyantha_XP_040378886        -----MATKTPNPPPPPASPRRSAPP---PV-TMLSPSFSRNPLR---------SSAGPV
Oryza_sativa_XP_015634019             MGTEPSQTPTPPPPPPPPASPRRRAPQ---PA-TILSPSFSRNPLRATAA--SASASAGPV
Brachypodium_distachyon_XP_003577118  ---------MASPPE--------------PAIPVLSPLSSR-------------LSAAAD
Aegilops_tauschii_XP_020160360        -----MAKKTPSPPPRARSRRGAAPTSPTPA-AALSPPFSPAPLRTRLG--AAAVAAAAA
Triticum_dicoccoides_XP_037485311     -----MAKNTPSPPPRARSRRGAAPTSPTPA-AALSPPFSLAPLRTRLGAAAAAAAAAAA
                                                  *                *  . ***  *                  .

Sorghum_bicolor_XP_021304566          ADGDHDAAVSEHACVTLSEWWLATAEGDDQKIAVAGTFERNQTVQEYSPAPIAKRHTSSV
Setaria_italica_XP_022683723          ASAASDLDAAERPCVTMFEWWLTRVEGDDRKIAVGGLFERNQTVQEFAPATITKRYETCV
Oryza_brachyantha_XP_040378886        PFHCD----EERPCVELFNWWLERVEGDDRKVRVAGHAERGRKLHLFTSAPIVKRHEACF
Oryza_sativa_XP_015634019             PSPSSSSDCEEHPCVELFDWWLKRVEGDDRKVRIAGHTERNHKPHLFTSAPIVKRHKACM
Brachypodium_distachyon_XP_003577118  SSSVSSSAPVEHPCFTLQNWWLVRVEGEERKIAVSGFTQRGDA---FTSAPIAKRHESLV
Aegilops_tauschii_XP_020160360        AAAAASSSPVEHPCVTLCEWWPVRVEGEERKLAVSGFTERNDA---FTSAPIAHRYEPLT
Triticum_dicoccoides_XP_037485311     AAAAASSSPVEHPCVTLWEWWPVRVEGEERKLAVSGFTERNDA---FTSAPIAHRYEPLT
                                      .         *..*. : :**   .**::.*: :.*  :*.     ::.*.*.:*: .

Sorghum_bicolor_XP_021304566          LETEEGTVLRLHGLHNVLRTYHNGYSAKVYSEFLNGFPDWWQSCKPCNPKLMNSHTECCS
Setaria_italica_XP_022683723          VETEDGIVLLIRGSLNVSRTIANGYSSKVCENFMIGFPCWWQSC--------NL-LYPKE
Oryza_brachyantha_XP_040378886        LEAEDSVIVLINGPLDLSQMQKHGYSLEVCEKFMVGFPYFWERY--------NLGSQASS
Oryza_sativa_XP_015634019             LEAEDSIIVLIDGPLDLSQMENNGYSLEVCEKFMTGFPCLWESY--------NLGSQQSC
Brachypodium_distachyon_XP_003577118  LEDEDGVVVRIDGLMSLCRMRRNGFSLQICESFLIGFPSWWESW--------DSHFESQP
Aegilops_tauschii_XP_020160360        LQDEGGVVVLLHGSINLLRMRENGFSVQICEQFMIGFPFWWETW--------DSHMESYP
Triticum_dicoccoides_XP_037485311     LQDEGGVVVLLHGSISLLRMRQNGFSVQICEQFMIGFPSWWETW--------DSHMESYP
                                      :: * . :: : *  .: .   :*:* :: ..*: ***  *:          :

Sorghum_bicolor_XP_021304566          SNASNSGVDSTQFYLERYMQGRRLDSYGTYLISKFPDILASFLHNDA-VFQKS-------
Setaria_italica_XP_022683723          TGTGNSEVDSTRFYLEKFQLGERFHSHGTSLLSELLNSVRSYSRNDA-AFQKSSHLPNGA
Oryza_brachyantha_XP_040378886        CKTSKLQDSSTKFYLEKFQLGNFIDKVGYSFIASLLNNGTHFSGDAG-SFENV-------
Oryza_sativa_XP_015634019             SYTSISRDGRTKFYLERFQIGNFIDKVGSSFLANLLNNSRSSSGNDADSFEKG-------
Brachypodium_distachyon_XP_003577118  TSSSNSQEDSSQIYLKIFQLGNVVQKSVASFIKNPLHDAKIFRRYVADAFTQC-------
Aegilops_tauschii_XP_020160360        NCFIDPREGSAQFYLEKFQLGNFIQKFGPSFIEDLLNNAKNFPIDHLDAFTES-------
Triticum_dicoccoides_XP_037485311     NCFIDPREGSAQFYLEKFQLGNFIQKFGPSFIEDLLNNAKNFPIDHLDAFTES-------
                                              . :.:**: :  *  . .    :: .
```

```
Sorghum_bicolor_XP_021304566          ------------------------------------------------------------
Setaria_italica_XP_022683723          PRFEEYTGDGDIAINENAAASNDDRERHEAACNEVYNVDMHMTACRALRERDGGYIDTHA
Oryza_brachyantha_XP_040378886        ------------------------------------------------------------
Oryza_sativa_XP_015634019             ------------------------------------------------------------
Brachypodium_distachyon_XP_003577118  ------------------------------------------------------------
Aegilops_tauschii_XP_020160360        ------------------------------------------------------------
Triticum_dicoccoides_XP_037485311     ------------------------------------------------------------

Sorghum_bicolor_XP_021304566          ----------------------------------------------------------SH
Setaria_italica_XP_022683723          SLVLTVECPNDAANKEADSATPTSTCDQRKAQHVALSKKAASKQNEDVPTSVCLDVQNSY
Oryza_brachyantha_XP_040378886        ----------------------------------------------------------FY
Oryza_sativa_XP_015634019             ----------------------------------------------------------SY
Brachypodium_distachyon_XP_003577118  ------------------------------------------------------------
Aegilops_tauschii_XP_020160360        ------------------------------------------------------------
Triticum_dicoccoides_XP_037485311     ------------------------------------------------------------

Sorghum_bicolor_XP_021304566          LLNGKPRFEEYTCDGDITTNENAAASSEAATGDQRIP------EVSLEVRGCRKETQ---
Setaria_italica_XP_022683723          LPNRTPRFEEYTCDGDIATNENAAASNDDRERDEAVCNEVDNVEIHLTVDRALTERDDGD
Oryza_brachyantha_XP_040378886        LSNKKPRFEEYACDIDISAKENTTAFNEGNEGSSAVCNKVGNGKIDLIVESTSKDRDHG-
Oryza_sativa_XP_015634019             LSNKKPRFEEYTCDLDISAKEKTTAFNEGSTGSLAVCNKVGNQQIDLVVKSFSKERGHGN
Brachypodium_distachyon_XP_003577118  -----SRFDEYSFDNDTSTKGKTVASNDASEGPAAVANEVDNMEIDLIVSSTSQERGHVD
Aegilops_tauschii_XP_020160360        -----SRFQEYICGNDASTKENSAASDDAR------PATVANVEIGLTASSISQERDHVD
Triticum_dicoccoides_XP_037485311     -----SRFQEHNCGNDASTNENSAASDDAR------PATVANVEIGLTTSSTSQERDHVD
                                                       ::: ::.* .:               :: * .     :

Sorghum_bicolor_XP_021304566          ------------------------------------------------HMSLTDKAAVDE
Setaria_italica_XP_022683723          IDINASLVLTMECTNDASNEEADNALSTSDQRTPVISLKTQGCWEKTGHVALNKKAAVDE
Oryza_brachyantha_XP_040378886        ------VTSTEEFTRDETSEQAGNQNEFI-------------------HPDVEDKEA---
Oryza_sativa_XP_015634019             IDLSASLTSIEETTRDKTSEDAGNQNEFI-------------------HSDAEYQEA---
Brachypodium_distachyon_XP_003577118  ISCNASFAPTEKCTSDETYKEAENQNDSM-------------------HPDVTEQEA---
Aegilops_tauschii_XP_020160360        IECNVSLAPAETYTGDETCKEAGNQNDTM-------------------HPDAREEDA---
Triticum_dicoccoides_XP_037485311     IECNVSLAPAETYTGDETCKEAGNQNDTT-------------------HPDAREDNA---
                                                                                      *     . *
```

```
Sorghum_bicolor_XP_021304566          EMPASVYLDMQNSLCLSNGTPILEEYTCDGYIPPNE------------------------
Setaria_italica_XP_022683723          GMPTSVCLDVQNSSYLSNETTRLEKNTCIGDTPTNE------------------------
Oryza_brachyantha_XP_040378886        ----ASHLVNSDSIY---------------------------------------------
Oryza_sativa_XP_015634019             ----GSHLVNSDSIYGMSTESGNQNEFIHADAEHQE------------VGSHVVNSDSNF
Brachypodium_distachyon_XP_003577118  ----GNHSVNSDLIC-NRSRDRMPSDLEDGN-----------------------------
Aegilops_tauschii_XP_020160360        ----GSHLFNSDWTC-TMCPDHMPNDSEGGNATSAENATMSPDNMPNDSEGGNVTSAKNA
Triticum_dicoccoides_XP_037485311     ----GSHLFNSDWTC-TMCPDHMPNDSEGGNATRAE----------------------ND
                                          .     .:

Sorghum_bicolor_XP_021304566          ---------DAAASNDDNERYIATSKEVNNMEKIVLVTGSPSRERGHDDIATDVAV----
Setaria_italica_XP_022683723          ---------GAAALNDNSERCTSVLEEVNS-VETSFIVGSVTRERGHDDVATNVSLTPTV
Oryza_brachyantha_XP_040378886        DKSTDNMTFGMGDGSANAGSSVGQG------SKEVLATVLPERANFSSD-----------
Oryza_sativa_XP_015634019             DMSTDNMICEMGDGSANAGSAVSQG------SKEVLATVLPERANLSPD-----------
Brachypodium_distachyon_XP_003577118  ---------------TNAGNSTDV-------ALCHLATAQPERVNCCSEIP---------
Aegilops_tauschii_XP_020160360        TMSPDHMSPDNMPNDSEGGNATGAENSVELLAKYPLAIVPPENANCCSEIP---------
Triticum_dicoccoides_XP_037485311     TMSPDHMSPDNMPNDSEGGNATSAENSVELLGKYPLAVVLPESANCCSEIP---------
                                                      :                  :        .   :

Sorghum_bicolor_XP_021304566          -----SELVHSTPATGTYRKKTPVASLKSQGSWKENQPVASNKKMKLID----------P
Setaria_italica_XP_022683723          ECTNDAVNEGVDNTSLLGCKTTPVASLKSQGCQEKQQHIPSNEKQ---------------
Oryza_brachyantha_XP_040378886        -----DCLDNILPIS----TCNSNNCVENQTCLEIAQHITLNEEVVQNEDMSTSVHSDGE
Oryza_sativa_XP_015634019             -----SCLDNILPIS----TCNSNNCLENQGFPEIAQHMTLNEEVVPNEDISTSVHSDVE
Brachypodium_distachyon_XP_003577118  -----GALQNIQPLS---NQRNPVASLKNQSHPKRTEDISLNQKAVPIEDTSTSIRSHVL
Aegilops_tauschii_XP_020160360        -----GASQSVEPSS---YQSTPVASLKNQHCLETTEHITLTQKAVSNEDTPSSIHSDVQ
Triticum_dicoccoides_XP_037485311     -----GASQSVEPSS---YQSTPVASLKNQHCLETTEHITLTQKAVSNEDTPSSIHSDVQ
                                                    :      ..  .::.*   :  : :. .::

Sorghum_bicolor_XP_021304566          CLGKQHVGRPKKRISPHAKCQSA-------------------------------------
Setaria_italica_XP_022683723          -----PSGPPKKQRSALEKLRGA-------------------------------------
Oryza_brachyantha_XP_040378886        SLRN-PAGPAKEQISKSNVLQGAQRWPKKHVGSAQEQRPER-------------------
Oryza_sativa_XP_015634019             SLGN-PVGPAEVQRSECDILQGAPRSPKQNVGSAQEQRPEQSMSQGAARSPMIRTPIPDG
Brachypodium_distachyon_XP_003577118  SSEK-TVGPSKKQRSAQDKL----------------LSP---------------------
Aegilops_tauschii_XP_020160360        SQEKQTVGSAEKRRSAKQVL----------------ERP---------------------
Triticum_dicoccoides_XP_037485311     SQEKQTVGSAEKRRSAKQVL----------------ERP---------------------
                                             * .: . *
```

```
Sorghum_bicolor_XP_021304566          -------------------------TRSPGTRNPASYVLWSPLTRDKATSLSMSTPEDLE
Setaria_italica_XP_022683723          -------------------------TRSPLT-SPVPYAHDSPLTRGRTTSLSMSTPESLK
Oryza_brachyantha_XP_040378886        -------------------YMSPGATRSPMIRTAIPYAHCSPLTRAKAKSSSVSTPESLE
Oryza_sativa_XP_015634019             APSLRNQHLGSAQEQRSEHFMLKGATRSPMIRTPIPYGHYSPLTRGKAKSSSVSTPESLK
Brachypodium_distachyon_XP_003577118  -------------------------ARLRGTRNPISYVHHSPHTRGKAQSLSISTPESLE
Aegilops_tauschii_XP_020160360        -------------------------TRSPMTRTSAPYGHKSRLTRSRAQSLSISTPECLK
Triticum_dicoccoides_XP_037485311     -------------------------TRSPMTRTSAPYVHKSRLTRSRAQSLSISTPESLK
                                                               :*     .. .*   *  ** .: * *:**** *:

Sorghum_bicolor_XP_021304566          LKRSRSGRVIVPKLDNWCQTIVYGRDGLIAAVIGLDSPALP-KWSESKTDRRKKRKTK--
Setaria_italica_XP_022683723          LRKTRSGRVVVPTLDKGCQRIVYDMDGAIVGVVGLDSPS-P-KGSKLETNARKKKNAEPA
Oryza_brachyantha_XP_040378886        LRRTRSGRVVVPPLDPGRQRIIYDKDGLVSGVAGLELQS-PLKGSKSRTPAKKRRAH---
Oryza_sativa_XP_015634019             LRRTRSGRVVVPTLDPGCQRIVYDRDGLVSGVAGLEFESPPLKGNESRTPESKRRVR---
Brachypodium_distachyon_XP_003577118  MTRTKSGRVVVPPLDLGCERILYGNNHLVLGVAPVKLHSPPIKGSKPETPARKRRAR---
Aegilops_tauschii_XP_020160360        MRRTKSGRVVVPQLDPGSSRIVYDNNGLISGVA-------PVTGNKSVKPARKTRGPL--
Triticum_dicoccoides_XP_037485311     MRRTKSGRVVVPQLDPGRSRIVYDNNGLISGVA-------PVDGKKSARPAKKTRGPL--
                                      : .:.****:** **   . *:*. :  : .*        *   .:      * .

Sorghum_bicolor_XP_021304566          ------------------
Setaria_italica_XP_022683723          VAASYKLKTYARKKRRAE
Oryza_brachyantha_XP_040378886        ------------------
Oryza_sativa_XP_015634019             ------------------
Brachypodium_distachyon_XP_003577118  ------------------
Aegilops_tauschii_XP_020160360        ------------------
Triticum_dicoccoides_XP_037485311     ------------------
```
