# Supplementary material for: Recurrent Plant-Specific Duplications of KNL2 and its Conserved Function as a Kinetochore Assembly Factor
Source: Mol Biol Evol. 2022 Jun 7;39(6):msac123. doi: 10.1093/molbev/msac123 (PMC9210943; doi:10.1093/molbev/msac123)
Supplement: msac123_Supplementary_Data [file msac123_supplementary_data.zip › Supplementary_FIle_6_delta_KNL2_alignment.pdf]

δKNL2

SANTA domain   conserved hydrophobic motifs   CENPC-k-like   imperfect_duplications

```
Aegilops_tauschii_XP_020165136      MRTRSMASKPEPVPSTHGTAARAPAPASVSASTHGTAARAAARASVSASTHGKAARAPAR
Triticum_aestivum_SPT18119          MRTRSMASKPEPVPSTHGTAARAPAPASVSASTHGTAARAAARASVSASTHGKAARAPAR
Oryza_brachyantha_XP_015691873      ------------------------------------------------------------
Oryza_sativa_XP_015626456           -------MATQPRAGDGAAAAA--------------------------------------
Setaria_italica_XP_012703033        -------MASEPPPGSTGEATRTP------------------------------------
Sorghum_bicolor_KAG0543345          -------MPSQPAPDSTGGSPRIPVASAEAVRRVVDFFKTNGKLRSVTGAAVSSPLLP--
Zea_mays_NP_001145421               -------MPSQPQPSSIGGTSQTP------------------------------------

Aegilops_tauschii_XP_020165136      ASVSA-----------------STHGTAARAPAPASVSASTHGTAARAPAPASVSASTHG
Triticum_aestivum_SPT18119          ASVSASTHGTAARAPTPASVSASTHGTAARAPAPASVSASTHGTAARAPAPASVSASTHG
Oryza_brachyantha_XP_015691873      ------------------------------------------------------------
Oryza_sativa_XP_015626456           ------------------------------------------------------------
Setaria_italica_XP_012703033        ------------------------------------------------PAP---------
Sorghum_bicolor_KAG0543345          -----------------RGAATPPLAGMPSQPPQGSAGGSPRISAPFQPAPDYTGGSHRT
Zea_mays_NP_001145421               --------------------------------------------APFQTAPVFTGGSPRT

Aegilops_tauschii_XP_020165136      TAARAPAPVSVSAPTHGTAAPAPPPASVRAPTYCATVQRCVALLDWWLVRGQG--GKIRV
Triticum_aestivum_SPT18119          TAARAPAPVSVSAPTHGTAAPAPPPASVRAPTYCATVQRCVALLDWWLVRGQG--GKIRV
Oryza_brachyantha_XP_015691873      -----------------------------------------------MQRVEGEEGKVRV
Oryza_sativa_XP_015626456           ---------------AAKEAPA-----------VSYLQACVELDDWWLERVEGEEGKVRV
Setaria_italica_XP_012703033        ---------------SAAAAPR-----------VSYVQQCVVLVDWWLERVEGEEGKIRV
Sorghum_bicolor_KAG0543345          PAPGSIGVVPRIPELSAQAVDR-----------IA--RKCIILVDWWLERVEGEEGKIRV
Zea_mays_NP_001145421               -----------LATLSAQSVPR-----------VT--RRSIALVDWCLERVEGEEGKIRV
                                                                                   : * :*  **:**

Aegilops_tauschii_XP_020165136      AGYIDNV---------------EKNRAGRVFSSGSITVRHADGTLETADNKIVLTRGPLN
Triticum_aestivum_SPT18119          AGYIDNV---------------EKNRAGRVFSSGSITVRHADGTLETADNKIVLTRGPLN
Oryza_brachyantha_XP_015691873      VGSDTTT-----------------SRACRKFTSASIKTRHANGDIETADSVIIMTVGPPD
Oryza_sativa_XP_015626456           VGSNTTT-----------------SRAGRRFTSASIKTRHASGDLETEDGIIIMIARPPN
Setaria_italica_XP_012703033        AGIASTA----QMRKGASSSTGNRNVAGRVFRSAAIGRRHDQHAIETEDGYKIQIGRLLN
Sorghum_bicolor_KAG0543345          AGTTFTPRMAEQMRKGASSS--NMRMAVRVFRSSAIVKRHDYTSIESEDGYQIEIGHCLN
Zea_mays_NP_001145421               AGTTYTPQTSPQTRREASSSKGSRKVAGRVFRSSAIVRRHDHFRIMSEDGYLIRIGCLLN
                                    .*   .                    * * * *.:*  **    : : *.  :      :
```

```
Aegilops_tauschii_XP_020165136      IEQMHWNGFSREVSEQFRLGFPIQWEKYANSNMKQANEHI--------LSPAKSTEYCVE
Triticum_aestivum_SPT18119          IEQMHWNGFSREVSEQFRLGFPIQWEKYANSNMKQANEHI--------LSPAKSTEYCVE
Oryza_brachyantha_XP_015691873      ISKMHQNGFPHEVSKYFMLGFPVEWEKYINASMSEMNDQP--------QSPLKSTEYYIE
Oryza_sativa_XP_015626456           ISKMHLNGFPDEVSKHFSLGFPVQWENIINANMAEMNKQP--------QSPLKSTEYYIE
Setaria_italica_XP_012703033        VPRTRDNGFPEKVCKCFEFGFPIQWLKLVNPKMEQQNEQA--QSESTADAPRHSVKYWME
Sorghum_bicolor_KAG0543345          IPKTRENGFSEEVCESFDFGFPDLWQRLVNPKMVPDDEHALSPSETTTGPPSPSVEDYMA
Zea_mays_NP_001145421               IPKTRNNGFSEEVCECFEFGFPIQWHRLVNPKMVPDNKHALSPSETTASAPSHSVGYYME
                                    : . . ***. :*.: * :***  * .  *..*   :.:          .*  *.   :

Aegilops_tauschii_XP_020165136      KFLRSSFANSMEHTLTGFDFRTSKESTGNTDGPGLPNYV-----KPRIQEPSGNSVGYDN
Triticum_aestivum_SPT18119          KFLRSSFANSMEHTLTGFDFRTSKESTGNTDGPGLPNYV-----KPRIQEPSGNSVGYDN
Oryza_brachyantha_XP_015691873      KFLQGNLKYSMGL-FAWDYLNTSERSTSDTDRFPSQRHSNSSNRRHNFEDY---------
Oryza_sativa_XP_015626456           KFLRGNLKYSMGL-FSWDGLNIYQGSRSDADRFPSERLSNSSNGRPTVEDPTANTDCNVN
Setaria_italica_XP_012703033        EFLSDDLTNLKKYASEENDSYSSAGYTSNTDGPAIQSLSNLPDGNA--------------
Sorghum_bicolor_KAG0543345          KFLSDSFSSKIGFDFTENDFDS--------------------------------------
Zea_mays_NP_001145421               KFLSDSFSNSNRYSFTENDSYTSVVCTGSRNGLTTQTLSNLPDDNA--------------
                                    :** ..:

Aegilops_tauschii_XP_020165136      SVSNMAASEGLCNDRMGTPDESFEDP----------------------------------
Triticum_aestivum_SPT18119          SVSNMAASEGLCNDRMGTPDESFEDP----------------------------------
Oryza_brachyantha_XP_015691873      ------------------------------------------------------------
Oryza_sativa_XP_015626456           FMGTLATSEEFCTGRMDMPEEPRATPSETCGNDQENNQHLCMLMNTCENG----------
Setaria_italica_XP_012703033        --GNMAASGGLYGGRTNMPGKPLARPRETSCSGQESDQHESMQIDTSEQGLDNHSISSVS
Sorghum_bicolor_KAG0543345          ------------------------------------------------------------
Zea_mays_NP_001145421               --GNITVSWGLYGLGMDMSEKPWTPPAEACNNRQESDQHESMQIDACKQELVNRSMSSVS

Aegilops_tauschii_XP_020165136      --------GPGETCNGQA------------------------SRADNSHEDIQTDASGQR
Triticum_aestivum_SPT18119          --------GPGETCNGQA------------------------SRADNSHEDIQTDASGQR
Oryza_brachyantha_XP_015691873      -----AFVQYQESSAGPSVDTA------------KNARNQVEQDADIQQENMHRYSSEQQ
Oryza_sativa_XP_015626456           -----NKVQHGTSSVGPSVVPA-------EKYVRS----KAEQDALLVNDSTSH------
Setaria_italica_XP_012703033        VNQNTGSFCPNSKVDDSILATSKIMSVEKESYRRRVGSSKADEDADIQHENMQSCSNEHE
Sorghum_bicolor_KAG0543345          -----GSISSDSKVDGNILAPSKISSVVNEGYRSTVGCGQAKKDANIQQENMPSCSSEHA
Zea_mays_NP_001145421               VKQSISSISPNSKVDGNRIVPSKIMSVVNESYRSTVGCGQAEEDVGIQQEKKHSCSSEHG
                                                .  .                          . .   ::.

Aegilops_tauschii_XP_020165136      IVTHSADSTLVN---NDIDKIEEERGSSKLGNSSVCPGTEHVSEALNQGASPEHGSVQCS
Triticum_aestivum_SPT18119          IVTHSADSTLVN---NDIDKIEEERGSSKLGNSSVCPGTEHVSEALNQGASPEHGSVQCS
Oryza_brachyantha_XP_015691873      IVTHSNESALINNCTNEVSSGLEDWETPKCGKASTCQGTKDALDITTEGMNPQLGVNQGS
Oryza_sativa_XP_015626456           -----------------VSSVLGDCATPKCGKSLTHLGTKDALE-TNEGMNPQFGVPQGS
Setaria_italica_XP_012703033        IVTLPIDSAIVNENPNSTSSDLEKPGTPKCGKASMNLGSTDALELPTERMTPQFGAVQGS
Sorghum_bicolor_KAG0543345          MV-----------------------TPKFGKDSVNLGTTDALELPTEGMTPKFGAIRGS
Zea_mays_NP_001145421               MVTLSI---------NCTSSQLGAPGIPKFGKDSVNLWTTDALELSTEGMTPKIGA----
                                                               .* *:      :  . :  .:  .*: *
```

```
Aegilops_tauschii_XP_020165136      -----RRLRSGKVYGMSNGASLKRRYSKRKTMQHGTLSMKVIPTEETTPPAGPTCHKKAG
Triticum_aestivum_SPT18119          -----RRLRSGKVYGMSNGASLKRRYSKRKTMQHGTLSMKVIPTEETTPPAGPTCHKKGG
Oryza_brachyantha_XP_015691873      EDNTVRRLRNGKVFGMSSSASLKKVVCKRGRMQVKTFLEKIIPIEDVTHPADLISREKVG
Oryza_sativa_XP_015626456           EGSTVRRLRNGKVIVISTSASTKK-VYKRARMQDNTFSENVIPNKNVTCPTGLISQENVG
Setaria_italica_XP_012703033        EDSPVRRLRSGKVFGMPSGGLMKS-GHKKRKIQHEASSQNMIPNEGDTSTADLTSHENDS
Sorghum_bicolor_KAG0543345          EDSIGRRLRSGKVL--PIGGPMKK----QKKIQQQMV------NQGATPAADLTSHENDF
Zea_mays_NP_001145421               -DSTDRRLRSGKILGMPSGGLMNR-GHKLKKIKQEASSKQMV-NQGATCTVDLTSHENDF
                                         ****.**:  :. ..  :        ::           :  * ...  ..::

Aegilops_tauschii_XP_020165136      RGIHGLCSAGLM---------------------------
Triticum_aestivum_SPT18119          SVAQITALDKLQSNDSGRK--G-HGRPRKKARR------
Oryza_brachyantha_XP_015691873      SVA-VSMSAKLQIHDSPRK--G-PGRPSKRGKRKRS---
Oryza_sativa_XP_015626456           SVA-VTAAAKLQIHDTPRK--GRRGRLRKSEKRKRS---
Setaria_italica_XP_012703033        SAAGGVTKDKQESHDSHRGISAKKAKKKRESSKLFWNWC
Sorghum_bicolor_KAG0543345          SAAEVVVKENLGSDDSCGKVTGQGRIAEGKGKRKRKRV-
Zea_mays_NP_001145421               SAAEIVVEEKLESHSSCLK--GRGGPAKGKRKRERW---
```
